# The Impact of Exercise on Intervertebral Disc Health: A Systematic Review and Meta-Analysis

**DOI:** 10.1007/s40279-025-02336-w

**Published:** 2026-03-16

**Authors:** Claire L. Samanna, Patrick J. Owen, Ulrike H. Mitchell, Katja Ehrenbrusthoff, Tobias Saueressig, Eva Moreira, Nitin K. Arora, Niamh L Mundell, Jamie L Tait, Lars Donath, Vera Karner, Daniel L. Belavý

**Affiliations:** 1 Eastern Health Clinical School, Monash University, Melbourne, Victoria Australia; 2 Eastern Health Emergency Medicine Program, Melbourne, Victoria Australia; 3 Department of Exercise Sciences, Brigham Young University, Provo, UT USA; 4 Department Für Pflege-, Hebammen und Therapiewissenschaften, Division of Physiotherapy, Bochum University of Applied Sciences, Bochum, Germany; 5 Physio Meets Science GmbH, Leimen, Germany; 6 Institute of Physical Activity and Nutrition, School of Exercise and Nutrition Sciences, Deakin University, Geelong, Victoria Australia; 7 Institute of Exercise Training and Sport Informatics, German Sports University Cologne, Cologne, Germany; 8 University of Applied Sciences for Health Professions Upper Austria, Linz, Austria

## Abstract

**Background:**

The structure and function of human tissue, such as bone, muscle and tendon, can be improved with targeted exercise training. However, the effects of exercise training on intervertebral disc tissue remain unclear.

**Objective:**

We aimed to examine the impact of physical loading exposure (exercise training, sport and physical activity) on intervertebral disc (IVD) health compared to non-physical loading (or lower volume of the same physical loading) controls.

**Methods:**

We conducted a systematic review and meta-analysis. Seven electronic databases (PubMed, CINAHL, SPORTDiscus, EMBASE, CENTRAL, Web of Science and Scopus) and two trial registries (World Health Organization International Clinical Trials Registry Platform and National Institutes of Health) were searched from inception to 3 June, 2025. Forward and backward citation tracking was conducted for included reports. Certainty of evidence was assessed using the Grading of Recommendations Assessment, Development and Evaluation criteria (GRADE). Risk of bias was assessed using Cochrane RoB2 and Johanna Briggs Institute critical appraisal checklists. We included randomised controlled trials (*n* = 2), cohort (*n* = 9) and cross-sectional studies (*n* = 28) of any physical loading compared to controls with continuous measurements of IVD health (e.g. T2-relaxation, IVD height) via magnetic resonance imaging and/or categorically graded measures of IVD degeneration (e.g. Pfirrmann grade).

**Results:**

Forty-five reports of 39 studies (participants: 4152) were included. The pairwise random-effects meta-analysis estimated the standardised mean difference (Hedges’ *g*) of continuous outcomes for combined physical loading and independent subgroups and odds ratios (Paule–Mandel estimator) of categorical outcomes for combined physical loading only. A meta-analysis revealed upright bipedal loading (mostly running; Hedges’ g [95% confidence interval] 0.31 [0.12, 0.50]; *P* = 0.002; *n* = 7, GRADE: very low) was associated with better IVD health; however, no other subgroup of physical loading was associated with better or worse IVD health. Combined physical loading revealed greater odds of IVD degeneration via reduced signal intensity (odds ratio [95% confidence interval] 2.80 [1.53, 5.11], *P* = 0.001; *n* = 5, GRADE: low); however, no other measure of IVD degeneration was significant.

**Conclusions:**

Running was the only physical loading exposure associated with better IVD health. The mixed results from the combined physical loading analyses suggest that the type of physical loading plays a role in IVD health. As our estimates rely on observational data, prospective running interventions that examine the causal effect on IVD health appear warranted.

**Clinical Trial Registration:**

The review was prospectively registered with International Prospective Register of Systematic Reviews (PROSPERO) [CRD42022366391]. See Supplement P of the Electronic Supplementary Material for protocol deviations since registration.

**Supplementary Information:**

The online version contains supplementary material available at 10.1007/s40279-025-02336-w.

## Key Points

**Table:** 

Physical loading through running was associated with better lumbar intervertebral disc health compared with control groups who did not participate in the same physical loading.
No other form of physical loading exposure was significantly associated with better or worse intervertebral disc health.
Continuous intervertebral disc outcomes had greater sensitivity for detecting intervertebral disc health compared with categorical outcomes.

## Introduction

Spinal intervertebral discs (IVDs) are integral to maintaining spinal stability and mobility, and structural changes are recognised as contributing factors in the development of back pain [1]. In particular, deterioration in IVD health, such as reduced height, less hydration and increased fibrous tissue, is more commonly observed in individuals experiencing low back pain (LBP) [2]. Clinical exercise protocols for LBP primarily target muscle systems, with no established treatment for managing LBP arising from poor IVD health. Therefore, the potential to ‘strengthen’ the IVD via exercise training is of clinical relevance [3–5]. However, based on the slow turnover rates of IVD components such as aggrecan, which renew over decades [6], it is possible that IVD metabolism is too delayed to respond to the mechanical stimuli associated with exercise training. However, this theory has since been challenged by basic science. Dynamic axial compressive loading applied to IVD cells in vitro led to positive changes in IVD metabolism [4]. This was further supported by animal research [7, 8] where 3 weeks of running exercise led to anabolic adaptation in the IVD [9]. Thus, evidence from cell and animal research suggests that improving IVD health via exercise training is plausible.

The concept of human tissue adapting to physical loading via exercise training, sport participation or physical activity is well established [10–15]. Wolff [10] described the theory of bone adaptation to loading. In the intervening years, research determined that high-impact exercise training protocols, such as those involving jumping and bounding, were beneficial for bone mass accrual (osteogenic) [11]. Similarly, exercise training protocols can also have positive effects on muscles, such as increasing the cross-sectional area of muscle fibres [13], and tendons, by increasing the size and number of collagen fibrils [14, 15]. The extent of soft-tissue remodelling is dependent on the magnitude of the stressor, including intensity, duration and frequency [12]. However, whether exercise training can elicit similar adaptations in IVD tissue remains less established [5].

Studies assessing the impact of physical loading on IVD tissue via magnetic resonance imaging (MRI) date back to the 1980s [16]. Greater IVD degeneration was frequently observed in elite athletes compared with non-athletes [17]. However, many of these studies had small sample sizes for each sport and measured prevalence of IVD degeneration only, which is unreliable owing to the confounding influence of age [18] and LBP status [2]. A U-shaped relationship has been proposed, indicating that poorer IVD health occurs at the extreme ends of the exercise training spectrum, specifically in individuals who are highly sedentary and in those exposed to high volumes of explosive physical loading, as seen in athletic populations [5]. Moderate exercise training appears to be associated with increased IVD health [5], albeit what constitutes moderate exercise training in the context of IVD health is not clearly defined. A narrative review by Belavý et al. [5] noted that no human studies had prospectively examined whether exercise training affects IVD tissue. Since then, several groups have established initial proof-of-principle that exercise training may be linked with IVD health. These include the first randomised controlled trial (RCT) with a 6-month strength and conditioning intervention [19] and a more recent RCT protocol proposing a 3-month running intervention [20]. These recent studies, along with several other observational works, have employed methods to detect an improvement in IVD health rather than the degree of degeneration, which was previously the primary focus. To date, only one systematic review has examined running studies [21]; however, no meta-analysis has been conducted. Therefore, there is a need to systematically synthesise data from studies involving various forms of physical loading through a meta-analysis, to address the limitations of small sample sizes at the individual study level.

Historically, categorical radiological grading schemes were used to assess the degree of IVD degeneration, such as the five grades proposed by Pfirrmann et al., which range from healthy to severe degeneration [22]. However, categorical grading schemes are limited by the extensive training required to implement these schemes, inconsistent reliability because of inter-rater subjectivity [23], and the limited sensitivity to detect subtle matrix-level changes, such as hydration. Subsequently, a range of continuous IVD methods have been refined. Examples include IVD T2-relaxation (T2), which is a value representing the water content and structural make-up of the inner matrix [24], and the apparent diffusion coefficient, which indicates the speed of water molecule diffusion [25]. Therefore, our review primarily aimed to determine whether exercise training, sport participation, and physical activity improve continuous measures of IVD health, compared with controls who do not participate in the same physical loading. Primary sub-aims examined the same continuous IVD measures across subgroups of physical loading, grouped by loading forces at the lumbar spine, aerobic endurance training, and the same physical loading at a lower volume. Secondary aims examined differences in the categorical grading of IVD health with combined physical loading. The final aim compared the sensitivity of continuous measures of IVD health to categorical grading schemes.

## Methods

The current review was conducted and reported in accordance with the Preferred Reporting Items for Systematic Reviews and Meta-Analyses guidelines [26, 27] and checklist [28]. The review was prospectively registered with PROSPERO (CRD42022366391).

### Eligibility Criteria

Inclusion criteria followed the Participants, Exposure, Comparators, Outcomes, and Study design (PECOS) framework [29]. *Population*: human (no restrictions on medical diagnosis, age, sex and race). *Exposure*: physical loading via exercise training (an activity requiring physical effort with the intention to improve health and fitness) [30], sport participation (an activity requiring physical exertion and skill involving competition of a team or individuals and physical activity) [31] or physical activity (any bodily movement produced by skeletal muscle contracting [30]; see Supplement A of the Electronic Supplementary Material [ESM] for exposure subgroups). Studies on the acute effects (< 1 week) of physical loading were excluded. Studies examining the effect of strict inactivity, bed rest, spaceflight or disuse/immobilisation were excluded. Studies that assessed IVD health across different occupations but did not explicitly measure physical activity were excluded. *Comparator*: the reference group as defined by the authors (or implied if the control was not overtly stated, yet met one of the following requirements), for example non-athletes or sedentary, people without participation in the same physical loading exposure as the intervention group, athletes who do the same activity but at a lower training load or a reference group for logistic regression where the reference group has a value of one for the odds ratio (OR). *Outcome*: for the primary research questions, outcomes that quantified lumbar IVD health on a continuous scale via MRI were included. These could consist of any continuous measures of IVD physiology (e.g. T2, signal intensity, apparent diffusion coefficient) and/or structure (e.g. height, width, volume). For the secondary research question, outcomes reporting categorical measures of lumbar IVD degeneration were included (Pfirrmann scale or other ordinal scales of degeneration) [22]. Where studies did not report data on the lumbar IVD separate from other radiological abnormalities of the spine, such as osteophytes and Schmorl nodes, these studies were excluded. The identified full-text reports were searched for research question three, the use of continuous and categorical IVD measures within a single study. *Study design*: any study design that reported data in aggregate form (e.g. RCT, cohort study, cross-sectional) as a full peer-reviewed journal publication (i.e. grey literature, including theses and conference abstracts, was excluded). Possibly relevant trial registrations and/or published trial protocols were also included. Authors were contacted to clarify the eligibility criteria for the systematic review and to request data, if necessary. Case-series studies were excluded. No restrictions were made on language. Retracted studies were excluded, and this was verified by automated cross-checking of the Retraction Watch database (v.1.0.7.0; The Centre For Scientific Integrity, New York, NY, USA) via citation software Zotero (v6.0.27; Digital Scholar, Vienna, VI, USA).

### Information Sources and Search Strategy

Electronic database search of PubMed (no limits), EMBASE (via Ovid; limits: exclude MEDLINE records), CINAHL (via EBSCOhost; no limits), SPORTDiscus (via EBSCOhost; no limits), CENTRAL (limits: trials), Web of Science (no limits) and Scopus (limits: not animals) was conducted from inception to 3 June, 2025. The full search strategy is presented in Supplement B of the ESM. Unpublished and ongoing trials were searched via the US National Institutes of Health (https://clinicaltrials.gov/) and World Health Organization International Clinical Trial Registry Platform (https://trialsearch.who.int/Default.aspx), which collates 17 international trial registries. Further, prior relevant review articles [3, 5, 32–34] were identified via PubMed and Google Scholar. Backwards (i.e. reference list) and forward (i.e. GoogleScholar citations) citation tracking was applied to the included reports.

### Selection Process, Data Collection Process, and Data Items

Search outputs were exported to Covidence (Veritas Health Innovation, Melbourne, VIC, Australia), and duplicate records were automatically removed. Prior to independent title and abstract screening, a pilot screening was conducted with 100 randomly selected records by nine authors to ensure a consistent approach (CLS, EM, UHM, NKA, NLM, PJO, VK, DLB, TS). Similarly, a pilot of ten randomly selected records was completed by nine authors (CLS, EM, UHM, NKA, NLM, PJO, VK, DLB, TS) prior to full-text screening. A pilot extraction of five randomly selected records was conducted by five authors (CLS, NKA, PJO, DLB, TS) prior to data extraction for refining the extraction sheet. During the pilot, conflicts were resolved via discussion among the entire authorship team. Subsequently, four independent authors (UHM, EM, VK, TSK) screened in duplicate for the title/abstract and full-text stages. Two independent authors (CLS, NKA) completed the data extraction in duplicate. All disagreements were adjudicated by three independent authors (PJO, NLM, DLB). The following study information was extracted: publication information (author, year, funding, conflict of interest), study design, study demographics (intervention and control group inclusion/exclusion criteria, participant age, sex, number and percentage with LBP), continuous outcomes reflective of IVD health and categorical outcomes reflective of IVD degeneration (includes prevalence outcomes). Multiple reports were combined into a single study and were verified by linking author names, trial registrations, population and timeframe. Authors were contacted where uncertainty persisted [35]. In cases of RCTs/cohort studies, baseline and the final follow-up measures closest to the end of the exposure for each relevant IVD outcome were recorded. Lumbar IVD health outcomes were extracted as the number of participants, mean and standard deviation. For the studies that did not report the mean and the standard deviation, alternate measures of central tendency (e.g. median) and spread (e.g. interquartile range) were used to compute mean and standard deviation using standard data-converting equations (Supplement C of the ESM) [36]. Where data for lumbar levels could not be differentiated from other regions of the spine (e.g. thoracic or whole spine), the narrowest available average data were recorded (e.g. thoracolumbar in preference to whole spine, where possible). Data presented in the figures were extracted using WebPlotDigitizer (v4.6; Ankit Rohatgi, Pacifica, CA, USA), a method demonstrating high interrater reliability and validity [37]. Where the data could not be extracted, data requests were made to corresponding authors on three occasions, over a 3-week period (Supplement D of the ESM). Both data extractions were uploaded to a data share repository (https://osf.io/IVDEX) [38].

### Study Risk of Bias Assessment

Two independent authors, with no authorship conflicts for the included studies (CLS, NKA), completed a risk of bias assessment for all included studies. A third independent author (NLM) adjudicated where necessary. Cross-sectional and cohort studies were assessed with the Johanna Briggs Institute Checklist for Analytical Cross-sectional Studies and Cohort studies [39], respectively. Studies were classified as being low risk of bias if > 70% of the questions had a ‘yes’ response, high risk of bias if < 50% of the questions had a ‘yes’ response, and the remaining studies were classified as having ‘some concerns’. Randomised controlled trial primary outcomes were assessed with the ‘Risk of Bias 2’ tool and classified as having ‘low risk’, ‘some concerns’ or ‘high risk’ according to the recommended algorithm [40].

### Effect Measures and Synthesis Methods

A pairwise, random-effects, restricted maximum likelihood meta-analysis estimated the standardised mean difference (Hedges’ *g*) of several MRI outcomes characterising IVD health between the physical loading exposure and control groups for continuous outcomes. Crude data, unadjusted for confounders, were used in the current meta-analysis unless only adjusted data were available [41]. Physical loading exposure subgroups are presented in Table 1 and classified in Supplement A of the ESM. Where studies had multiple physical loading exposures, only the participant data with the relevant exposure were pooled for an effect size. A random-effects meta-analysis of OR estimated the effect of the prevalence of individuals with degeneration, using the Paule–Mandel estimator for between-study variance *T*^2^.

**Table 1:** Physical loading exposure subgroups

Physical loading exposure	Description
Combined physical loading	All physical loading with appropriate data available; studies with multiple physical loading exposures were pooled into a single effect size
Upright bipedal	Walking, running, skiing, sports involving predominantly running (basketball, soccer)
Non-upright non-contact	Cycling, swimming, rowing
Explosive loading in end range	Weightlifting, martial arts, baseball, gymnastics
Aerobic	Running, walking, skiing, rowing, swimming
High load vs low load	Comparison of two groups within the same sport at different training volumes

Hedges’ g effect sizes were interpreted as: *g* = 0.2 (small), *g* = 0.5 (medium) and *g* = 0.8 (large) [42], and OR as: OR = 1.5 (small), OR = 2.5 (medium), OR = 4 (large) and OR = 10 (very large) [43], both with 95% confidence intervals (CIs). The Hartung–Knapp–Sidik–Jonkman method was employed in any meta-analysis with fewer than five studies, as per recommendations [44]. Measures of heterogeneity included *I*^2^ and 95% prediction intervals [45]. The prediction interval offers an anticipated range of a certain probability (e.g. 95%) that the genuine effect size in a new study will lie within; however, it is only applied where three or more studies were included within the meta-analysis [45]. Where ten or more studies were included, random-effects meta-regression assessed the heterogeneity of participant characteristics associated with IVD health (e.g. baseline mean age, sex and LBP) [46]. The random-effects meta-regression model used the standardised mean difference for continuous outcomes with an identity link function and OR for binary outcomes with a logit link function. Between-study variability was accounted for with random effects (i.e. the standardised mean difference variability not explained by the outcome variable) and unexplained variability by residual variability, both assumed to follow a normal distribution with a mean of zero and Tau [47].

All statistical analyses were completed in Stata (v17; StataCorp, College Station, TX, USA) with an *α* of 0.05 adopted for all analyses. Data and statistical codes used are recorded in Supplement E of the ESM. Prior to analysis, (1) reverse scale data (i.e. where smaller values indicated greater IVD health) were multiplied by − 1; (2) repeated outcome data were removed, for example, preferencing outcome data for each lumbar level or by IVD subregion instead of average lumbar region, and (3) where multiple outcomes in a study reflected different aspects of IVD health, a synthetic effect size was computed for each study using the mean effect size and variance calculated using an assumed correlation between outcomes of *ρ* = 0.8 [48]. Further details about the data handling process are available in Supplement C of the ESM.

A sensitivity analysis examined the influence of individual studies via leave-one-out meta-analysis, where there were more than two studies [49]. Synthetic effect sensitivity analyses were also conducted using *ρ* = 0.0, *ρ* = 0.2, *ρ* = 0.4, *ρ* = 0.6 and *ρ* = 1.0. We also performed a random effects meta-analysis with robust variance estimation [50] and a range of correlation values (*ρ* = 0.0, *ρ* = 0.2, *ρ* = 0.4, *ρ* = 0.6, *ρ* = 0.8 and *ρ* = 1.0) when ten or more studies were included within the meta-analysis, and with a *ρ* = 0.8 where there were fewer than ten studies to test assumptions for normality [51].

### Reporting Bias Assessment

Egger’s *P*, funnel plots and the trim-and-fill method were interpreted to assess publication and small study bias, where at least ten studies were included [52]. As these statistical tests cannot differentiate between small-study effects and reporting bias [53], the existence of potentially unpublished trials was further assessed via our search of trial registries.

### Certainty Assessment

Certainty of evidence of each pairwise meta-analysis [54] was assessed via the Grading of Recommendations, Assessment, Development, and Evaluations (GRADE), where the certainty began as “low” given the inclusion of observational studies. Implementation of each GRADE criterion and adoption of the Confidence in Network Meta Analysis (CINeMA) approach [55] for imprecision and inconsistency are outlined in Supplement F of the ESM. Given the lack of established clinically meaningful cut-offs for outcomes of IVD health, the region of equivalence was defined as *g* = − 0.5 to *g* = 0.5 [42] and OR = 0.8 to OR = 1.25 [55]. Two independent authors (CLS, TS) implemented the GRADE assessments, and a third independent author (NLM) adjudicated where necessary.

### Equity, Diversity and Inclusion Statement

Our author team comprised six women and six men, including junior, mid-career and senior researchers from a variety of disciplines and located across four countries in Australasia, North America and Europe. The inclusion criteria were not limited by LBP status, and we did not purposefully include people from marginalised communities.

## Results

### Study Selection

**Fig. 1:**
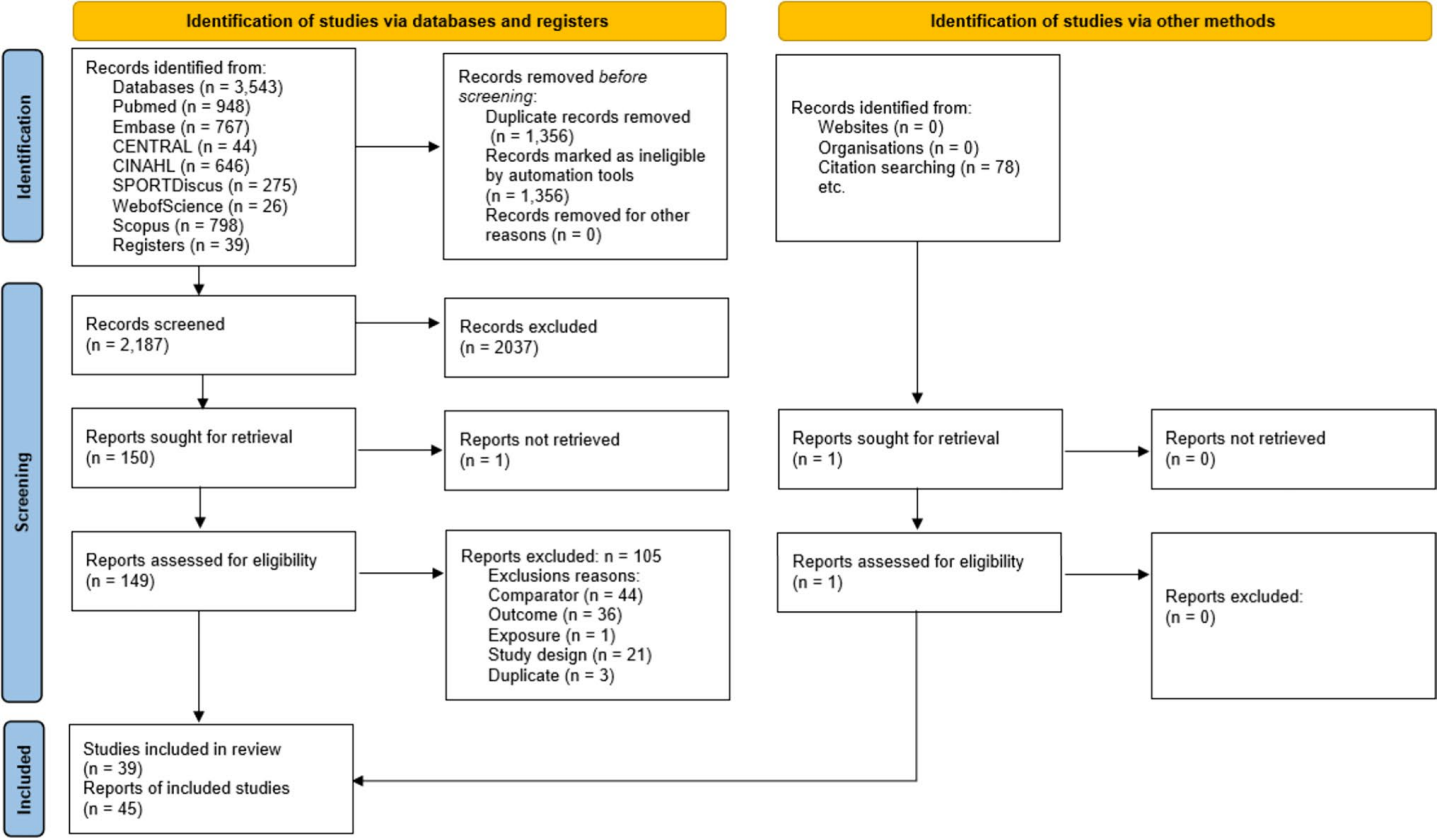
Preferred reporting items for systematic reviews and meta-analyses (PRISMA) flowchart of the study selection process

Figure 1 illustrates the study search process. The search yielded 3543 records, and after the screening process, 45 full-text reports of 39 studies were deemed eligible for inclusion. Ineligible reports with reasons for exclusion are documented in Supplement G of the ESM. Ten author groups [56–65] were followed up with data requests for missing data. Four author groups replied [57, 59, 62, 63] and two provided data for three reports (Supplement D of the ESM) [62, 63, 66].

### Study Characteristics

#### Population

In total, there were 4152 participants (mean: *n* = 101, median: *n* = 46, minimum: *n* = 17, maximum: 778, female: 20%), which included 2219 participants in the exposure groups (mean: *n* = 35, median: *n* = 22, minimum: *n* = 8, maximum: *n* = 219, female: 23%), and 1942 control participants (mean: *n* = 47, median: *n* = 20, minimum: *n* = 4, maximum: *n* = 716, female 17%). For participants with physical loading exposure, the mean age ranged from 12 to 68 years (studies reporting: *n* = 38, 97%), and the percentage with LBP was 37% (studies reporting: *n* = 31, 79%). For the control groups, mean age ranged from 11 to 56 years (studies reporting: *n* = 35, 90%) and the percentage with LBP was 14% (studies reporting: *n* = 30, 79%).

#### Exposure

The physical loading exposure population groups had varied inclusion criteria, including national ranking [16, 60, 67, 68], elite [41, 58, 65, 69–79], greater than 12 weeks [80], 12 months [81], 2 years [82], 5 years [56, 59, 62, 83, 84], or 8 years [64, 85], regular power or endurance sports over the lifetime [61], running greater than 50 km per week [86] and/or 20–40 km per week [63, 87], running greater than 100 km per month [88], cycling greater than 150 km per week for greater than 5 years, [63, 66] yoga instructors [89] and individuals engaging in physical activity in the past 14 days [90], 20.1–28.9 metabolic equivalent hours per week [91], greater than 2 h per week [92], and vigorous physical activity [93, 94]. Physical loading exposures varied, including rowing [58, 65, 81], yoga [80, 89], skiing [41, 61, 69, 75, 76, 85], weightlifting [16, 56, 61, 67, 82], wrestling [16, 67, 68], orienteering (i.e. running to find locations on a map) [67], ice hockey [67], baseball [83, 84], basketball [83, 84], Chinese martial arts [78], kendo (i.e. Japanese martial art) [83, 84], running [61, 63, 83, 84, 86–88], soccer [68, 83, 84], swimming [70, 71, 83, 84], cycling [63, 66], horseback riding [72], dance [64], gymnastics [60, 68, 73, 95], cricket [74, 96], tennis [68], climbing [79], cross-fit [77], any regular sport [59, 62], high physical activity volume [90–92, 94], moderate physical activity volume [90–93] and vigorous physical activity intensity [93, 94].

#### Comparator

The comparator groups consisted of non-athletes not participating in the same sport as the exposure [41, 56, 64, 72, 74, 75, 79, 80, 88, 89] or not participating in any regular sport [58–60, 62, 65–69, 71, 73, 76, 77, 83, 84, 86, 87], lower volume of the same exercise exposure [70, 95], sedentary or low physical activity [63, 78, 81, 82, 90–92, 94], baseline timepoint as the control [57, 65, 85, 96–99], twin with exercise discordance [61] and a historical control sampled at random [16].

#### Outcomes

Thirteen reports reported continuous outcomes only (RCT, *n* = 1, cohort, *n* = 2, cross-sectional, *n* = 10), 29 reports reported categorical outcomes only (RCT, *n* = 1, cohort, *n* = 7, cross-sectional, *n* = 21), and five reports reported both continuous and categorical outcomes (cohort, *n* = 1, cross-sectional, *n* = 4). The majority of IVD outcomes were assessed at the lumbar spine; however, two studies reported only a combined average of the thoracic and lumbar spine [41, 92]. There were several continuous MRI IVD measures where higher values described better health, including T2 (*n* = 5) [41, 56, 63, 65, 66, 86, 87], apparent diffusion coefficient (*n* = 2) [86, 94], T2 intensity (*n* = 2) [63, 94], glycosaminoglycan content (*n* = 1) [65], nucleus pulposus T1p (*n* = 1) [82] and Dixon water fraction % (*n* = 1) [63]. Quantitative structural IVD measures included height, width, area and volume [57, 63, 66, 83, 84, 86, 87]. Fractional anisotropy (*n* = 1) [94] was the only outcome where lower values described better health. All categorical outcomes described worse IVD health with higher values. The average Pfirrmann grade was measured on a continuous scale [57, 58, 63, 66, 77, 80, 86, 87, 89, 97]. Other categorical outcomes were measured as prevalence outcomes, including Pfirrmann grade > 2 [19, 58, 70–72, 74–76, 79, 80, 83, 89, 95–97], reduced signal intensity > 1 [67–69, 73, 75, 76, 81], and IVD height reduction > 1 (on a four-point ordinal scale from normal [zero] to severe height reduction [three]) [67, 69, 75, 76] or a > 50% height reduction [16, 68, 73].

#### Study Design

There were two RCTs, nine cohort studies and 28 cross-sectional studies. For ease of reporting, the RCT studies were combined with cohort analyses. Five studies included multiple publications [41, 63, 66, 68, 73, 75, 76, 83, 84, 87, 99], and therefore multiple reports were combined for all analyses, leaving a total of 28 cross-sectional studies and 11 RCT/cohort studies available for meta-analysis.

### Risk of Bias of Included Studies

The risk of bias of included cross-sectional reports (*n* = 32) is reported in Table 2. Collated, 16 reports had a low risk of bias (50%), 12 reports had some concerns (38%) and four reports had a high risk of bias (13%). Collectively, 69% clearly defined the inclusion criteria (criteria #1), 50% described the study subjects and setting in detail (criteria #2), 84% measured the physical loading exposure in a valid and reliable way (criteria #3), 72% used continuous standard criteria for measuring the physical loading exposure (criteria #4), 63% identified confounding factors (criteria #5), 41% used strategies to deal with confounding factors (criteria #6), 56% measured IVD outcomes in a valid and reliable way (criteria #7) and 100% employed an appropriate statistical analysis (criteria #8). The risk of bias of included cohort reports (*n* = 11) is reported in Table 2. Six reports had a low risk of bias (45%), five reports had some concerns (45%) and one report had a high risk of bias (9%). Collectively, 55% recruited all participants from the same population (criteria #1), 73% measured the exposures in the same way for both groups (criteria #2), 73% measured the physical loading exposure in a valid and reliable way (criteria #3), 64% identified confounding factors (criteria #4), 64% reported strategies to deal with confounding factors (criteria #5), 18% used participants free from the outcome at the start of the study (criteria #6), and 55% measured IVD outcomes in a valid and reliable way (criteria #7),100% used sufficient follow-up time for the outcome to occur (criteria #8), 73% had completed follow-up or described reasons for loss to follow-up (criteria #9), 45% reported strategies to address an incomplete follow-up (criteria #10) and 100% employed an appropriate statistical analysis (criteria #11). The risk of bias of primary outcomes of included RCTs (*n* = 2) is reported in Table 3 for the average Pfirrmann grade [80] and IVD T2 [19]. Collated, one report had some concerns for domain one [80] and all other domains for both reports were rated as low concerns. Hence, the overall risk of bias was low for one study [19], and the other had some concerns [80].

**Table 2:** Risk of bias of included reports using the Johanna Briggs Institute critical appraisal checklist for analytical cross-sectional and cohort studies

Study	Criteria								
	#1	#2	#3	#4	#5	#6	#7	#8	Overall
*Cross-sectional studies*									
Belavý et al. [87] 2017	Yes	Unclear	Yes	Yes	Yes	Yes	Unclear	Yes	Low
Belavý et al. [66] 2019	Yes	Unclear	Yes	Yes	Yes	Yes	Unclear	Yes	Low
Belavý et al. [63] 2020	Yes	Unclear	Yes	No	Yes	Unclear	Unclear	Yes	Some concerns
Benedikter et al. [58] 2022	Yes	No	Yes	Yes	No	No	Yes	Yes	Some concerns
Bowden et al. [94] 2018	No	Unclear	Yes	Yes	Yes	Yes	Unclear	Yes	Some concerns
Capel et al. [64] 2009	Yes	No	Yes	Yes	No	No	Yes	Yes	Some concerns
Cheng et al. [89] 2008	Unclear	Yes	No	No	No	No	Unclear	Yes	High
Folkvardsen et al. [71] 2016	Unclear	No	Yes	Yes	Yes	Yes	Yes	Yes	Low
Granhed et al. [16] 1988	Unclear	Unclear	Yes	Unclear	No	No	No	Yes	High
Hangai et al. [59] 2008	No	No	No	Unclear	Yes	Yes	Yes	Yes	Some concerns
Hangai et al. [83] 2009	Yes	Yes	No	Yes	Yes	Yes	Yes	Yes	Low
Hellström et al. [68] 1990	Unclear	Unclear	Yes	No	No	No	Unclear	Yes	High
Identeg et al. [79] 2023	Yes	Yes	Yes	Yes	Yes	No	Yes	Yes	Low
Jentzsch et al. [56] 2020	Yes	Yes	Yes	Yes	Yes	Yes	Unclear	Yes	Low
Kaneoka et al. [70] 2007	No	Yes	Yes	No	No	No	Yes	Yes	Some concerns
Koyama et al. [95] 2022	Unclear	Yes	Yes	Yes	Yes	No	Yes	Yes	Low
Kraft et al. [72] 2009	No	No	No	Yes	No	No	Yes	Yes	High
Lagerstrand et al. [41] 2021	Yes	Yes	Yes	Yes	Yes	Yes	Yes	Yes	Low
Li et al. [88] 2024	Yes	Yes	Yes	Yes	No	No	Yes	Yes	Low
Maurer et al. [81] 2011	Yes	Yes	Yes	Yes	Yes	No	Yes	Yes	Low
Maurer et al. [92] 2020	Yes	Yes	Yes	Yes	Yes	Yes	Yes	Yes	Low
Mitchell et al. [86] 2020	Yes	Yes	Yes	Yes	Yes	No	Unclear	Yes	Low
Owen et al. [84] 2021	Yes	Unclear	Yes	Yes	Yes	Yes	Unclear	Yes	Low
Ranson et al. [74] 2005	Yes	Unclear	Yes	Yes	No	No	Yes	Yes	Some concerns
Swärd et al. [73] 1991	Yes	Yes	Yes	No	No	No	No	Yes	Some concerns
Teichtahl et al. [90] 2015	Yes	Yes	No	Yes	Yes	Yes	Yes	Yes	Low
Tertti et al. [60] 1990	Unclear	Unclear	Yes	Yes	Yes	No	Unclear	Yes	Some concerns
Thoreson et al. [69] 2017	Yes	No	Yes	Yes	Yes	No	No	Yes	Some concerns
Vadalà et al. [82] 2014	Yes	Yes	Yes	Yes	Yes	Yes	Yes	Yes	Low
Wegner et al. [77] 2023	Yes	Yes	Yes	No	No	No	Unclear	Yes	Some concerns
Witwit et al. [75] 2018	Yes	Unclear	Yes	No	No	Unclear	Yes	Yes	Some concerns
Zhang et al. [78] 2023	Yes	Yes	Yes	Yes	Yes	Yes	Yes	Yes	Low

Study	Criteria											
	#1	#2	#3	#4	#5	#6	#7	#8	#9	#10	#11	Overall
*Cohort studies*												
Baranto et al. [98] 2006	No	No	Yes	Yes	Yes	No	No	Yes	Yes	No	Yes	Some concerns
Baranto et al. [67] 2009	Yes	Yes	Yes	No	No	No	Unclear	Yes	No	No	Yes	High
Burnett et al. [96] 1996	Unclear	Yes	Yes	No	No	No	No	Yes	Yes	Yes	Yes	Some concerns
Elfering et al. [62] 2002	Yes	Yes	no	Yes	Yes	Yes	Yes	Yes	No	No	Yes	Low
Feuerriegel et al. [85] 2025	Yes	Yes	Yes	No	No	No	Unclear	Yes	Yes	No	Yes	Some concerns
Frenken et al. [65] 2022	Yes	Unclear	Unclear	Yes	Yes	No	Yes	Yes	Unclear	Unclear	Yes	Some concerns
Horga et al. [57] 2022	Yes	Yes	Yes	Yes	Yes	No	Yes	Yes	Yes	Yes	Yes	Low
Rosenqvist et al. [99] 2023	Yes	No	Yes	Yes	Yes	No	Unclear	Yes	Yes	No	Yes	Some concerns
Shimozaki et al. [97] 2018	No	Yes	Yes	No	No	Yes	Yes	Yes	Yes	Yes	Yes	Low
Videman et al. [61] 1997	No	Yes	Yes	Yes	Yes	Unclear	Yes	Yes	Yes	Yes	Yes	Low
Witwit et al. [76] 2022	No	Yes	No	Yes	Yes	No	Yes	Yes	Yes	Yes	Yes	Low

*Cross-sectional studies criteria*: #1: Were the criteria for inclusion in the sample clearly defined?, #2: Were the study participants and the setting described in detail?, #3: Was the exposure measured in a valid and reliable way?, #4: Were continuous, standard criteria used for measurement of the condition?, #5: Were confounding factors identified?, #6: Were strategies to deal with confounding factors stated?, #7: Were the outcomes measured in a valid and reliable way?, #8: Was appropriate statistical analysis used?

*Cohort studies criteria*: #1: Were the two groups similar and recruited from the same population?, #2: Were the exposures measured similarly to assign people to both exposed and unexposed groups?, #3: Was the exposure measured in a valid and reliable way?, #4: Were confounding factors identified?, #5: Were strategies to deal with confounding factors stated?, #6: Were the groups/participants free of the outcome at the start of the study (or at the moment of exposure)?, #7: Were the outcomes measured in a valid and reliable way?, #8: Was the follow-up time reported and sufficient to be long enough for outcomes to occur?, #9: Was follow-up complete, and if not, were the reasons to loss to follow-up described and explored?, #10: Were strategies to address incomplete follow-up utilised?, #11: Was appropriate statistical analysis used?

**Table 3:** ROB of included randomised controlled reports using Cochrane Risk of Bias 2 tool

Study	#1.1	#1.2	#1.3	#2.1	#2.2	#2.3	#2.4	#2.5	#2.6	#3.1	#3.2	#3.3	#4.1	#4.2	#4.3	#5.1	#5.2	#5.3
Owen et al. [19] 2020	Yes	Probably Yes	No	Yes	Yes	Probably No			Yes	No	Yes	Probably No	No	No	No	Yes	No	No
Telles et al. [80] 2016	Yes	NI	No	Yes	Yes	Probably No	No	Yes	Yes	No	Probably Yes		No	No	No	Yes	No	Probably No

Study	Domain 1	Domain 2	Domain 3	Domain 4	Domain 5	Overall ROB
Score for each domain and overall risk of bias						
Owen et al. 2020	Low	Low	Low	Low	Low	Low
Telles et al. 2016	Some concerns	Low	Low	Low	Low	Some concerns

#1.1 Was the allocation sequence random? #1.2 Was the allocation sequence concealed until participants were enrolled and assigned to interventions? #1.3 Did baseline differences between intervention groups suggest a problem with the randomisation process? #2.1. Were participants aware of their assigned intervention during the trial? #2.2. Were carers and people delivering the interventions aware of participants’ assigned intervention during the trial? #2.3. If Y/PY/NI to 2.1 or 2.2: Were there deviations from the intended intervention that arose because of the trial context? #2.4 If Y/PY to 2.3: Were these deviations likely to have affected the outcome? #2.5. If Y/PY/NI to 2.4: Were these deviations from intended intervention balanced between groups?,#2.6 Was an appropriate analysis used to estimate the effect of assignment to intervention?, #2.7 If N/PN/NI to 2.6: Was there potential for a substantial impact (on the result) of the failure to analyse participants in the group to which they were randomised?, #3.1 Were data for this outcome available for all, or nearly all, participants randomised?, #3.2 If N/PN/NI to 3.1: Is there evidence that the result was not biased by missing outcome data?,3.3 If N/PN to 3.2: Could missingness in the outcome depend on its true value?, #4.1 Was the method of measuring the outcome inappropriate?, #4.2 Could measurement or ascertainment of the outcome have differed between intervention groups?, #4.3 If N/PN/NI to 4.1 and 4.2: Were outcome assessors aware of the intervention received by study participants?, #5.1 Were the data that produced this result analysed in accordance with a pre-specified analysis plan that was finalised before unblinded outcome data were available for analysis?, #5.2. Is the numerical result being assessed likely to have been selected, on the basis of the results, from multiple eligible outcome measurements (e.g. scales, definitions, timepoints) within the outcome domain? #5.3 Is the numerical result being assessed likely to have been selected, on the basis of the results, from multiple eligible analyses of the data?

*ROB* risk of bias

### Results of Individual Studies

Characteristics of the 45 reports are described in Table 4. All outcomes for studies included in the meta-analysis, including all data handling steps, are reported in Supplement C of the ESM. There were three cross-sectional studies and one cohort study with heterogeneous outcomes because of a variation in the definitions of degeneration, which were not included in the meta-analysis, and will be reported here [59–61, 64]. Results from the three cross-sectional studies reported: (1) sport activities were associated with degeneration at L5/S1 only (OR [95% CI] 3.36 [1.31, 9.90], *n* = 270) [59]; (2) IVD degeneration was uncommon in young competitive gymnasts with signal intensity reductions > 50% present in three of 35 gymnasts and one of ten controls [60]; and (3) 33% of a group of dancers (total *n* = 40) had lumbar IVD degeneration compared with 45% of the control group (total *n* = 20) [64]. The cohort study observed monozygotic twins over 7 years and reported that power sports participation was associated with IVD degeneration, via reduced signal intensity compared to twin controls without the same sports participation; however, there was no evidence to suggest exposure to endurance sports was associated with IVD degeneration compared to the twin controls [61].

**Table 4:** Participant characteristics of included studies

Study	Study design	Exercise groups	Age (years)	*N* (*n* female)	*N* back pain	Control group	Age (years)	*N* (*n* female)	*N* back pain
*Cross-sectional studies*									
Belavý et al. [63, 66, 87] 2017, 2019 and 2020	Cross-sectional	Cyclists: ≥ 150 km/wk, > 5 y	29.9 (3.8)	22 (54)	0	No regular sport for 5 y: < 150 min PA/wk, < 15 min walk in commute	29.3 (3.7)	24 (9)	0
		Running: 20–40 km/wk, > 5 y	30.2 (3.2)	30 (17)	0				
		Running: > 50 km/wk, > 5 y	30.1 (3.9)	25 (14)	0				
Benedikter et al. [58] 2022	Cross-sectional	Elite rowers	23.4 (3.0)	20(9)	0	No elite sport, < 3 h/wk exercise	24.4 (3.0)	37 (18)	–
Bowden et al. [94] 2018	Cross-sectional	> 30 min mod-vig PA	46.1 (6.4)	14 (8)	0	< 30 min mod-vig PA	43 (6.5)	12 (9)	0
Capel et al. [64] 2009	Cross-sectional	Dancers for > 8 y (ballet or flamenco)	24.17 (4.0)	40 (40)	0	No dance or sport, but had variation in PA levels	22.3 (3.1)	20 (20)	0
Cheng et al. [89] 2008	Cross-sectional	Yoga instructors	45.1 (10.6)	18 (15)	0	Asymptomatic, non-yoga control	50.6 (8.5)	18 (13)	0
Folkvardsen et al. [71] 2016	Cross-sectional	Elite competitive swimmers for minimum 8 y, average training > 30 km/wk in last 2 y	18.7 (–)	100 (40)	52	< 3 h brisk activities/wk, swimming < 1/month, no current sport participation	20.8 (–)	96 (67)	64
Granhed et al. [16] 1988	Cross-sectional	Wrestlers (former top ranked)	49.3 (6.2)	32 (0)	19	Historical control group	43.5 (–)	716 (–)	–
		Weightlifters (former top ranked)	50.7 (7.2)	30 (0)	3				
Hangai et al. [59] 2008	Cross-sectional	Elderly playing sports > 3/wk for > 5 y	68.4 (6.3)	34 (156*)	179*	Elderly not engaging in sports	68.4 (6.3)	208 (156*)	179*
Hangai et al. [83] 2009 and Owen et al. [84] 2021	Cross-sectional	Baseball: > 5 y	20 (1.0)	57 (0)	3	Non-active: no sport > 3 times/wk in lifetime	19 (1.0)	71 (36)	0
		Basketball: > 5 y	0	63 (19)	1				
		Kendo: > 5 y	20 (1.0)	51 (15)	0				
		Running: > 5 y	19 (1.0)	43 (10)	5				
		Soccer: > 5 y	19 (0.0)	47 (0)	2				
		Swimming: > 5 y	19 (1.0)	47 (9)	4				
Hellström et al. [68] 1990 and Swärd et al. [73] 1991	Cross-sectional	Wrestler athletes	20.5 (2.2)	30 (0)	0	Non-athletes	20.5 (2.2)	30 (0)	0
		Soccer athletes	21 (2.4)	31 (0)	0				
		Tennis athletes	21.5 (2.9)	20 (0)	0				
		Male gymnast athletes	22 (3.0)	26 (0)	0				
		Female gymnast athletes	19.5 (2.8)	26 (26)	0				
Identeg et al. [79] 2023	Cross-sectional	High-performance climbers > 5 y (Elite in last 1 y)	23.1 (3.2)	15 (8)	11	Controls with no elite sport or climbing history	24.3 (1.5)	15 (8)	9
Jentzsch et al. [56] 2020	Cross-sectional	Weightlifters: > 4 times/wk, > 5 y	31.4 (8.7)	12(4)	0	Non-weight lifters: < 2 days/wk	27.9 (4.3)	13 (6)	0
Kaneoka et al. [70] 2007	Cross-sectional	Elite swimmers	19.6 (–)	56 (21)	43	Recreational swimmers	21.1 (–)	38 (14)	33
Kraft et al. [72] 2009	Cross-sectional	Elite national horseback riders	32.4 (9.3)	58 (40)	51	Non-riders, performed other sports	28.7 (5.6)	30 (13)	10
Lagerstrand et al. [41] 2021 and Rosenqvist et al. [99] 2023	Cross-sectional	Elite skiers	18.2 (1.1)	58 (28)	–	Active controls: < 2 h sport/wk	16.4 (0.6)	26 (17)	–
Li et al. [88] 2024	Cross-sectional	Amateur marathon runners (> 1 y, > 100 km/mo)	41.7 (7.2)	54 (0)	–	Healthy volunteers	37.7 (5.3)	30 (0)	–
Maurer et al. [81] 2011	Cross-sectional	Rowers > 12 mo, training > 5 times/wk	16 (1.6)	22 (0)	0	No regular PA	16.27 (1.3)	22 (0)	0
Maurer et al. [92] 2020	Cross-sectional	Regular PA > 2 h/wk	56.3 (9.2)	113 (161)	56	No or nearly no PA	56.3 (9.2)	97 (–)	52
		Regular PA for 1 h/wk	56.3 (9.2)	118 (–)	66				
		Irregular PA for 1 h/wk	56.3 (9.2)	57 (–)	38				
Mitchell et al. [86] 2020	Cross-sectional	Running: > 50 km/wk for > 10 y	48 (4.0)	9 (0)	–	Non-active: < 150 min mod PA/wk, < 10 min commute walking and no sport > 10 y	50 (4.0)	8 (0)	–
Ranson et al. [74] 2005	Cross-sectional	Professional fast cricket bowlers	26 (4.0)	36 (0)	0	Active controls	25 (5.0)	17 (0)	0
Teichtahl et al. [90] 2015	Cross-sectional	Active PA: active on 9–14 days/14 days	46.5 (6.5)	19 (10)	14	Inactive: 0 days PA/14 days	47.2 (5.0)	15 (13)	12
		Moderate PA: active on 1–8 days/14 days	45.1 (4.3)	38 (26)	32				
Tertti et al. [60] 1990	Cross-sectional	National/international gymnasts	12 (2.6)	35 (17)	11	No sport history	12 (2.5)	10 (–)	–
Thoreson et al. [69] 2017	Cross-sectional	Elite mogul skiers	17.6 (1.0)	16 (2)	8	No sport > 2 h/wk	16.4 (0.6)	28 (19)	12
Vadalà et al. [82] 2014	Cross-sectional	Weightlifters, > 2 y weightlifting, could lift > 1.5 times body weight	25.3 (4.4)	13 (0)	0	Sedentary lifestyle: no previous or current sport	23.5 (1.3)	13 (0)	0
Wegner et al. [77] 2023	Cross-sectional	Female CrossFit athletes	26 (2.7)	9 (9)	–	Healthy non-athletic female control	29.5 (2.9)	10 (10)	–
Witwit et al. [75, 76] 2018 and 2022	Cross-sectional	Elite skiers	18.2 (1.1)	75 (35)	18	Non-skiers	16.4 (0.6)	27 (18)	8
Zhang et al. [78] 2023	Cross-sectional	Elite martial arts athletes	20.1 (1.1)	9 (5)	–	Sedentary controls	21.4 (1.5)	18 (10)	–
		Amateur enthusiasts	21.1 (2.0)	18 (10)	–				

Study	Study design	Exercise groups	Age, y	*N* (*n* female)	*N* back pain	Control group	Age, y	*N* (*n* female)	*N* back pain
*RCT/cohort studies with a separate control group*									
Baranto et al. [67] 2009	Cohort	Weightlifters since age 10 y at national/international level	30 (5.8)	21 (0)	12	Non-athletes with no current or previous sport	28 (4.2)	21 (0)	9
		Wrestlers since age 10 y at national/international level	24 (5.7)	13 (0)	6				
		Orienteers since age 10 y at national/international level	25 (4.1)	18 (0)	7				
		Ice-hockey players since age 10 y at national/international level	24 (3.3)	19 (0)	14				
Elfering et al. [62] 2002	Cohort	Occasional sport < 1/wk	35.8 (7.8)	9 (11*)	18*	No sport	35.8 (7.8)	10 (11*)	18*
		Regular sport > 1/wk	35.8 (7.8)	15 (–)	–				
		Regular competitive sports	35.8 (7.8)	7 (–)	–				
Horga et al. [57] 2022	Cohort	Post-16 wk marathon training	34 (10.6)	21 (–)	0	Runners dropped out of training	34 (10.6)	4 (–)	0
Koyama et al. [95] 2022	Cohort	International gymnasts	19.6 (1.4)	7 (–)	13	Regional gymnasts	19.4 (0.8)	7 (–)	11
		National gymnasts	19.3 (0.6)	37 (–)	39				
Lagerstrand et al. [41] 2021 and Rosenqvist et al. [99] 2023	Cohort	High skiing training dose > 5 h/wk	17.2 (0.4)	24 (5)	0	Low-normal training dose, < 5 h/wk	16.6 (0.5)	11 (7)	0
Owen et al. [19] 2020	RCT	Chronic low back pain, 6-mo strength and conditioning training	35 (5)	20 (10)	20	Chronic low back pain, 6-mo motor control and manual therapy	35 (4)	20 (9)	20
Telles et al. [80] 2016	RCT	Individuals with disc degeneration, completing yoga training (6 mo)	36.1 (7.33)	20 (11)	20	Individuals with disc degeneration, waitlist control (6 mo)	37.4 (4.9)	20 (12)	20
Videman et al. [61] 1997	Cohort	Endurance sports (running or cross-country skiing)	49.6 (7.1)	22 (0)	–	Twin with endurance exercise discordance	49.6 (7.1)	22 (0)	–
		Power sports (weightlifting)	46.5 (8.3)	12 (0)	–	Twin with power sport exercise discordance	46.5 (8.3)	12 (0)	–
Witwit et al. [75, 76] 2018 and 2022	Cohort	Elite skiers, 2-y follow-up	20 (0.6)	30 (13)	14	Non-athletes	19 (0.5)	16 (12)	6

Study	Study design	Exercise groups	Ag (y)	*N* (*n* female)	*N* back pain	Control group	Age (y)	*N* (*n* female)	*N* back pain
*RCT/cohort studies with baseline as a control*									
Baranto et al. [98] 2006	Cohort	Elite divers ≥ 10 y at highest possible ranking, 6 y post	21.2 (3.0)	17 (–)	16	Elite divers ≥ 10 y at highest possible ranking, baseline	16.4 (3.1)	18 (14)	0
Benedikter et al. [58] 2022	Cross-sectional	Elite rowers, end of season	–	20 (9)	0	Elite rowers, start of season	23.4 (3.0)	20 (9)	0
Burnett et al. [96] 1996	Cohort	Fast bowlers, post 2 y	16.3 (0.6)	19 (0)	6	Fast bowlers, baseline	13.6 (0.6)	19 (0)	1
Feuerriegel et al. [85] 2025	Cohort	Youth competitive skiers, post 2 y	19.6 (1.2)	63 (25)	27	Youth competitive skiers, baseline	15.3 (1.3)	63 (25)	25
Frenken et al. [65] 2022	Cohort	Elite rowers, preseason (high training load)	–	17 (9)	0	Elite rowers post season recovery, low training load	23.9 (3.3)	17 (9)	0
Horga et al. [57] 2022	Cohort	Post-16 wk marathon training	–	21 (9)	0	Runners with no marathon history, 3–4 h running/wk, baseline	30 (10.0)	28 (14)	0
Koyama et al. [95] 2022	Cohort	2-y follow-up, all gymnasts	–	51 (–)	–	All gymnasts, baseline	19.35 (0.8)	51 (–)	–
Owen et al. [19] 2020	RCT	Strength and conditioning training, 6-month follow-up	–	17 (–)	0	Individuals with chronic low back pain, baseline	35 (5.0)	20 (10)	20
Lagerstrand et al. [41] 2021 and Rosenqvist et al. [99] 2023	Cohort	High skiing training dose > 5 h/wk, 2-y follow-up	–	24 (5)	0	High skiing training dose > 5 h/wk, baseline	17.2 (0.4)	24 (5)	0
Lagerstrand et al. [41] 2021 and Rosenqvist et al. [99] 2023	Cohort	Low-normal training dose, < 5 h/wk, 2-y follow-up	–	11 (7)	0	Low-normal training dose, < 5 h/wk, baseline	16.6 (0.5)	11 (7)	0
Shimozaki et al. [97] 2018	Cohort	Weightlifters, 3-y follow-up	–	12 (6)	3	Weightlifters for > 2 y, baseline	11.4 (2.0)	12 (6)	0
Telles et al. [80] 2016	RCT	Daily yoga, 3-mo follow-up	–	14 (–)	20	Yoga group, baseline	36.1 (7.3)	20 (11)	20
Witwit et al. [75, 76] 2018 and 2022	Cohort	Elite skiers, 2-y follow-up	–	30 (13)	14	Elite skier, baseline	20 (0.6)	30 (13)	14

Data are counts or mean (standard deviation)

*h* hours, *min* minutes, *mo* months, *mod* moderate, *NR* not reported, *PA* physical activity, *RCT* randomised controlled trial, *vig* vigorous, *wk* week, *y* years

*n across the full sample including intervention, control and all exposure groups (where relevant)

### Results of Syntheses

#### Primary Synthesis: Continuous Measures of IVD Health

##### Combined Physical Loading

**Fig. 2:**
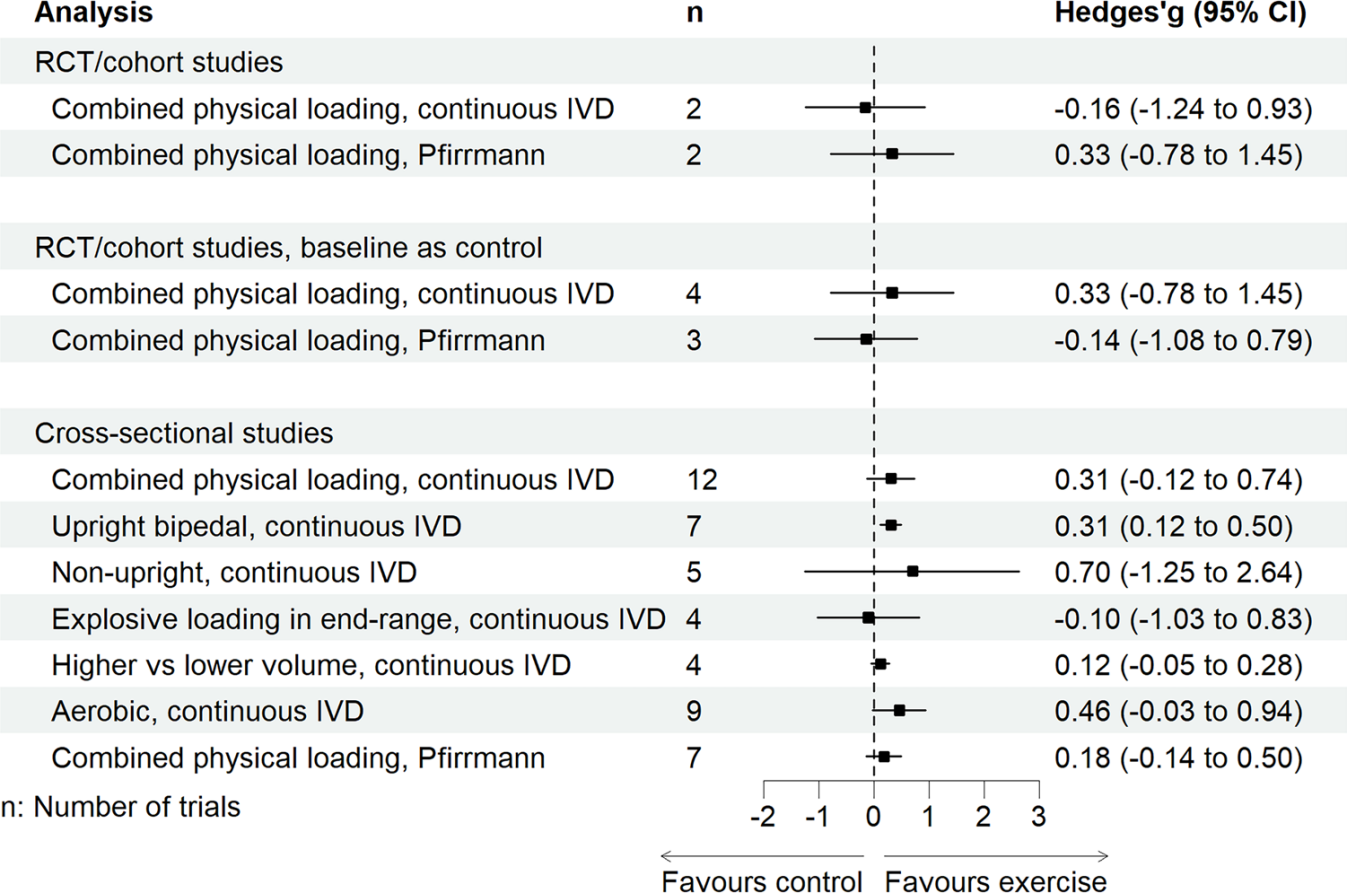
Overall effect sizes of a pairwise, random-effects, restricted maximum likelihood meta-analysis of continuous intervertebral disc (IVD) outcomes and categorical Pfirrmann grade average compared to a control. *CI* confidence interval, *RCT* randomised controlled trial

All continuous IVD outcome effect sizes are presented in Table 5, with overall forest plots in Fig. 2 and individual forest plots in Supplement H of the ESM. Primary analysis of cross-sectional studies revealed a small effect of better IVD health compared with the control with combined physical loading exposure, albeit with wide CIs (studies, *n* = 12, combination of all listed physical loading exposures). However, a sensitivity analysis via a leave-one-out meta-analysis showed that omitting a weightlifting study (Vadalà et al.) [82] revealed a significant moderate effect favouring combined physical loading (Hedges’ *g* [95% CI]: 0.41 [0.01, 0.81], *P* = 0.044). Meta-regression revealed neither age (*β* [95% CI] 0.01 [− 0.03, 0.05], *P* = 0.651) nor sex (*β* [95% CI] 0.01 [− 0.01, 0.02], *P* = 0.541) moderated these observations and neither explained any statistical heterogeneity (*R*^2^: 0%). No investigation was made into LBP moderation because of the overall paucity in reporting. In RCTs/cohort studies, participants engaging in combined physical loading revealed a small effect of better IVD health, with a wide CI compared with the baseline control group (studies, *n* = 4) and a negligible effect compared to a separate control group (studies, *n* = 2).

**Table 5:** Summary of findings from a pairwise, random-effects, restricted maximum likelihood meta-analysis of physical loading exposures compared with a comparator

Intervention type	Studies	*N*	Hedges’ *g* (95% CI)	*P*-value	Effect size	*I*^2^	Prediction interval	Egger’s P	High ROB	GRADE
**Continuous IVD outcomes**										
** Cross-sectional studies**										
Combined physical loading	12	974	0.31 (− 0.12, 0.74)	0.158	Small	98%	− 1.38, 1.99	0.241	0%	Very low^a,b^
Upright bipedal	7	586	0.31 (0.12, 0.50)	0.002	Small	86%	− 0.30, 0.91	–	0%	Very low^a^
Non-upright, non-contact	5	260	0.70 (− 1.25, 2.64)	0.336	Medium	99%	− 5.02, 6.41	–	0%	Very low^c^
Explosive loading in end range	4	245	− 0.10 (− 1.03, 0.83)	0.756	Negligible	89%	− 7.86, 7.30	–	0%	Very low^c^
Aerobic exercise	9	802	0.46 (− 0.03, 0.94)	0.065	Small	98%	− 1.33, 2.43	–	0%	Very low^a,b^
Higher vs lower physical loading	4	174	0.12 (− 0.05, 0.28)	0.104	Negligible	0%	− 0.10, 0.34	–	0%	Low
** RCT and cohort studies (control group as a comparator at the final timepoint)**										
Combined physical loading	2	65	− 0.16 (− 1.24, 0.93)	0.418	Negligible	0%	–	–	0%	Very low^c^
**RCT and cohort studies (baseline timepoint as a comparator)**										
Combined physical loading	4	90	0.33 (− 0.78, 1.45)	0.454	Small	94%	− 2.54, 3.204	–	0%	Very low^c^
**Average Pfirrmann grade of IVD degeneration**										
** Cross-sectional studies**										
Combined physical loading	7	675	0.18 (− 0.14, 0.50)	0.276	Negligible	85%	− 0.92,1.27	–	17%	Very low^a,b^
** RCT and cohort studies (baseline timepoint as a comparator)**										
Combined physical loading	3	53	− 0.14 (− 1.08, 0.79)	0.576	Negligible	64%	− 4.84, 4.55	–	0%	Very low^c^

Study	Intervention type	*N*	IVD outcome	Hedges’ *g* (95% CI)	Effect size	High ROB
**Continuous IVD outcomes compared to categorical IVD outcomes of combined physical loading exposures**						
Belavý et al. [63, 66, 87] 2017, 2019 and 2020	Cross-sectional	101	Continuous	0.27 [0.15, 0.39]	Small	0%
			Categorical	0.37 [− 0.00, 0.74]	Small	0%
Horga et al. [57] 2020	Cohort (separate control)	25	Continuous	− 0.04 [− 0.39, 0.31]	Negligible	0%
			Categorical	0.00 [− 0.71, 0.71]	Negligible	0%
Mitchell et al. [86] 2020	Cross-sectional	17	Continuous	0.88 [0.53, 1.23]	Large	0%
			Categorical	0.77 [0.24, 1.30]	Large	0%

Hartung–Knapp–Sidik–Jonkman adjustment applied for fewer than five studies. GRADE ratings started at low, given studies were observational *CI* confidence interval, *GRADE* Grading of Recommendations Assessment, Development, and Evaluation criteria, *IVD* intervertebral disc, *RCT* randomised controlled trial, *ROB* risk of bias

^a^Downgraded by one level because of inconsistency

^b^Downgraded by one level because of imprecision

^c^Downgraded by two levels because of imprecision

##### Physical Loading Subgroups

Participants engaging in upright bipedal physical loading (studies, *n* = 7), including running, skiing, physical activity (80% running based), brisk walking/running and running-based sports (running, basketball, soccer) revealed a small effect for better IVD health compared with the control. Participants engaging in any aerobic physical loading (studies, *n* = 9), including running, skiing, physical activity (80% running based), brisk walking/running, running-based sports (running, basketball, soccer), rowing and swimming revealed a small effect size favouring physical loading, albeit with a wide CI. However, independently omitting the two rowing studies improved overall confidence, favouring aerobic physical loading, revealing a small effect when omitting Benedikter et al. [58] (Hedges’ *g* [95% CI] 0.54 [0.02, 1.06], *P* = 0.040) or a negligible effect when omitting Frenken et al. [65] (Hedges’ *g* [95% CI] 0.23 [0.02, 0.45], *P* = 0.033). Participants engaging in non-upright non-contact physical loading (studies, *n* = 5), including rowing, swimming and cycling revealed a medium effect size, albeit with a wide CI. Participants engaging in explosive loading in the end range (studies, *n* = 4) including weightlifting, kendo, martial arts, and baseball or participants exposed to higher versus lower loading of the same type of physical load (studies *n* = 4) including skiing, physical activity (80% running based), running and martial arts revealed a negligible difference in IVD health compared to control participants, with wide CIs.

#### Secondary Syntheses: Categorical Measures of IVD Health

**Fig. 3:**
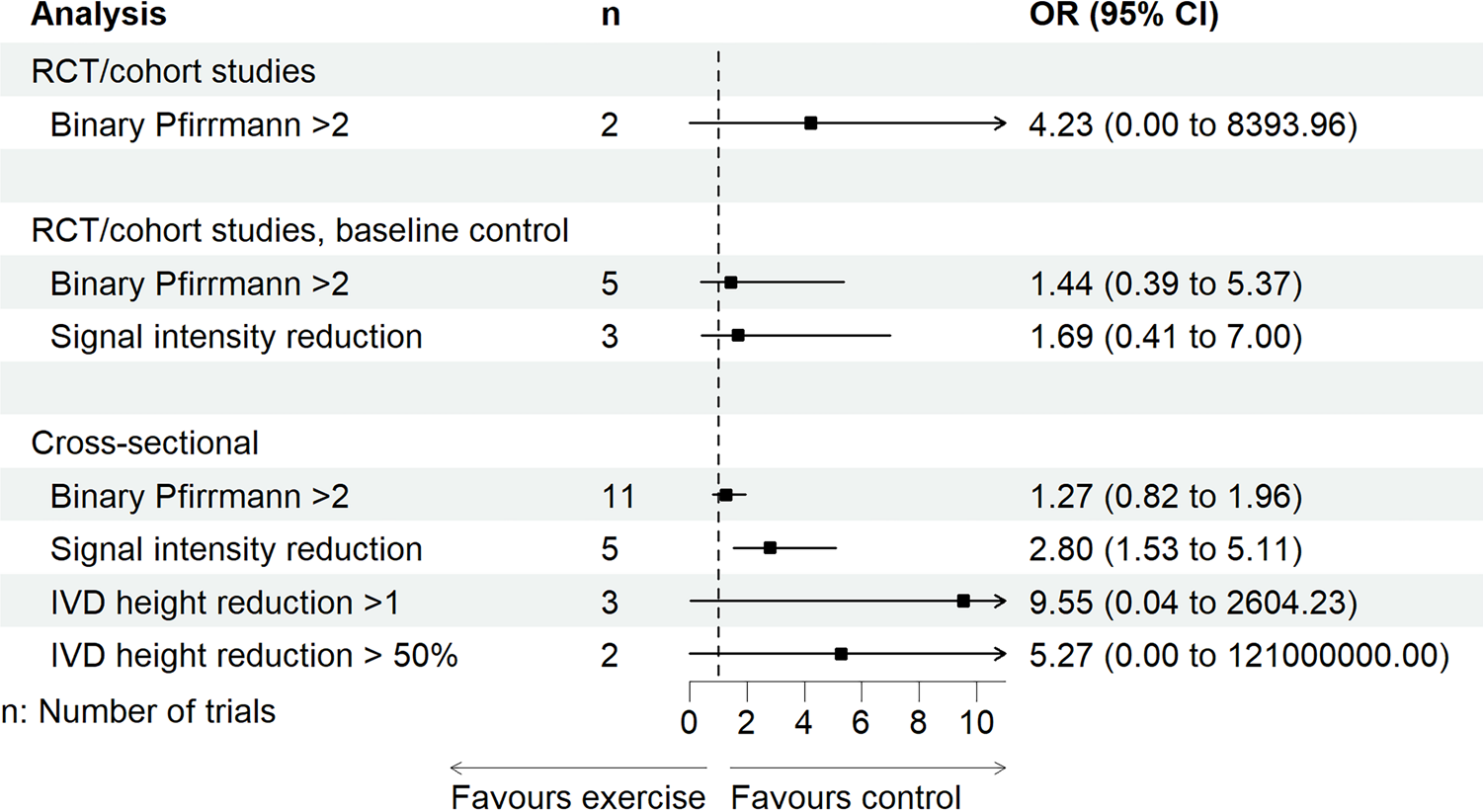
Overall effect sizes of a pairwise, random-effects meta-analysis of odds ratios with a Paule-Mandel estimator of categorical outcomes of binary intervertebral disc (IVD) degeneration from combined physical loading compared to a control. *CI* confidence interval, *OR* odds ratio, *RCT* randomised controlled trial

**Fig. 4:**
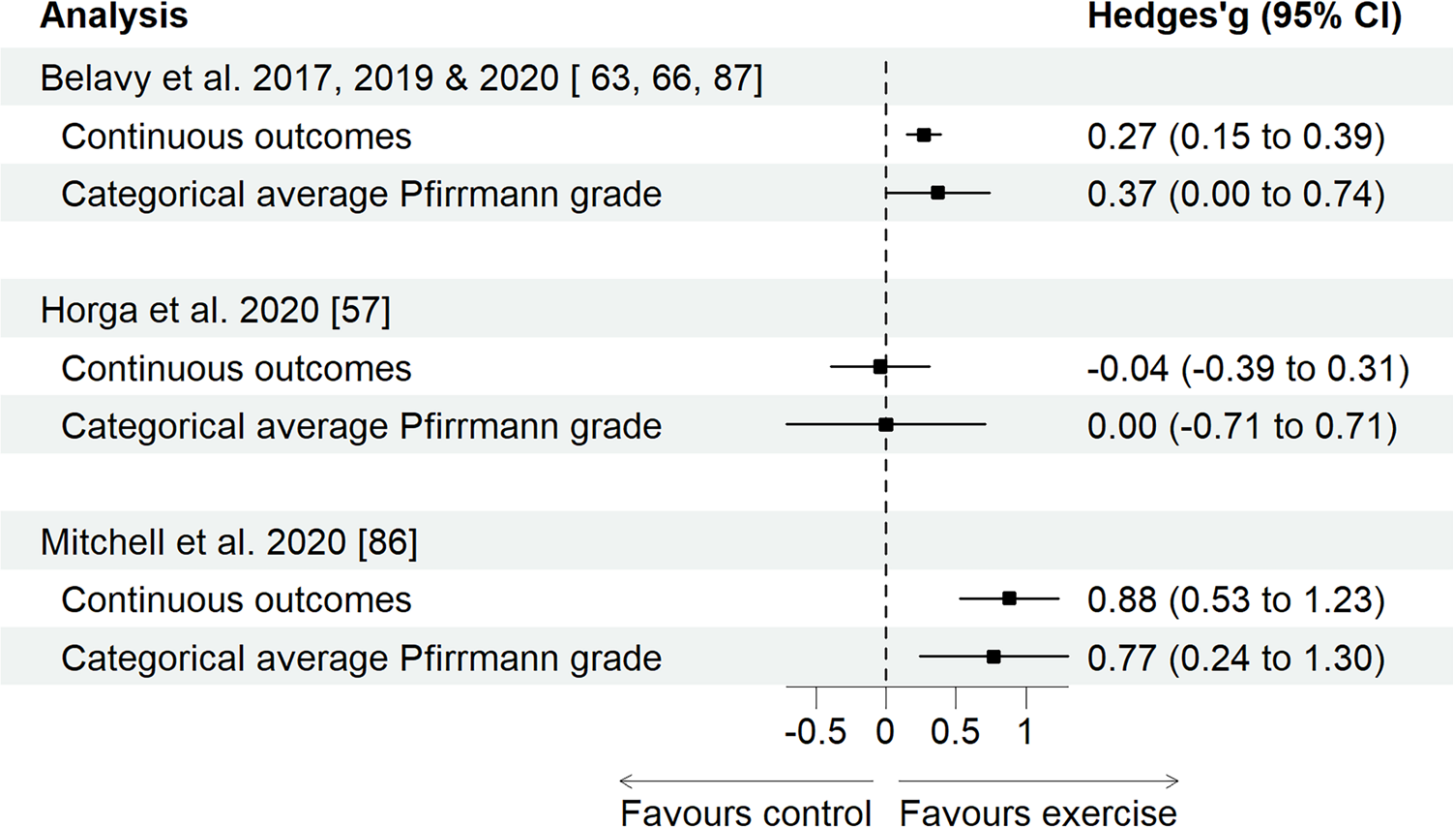
Continuous intervertebral disc (IVD) outcomes compared to categorical IVD outcomes. A pairwise random-effects restricted maximum likelihood meta-analysis of continuous IVD outcome effect size with categorical IVD outcome effect size from cross-sectional and randomised controlled trials/cohort studies for combined physical loading. *CI* confidence interval

All categorical outcomes were synthesised by combining physical loading only, without subgroups of physical loading, owing to the overall paucity (Supplement H of the ESM). Combined physical loading revealed a negligible effect for an average Pfirrmann grade in RCTs/cohort studies with baseline as the comparator (studies, *n* = 3) and cross-sectional studies (studies, *n* = 7), both with wide CIs (Table 5 and Fig. 2). However, a leave-one-out meta-analysis revealed a significant small effect favouring physical loading (reduced Pfirrmann grade) when omitting a rowing study from the cross-sectional study analysis (Benedikter et al. [58]; Hedges’ g [95% CI]: 0.31 [0.13, 0.48], *P* = 0.001). Prevalence of degeneration according to Pfirrmann grade > 2 in cross-sectional studies (*n* = 11) revealed a negligible effect compared to the control, with wide CIs (Table 6 and Fig. 3). These effects were not moderated by age (*β* [95% CI] − 0.01 [− 0.031,0.01], *P* = 0.322, *R*^2^: 3%) or LBP (*β* [95% CI] 0.01 [− 0.00, 0.02], *P* = 0.057, *R*^2^: 55%), however, they were moderated by sex (female *β* [95% CI] − 0.04 [− 0.09, − 0.01], *P* = 0.010), which explained 62% of the statistical heterogeneity. Prevalence of Pfirrmann grade > 2 in RCTs/cohort studies with baseline as the control (studies, *n* = 5) revealed a negligible effect; however, RCTs/cohort studies with a separate control (studies, *n* = 2) revealed a large effect size favouring the control, albeit both with wide CIs. IVD degeneration via reduced signal intensity (> 1 on a three-point scale) revealed a medium effect size favouring control in cross-sectional studies (studies, *n* = 5, Fig. 3). However, any reduced signal intensity in RCTs/cohort studies with baseline as a control (studies, *n* = 3) revealed a small effect favouring the control (Table 6, Fig. 3), however, with wide CIs. IVD height reduction > 1 (on a four-point scale, studies, *n* = 3) and IVD height reduction > 50% (studies *n* = 2) revealed large effect sizes favouring the control in cross-sectional studies; however, with very wide CIs. The final synthesis comparing the sensitivity of IVD measures revealed that all continuous measures of IVD health have smaller variance compared with all categorical measures (studies, *n* = 3, Table 5 and Fig. 4).

**Table 6:** Summary of findings from a pairwise random-effects meta-analysis of odds ratios with a Paule–Mandel estimator meta-analysis of categorical outcomes of IVD degeneration of physical loading exposures compared to a comparator

Intervention type	Studies	*N*	Odds ratio (95% CI)	*P* value	Effect size	*I*^2^	Prediction interval	Egger’s *P*	High ROB	GRADE
**Pfirrmann grading of > 2 IVD, cross-sectional studies**										
Combined physical loading	11	13,379	1.27 (0.82, 1.96)	0.283	Negligible	80%	0.28, 5.68	0.42	20%	Very low^a,b^
**Pfirrmann grading of > 2 IVD, RCTs and cohort studies (baseline timepoint as a comparator)**										
Combined physical loading	5	506	1.44 (0.39, 5.37)	0.586	Negligible	79%	0.02, 126.87	–	0%	Very low^c^
**Pfirrmann grading of > 2 IVD, RCTs and cohort studies (control group as a comparator at the final timepoint)**										
Combined physical loading	2	70	4.23 (0.00, 8393.96)	0.250	Large	0%	–	–	0%	Very low^c^
**IVD signal intensity reduction > 1, cross-sectional studies**										
Combined physical loading	5	276	2.80 (1.53, 5.11)	0.001	Medium	0%	1.05, 7.45	–	40%	Low
**IVD signal intensity reduction, RCTs and cohort studies (baseline timepoint as a comparator)**										
Combined physical loading	3	111	1.69 (0.41, 7.00)	0.255	Small	6%	0.02, 169.05	–	0%	Very low^b^
**IVD height reduction > 1, cross-sectional studies**										
Combined physical loading	3	203	9.55 (0.04, 2604.23)	0.226	Large	63%	0.00, 160,000,000,000,000	–	33%	Very low^c^
**IVD height reduction > 50%, cross-sectional studies**										
Combined physical loading	2	924	5.27 (0.00, 121,000,000)	0.430	Large	89%	–	–	100%	Very low^c,d^

Odds ratio > 1 in favour of less degeneration, odds ratio < 1 in favour of more degeneration. Hartung–Knapp–Sidik–Jonkman adjustment applied for fewer than five studies. GRADE ratings started at low, given studies were observational

*CI* confidence interval, *GRADE* Grading of Recommendations Assessment, Development, and Evaluation criteria, *IVD* intervertebral disc, *RCT* randomised controlled trial, *ROB* risk of bias

^a^Downgraded by one level because of inconsistency

^b^Downgraded by one level because of imprecision

^c^Downgraded by two levels because of imprecision

^d^Downgraded by two levels because of a high risk of bias

#### Statistical Heterogeneity

Investigation into statistical heterogeneity was plausible for the combined physical loading continuous IVD outcomes and binary Pfirrmann prevalence analysis, as reported above. No other analysis was investigated for statistical heterogeneity owing to overall data paucity, with fewer than ten studies in the meta-analysis.

#### Sensitivity Analyses

Sensitivity testing (Supplement I-M of the ESM) revealed no difference in estimates for different ρ-values and robust variance estimators for all analyses. No other leave-one-out meta-analysis led to a significantly different effect estimate.

### Reporting Biases

Because of data paucity (fewer than ten studies), only the primary analysis for combined physical loading (cross-sectional studies) and secondary analysis for binary Pfirrmann grade > 2 (cross-sectional studies) were inspected via a funnel plot (Supplement N of the ESM). No asymmetry was observed, and the trim-and-fill method had no impact on results (Supplement O of the ESM), which may indicate a lack of publication and/or small-study bias [54].

### Certainty of Evidence

Overall certainty of evidence for the primary analysis of continuous IVD outcomes was rated very low across all analyses, except for ‘higher vs lower physical loading’, which was rated as low (Table 5). GRADE ratings started as low as all analyses contained observational studies. Reasons for downgrading primary analyses by (a) one level were inconsistency (3/8 analyses) and imprecision (2/8 analyses), and (b) by two levels was imprecision (4/8 analyses). Overall certainty of evidence for a secondary analysis of categorical outcomes was rated very low across all, except for a categorical signal intensity > 1, which was rated as low. Reasons for downgrading secondary analyses by (a) one level were inconsistency (2/9 analyses) and imprecision (3/9), and (b) by two levels were imprecision (5/9 analyses) and a high risk of bias 1/9 analyses.

## Discussion

This is the first systematic review to incorporate a meta-analysis examining the associations between physical loading and MRI-derived markers of IVD health. Our results revealed upright bipedal physical loading was associated with better IVD outcomes compared with the control. All other continuous IVD outcomes were not significant, with two physical loading subgroups favouring better IVD health (aerobic and non-upright, non-contact) and two subgroups revealing negligible effects compared to the control (explosive loading in the end range and ‘higher vs lower physical loading’). Overall data paucity limited confidence in the synthesis of RCTs/cohort studies. Prevalence of degeneration according to categorical outcomes revealed decreased IVD signal intensity with combined physical loading compared with the control; however, no other categorical outcome revealed significant differences.

When all physical loading exposures were considered together, there was no difference in continuous measures of IVD health compared to the control. However, a sensitivity analysis revealed omitting two studies (weight lifting [82] and rowing) [58] independently led to a significant effect favouring better IVD health with combined physical loading. This suggests that overall physical loading may be beneficial for IVD health and supports existing mechanistic theories that the maintenance of IVD metabolism is promoted through physical loading [4, 100]. Daily movement creates a pumping action that helps transport fluid and nutrients through the cartilage endplates [4], whereas supine rest allows for water reabsorption, leading to nutrients being returned to the IVD through diurnal fluctuations in hydration [100]. Hence, physical loading is essential for maintaining IVD health, with a caveat that the relationship between physical loading and IVD health is not linear [5]. A U-shaped relationship is commonly used to illustrate the correlation between physical loading and IVD health, with both insufficient and excessive physical loading compromising IVD nutrient transport and metabolic activity [4]. Hence, weightlifting and rowing may represent physical loads that compromise IVD metabolism. Weightlifting produces substantial lumbar loading, with a range from 18,000–36,000 N when lifting 212335 kg [101], which is considerably larger than that observed during running (~ 4775 N at a 4.46-min/km pace) [102]. Rowing generates lumbar loads similar to running (~ 5051 N) [102], but the flexed posture associated with rowing transfers loading through the anterior aspect of the IVD, effectively doubling loads. This initiates asymmetric loading, which is associated with microtrauma [103]. This further supports the positive effect of aerobic physical loading after excluding rowing studies. Thus, various forms of physical loading may promote better IVD health; however, benefits diminish when spinal loads surpass a threshold.

The explosive loading in the end range of motion subgroup likely represented physical loads that exceed the threshold for optimal IVD health. This subgroup included conditions in which the lumbar spine was exposed to either high-magnitude loads or explosive forces at the end range of lumbar motion, for example, end-range lumbar flexion to extension in a weightlifting clean and press or combining end-range flexion and extension with explosive rotation in baseball pitching/hitting. Previous theories suggest that exposure to repeated torsion, flexion and complex loading manoeuvres can result in microtrauma, which often leads to asymmetrical changes and serves as a precursor to IVD degeneration progression [103]. However, our meta-analysis revealed no difference in IVD health compared to the control, albeit with wide confidence and very low certainty across the available four studies. However, closer examination of individual studies demonstrated an increased prevalence of IVD degeneration compared with controls with cricket [96], weightlifting [97], gymnastics [68, 73] and wrestling [68, 73]. These studies were not included in the meta-analysis because of the heterogeneity of categorical outcomes with differing definitions of what constituted degeneration. Whilst our meta-analysis disagrees with these individual study findings, we encourage caution when interpreting our results because of the limited data available. The explosive loading in the end-range subgroup included a variety of sports, such as weightlifting, baseball and martial arts; however, these individual sports likely place varying loads on the lumbar spine. Consistent methods of measuring IVD health across studies would allow for pooling of studies with the same physical loading exposure, to increase confidence in the effect sizes, for example, comparing weightlifting with weightlifting. This would enable more precise conclusions to be drawn on the impact of sports involving explosive loading in the end range of lumbar motion on IVD health.

Upright bipedal physical loading was associated with better IVD health compared with the control, with 80% of these studies including running physical loading. This suggests that running exercise training is appropriate for promoting IVD health. These observations align with past animal models, where running interventions increased IVD proteoglycan content and hydration in both rats [9] and dogs [7]. Biomechanically, running exposes spinal structures to axial compression in a cyclic loading pattern [104]. A loading rate equivalent to slow running has been suggested as optimal for an IVD matrix turnover and promoting IVD health [4]. Our analyses included cross-sectional studies with running exposure during general physical activity [94], long-distance training [86, 87] and team sports [84], and in both young [87] and middle-aged adults [86]. Thus, the substantial heterogeneity across studies implies that various types of running physical loading were associated with greater IVD health and that running was beneficial across different population groups. However, the most optimal running parameters remain unclear. A caveat to this observation is the inclusion of skiing exposure in the upright bipedal subgroup, albeit represented by a single study in the synthesis of six studies, and biomechanically has similar joint contact forces to the axial cyclic compression, like running [105]. However, running was represented in five of the studies; therefore, prospective running trials are needed to examine causation. These trials will also provide insight into the optimal running parameters to promote IVD health, for example, speed, distance and frequency, and are currently being conducted in individuals with chronic LBP [20].

Synthesis of categorical outcomes revealed some moderate and larger effect sizes for reduced disc height in cross-sectional studies of combined physical loading. However, because of the presence of very wide CIs and unrealistically large OR estimates, our confidence in the results is limited and likely influenced by sparse-data bias [106]. Many studies (65%) did not report objective continuous measures of IVD health and were included in the secondary synthesis only. Further, a subgroup meta-analysis was not feasible because of inconsistent categorical outcome reporting methods, including the use of divergent sum scores, binary cut-offs and dissimilar grading schemes. Reduced signal intensity synthesis was the only significant categorical analysis, indicating that combined physical loading was associated with reduced IVD signal intensity compared with the control. Notably, 40% of these studies were rated as having a high risk of bias, warranting cautious interpretation. Furthermore, consistent with 71% of studies reporting categorical outcomes, degeneration prevalence was typically defined using binary cut-offs [107]. Because of being based on structural changes that commonly occur over decades, these methods lack the sensitivity to detect more subtle changes, such as those occurring within the timeframe of a typical exercise training intervention [18]. Thus, the inability of categorical methods to assess IVD health and detect these small, yet potentially important physiological changes, supports the use of more reliable continuous measures.

Comparison of categorical grading schemes with continuous measures of IVD health revealed the latter had greater sensitivity, denoted by smaller CIs. The most frequent continuous method reported, T2, serves as a proxy for hydration status and the structural composition of the IVD [24]. T2 is reported as a time constant (ms) of the realignment of protons within water molecules to return to equilibrium following magnetisation [24]. Thus, increased hydration increases the time to reach equilibrium, resulting in a higher T2 value that reflects a healthier IVD [24]. T2 has received growing attention in the last 5 years, owing to its sensitivity to reflect distinct physical activities in populations [63]. Further, T2 has shown excellent long-term reliability observed in nine measurements over 368 days (intraclass correlation coefficient = 0.98) [108]. Studies comparing these continuous techniques are limited and have been suggested as unnecessary given the time, effort and cost required when each technique uses objective data measuring various aspects of IVD health [5]. Only 35% (*n* = 13) [19, 41, 56, 57, 63, 65, 66, 82, 84, 86, 87, 94] of the studies in the current review employed continuous objective methods to assess IVD health, highlighting a unique opportunity to re-analyse past MRI data using these techniques. While manual tracing remains time intensive, emerging automation of these processes shows promise for the future [109]. Future studies examining IVD health could consider employing these highly reliable and sensitive measures to facilitate more robust comparisons between studies, enabling a further subgroup analysis.

Insufficient data prevented the determination of statistical heterogeneity attributed to LBP status, and an outstanding question remains whether IVD health is causally linked with LBP and disability. Here, we summarise the clinical implications of our results within the broader literature. Individuals experiencing LBP are eight times more likely to have an IVD bulge and two times more likely to have IVD degeneration [2]. Several mechanisms are proposed to explain this link. For example, nerve ingrowth is evident with greater IVD degeneration [110]. This increases the number of mechano-receptors within the IVD, thus increasing the potential for afferent signalling pathways and nociception [111]. These are further activated through abnormal loading patterns due to structural IVD changes [111]. Elevated levels of inflammatory biomarkers have also been reported with IVD degeneration, which is another likely contributing factor [112]. However, the correlation between IVD degeneration and LBP is considered weak [112]. Magnetic resonance imaging data revealed that > 50% of asymptomatic individuals aged 30–39 years have IVD degeneration, with a higher prevalence correlating with increasing age [18]. Other causative rationales for the primary pain mechanism of LBP are attributed to other spinal structures, for example, facet joint [111], vertebral body [111] and paraspinal musculature [112], whilst other groups attribute LBP to several biopsychosocial factors [113]. Although degenerative changes in the IVD are increasingly recognised as a normal part of ageing, the stronger correlations in individuals experiencing LBP suggest that degenerative changes may contribute to LBP via mechanistic pathways in some cases. Therefore, this relationship should be explored further via a causal mediation analysis to understand the potential mechanistic role of improving IVD health on LBP.

The use of robust MRI-based methods for the continuous IVD outcomes represents a strength of the evidence; however, several limitations remain. Despite the growth in the number of studies assessing IVD changes and physical loading exposures, the cross-sectional design of most of the studies and the lack of controlled designs have prevented causal inference and markedly impacted the confidence in our effect estimates. Second, the heterogeneity of MRI protocols presents a limitation, with 28 studies failing to account for factors that may influence MRI data, such as diurnal fluctuations and same-day physical activity. Further, overall data paucity led to: (1) small subgroups with potential small-sample and sparse-data bias, (2) limitations in heterogeneity testing and (3) reliance on the standardised mean difference because of heterogeneity in outcomes, which prevented the use of confounder-adjusted effect sizes. Last, IVD health and LBP do not always correlate, which limits the clinical implications of these findings, although exercise has well-established efficacy for LBP and overall health [114]. The review process was strengthened by the meta-analytical methods, which accounted for data paucity by including data from multiple outcomes in one meta-analysis. The limitations of the review process include the post hoc addition of subgroup exposure groups based on data availability, which constitutes a protocol deviation and introduces potential bias. The small number of studies further limited the accuracy of between-study variance estimation (tau2), therefore affecting the reliability of the pooled effect. Finally, categorising sports with varied spinal loading patterns poses a risk of exposure misclassification, potentially obscuring biomechanical differences such as those between pitching and batting in baseball.

## Conclusions

Exposure to combined types of physical loading revealed better IVD health when physical loads that might exceed a certain threshold of IVD loading were omitted.  This suggests the type of physical loading is an important factor in facilitating both IVD health and degeneration. Specifically, a prior history of upright bipedal physical loading, like running, was associated with better IVD health, revealing a small positive effect. However, no other physical loading subgroup was significantly associated with IVD health when all studies were included. The scarcity of longitudinal studies underscores the need for prospective running interventions to examine the causal effects of upright bipedal loading. Last, employing continuous measures of IVD health will likely produce greater sensitivity for detecting IVD health compared with categorical scales of IVD degeneration.

## Supplementary Information

Below is the link to the electronic supplementary material.

Supplementary file1 (DOCX 1653 KB)

## Declarations

### Availability of data and material

All data are publicly available via repositories (https://osf.io/IVDEX).

### Code availability

The statistical code is publicly available via repositories (https://osf.io/IVDEX).

### Patient and public involvement

Patients and/or the public were not involved in the design, conduct, reporting or dissemination plans of this research.
