# Supplementary material for: The Impact of Exercise on Intervertebral Disc Health: A Systematic Review and Meta-Analysis
Source: Sports Med. 2026 Mar 16;56(4):941–65. doi: 10.1007/s40279-025-02336-w (PMC13124787; doi:10.1007/s40279-025-02336-w)
Supplement: Supplementary file 1 — Supplementary file1 (DOCX 1653 KB) [file 40279_2025_2336_MOESM1_ESM.docx]

**SUPPLEMENT**

[Supplement A: Classifications of exposures in review 2](#_gvc1xzyu2izi)

[Supplement B: Search terms and database search results 3](#_i88xspakm5zx)

[Supplement C: Data handling 7](#_4qthtia2afdu)

[Supplement D: Data requests 39](#_nfpyr4bk72h5)

[Supplement E: Statistical code and data 40](#_vra35x6i3zd7)

[Supplement F: Operationalisation of GRADE criteria 76](#_nivuaesz5cpb)

[Supplement G: Reasons for exclusion at full-text screening 78](#_ij8j065ftlm)

[Supplement H: Forest plots for primary and secondary synthesis. 84](#_g69bn9nfrrkc)

[Supplement I: Random-effects meta-analysis sensitivity analyses for primary syntheses continuous IVD health and combined physical loading compared to control. 92](#_s9pokr7s5fr0)

[Supplement J Random-effects meta-analysis sensitivity analyses for primary sub-group syntheses if continuous IVD health 94](#_iwrrzdteiw4h)

[Supplement K: Random-effects meta-analysis sensitivity analyses for secondary synthesis IVD degeneration according to average Pfirrmann grade compared to controls. 97](#_etbcu39zsrh6)

[Supplement L: Random-effects meta-analysis sensitivity analyses for secondary synthesis IVD degeneration according to prevalence of Pfirrmann grade >2 compared to controls. 98](#_pmocy0f3a2pz)

[Supplement M: Random-effects meta-analysis sensitivity analyses for secondary synthesis according to prevalence of other measures of IVD compared to control. 99](#_6ur6r5fjqda6)

[Supplement N: Funnel plots for primary and secondary syntheses where n≥10 100](#_3b05763is49r)

[Supplement O: Trim-and-fill funnel plots for primary and secondary syntheses where n≥10 101](#_rdmq8v5bqxan)

[Supplement P: Protocol amendments since registration 102](#_q6gtktwyqb53)

#

# Supplement A: Classifications of exposures in review

Physical loading exposures defined

**Upright bipedal**: Included sport and exercise exposures with rhythmic, cyclic movements in an upright axial position, typically where running was the predominate activity.

**combined physical loading:** any type of exercise training, sport participation or physical activity.

**Nonupright noncontact:** Sport and exercise interventions were where the body position is not standing and with no physical contact.

**Extreme range of motion:** Sport and exercise interventions include movements involving power-based movements involving extreme ranges of spinal motion.

**High vs low physical activity**: Exposures record as physical activity or metabolic expenditure where there was a higher volume (exposure) and lower volume (control) of physical activity.

**Aerobic physical loading:** sport, exercise of physical activity relying predominantly on the aerobic energy system or is continuous in nature (without breaks) for greater than one minute.

# Supplement B: Search terms and database search results

Search was from inception to 30/09/2022 and was updated on 30.01.2024 and again on 03.06.2025 limiting the search from the prior search date to the current date.

| **MEDLINE (Pubmed, searched on 03.06.2025)** | |  |
| --- | --- | --- |
| **Search** | **Query** | **Hits** |
| #1 | "intervertebral dis*"[Title/Abstract] OR "disc degeneration"[Title/Abstract] OR "disk degeneration"[Title/Abstract] | 25282 |
| #2 | "exercise"[MeSH Terms] OR "exercise"[All Fields] OR "exercises"[All Fields] OR "exercise therapy"[MeSH Terms] OR ("exercise"[All Fields] AND "therapy"[All Fields]) OR "exercise therapy"[All Fields] OR "exercise's"[All Fields] OR "exercised"[All Fields] OR "exerciser"[All Fields] OR "exercisers"[All Fields] OR "exercising"[All Fields] | 635849 |
| #3 | ("physical"[All Fields] AND "activity"[All Fields]) OR "physical activity"[All Fields] | 316354 |
| #4   | "sport's"[All Fields] OR "sports"[MeSH Terms] OR "sports"[All Fields] OR "sport"[All Fields] OR "sporting"[All Fields] | 500180 |
| #5 | "athlete's"[All Fields] OR "athletes"[MeSH Terms] OR "athletes"[All Fields] OR "athlete"[All Fields] OR "athletically"[All Fields] OR "athlets"[All Fields] OR "athletic"[All Fields] OR "athletics"[All Fields] | 127874 |
| #6 | #2 OR #3 OR #4 OR #5 | 1076162 |
| #7 | animals[MeSH Terms] NOT humans[MeSH Terms] | 5,342,601 |
| #8 | ((#1 AND #6) NOT #7) | 898 |
| #9 | #8 AND 2024/01/30:2025/06/03[Date - Publication] | 136 |

| **CENTRAL: (searched on 03.06.2025)** | |  |
| --- | --- | --- |
| **Search** | **Query** | **Hits** |
| #1 | (intervertebral dis*):ti,ab,kw OR (disc degeneration):ti,ab,kw OR (disk degeneration):ti,ab,kw | 4619 |
| #2 | MeSH descriptor: [exercise] explode all trees | 40108 |
| #3 | MeSH descriptor: [sports] explode all trees | 22743 |
| #4 | athlet*:ti,ab,kw | 15121 |
| #5 | #2 or #3 or #4 | 56247 |
| #6 | #1 and #5 | 48 |
| #7 | Limits: Trials with Cochrane Library publication date from Feb 2024 to Jun 2025 | 4 |

| **EMBASE (limit: excluding medline, searched on 03.06.2025):** | |  |
| --- | --- | --- |
| **Search** | **Query** | **Hits** |
| #1 | intervertebral dis*.ab,ti. OR disc degeneration.ab,ti OR disk degeneration.ab,ti | 27535 |
| #2 | exp exercise/ OR exercis*.af. | 824210 |
| #3 | (physical.af. AND activity.af.) OR physical activity.af. | 610052 |
| #4 | exp sport/ OR sport*.af. | 530610 |
| #5 | exp athlete/ OR athlet*.af. OR athletic*.af. | 148729 |
| #6 | 2 or 3 or 4 or 5 | 1583463 |
| #7 | 1 and 6 | 1740 |
| #8 | limit 7 to "remove medline records" | 728 |
| #9 | 7 not 8 | 1012 |
| #10 | \|  \| limit 9 to dd=20240131-20250603 \| \| --- \| --- \| | 105 |

| **CINAHL (no limits, searched on 03.06.2025)** | |  |
| --- | --- | --- |
| **Search** | **Query** | **Hits** |
| #1 | TI (intervertebral dis*) OR AB (intervertebral dis*) OR TI (disc degeneration) OR AB (disc degeneration) OR TI (disk degeneration) OR AB (disk degeneration) | 5152 |
| #2 | (MH "Exercise+") OR TX (exercis*) | 495030 |
| #3 | MH physical activity+ OR (TX physical AND TX activity) | 426921 |
| #4 | MH sport+ OR TX sport* | 311421 |
| #5 | MH athlete+ OR TX athlet* OR TX athletic* | 118636 |
| #6 | S2 or S3 or S4 or S5 | 911015 |
| #7 | S1 and S6 | 587 |
| #8 | S7 and EM 20240130-20250603 | 38 |

| **SPORTDiscus (no limits, searched on 03.06.2025)** | |  |
| --- | --- | --- |
| **Search** | **Query** | **Hits** |
| #1 | TI (intervertebral dis*) OR AB (intervertebral dis*) OR TI (disc degeneration) OR AB (disc degeneration) OR TI (disk degeneration) OR AB (disk degeneration) | 1,081 |
| #2 | (MH "Exercise+") OR TX (exercis*) | 320523 |
| #3 | MH physical activity+ OR (TX physical AND TX activity) | 122096 |
| #4 | MH sport+ OR TX sport* | 1164852 |
| #5 | MH athlete+ OR TX athlet* OR TX athletic* | 486756 |
| #6 | S2 or S3 or S4 or S5 | 1466416 |
| #7 | S1 and S6 | 275 |
| #8 | S7 and EM 20240130-20250603 | 0 |

| **Web of Science (search on 03.06.2025)** | |  |
| --- | --- | --- |
| **Search** | **Query** | **Hits** |
| #1 | ts=(intervertebral dis*" OR "disc degeneration" OR "disk degeneration) | 426 |
| #6 | (ts=("exercise" OR "exercises" OR "exercise therapy" OR ("exercise" AND "therapy") OR "exercise's" OR "exercised" OR "exerciser" OR "exercisers" OR "exercising")) OR (ts=("physical activity" OR ("physical" AND "activity"))) OR (ts=("sport" OR "sports" OR "sporting")) OR (ts=("athlete" OR "athletes" OR "athletically" OR "athletic" OR "athletics")) | 1023780 |
| #7 | #1 AND #3 | 26 |
| #8 | limit Publication Date to 2024-01-30 to 2025-06-03 | 2 |

| **Scopus (search on 03.06.2025)** | |  |
| --- | --- | --- |
| **Search** | **Query** | **Hits** |
| #1 | TITLE-ABS ("intervertebral dis*" OR "disc degeneration" OR "disk degeneration" ) | 28,262 |
| #2 | TITLE-ABS ("exercise" OR "exercises" OR "exercise therapy" OR "exercised" OR "exerciser" OR "exercisers" OR "exercising" ) | 683,023 |
| #3 | TITLE-ABS ( "physical activity" OR ( "physical" AND "activity" ) | 460,989 |
| #4 | TITLE-ABS ( "sport" OR "sports" OR "sporting" ) | 297,094 |
| #5 | TITLE-ABS ("athlete" OR "athletes" OR "athletically" OR "athletic" OR "athletics" ) | 157,454 |
| #6 | TITLE-ABS ( "intervertebral dis*" OR "disc degeneration" OR "disk degeneration" ) AND (TITLE-ABS ("exercise" OR "exercises" OR "exercise therapy" OR "exercised" OR "exerciser" OR "exercisers" OR "exercising") OR TITLE-ABS ("physical activity" OR ("physical" AND "activity")) OR TITLE-ABS ("sport" OR "sports" OR "sporting") OR TITLE-ABS ("athlete" OR "athletes" OR "athletically" OR "athletic" OR "athletics") AND NOT TITLE-ABS-KEY ("animal"*)) | 798 |

| **Trial Registry: NIH (Searched on 10.02.2024)** | | |
| --- | --- | --- |
| **Search** | **Query** | **Hits** |
| Condition or disease |  |  |
| Other terms | (intervertebral disc) OR (intervertebral disk) OR (disc degeneration) OR (disk degeneration) |  |
| Intervention/treatment | exercise OR sport OR physical activity |  |
| Outcome measures | (magnetic resonance) OR MRI OR X-ray OR radiolog* OR CT OR computed tomogr* |  |
|  |  | 20 |

| **Trial Registry: WHO (Searched on 14.02.2024)** | | |
| --- | --- | --- |
| **Search string:** | **Query** | **Hits** |
| Condition or disease | ((intervertebral disc) OR (intervertebral disk) OR (disc degeneration) OR (disk degeneration)) AND (exercise OR sport OR physical activity) |  |
|  |  | 19 |

|  |  | **TOTAL from all searches** | | | | | | | | |  |
| --- | --- | --- | --- | --- | --- | --- | --- | --- | --- | --- | --- |
| **Databases (03.06.2025)** | | | Pubmed | Embase | CENTRAL | Cinahl | Sportdiscus | **Web of Science** | **Scopus** | **Total** | |
|  | | | 948 | 767 | 44 | 646 | 275 | 26 | 798 | 3504 | |
| **Trial Registries(01.10.2022 & 10.02.2024)** | | |  | NH |  | Who |  |  |  |  | |
|  | | |  | 20 |  | 19 |  |  |  | 39 | |
| **Duplicates removed (by Covidence)** | | |  |  |  |  |  |  |  | 1356 | |
| **Total (with duplicates removed)** | | |  |  |  |  |  |  |  | 2187 | |

**Prior Reviews**

Search via Pubmed on 05.10.2022 and 30/01/24 [(exercise OR sport OR physical activity) AND (intervertebral disc) AND ("review"[Title/Abstract]))](https://pubmed.ncbi.nlm.nih.gov/?term=(exercise%20OR%20sport%20OR%20physical%20activity)%20AND%20(intervertebral%20disc)%20AND%20(%22review%22%5BTitle%252FAbstract%5D))&size=100) and GoogleScholar "[exercise intervertebral disc review](https://scholar.google.com/scholar?hl=en&as_sdt=0%2C5&q=exercise+intervertebral+disc+review&btnG=)"

- Li, Yang, Wang, Liu (2021) Stem Cell Therapy and Exercise for Treatment of Intervertebral Disc Degeneration Stem Cells Int 2021:7982333 <https://doi.org/10.1155/2021/7982333> (DONE/UPLOADED 05.10.2022)
- Steele, Bruce-Low, Smith, Osborne, Thorkeldsen (2015) Can specific loading through exercise impart healing or regeneration of the intervertebral disc? The Spine Journal 15: 2117-2121 <https://doi.org/10.1016/j.spinee.2014.08.446> (DONE/UPLOADED 05.10.2022)
- Mitchell UH, Helgeson K, Mintken P (2017) Physiological effects of physical therapy interventions on lumbar intervertebral discs: A systematic review. Physiother Theory Pract 33(9):695-705 <https://doi.org/10.1080/09593985.2017.1345026> (DONE/UPLOADED 05.10.2022)
- Belavý DL, Albracht K, Bruggemann G-P, Vergroesen P-P A, van Dieën JH (2016): Can exercise positively influence the intervertebral disc? Sports Medicine 46(4): 473-485. <http://dx.doi.org/10.1007/s40279-015-0444-2> (DONE/UPLOADED 05.10.2022)
- Olin T, Wells A (2021): Conservative Treatments on Intervertebral Disc Hydration: A Scoping Review.  Franciscan Missionaries of Our Lady University ProQuest Dissertations Publishing,  2022. 28864737 <https://www.proquest.com/openview/88f38a852954cda1d93e4d2f52fbb260/1>. Ordered via library, as only preview available
- Chang, MC., Park, D., Kim, JH., & Choo, YJ. (2024). Effect of exercise on stabilizing and strengthening core muscles for patients with herniated lumbar disc: A systematic review and meta-analysis. Asian journal of surgery, 47(1), 731-733. <https://pubmed.ncbi.nlm.nih.gov/38135533/> (DONE 30/01/2024)
- Shu D, Dai S, Wang J, Meng F, Zhang C, Zhao Z. (2024) Impact of Running Exercise on Intervertebral Disc: A Systematic Review. Sports Health. Published online. <https://journals.sagepub.com/doi/abs/10.1177/19417381231221125.> (DONE 30/01/2024)

# Supplement C: Data handling

|  | |  | |  | | | | | | | | **Physical loading group** | | | | | | | **Control group** | | | | | | | | | | |  | | | |  | | | | | | |  |  |  |
| --- | --- | --- | --- | --- | --- | --- | --- | --- | --- | --- | --- | --- | --- | --- | --- | --- | --- | --- | --- | --- | --- | --- | --- | --- | --- | --- | --- | --- | --- | --- | --- | --- | --- | --- | --- | --- | --- | --- | --- | --- | --- | --- | --- |
| **Study** | | **Outcomes** | | **Level** | | **Mean** | | | | | | **SD** | | | **N** | | | **Mean** | | | | **SD** | | | | | **N** | | |  | | | | **Data source** | | | | | | |  |  |  |
| **Primary synthesis: combined physical loading** | | | |  | |  | | | | | |  | | |  | | |  | | | |  | | | | |  | | |  | | | | | | | | | | |  |  |  |
| Belavy 2017, 2019 & 2020 | | IVD anteroposterior width | | LxAx | | 26.25 | | | | | | 3.16 | | | 73 | | | 25.6 | | | | 2.4 | | | | | 24 | | | Averaged from 2 reports: 2017: Table 1, pooled 20-40km and 50km running groups and 2019: Table 2 | | | | | | | | | | |  |  |  |
| Belavy 2017, 2019 & 2020 | | IVD average area | | LxAx | | 263.33 | | | | | | 59.39 | | | 55 | | | 250.2 | | | | 41.2 | | | | | 24 | | | Averaged from 2 reports: 2017: Table 1, pooled 20-40km and 50km running groups and 2019: Table 2 | | | | | | | | | | |  |  |  |
| Belavy 2017, 2019 & 2020 | | IVD average distance | | LxAx | | 34.31 | | | | | | 2.75 | | | 73 | | | 34.2 | | | | 2 | | | | | 24 | | | Averaged from 2 reports: 2017: Table 1, pooled 20-40km and 50km running groups and 2019: Table 2 | | | | | | | | | | |  |  |  |
| Belavy 2017, 2019 & 2020 | | IVD average volume | | LxAx | | 10.30 | | | | | | 3.14 | | | 73 | | | 9.5 | | | | 2.3 | | | | | 24 | | | Averaged from 2 reports: 2017: Table 1, pooled 20-40km and 50km running groups and 2019: Table 2 | | | | | | | | | | |  |  |  |
| Belavy 2017, 2019 & 2020 | | IVD height relative to vertebral body height | | LxAx | | 0.30 | | | | | | 0.05 | | | 73 | | | 0.28 | | | | 0.05 | | | | | 24 | | | Averaged from 2 reports: 2020: Imputed from Figure 2 (WebPlotDigitizer), pooled running groups, averaged levels and 2019: Table 2 | | | | | | | | | | |  |  |  |
| Belavy 2017, 2019 & 2020 | | IVD T2-time | | L1-L2 | | 116.24 | | | | | | 20.2 | | | 73 | | | 107.6 | | | | 6.62 | | | | | 24 | | | Averaged from 2 reports: 2017: Imputed from Figure 2 (WebPlotDigitizer), pooled running groups and 2019: accessed from author | | | | | | | | | | |  |  |  |
| Belavy 2017, 2019 & 2020 | | IVD T2-time | | L2-L3 | | 114.99 | | | | | | 17.08 | | | 73 | | | 108.8 | | | | 11.49 | | | | | 24 | | | Averaged from 2 reports: 2017: Imputed from Figure 2 (WebPlotDigitizer), pooled running groups and 2019: accessed from author | | | | | | | | | | |  |  |  |
| Belavy 2017, 2019 & 2020 | | IVD T2-time | | L3-L4 | | 111.19 | | | | | | 18.29 | | | 73 | | | 108.3 | | | | 12.61 | | | | | 24 | | | Averaged from 2 reports: 2017: Imputed from Figure 2 (WebPlotDigitizer), pooled running groups and 2019: accessed from author | | | | | | | | | | |  |  |  |
| Belavy 2017, 2019 & 2020 | | IVD T2-time | | L4-L5 | | 105.65 | | | | | | 18.93 | | | 73 | | | 100.1 | | | | 16.97 | | | | | 24 | | | Averaged from 2 reports: 2017: Imputed from Figure 2 (WebPlotDigitizer), pooled running groups and 2019: accessed from author | | | | | | | | | | |  |  |  |
| Belavy 2017, 2019 & 2020 | | IVD T2-time | | L5-S1 | | 104.91 | | | | | | 19.31 | | | 73 | | | 94.98 | | | | 20.71 | | | | | 24 | | | Averaged from 2 reports: 2017: Imputed from Figure 2 (WebPlotDigitizer), pooled running groups and 2019: accessed from author | | | | | | | | | | |  |  |  |
| Belavy 2017, 2019 & 2020 | | IVD T2-time nucleus/annulus ratio | | LxAx | | 1.49 | | | | | | 0.13 | | | 77 | | | 1.4 | | | | 0.15 | | | | | 24 | | | 2020 report: Accessed n from author, imputed SD from SE from Table 1, pooled all exercise groups | | | | | | | | | | |  |  |  |
| Belavy 2017, 2019 & 2020 | | IVD T2 weight signal intensity | | LxAx | | 322 | | | | | | 24.23 | | | 77 | | | 326.9 | | | | 24 | | | | | 24 | | | 2020: Accessed n from author, imputed SD from SE from Table 1, pooled all exercise groups | | | | | | | | | | |  |  |  |
| Belavy 2017, 2019 & 2020 | | IVD T2 weight signal intensity nucleus/annulus ratio | | LxAx | | 3.35 | | | | | | 0.34 | | | 77 | | | 3.31 | | | | 0.34 | | | | | 24 | | | 2020: Accessed n from author, imputed SD from SE from Table 1, pooled all exercise groups | | | | | | | | | | |  |  |  |
| Belavy 2017, 2019 & 2020 | | IVD Dixon water fraction | | LxAx | | 87.42 | | | | | | 3.19 | | | 77 | | | 86 | | | | 2.94 | | | | | 24 | | | 2020: Accessed n from author, imputed SD from SE from Table 1, pooled all exercise groups | | | | | | | | | | |  |  |  |
| Belavy 2017, 2019 & 2020 | | IVD Dixon water fraction nucleus/annulus ratio | | LxAx | | 1.01 | | | | | | 0.01 | | | 77 | | | 1.01 | | | | 0.01 | | | | | 24 | | | 2020: Accessed n from author, imputed SD from SE from Table 1, pooled all exercise groups | | | | | | | | | | |  |  |  |
| **Belavy 2017, 2019 & 2020** | | **Synthetic effect size: ρ \| Hedges’ g \| standard error** | | | | | | | | | | | | | | | | | | | | | | | | | | | |  | | | |  | | | | | | |  |  |  |
|  | | ρ=0.0 \| 0.27 \| 0.06 | |  | |  | | | | | |  | | |  | | |  | | | |  | | | | |  | | |  | | | |  | | | | | | |  |  |  |
|  | | ρ=0.2 \| 0.27 \| 0.06 | |  | |  | | | | | |  | | |  | | |  | | | |  | | | | |  | | |  | | | |  | | | | | | |  |  |  |
|  | | ρ=0.4 \| 0.27 \| 0.06 | |  | |  | | | | | |  | | |  | | |  | | | |  | | | | |  | | |  | | | |  | | | | | | |  |  |  |
|  | | ρ=0.6 \| 0.27 \| 0.06 | |  | |  | | | | | |  | | |  | | |  | | | |  | | | | |  | | |  | | | |  | | | | | | |  |  |  |
|  | | ρ=0.8 \| 0.27 \| 0.06 | |  | |  | | | | | |  | | |  | | |  | | | |  | | | | |  | | |  | | | |  | | | | | | |  |  |  |
|  | | ρ=1.0 \| 0.27 \| 0.06 | |  | |  | | | | | |  | | |  | | |  | | | |  | | | | |  | | |  | | | |  | | | | | | |  |  |  |
| Benedikter 2022 | | IVD T2- time | | L2-L3 | | 32.82 | | | | | | 30.31 | | | 17 | | | 37.99 | | | | 32.71 | | | | | 37 | | |  | | | | Table 2 | | | | | | |  |  |  |
| Benedikter 2022 | | IVD T2- time | | L3-L4 | | 35.94 | | | | | | 27.94 | | | 17 | | | 43.42 | | | | 32.49 | | | | | 37 | | |  | | | | Table 2 | | | | | | |  |  |  |
| Benedikter 2022 | | IVD T2- time | | L4-L5 | | 40.31 | | | | | | 34.98 | | | 17 | | | 52.50 | | | | 39.76 | | | | | 37 | | |  | | | | Table 2 | | | | | | |  |  |  |
| Benedikter 2022 | | IVD T2- time | | L5-S1 | | 39.05 | | | | | | 40.15 | | | 17 | | | 46.12 | | | | 35.09 | | | | | 37 | | |  | | | | Table 2 | | | | | | |  |  |  |
| **Benedikter 2022** | | **Synthetic effect size: ρ \| Hedges’ g \| standard error** | | | | | | | | | | | | | | | | | | | | | | | | | | | |  | | | |  | | | | | | |  |  |  |
|  | | ρ=0.0 \| -0.22 \| 0.15 | |  | |  | | | | | |  | | |  | | |  | | | |  | | | | |  | | |  | | | | | | | | | | |  |  |  |
|  | | ρ=0.2 \| -0.22 \| 0.15 | |  | |  | | | | | |  | | |  | | |  | | | |  | | | | |  | | |  | | | | | | | | | | |  |  |  |
|  | | ρ=0.4 \| -0.22 \| 0.15 | |  | |  | | | | | |  | | |  | | |  | | | |  | | | | |  | | |  | | | | | | | | | | |  |  |  |
|  | | ρ=0.6 \| -0.22 \| 0.15 | |  | |  | | | | | |  | | |  | | |  | | | |  | | | | |  | | |  | | | | | | | | | | |  |  |  |
|  | | ρ=0.8 \| -0.22 \| 0.15 | |  | |  | | | | | |  | | |  | | |  | | | |  | | | | |  | | |  | | | | | | | | | | |  |  |  |
|  | | ρ=1.0 \| -0.22 \| 0.15 | |  | |  | | | | | |  | | |  | | |  | | | |  | | | | |  | | |  | | | | | | | | | | |  |  |  |
| Bowden 2018 | | IVD Fractional anisotropy | | LxAx | | 0.15 | | | | | | 0.06 | | | 14 | | | 0.17 | | | | 0.11 | | | | | 12 | | | Table 3 | | | | | | | | | | |  |  |  |
| Bowden 2018 | | IVD apparent diffusion coefficient | | LxAx | | 1.15 | | | | | | 0.48 | | | 14 | | | 0.79 | | | | 0.49 | | | | | 12 | | | Table 3 | | | | | | | | | | |  |  |  |
| Bowden 2018 | | IVD T2 signal intensity | | LxAx | | 55.8 | | | | | | 24.7 | | | 14 | | | 49.7 | | | | 21.4 | | | | | 12 | | | Table 3 | | | | | | | | | | |  |  |  |
| **Bowden 2018** | | **Synthetic effect size: ρ \| Hedges’ g \| standard error** | | | | | | | | | | | | | | | | | | | | | | | | | | | |  | | | |  | | | | | | |  |  |  |
|  | | ρ=0.0 \| 0.40 \| 0.23 | |  | |  | | | | | |  | | |  | | |  | | | |  | | | | |  | | |  | | | |  | | | | | | |  |  |  |
|  | | ρ=0.2 \| 0.40 \| 0.24 | |  | |  | | | | | |  | | |  | | |  | | | |  | | | | |  | | |  | | | |  | | | | | | |  |  |  |
|  | | ρ=0.4 \| 0.40 \| 0.24 | |  | |  | | | | | |  | | |  | | |  | | | |  | | | | |  | | |  | | | |  | | | | | | |  |  |  |
|  | | ρ=0.6 \| 0.40 \| 0.25 | |  | |  | | | | | |  | | |  | | |  | | | |  | | | | |  | | |  | | | |  | | | | | | |  |  |  |
|  | | ρ=0.8 \| 0.40 \| 0.25 | |  | |  | | | | | |  | | |  | | |  | | | |  | | | | |  | | |  | | | |  | | | | | | |  |  |  |
|  | | ρ=1.0 \| 0.40 \| 0.26 | |  | |  | | | | | |  | | |  | | |  | | | |  | | | | |  | | |  | | | |  | | | | | | |  |  |  |
| Frenken 2022 | | IVD glycosaminoglycan content (%) | | LxAx | | 2.58 | | | | | | 0.27 | | | 17 | | | 1.66 | | | | 0.38 | | | | | 44 | | | Results section (pg.3) | | | | | | | | | | |  |  |  |
| Jentzsch 2020 | | IVD T2-values average whole spine | | LxAx | | 85.60 | | | | | | 14.90 | | | 12 | | | 87.20 | | | | 13.50 | | | | | 13 | | | Table 2 (evening) | | | | | | | | | | |  |  |  |
| Lagerstrand 2021 & Rosenqvist 2023 | | IVD T2 value subregion 1 | | Lx/Tx Ax | | 0.74 | | | | | | 0.33 | | | 58 | | | 0.76 | | | | 0.34 | | | | | 26 | | | 2021 report: Imputed from Figure 3 (WebPlotDigitizer) | | | | | | | | | | |  |  |  |
| Lagerstrand 2021 & Rosenqvist 2023 | | IVD T2 value subregion 2 | | Lx/Tx Ax | | 1.06 | | | | | | 0.35 | | | 58 | | | 1.14 | | | | 0.4 | | | | | 26 | | | 2021 report: Imputed from Figure 3 (WebPlotDigitizer) | | | | | | | | | | |  |  |  |
| Lagerstrand 2021 & Rosenqvist 2023 | | IVD T2 value subregion 3 | | Lx/Tx Ax | | 1.3 | | | | | | 0.48 | | | 58 | | | 1.33 | | | | 0.43 | | | | | 26 | | | 2021 report: Imputed from Figure 3 (WebPlotDigitizer) | | | | | | | | | | |  |  |  |
| Lagerstrand 2021 & Rosenqvist 2023 | | IVD T2 value subregion 4 | | Lx/Tx Ax | | 1.13 | | | | | | 0.44 | | | 58 | | | 1.12 | | | | 0.41 | | | | | 26 | | | 2021 report: Imputed from Figure 3 (WebPlotDigitizer) | | | | | | | | | | |  |  |  |
| Lagerstrand 2021 & Rosenqvist 2023 | | IVD T2 value subregion 5 | | Lx/Tx Ax | | 0.53 | | | | | | 0.28 | | | 58 | | | 0.47 | | | | 0.23 | | | | | 26 | | | 2021 report: Imputed from Figure 3 (WebPlotDigitizer) | | | | | | | | | | |  |  |  |
| **Lagerstrand 2021** | | **Synthetic effect size: ρ \| Hedges’ g \| standard error** | | | | | | | | | | | | | | | | | | | | | | | | | | | | | | | | | | | | | | |  |  |  |
|  | | ρ=0.0 \| -0.02 \| 0.11 | |  | |  | | | | | |  | | |  | | |  | | | |  | | | | |  | | |  | | | | | | | | | | |  |  |  |
|  | | ρ=0.2 \| -0.02 \| 0.11 | |  | |  | | | | | |  | | |  | | |  | | | |  | | | | |  | | |  | | | | | | | | | | |  |  |  |
|  | | ρ=0.4 \| -0.02 \| 0.11 | |  | |  | | | | | |  | | |  | | |  | | | |  | | | | |  | | |  | | | | | | | | | | |  |  |  |
|  | | ρ=0.6 \| -0.02 \| 0.11 | |  | |  | | | | | |  | | |  | | |  | | | |  | | | | |  | | |  | | | | | | | | | | |  |  |  |
|  | | ρ=0.8 \| -0.02 \| 0.11 | |  | |  | | | | | |  | | |  | | |  | | | |  | | | | |  | | |  | | | | | | | | | | |  |  |  |
|  | | ρ=1.0 \| -0.02 \| 0.11 | |  | |  | | | | | |  | | |  | | |  | | | |  | | | | |  | | |  | | | | | | | | | | |  |  |  |
| Li 2024 | | IVD T2 average annulus | | L1-L2 | | 21.54 | | | | | | 4.47 | | | 54 | | | 19.42 | | | | 3.68 | | | | | 30 | | | Imputed from Figure 4A (WebPlotDigitizer), | | | | | | | | | | |  |  |  |
| Li 2024 | | IVD T2 average annulus | | L2-L3 | | 22.5 | | | | | | 5.07 | | | 54 | | | 21.28 | | | | 5.09 | | | | | 30 | | | Imputed from Figure 4A (WebPlotDigitizer), | | | | | | | | | | |  |  |  |
| Li 2024 | | IVD T2 average annulus | | L3-L4 | | 21.99 | | | | | | 4.72 | | | 54 | | | 19.35 | | | | 3.47 | | | | | 30 | | | Imputed from Figure 4A (WebPlotDigitizer), | | | | | | | | | | |  |  |  |
| Li 2024 | | IVD T2 average annulus | | L4-L5 | | 23.3 | | | | | | 5.3 | | | 54 | | | 21.68 | | | | 4.58 | | | | | 30 | | | Imputed from Figure 4A (WebPlotDigitizer), | | | | | | | | | | |  |  |  |
| Li 2024 | | IVD T2 average annulus | | L5-S1 | | 21.59 | | | | | | 5.55 | | | 54 | | | 19.85 | | | | 4.51 | | | | | 30 | | | Imputed from Figure 4A (WebPlotDigitizer), | | | | | | | | | | |  |  |  |
| Li 2024 | | IVD T2 average nucleus | | L1-L2 | | 42.23 | | | | | | 11.27 | | | 54 | | | 39.95 | | | | 10.72 | | | | | 30 | | | Imputed from Figure 4B (WebPlotDigitizer), | | | | | | | | | | |  |  |  |
| Li 2024 | | IVD T2 average nucleus | | L2-L3 | | 47.51 | | | | | | 7.74 | | | 54 | | | 44.71 | | | | 13.51 | | | | | 30 | | | Imputed from Figure 4B (WebPlotDigitizer), | | | | | | | | | | |  |  |  |
| Li 2024 | | IVD T2 average nucleus | | L3-L4 | | 49.34 | | | | | | 13.4 | | | 54 | | | 47.01 | | | | 15.79 | | | | | 30 | | | Imputed from Figure 4B (WebPlotDigitizer), | | | | | | | | | | |  |  |  |
| Li 2024 | | IVD T2 average nucleus | | L4-L5 | | 51.21 | | | | | | 15.94 | | | 54 | | | 43.3 | | | | 15.84 | | | | | 30 | | | Imputed from Figure 4B (WebPlotDigitizer), | | | | | | | | | | |  |  |  |
| Li 2024 | | IVD T2 average nucleus | | L5-S1 | | 47.66 | | | | | | 18.48 | | | 54 | | | 44.16 | | | | 17.41 | | | | | 30 | | | Imputed from Figure 4B (WebPlotDigitizer), | | | | | | | | | | |  |  |  |
| **Li 2024** | | **Synthetic effect size: ρ \| Hedges’ g \| standard error** | | | | | | | | | | | | | | | | | | | |  | | | | |  | | |  | | | | | | | | | | |  |  |  |
|  | | ρ=0.0 \| 0.33 \| 0.07 | |  | |  | | | | | |  | | |  | | |  | | | |  | | | | |  | | |  | | | | | | | | | | |  |  |  |
|  | | ρ=0.2 \| -0.33 \| 0.07 | |  | |  | | | | | |  | | |  | | |  | | | |  | | | | |  | | |  | | | | | | | | | | |  |  |  |
|  | | ρ=0.4 \| 0.33 \| 0.07 | |  | |  | | | | | |  | | |  | | |  | | | |  | | | | |  | | |  | | | | | | | | | | |  |  |  |
|  | | ρ=0.6 \| 0.33 \| 0.07 | |  | |  | | | | | |  | | |  | | |  | | | |  | | | | |  | | |  | | | | | | | | | | |  |  |  |
|  | | ρ=0.8 \| 0.33 \| 0.07 | |  | |  | | | | | |  | | |  | | |  | | | |  | | | | |  | | |  | | | | | | | | | | |  |  |  |
|  | | ρ=1.0 \| 0.33 \| 0.07 | |  | |  | | | | | |  | | |  | | |  | | | |  | | | | |  | | |  | | | | | | | | | | |  |  |  |
| Mitchell 2020 | | Apparent diffusion coefficient | | L5-S1 | | 249 | | | | | | 175.2 | | | 9 | | | 202.3 | | | | 149.5 | | | | | 8 | | | Table 3 | | | | | | | | | | |  |  |  |
| Mitchell 2020 | | IVD height to vertebral body height ratio | | L2-L3 | | 0.33 | | | | | | 0.04 | | | 9 | | | 0.23 | | | | 0.02 | | | | | 8 | | | Table 4 | | | | | | | | | | |  |  |  |
| Mitchell 2020 | | IVD height to vertebral body height ratio | | L3-L4 | | 0.32 | | | | | | 0.05 | | | 9 | | | 0.25 | | | | 0.03 | | | | | 8 | | | Table 4 | | | | | | | | | | |  |  |  |
| Mitchell 2020 | | IVD height to vertebral body height ratio | | L4-L5 | | 0.31 | | | | | | 0.06 | | | 9 | | | 0.26 | | | | 0.04 | | | | | 8 | | | Table 4 | | | | | | | | | | |  |  |  |
| Mitchell 2020 | | IVD height to vertebral body height ratio | | L5-S1 | | 0.28 | | | | | | 0.05 | | | 9 | | | 0.22 | | | | 0.04 | | | | | 8 | | | Table 4 | | | | | | | | | | |  |  |  |
| Mitchell 2020 | | IVD T2- time average | | L2-L3 | | 101.2 | | | | | | 13.6 | | | 9 | | | 93.1 | | | | 26.6 | | | | | 8 | | | Table 4 | | | | | | | | | | |  |  |  |
| Mitchell 2020 | | IVD T2- time average | | L3-L4 | | 95.1 | | | | | | 13.9 | | | 9 | | | 89.6 | | | | 26.8 | | | | | 8 | | | Table 4 | | | | | | | | | | |  |  |  |
| Mitchell 2020 | | IVD T2- time average | | L4-L5 | | 83.9 | | | | | | 12.2 | | | 9 | | | 85.7 | | | | 32.7 | | | | | 8 | | | Table 4 | | | | | | | | | | |  |  |  |
| Mitchell 2020 | | IVD T2- time average | | L5-S1 | | 85.1 | | | | | | 14.8 | | | 9 | | | 78.8 | | | | 14.9 | | | | | 8 | | | Table 4 | | | | | | | | | | |  |  |  |
| **Mitchell 2020** | | **Synthetic effect size: ρ \| Hedges’ g \| standard error** | | | | | | | | | | | | | | | | | | | | | | | | | | | | | | | | | | | | | | |  |  |  |
|  | | ρ=0.0 \| 0.88 \| 0.18 | | | | | | | | | | | | | | | |  | | | | | | | | | | | |  | | | |  | | | | | | |  |  |  |
|  | | ρ=0.2 \| 0.88 \| 0.18 | |  | |  | | | | | |  | | |  | | |  | | | |  | | | | |  | | |  | | | | | | | | | | |  |  |  |
|  | | ρ=0.4 \| 0.88 \| 0.18 | |  | |  | | | | | |  | | |  | | |  | | | |  | | | | |  | | |  | | | | | | | | | | |  |  |  |
|  | | ρ=0.6 \| 0.88 \| 0.18 | |  | |  | | | | | |  | | |  | | |  | | | |  | | | | |  | | |  | | | | | | | | | | |  |  |  |
|  | | ρ=0.8 \| 0.88 \| 0.18 | |  | |  | | | | | |  | | |  | | |  | | | |  | | | | |  | | |  | | | | | | | | | | |  |  |  |
|  | | ρ=1.0 \| 0.88 \| 0.18 | |  | |  | | | | | |  | | |  | | |  | | | |  | | | | |  | | |  | | | | | | | | | | |  |  |  |
| Hangai 2009 & Owen 2021 | | IVD height to vertebral body height ratio | | L1-L2 | | 0.32 | | | | | | 0.04 | | | 308 | | | 0.31 | | | | 0.04 | | | | | 71 | | | 2021 report Table 2, pooled all sport groups | | | | | | | | | | |  |  |  |
| Hangai 2009 & Owen 2021 | | IVD height to vertebral body height ratio | | L2-L3 | | 0.36 | | | | | | 0.05 | | | 308 | | | 0.33 | | | | 0.05 | | | | | 71 | | | 2021 report Table 2, pooled all sport groups | | | | | | | | | | |  |  |  |
| Hangai 2009 & Owen 2021 | | IVD height to vertebral body height ratio | | L3-L4 | | 0.41 | | | | | | 0.05 | | | 308 | | | 0.38 | | | | 0.05 | | | | | 71 | | | 2021 report Table 2, pooled all sport groups | | | | | | | | | | |  |  |  |
| Hangai 2009 & Owen 2021 | | IVD height to vertebral body height ratio | | L4-L5 | | 0.46 | | | | | | 0.07 | | | 308 | | | 0.43 | | | | 0.07 | | | | | 71 | | | 2021 report Table 2, pooled all sport groups | | | | | | | | | | |  |  |  |
| Hangai 2009 & Owen 2021 | | IVD height to vertebral body height ratio | | L5-S1 | | 0.43 | | | | | | 0.07 | | | 308 | | | 0.43 | | | | 0.08 | | | | | 71 | | | 2021 report Table 2, pooled all sport groups | | | | | | | | | | |  |  |  |
| Hangai 2009 & Owen 2021 | | IVD nucleus-annulus signal intensity ratio | | L1-L2 | | 3.83 | | | | | | 0.87 | | | 308 | | | 3.78 | | | | 0.87 | | | | | 71 | | | 2021 report Table 2, pooled all sport groups | | | | | | | | | | |  |  |  |
| Hangai 2009 & Owen 2021 | | IVD nucleus-annulus signal intensity ratio | | L2-L3 | | 4.49 | | | | | | 0.96 | | | 308 | | | 4.40 | | | | 0.98 | | | | | 71 | | | 2021 report Table 2, pooled all sport groups | | | | | | | | | | |  |  |  |
| Hangai 2009 & Owen 2021 | | IVD nucleus-annulus signal intensity ratio | | L3-L4 | | 4.93 | | | | | | 1.04 | | | 308 | | | 4.96 | | | | 1.09 | | | | | 71 | | | 2021 report Table 2, pooled all sport groups | | | | | | | | | | |  |  |  |
| Hangai 2009 & Owen 2021 | | IVD nucleus-annulus signal intensity ratio | | L4-L5 | | 4.19 | | | | | | 1.27 | | | 308 | | | 4.34 | | | | 1.33 | | | | | 71 | | | 2021 report Table 2, pooled all sport groups | | | | | | | | | | |  |  |  |
| Hangai 2009 & Owen 2021 | | IVD nucleus-annulus signal intensity ratio | | L5-S1 | | 3.40 | | | | | | 1.12 | | | 308 | | | 3.47 | | | | 1.16 | | | | | 71 | | | 2021 report Table 2, pooled all sport groups | | | | | | | | | | |  |  |  |
| Hangai 2009 & Owen 2021 | | IVD width | | L1-L2 | | 26.84 | | | | | | 3.32 | | | 308 | | | 25.70 | | | | 3.30 | | | | | 71 | | | 2021 report Table 1, pooled all sport groups | | | | | | | | | | |  |  |  |
| Hangai 2009 & Owen 2021 | | IVD width | | L2-L3 | | 28.63 | | | | | | 3.36 | | | 308 | | | 27.70 | | | | 3.90 | | | | | 71 | | | 2021 report Table 1, pooled all sport groups | | | | | | | | | | |  |  |  |
| Hangai 2009 & Owen 2021 | | IVD width | | L3-L4 | | 29.24 | | | | | | 3.27 | | | 308 | | | 28.70 | | | | 3.40 | | | | | 71 | | | 2021 report Table 1, pooled all sport groups | | | | | | | | | | |  |  |  |
| Hangai 2009 & Owen 2021 | | IVD width | | L4-L5 | | 28.75 | | | | | | 3.47 | | | 308 | | | 27.80 | | | | 3.30 | | | | | 71 | | | 2021 report Table 1, pooled all sport groups | | | | | | | | | | |  |  |  |
| Hangai 2009 & Owen 2021 | | IVD width | | L5-S1 | | 23.90 | | | | | | 3.33 | | | 308 | | | 22.80 | | | | 3.00 | | | | | 71 | | | 2021 report Table 1, pooled all sport groups | | | | | | | | | | |  |  |  |
| **Hangai 2009 & Owen 2021** | | **Synthetic effect size: ρ \| Hedges’ g \| standard error** | | | | | | | | | | | | | | | | | | | | | | | | | | | | | | | | | | | | | | |  |  |  |
|  | | ρ=0.0 \| 0.21 \| 0.03 | | | | | | | | | | | | | | | | | | | | | | | | | | | |  | | | |  | | | | | | |  |  |  |
|  | | ρ=0.2 \| 0.21 \| 0.03 | |  | |  | | | | | |  | | |  | | |  | | | |  | | | | |  | | |  | | | | | | | | | | |  |  |  |
|  | | ρ=0.4 \| 0.21 \| 0.03 | |  | |  | | | | | |  | | |  | | |  | | | |  | | | | |  | | |  | | | | | | | | | | |  |  |  |
|  | | ρ=0.6 \| 0.21 \| 0.03 | |  | |  | | | | | |  | | |  | | |  | | | |  | | | | |  | | |  | | | | | | | | | | |  |  |  |
|  | | ρ=0.8 \| 0.21 \| 0.03 | |  | |  | | | | | |  | | |  | | |  | | | |  | | | | |  | | |  | | | | | | | | | | |  |  |  |
|  | | ρ=1.0 \| 0.21 \| 0.03 | |  | |  | | | | | |  | | |  | | |  | | | |  | | | | |  | | |  | | | | | | | | | | |  |  |  |
| Teichtahl 2015 | | IVD height average | | L5-S1 | | 11 | | | | | | 15.79 | | | 57 | | | 10.2 | | | | 15.88 | | | | | 15 | | | Imputed SD from SE in Table 1, pooled moderately active and active groups | | | | | | | | | | |  |  |  |
| Vadala 2014 | | IVD T1p of nucleus pulposus | | L1-L2 | | 102.53 | | | | | | 26.91 | | | 13 | | | 116.1 | | | | 36.54 | | | | | 13 | | | Imputed from median IQR in Table 1 | | | | | | | | | | |  |  |  |
| Vadala 2014 | | IVD T1p of nucleus pulposus | | L2-L3 | | 99.65 | | | | | | 17.77 | | | 13 | | | 114.9 | | | | 22.59 | | | | | 13 | | | Imputed from median IQR in Table 1 | | | | | | | | | | |  |  |  |
| Vadala 2014 | | IVD T1p of nucleus pulposus | | L3-L4 | | 94.18 | | | | | | 16.53 | | | 13 | | | 112.2 | | | | 28.16 | | | | | 13 | | | Imputed from median IQR in Table 1 | | | | | | | | | | |  |  |  |
| Vadala 2014 | | IVD T1p of nucleus pulposus | | L4-L5 | | 77.48 | | | | | | 20.51 | | | 13 | | | 110.7 | | | | 21.59 | | | | | 13 | | | Imputed from median IQR in Table 1 | | | | | | | | | | |  |  |  |
| Vadala 2014 | | IVD T1p of nucleus pulposus | | L5-S1 | | 79.78 | | | | | | 21.76 | | | 13 | | | 100.7 | | | | 24.92 | | | | | 13 | | | Imputed from median IQR in Table 1 | | | | | | | | | | |  |  |  |
| **Vadala 2014** | | **Synthetic effect size: ρ \| Hedges’ g \| standard error** | | | | | | | | | | | | | | | | | | | | | | | | | | | | | | | | | | | | | | |  |  |  |
|  | | ρ=0.0 \| -0.86 \| 0.19 | | | | | | | | | | | | | | | | | | | | | | | | | | | |  | | | |  | | | | | | |  |  |  |
|  | | ρ=0.2 \| -0.86 \| 0.19 | |  | |  | | | | | |  | | |  | | |  | | | |  | | | | |  | | |  | | | | | | | | | | |  |  |  |
|  | | ρ=0.4 \| -0.86 \| 0.19 | |  | |  | | | | | |  | | |  | | |  | | | |  | | | | |  | | |  | | | | | | | | | | |  |  |  |
|  | | ρ=0.6 \| -0.86 \| 0.19 | |  | |  | | | | | |  | | |  | | |  | | | |  | | | | |  | | |  | | | | | | | | | | |  |  |  |
|  | | ρ=0.8 \| -0.86 \| 0.19 | |  | |  | | | | | |  | | |  | | |  | | | |  | | | | |  | | |  | | | | | | | | | | |  |  |  |
|  | | ρ=1.0 \| -0.86 \| 0.19 | |  | |  | | | | | |  | | |  | | |  | | | |  | | | | |  | | |  | | | | | | | | | | |  |  |  |
| Zhang 2023 | | Nucleus pulposus T2 time, Average lumbar | | LxAx | | 110.7 | | | | | | 24.92 | | | *27* | | | 98.3 | | | | 22.2 | | | | | 18 | | | Imputed from Figure 4 (WebPlotDigitizer) | | | | | | | | | | |  |  |  |
| **Primary synthesis: combined physical loading, RCT/cohort studies with sperate control. Mean change from control and intervention groups at follow-up time point.** | | | | | | | | | | | | | | | | | | | | | | | | | | | | | | | | | | | | | | | | |  |  |  |
| Horga 2022 | | IVD height | | L3-L4 | | -0.2 | | | | | | 1 | | | 21 | | | 0 | | | | 1 | | | | | 4 | | | Table 3. Imputed SD from assumed variance for IVD height | | | | | | | | | | |  |  |  |
| Horga 2022 | | IVD height | | L4-L5 | | -0.09 | | | | | | 1 | | | 21 | | | 0 | | | | 1 | | | | | 4 | | | Table 3. Imputed SD from assumed variance for IVD height | | | | | | | | | | |  |  |  |
| Horga 2022 | | IVD height | | L5-S1 | | 0.04 | | | | | | 1 | | | 21 | | | 0 | | | | 1 | | | | | 4 | | | Table 3. Imputed SD from assumed variance for IVD height | | | | | | | | | | |  |  |  |
| Horga 2022 | | IVD height | | L3-L4 | | -0.18 | | | | | | 3.31 | | | 21 | | | 0 | | | | 3.31 | | | | | 4 | | | Table 3. Imputed SD from assumed variance for IVD height | | | | | | | | | | |  |  |  |
| Horga 2022 | | IVD width | | L4-L5 | | -0.16 | | | | | | 3.31 | | | 21 | | | 0 | | | | 3.31 | | | | | 4 | | | Table 3. Imputed SD from assumed variance for IVD width | | | | | | | | | | |  |  |  |
| Horga 2022 | | IVD width | | L5-S1 | | -0.05 | | | | | | 3.31 | | | 21 | | | 0 | | | | 3.31 | | | | | 4 | | | Table 3. Imputed SD from assumed variance for IVD width | | | | | | | | | | |  |  |  |
| Horga 2022 | | IVD distance | | L4 | | -0.12 | | | | | | 2.38 | | | 21 | | | 0 | | | | 2.38 | | | | | 4 | | | Table 3. Imputed SD from assumed variance for IVD width | | | | | | | | | | |  |  |  |
| Horga 2022 | | IVD distance | | L5 | | -0.09 | | | | | | 2.38 | | | 21 | | | 0 | | | | 2.38 | | | | | 4 | | | Table 3. Imputed SD from assumed variance for IVD width | | | | | | | | | | |  |  |  |
| Horga 2022 | | IVD distance | | S1 | | 0.13 | | | | | | 2.38 | | | 21 | | | 0 | | | | 2.38 | | | | | 4 | | | Table 3. Imputed SD from assumed variance for IVD width | | | | | | | | | | |  |  |  |
| **Horga 2022** | | **Synthetic effect size: ρ \| Hedges’ g \| standard error** | | | | | | | | | | | | | | | | | | | | | | | | | | | | | | | | | | | | | | |  |  |  |
|  | | ρ=0.0 \| -0.04 \| 0.18 | | | | | | | | | | | | | | | | | | | | | | | | | | | |  | | | |  | | | | | | |  |  |  |
|  | | ρ=0.2 \| -0.04 \| 0.18 | |  | |  | | | | | |  | | |  | | |  | | | |  | | | | |  | | |  | | | | | | | | | | |  |  |  |
|  | | ρ=0.4 \| -0.04 \| 0.18 | |  | |  | | | | | |  | | |  | | |  | | | |  | | | | |  | | |  | | | | | | | | | | |  |  |  |
|  | | ρ=0.6 \| -0.04 \| 0.18 | |  | |  | | | | | |  | | |  | | |  | | | |  | | | | |  | | |  | | | | | | | | | | |  |  |  |
|  | | ρ=0.8 \| -0.04 \| 0.18 | |  | |  | | | | | |  | | |  | | |  | | | |  | | | | |  | | |  | | | | | | | | | | |  |  |  |
|  | | ρ=1.0 \| -0.04 \| 0.18 | |  | |  | | | | | |  | | |  | | |  | | | |  | | | | |  | | |  | | | | | | | | | | |  |  |  |
| Owen 2020 | | Whole IVD T2-time, average lumbar spine | | LxAx | | -1.7 | | | | | | 4.7 | | | 17 | | | -0.3 | | | | 5.4 | | | | | 15 | | | Table 2 | | | | | | | | | | |  |  |  |
| Owen 2020 | | IVD apparent diffusion coeffcient | | L1-L2 | | -8.7 | | | | | | 94.8 | | | 17 | | | 20.4 | | | | 126.5 | | | | | 15 | | | Table 3 | | | | | | | | | | |  |  |  |
| Owen 2020 | | IVD apparent diffusion coeffcient | | L2-L3 | | -17.9 | | | | | | 94.8 | | | 17 | | | 9.8 | | | | 110.3 | | | | | 15 | | | Table 3 | | | | | | | | | | |  |  |  |
| Owen 2020 | | IVD apparent diffusion coeffcient | | L3-L4 | | -41.8 | | | | | | 102.9 | | | 17 | | | -37.1 | | | | 117.5 | | | | | 15 | | | Table 3 | | | | | | | | | | |  |  |  |
| Owen 2020 | | IVD apparent diffusion coeffcient | | L4-L5 | | -6.2 | | | | | | 149.6 | | | 17 | | | -1.2 | | | | 147.4 | | | | | 15 | | | Table 3 | | | | | | | | | | |  |  |  |
| Owen 2020 | | IVD apparent diffusion coeffcient | | L5-S1 | | -19.3 | | | | | | 121.2 | | | 17 | | | -29.6 | | | | 101.8 | | | | | 15 | | | Table 3 | | | | | | | | | | |  |  |  |
| Owen 2020 | | IVD volume | | LxAx | | 0 | | | | | | 0.6 | | | 17 | | | 0.3 | | | | 0.6 | | | | | 15 | | | Table 4 | | | | | | | | | | |  |  |  |
| Owen 2020 | | IVD height | | LxAx | | 0 | | | | | | 0.2 | | | 17 | | | 0.1 | | | | 0.2 | | | | | 15 | | | Table 4 | | | | | | | | | | |  |  |  |
| **Owen 2020** | | **Synthetic effect size: ρ \| Hedges’ g \| standard error** | | | | | | | | | | | | | | | | | | | | | | | | | | | | | | | | | | | | | | |  |  |  |
|  | | ρ=0.0 \| -0.22 \| 0.13 | | | | | | | | | | | | | | | | | | | |  | | | | | | | | | | | | |  | | | | | |  |  |  |
|  | | ρ=0.2 \| -0.22 \| 0.13 | |  | |  | | | | | |  | | |  | | |  | | | |  | | | | |  | | |  | | | | | | | | | | |  |  |  |
|  | | ρ=0.4 \| -0.22 \| 0.13 | |  | |  | | | | | |  | | |  | | |  | | | |  | | | | |  | | |  | | | | | | | | | | |  |  |  |
|  | | ρ=0.6 \| -0.22 \| 0.13 | |  | |  | | | | | |  | | |  | | |  | | | |  | | | | |  | | |  | | | | | | | | | | |  |  |  |
|  | | ρ=0.8 \| -0.22 \| 0.13 | |  | |  | | | | | |  | | |  | | |  | | | |  | | | | |  | | |  | | | | | | | | | | |  |  |  |
|  | | ρ=1.0 \| -0.22 \| 0.13 | |  | |  | | | | | |  | | |  | | |  | | | |  | | | | |  | | |  | | | | | | | | | | |  |  |  |
| **Primary synthesis: combined physical loading, RCT/cohort studies with baseline as control. Mean change from baseline to follow-up.** | | | | | | | | | | | | | | | | | | | | | | | | | | | | | | | | | | | | | | | | |  |  |  |
| Frenken 2022 | | IVD glycosaminoglycan content (%), average lumbar spine | | LxAx | | 2.58 | | | | | | 0.27 | | | 17 | | | 2.11 | | | | 0.18 | | | | | 17 | | | Results section (pg.3) | | | | | | | | | | |  |  |  |
| Horga 2022 | | IVD height | | L3-L4 | | 10.86 | | | | | | 1 | | | 28 | | | 10.41 | | | | 1 | | | | | 21 | | | Table 3. Imputed SD from assumed variance for IVD height | | | | | | | | | | |  |  |  |
| Horga 2022 | | IVD height | | L4-L5 | | 11.26 | | | | | | 1 | | | 28 | | | 11.17 | | | | 1 | | | | | 21 | | | Table 3. Imputed SD from assumed variance for IVD height | | | | | | | | | | |  |  |  |
| Horga 2022 | | IVD height | | L5-S1 | | 9.56 | | | | | | 1 | | | 28 | | | 9.6 | | | | 1 | | | | | 21 | | | Table 3. Imputed SD from assumed variance for IVD height | | | | | | | | | | |  |  |  |
| Horga 2022 | | IVD height | | L3-L4 | | 33.61 | | | | | | 3.31 | | | 28 | | | 33.43 | | | | 3.31 | | | | | 21 | | | Table 3. Imputed SD from assumed variance for IVD height | | | | | | | | | | |  |  |  |
| Horga 2022 | | IVD width | | L4-L5 | | 33.67 | | | | | | 3.31 | | | 28 | | | 33.51 | | | | 3.31 | | | | | 21 | | | Table 3. Imputed SD from assumed variance for IVD width | | | | | | | | | | |  |  |  |
| Horga 2022 | | IVD width | | L5-S1 | | 32.64 | | | | | | 3.31 | | | 28 | | | 32.59 | | | | 3.31 | | | | | 21 | | | Table 3. Imputed SD from assumed variance for IVD width | | | | | | | | | | |  |  |  |
| Horga 2022 | | IVD distance | | L4 | | 26.98 | | | | | | 2.38 | | | 28 | | | 26.86 | | | | 2.38 | | | | | 21 | | | Table 3. Imputed SD from assumed variance for IVD distance | | | | | | | | | | |  |  |  |
| Horga 2022 | | IVD distance | | L5 | | 24.63 | | | | | | 2.38 | | | 28 | | | 24.54 | | | | 2.38 | | | | | 21 | | | Table 3. Imputed SD from assumed variance for IVD distance | | | | | | | | | | |  |  |  |
| Horga 2022 | | IVD distance | | S1 | | 24.04 | | | | | | 2.38 | | | 28 | | | 24.17 | | | | 2.38 | | | | | 21 | | | Table 3. Imputed SD from assumed variance for IVD distance | | | | | | | | | | |  |  |  |
| **Horga 2022** | | **Synthetic effect size: ρ \| Hedges’ g \| standard error** | | | | | | | | | | | | | | | | | | | | | | | | | | | | | | | | | | | | | | |  |  |  |
|  | | ρ=0.0 \| 0.07 \| 0.10 | | | | | | | | | | | | | | | | | | | |  | | | | | | | | | | | | |  | | | | | |  |  |  |
|  | | ρ=0.2 \| 0.07 \| 0.10 | |  | |  | | | | | |  | | |  | | |  | | | |  | | | | |  | | |  | | | | | | | | | | |  |  |  |
|  | | ρ=0.4 \| 0.07 \| 0.10 | |  | |  | | | | | |  | | |  | | |  | | | |  | | | | |  | | |  | | | | | | | | | | |  |  |  |
|  | | ρ=0.6 \| 0.07 \| 0.10 | |  | |  | | | | | |  | | |  | | |  | | | |  | | | | |  | | |  | | | | | | | | | | |  |  |  |
|  | | ρ=0.8 \| 0.07 \| 0.10 | |  | |  | | | | | |  | | |  | | |  | | | |  | | | | |  | | |  | | | | | | | | | | |  |  |  |
|  | | ρ=1.0 \| 0.07 \| 0.10 | |  | |  | | | | | |  | | |  | | |  | | | |  | | | | |  | | |  | | | | | | | | | | |  |  |  |
| Owen 2020 | | Whole IVD T2-time, average lumbar spine | | LxAx | | 94.1 | | | | | | 4.7 | | | 17 | | | 95.8 | | | | 11.3 | | | | | 20 | | | Table 2 | | | | | | | | | | |  |  |  |
| Owen 2020 | | IVD apparent diffusion coeffcient | | L1-L2 | | 810.9 | | | | | | 94.8 | | | 17 | | | 819.6 | | | | 131.3 | | | | | 20 | | | Table 3 | | | | | | | | | | |  |  |  |
| Owen 2020 | | IVD apparent diffusion coeffcient | | L2-L3 | | 789.4 | | | | | | 94.8 | | | 17 | | | 807.3 | | | | 102.3 | | | | | 20 | | | Table 3 | | | | | | | | | | |  |  |  |
| Owen 2020 | | IVD apparent diffusion coeffcient | | L3-L4 | | 797.9 | | | | | | 102.9 | | | 17 | | | 807.3 | | | | 102.3 | | | | | 20 | | | Table 3 | | | | | | | | | | |  |  |  |
| Owen 2020 | | IVD apparent diffusion coeffcient | | L4-L5 | | 743.6 | | | | | | 149.6 | | | 17 | | | 749.8 | | | | 171.5 | | | | | 20 | | | Table 3 | | | | | | | | | | |  |  |  |
| Owen 2020 | | IVD apparent diffusion coeffcient | | L5-S1 | | 605.6 | | | | | | 121.2 | | | 17 | | | 624.9 | | | | 162.6 | | | | | 20 | | | Table 3 | | | | | | | | | | |  |  |  |
| Owen 2020 | | IVD volume | | LxAx | | 8.6 | | | | | | 0.6 | | | 17 | | | 8.6 | | | | 1.9 | | | | | 20 | | | Table 4 | | | | | | | | | | |  |  |  |
| Owen 2020 | | IVD height | | LxAx | | 8.1 | | | | | | 0.2 | | | 17 | | | 8.1 | | | | 0.8 | | | | | 20 | | | Table 4 | | | | | | | | | | |  |  |  |
| **Owen 2020** | | **Synthetic effect size: ρ \| Hedges’ g \| standard error** | | | | | | | | | | | | | | | | | | | | | | | | | | | | | | | | | | | | | | |  |  |  |
|  | | ρ=0.0 \| -0.09 \| 0.12 | | | | | | | | | | | | | | | |  | | | | | | | | | | | | | | | | | | | | | | |  |  |  |
|  | | ρ=0.2 \| -0.09 \| 0.12 | |  | |  | | | | | |  | | |  | | |  | | | |  | | | | |  | | |  | | | | | | | | | | |  |  |  |
|  | | ρ=0.4 \| -0.09 \| 0.12 | |  | |  | | | | | |  | | |  | | |  | | | |  | | | | |  | | |  | | | | | | | | | | |  |  |  |
|  | | ρ=0.6 \| -0.09 \| 0.12 | |  | |  | | | | | |  | | |  | | |  | | | |  | | | | |  | | |  | | | | | | | | | | |  |  |  |
|  | | ρ=0.8 \| -0.09 \| 0.12 | |  | |  | | | | | |  | | |  | | |  | | | |  | | | | |  | | |  | | | | | | | | | | |  |  |  |
|  | | ρ=1.0 \| -0.09 \| 0.12 | |  | |  | | | | | |  | | |  | | |  | | | |  | | | | |  | | |  | | | | | | | | | | |  |  |  |
| Lagerstrand 2021 & Rosenqvist 2023, high load | | IVD signal intensity | | LxAx | | 0.22 | | | | | | 0.23 | | | 10 | | | 0.23 | | | | 0.2 | | | | | 10 | | | 2023 report: High load data imputed from Figure 2 (WebPlotDigitizer) | | | | | | | | | | |  |  |  |
| Lagerstrand 2021 & Rosenqvist 2023, low load | | IVD signal intensity | | LxAx | | 0.22 | | | | | | 0.14 | | | 7 | | | 0.25 | | | | 0.18 | | | | | 7 | | | 2023 report: Low load data imputed from Figure 2 (WebPlotDigitizer) | | | | | | | | | | |  |  |  |
| **Primary synthesis: Upright bipedal, cross-sectional** | | | | | | | | | | | | | | | | | | | | | | | | | | | | | | | | | | | | | | | | |  |  |  |
| Belavy 2017 & 2020 | | IVD height to vertebral body height ratio | | L2-L3 | | 0.37 | | | | | | 0.07 | | | 55 | | | 0.36 | | | | 0.06 | | | | | 84 | | | 2017 report: Imputed from Figure 2 (WebPlotDigitizer), pooled 20-40km and 50km running groups | | | | | | | | | | |  |  |  |
| Belavy 2017 & 2020 | | IVD height to vertebral body height ratio | | L3-L4 | | 0.33 | | | | | | 0.09 | | | 55 | | | 0.31 | | | | 0.05 | | | | | 24 | | | 2017 report: Imputed from Figure 2 (WebPlotDigitizer), pooled 20-40km and 50km running groups | | | | | | | | | | |  |  |  |
| Belavy 2017 & 2020 | | IVD height to vertebral body height ratio | | L4-L5 | | 0.22 | | | | | | 0.05 | | | 55 | | | 0.21 | | | | 0.04 | | | | | 24 | | | 2017 report: Imputed from Figure 2 (WebPlotDigitizer), pooled20 40km and 50km running groups | | | | | | | | | | |  |  |  |
| Belavy 2017 & 2020 | | IVD height to vertebral body height ratio | | L5-S1 | | 0.23 | | | | | | 0.05 | | | 55 | | | 0.21 | | | | 0.03 | | | | | 24 | | | 2017 report: Imputed from Figure 2 (WebPlotDigitizer), pooled 20-40km and 50km running groups | | | | | | | | | | |  |  |  |
| Belavy 2017 & 2020 | | IVD T2- time | | L1-L2 | | 118.52 | | | | | | 19.82 | | | 55 | | | 107.62 | | | | 6.62 | | | | | 24 | | | 2017 report: Imputed from Figure 2 (WebPlotDigitizer), pooled 20-40km and 50km running groups | | | | | | | | | | |  |  |  |
| Belavy 2017 & 2020 | | IVD T2- time | | L2-L3 | | 118.32 | | | | | | 16.93 | | | 55 | | | 108.82 | | | | 11.49 | | | | | 24 | | | 2017 repot: Imputed from Figure 2 (WebPlotDigitizer), pooled 20-40km and 50km running groups | | | | | | | | | | |  |  |  |
| Belavy 2017 & 2020 | | IVD T2- time | | L3-L4 | | 116.44 | | | | | | 16.74 | | | 55 | | | 108.27 | | | | 12.61 | | | | | 24 | | | 2017 repot: Imputed from Figure 2 (WebPlotDigitizer), pooled 20-40km and 50km running groups | | | | | | | | | | |  |  |  |
| Belavy 2017 & 2020 | | IVD T2- time | | L4-L5 | | 110.54 | | | | | | 18.69 | | | 55 | | | 100.13 | | | | 16.97 | | | | | 24 | | | 2017 repot: Imputed from Figure 2 (WebPlotDigitizer), pooled 20-40km and 50km running groups | | | | | | | | | | |  |  |  |
| Belavy 2017 & 2020 | | IVD T2- time | | L5-S1 | | 108.05 | | | | | | 19.40 | | | 55 | | | 94.98 | | | | 20.71 | | | | | 24 | | | 2017 report: Imputed from Figure 2 (WebPlotDigitizer), pooled 20-40km and 50km running groups | | | | | | | | | | |  |  |  |
| Belavy 2017 & 2020 | | IVD T2-time nucleus/annulus ratio | | LxAx | | 1.49 | | | | | | 0.13 | | | 55 | | | 1.4 | | | | 0.15 | | | | | 24 | | | 2019: Accessed n from author, imputed SD from SE from Table 1, pooled 20-40km and 50km running groups | | | | | | | | | | |  |  |  |
| Belavy 2017 & 2020 | | IVD T2 weight signal intensity | | LxAx | | 318.92 | | | | | | 23.88 | | | 55 | | | 326.9 | | | | 24.00 | | | | | 24 | | | 2019: Accessed n from author, imputed SD from SE from Table 1, pooled 20-40km and 50km running groups | | | | | | | | | | |  |  |  |
| Belavy 2017 & 2020 | | IVD T2 weight signal intensity nucleus/annulus ratio | | LxAx | | 3.37 | | | | | | 0.35 | | | 55 | | | 3.31 | | | | 0.34 | | | | | 24 | | | 2019: Accessed n from author, imputed SD from SE from Table 1, pooled 20-40km and 50km running groups | | | | | | | | | | |  |  |  |
| Belavy 2017 & 2020 | | IVD Dixon water fraction | | LxAx | | 87.55 | | | | | | 3.17 | | | 55 | | | 86 | | | | 2.94 | | | | | 24 | | | 2019: Accessed n from author, imputed SD from SE from Table 1, pooled 20-40km and 50km running groups | | | | | | | | | | |  |  |  |
| Belavy 2017 & 2020 | | IVD Dixon water fraction nucleus/annulus ratio | | LxAx | | 1.01 | | | | | | 0.01 | | | 55 | | | 1.01 | | | | 0.01 | | | | | 24 | | | 2019: Accessed n from author, imputed SD from SE from Table 1, pooled 20-40km and 50km running groups | | | | | | | | | | |  |  |  |
| **Belavy 2017 & 2020** | | **Synthetic effect size: ρ \| Hedges’ g \| standard error** | | | | | | | | | | | | | | | | | | | | | | | | | | | | | | | | | | | | | | |  |  |  |
|  | | ρ=0.0 \| 0.29 \| 0.07 | | | | | | |  | | | | | | | | | | | | | | | | | | | | |  | | | | | | | | | |  |  |  |  |
|  | | ρ=0.2 \| 0.29 \| 0.07 | |  | |  | | | | | |  | | |  | | |  | | | |  | | | | |  | | |  | | | | | | | | | | |  |  |  |
|  | | ρ=0.4 \| 0.29 \| 0.07 | |  | |  | | | | | |  | | |  | | |  | | | |  | | | | |  | | |  | | | | | | | | | | |  |  |  |
|  | | ρ=0.6 \| 0.29 \| 0.07 | |  | |  | | | | | |  | | |  | | |  | | | |  | | | | |  | | |  | | | | | | | | | | |  |  |  |
|  | | ρ=0.8 \| 0.29 \| 0.07 | |  | |  | | | | | |  | | |  | | |  | | | |  | | | | |  | | |  | | | | | | | | | | |  |  |  |
|  | | ρ=1.0 \| 0.29 \| 0.07 | |  | |  | | | | | |  | | |  | | |  | | | |  | | | | |  | | |  | | | | | | | | | | |  |  |  |
| Bowden 2018 | | IVD Fractional anisotropy | | LxAx | | 0.15 | | | | | | 0.06 | | | 14 | | | 0.17 | | | | 0.11 | | | | | 12 | | | Table 3 | | | | | | | | | | |  |  |  |
| Bowden 2018 | | IVD apparent diffusion coefficient | | LxAx | | 1.15 | | | | | | 0.48 | | | 14 | | | 0.79 | | | | 0.49 | | | | | 12 | | | Table 3 | | | | | | | | | | |  |  |  |
| Bowden 2018 | | IVD T2 signal intensity | | LxAx | | 55.8 | | | | | | 24.7 | | | 14 | | | 49.7 | | | | 21.4 | | | | | 12 | | | Table 3 | | | | | | | | | | |  |  |  |
| **Bowden 2018** | | **Synthetic effect size: ρ \| Hedges’ g \| standard error** | | | | | | | | | | | | | | | | | | | | | | | | | | | | | | | | | | | | | | |  |  |  |
|  | | ρ=0.0 \| 0.40 \| 0.23 | | | | | | | | | | | | | | | | |  | | | | | | | | | | | | | | | | | | | | | |  |  |  |
|  | | ρ=0.2 \| 0.40 \| 0.24 | |  | |  | | | | | |  | | |  | | |  | | | |  | | | | |  | | |  | | | | | | | | | | |  |  |  |
|  | | ρ=0.4 \| 0.40 \| 0.24 | |  | |  | | | | | |  | | |  | | |  | | | |  | | | | |  | | |  | | | | | | | | | | |  |  |  |
|  | | ρ=0.6 \| 0.40 \| 0.25 | |  | |  | | | | | |  | | |  | | |  | | | |  | | | | |  | | |  | | | | | | | | | | |  |  |  |
|  | | ρ=0.8 \| 0.40 \| 0.25 | |  | |  | | | | | |  | | |  | | |  | | | |  | | | | |  | | |  | | | | | | | | | | |  |  |  |
|  | | ρ=1.0 \| 0.40 \| 0.26 | |  | |  | | | | | |  | | |  | | |  | | | |  | | | | |  | | |  | | | | | | | | | | |  |  |  |
| Lagerstrand 2021 & Rosenqvist 2023 | | IVD T2 value subregion 1 | | Lx/Tx Ax | | 0.74 | | | | | | 0.33 | | | 58 | | | 0.76 | | | | 0.34 | | | | | 26 | | | 2021 report: Imputed from figure 3 (WebPlotDigitizer) | | | | | | | | | | |  |  |  |
| Lagerstrand 2021 & Rosenqvist 2023 | | IVD T2 value subregion 2 | | Lx/Tx Ax | | 1.06 | | | | | | 0.35 | | | 58 | | | 1.14 | | | | 0.4 | | | | | 26 | | | 2021 report: Imputed from figure 3 (WebPlotDigitizer) | | | | | | | | | | |  |  |  |
| Lagerstrand 2021 & Rosenqvist 2023 | | IVD T2 value subregion 3 | | Lx/Tx Ax | | 1.3 | | | | | | 0.48 | | | 58 | | | 1.33 | | | | 0.43 | | | | | 26 | | | 2021 report: Imputed from figure 3 (WebPlotDigitizer) | | | | | | | | | | |  |  |  |
| Lagerstrand 2021 & Rosenqvist 2023 | | IVD T2 value subregion 4 | | Lx/Tx Ax | | 1.13 | | | | | | 0.44 | | | 58 | | | 1.12 | | | | 0.41 | | | | | 26 | | | 2021 report: Imputed from figure 3 (WebPlotDigitizer) | | | | | | | | | | |  |  |  |
| Lagerstrand 2021 & Rosenqvist 2023 | | IVD T2 value subregion 5 | | Lx/Tx Ax | | 0.53 | | | | | | 0.28 | | | 58 | | | 0.47 | | | | 0.23 | | | | | 26 | | | 2021 report: Imputed from figure 3 (WebPlotDigitizer) | | | | | | | | | | |  |  |  |
| **Lagerstrand 2021 & Rosenqvist 2023** | | **Synthetic effect size: ρ \| Hedges’ g \| standard error** | | | | | | | | | | | | | | | | | | | | | | | | | | | | | | | | | | | | | | |  |  |  |
|  | | ρ=0.0 \| -0.02 \| 0.11 | | | | | | | | | | | | | | | | | | | | |  | | | | | | | | | | | | |  | | |  | |  |  |  |
|  | | ρ=0.2 \| -0.02 \| 0.11 | |  | |  | | | | | |  | | |  | | |  | | | |  | | | | |  | | |  | | | | | | | | | | |  |  |  |
|  | | ρ=0.4 \| -0.02 \| 0.11 | |  | |  | | | | | |  | | |  | | |  | | | |  | | | | |  | | |  | | | | | | | | | | |  |  |  |
|  | | ρ=0.6 \| -0.02 \| 0.11 | |  | |  | | | | | |  | | |  | | |  | | | |  | | | | |  | | |  | | | | | | | | | | |  |  |  |
|  | | ρ=0.8 \| -0.02 \| 0.11 | |  | |  | | | | | |  | | |  | | |  | | | |  | | | | |  | | |  | | | | | | | | | | |  |  |  |
|  | | ρ=1.0 \| -0.02 \| 0.11 | |  | |  | | | | | |  | | |  | | |  | | | |  | | | | |  | | |  | | | | | | | | | | |  |  |  |
| Li 2024 | | IVD T2 average annulus | | L1-L2 | | 21.54 | | | | | | 4.47 | | | 54 | | | 19.42 | | | | 3.68 | | | | | 30 | | | Imputed from Figure 4A (WebPlotDigitizer), | | | | | | | | | | |  |  |  |
| Li 2024 | | IVD T2 average annulus | | L2-L3 | | 22.5 | | | | | | 5.07 | | | 54 | | | 21.28 | | | | 5.09 | | | | | 30 | | | Imputed from Figure 4A (WebPlotDigitizer), | | | | | | | | | | |  |  |  |
| Li 2024 | | IVD T2 average annulus | | L3-L4 | | 21.99 | | | | | | 4.72 | | | 54 | | | 19.35 | | | | 3.47 | | | | | 30 | | | Imputed from Figure 4A (WebPlotDigitizer), | | | | | | | | | | |  |  |  |
| Li 2024 | | IVD T2 average annulus | | L4-L5 | | 23.3 | | | | | | 5.3 | | | 54 | | | 21.68 | | | | 4.58 | | | | | 30 | | | Imputed from Figure 4A (WebPlotDigitizer), | | | | | | | | | | |  |  |  |
| Li 2024 | | IVD T2 average annulus | | L5-S1 | | 21.59 | | | | | | 5.55 | | | 54 | | | 19.85 | | | | 4.51 | | | | | 30 | | | Imputed from Figure 4A (WebPlotDigitizer), | | | | | | | | | | |  |  |  |
| Li 2024 | | IVD T2 average nucleus | | L1-L2 | | 42.23 | | | | | | 11.27 | | | 54 | | | 39.95 | | | | 10.72 | | | | | 30 | | | Imputed from Figure 4B (WebPlotDigitizer), | | | | | | | | | | |  |  |  |
| Li 2024 | | IVD T2 average nucleus | | L2-L3 | | 47.51 | | | | | | 7.74 | | | 54 | | | 44.71 | | | | 13.51 | | | | | 30 | | | Imputed from Figure 4B (WebPlotDigitizer), | | | | | | | | | | |  |  |  |
| Li 2024 | | IVD T2 average nucleus | | L3-L4 | | 49.34 | | | | | | 13.4 | | | 54 | | | 47.01 | | | | 15.79 | | | | | 30 | | | Imputed from Figure 4B (WebPlotDigitizer), | | | | | | | | | | |  |  |  |
| Li 2024 | | IVD T2 average nucleus | | L4-L5 | | 51.21 | | | | | | 15.94 | | | 54 | | | 43.3 | | | | 15.84 | | | | | 30 | | | Imputed from Figure 4B (WebPlotDigitizer), | | | | | | | | | | |  |  |  |
| Li 2024 | | IVD T2 average nucleus | | L5-S1 | | 47.66 | | | | | | 18.48 | | | 54 | | | 44.16 | | | | 17.41 | | | | | 30 | | | Imputed from Figure 4B (WebPlotDigitizer), | | | | | | | | | | |  |  |  |
| **Li 2024** | | **Synthetic effect size: ρ \| Hedges’ g \| standard error** | | | | | | | | | | | | | | | | | | | |  | | | | |  | | |  | | | | | | | | | | |  |  |  |
|  | | ρ=0.0 \| 0.33 \| 0.07 | |  | |  | | | | | |  | | |  | | |  | | | |  | | | | |  | | |  | | | | | | | | | | |  |  |  |
|  | | ρ=0.2 \| -0.33 \| 0.07 | |  | |  | | | | | |  | | |  | | |  | | | |  | | | | |  | | |  | | | | | | | | | | |  |  |  |
|  | | ρ=0.4 \| 0.33 \| 0.07 | |  | |  | | | | | |  | | |  | | |  | | | |  | | | | |  | | |  | | | | | | | | | | |  |  |  |
|  | | ρ=0.6 \| 0.33 \| 0.07 | |  | |  | | | | | |  | | |  | | |  | | | |  | | | | |  | | |  | | | | | | | | | | |  |  |  |
|  | | ρ=0.8 \| 0.33 \| 0.07 | |  | |  | | | | | |  | | |  | | |  | | | |  | | | | |  | | |  | | | | | | | | | | |  |  |  |
|  | | ρ=1.0 \| 0.33 \| 0.07 | |  | |  | | | | | |  | | |  | | |  | | | |  | | | | |  | | |  | | | | | | | | | | |  |  |  |
| Mitchell 2020 | | IVD height to vertebral body height ratio | | L2-L3 | | 0.33 | | | | | | 0.04 | | | 9 | | | 0.23 | | | | 0.02 | | | | | 8 | | | Table 3 | | | | | | | | | | |  |  |  |
| Mitchell 2020 | | IVD height to vertebral body height ratio | | L3-L4 | | 0.32 | | | | | | 0.05 | | | 9 | | | 0.25 | | | | 0.03 | | | | | 8 | | | Table 4 | | | | | | | | | | |  |  |  |
| Mitchell 2020 | | IVD height to vertebral body height ratio | | L4-L5 | | 0.31 | | | | | | 0.06 | | | 9 | | | 0.26 | | | | 0.04 | | | | | 8 | | | Table 4 | | | | | | | | | | |  |  |  |
| Mitchell 2020 | | IVD height to vertebral body height ratio | | L5-S1 | | 0.28 | | | | | | 0.05 | | | 9 | | | 0.22 | | | | 0.04 | | | | | 8 | | | Table 4 | | | | | | | | | | |  |  |  |
| Mitchell 2020 | | IVD T2- time average | | L2-L3 | | 101.2 | | | | | | 13.6 | | | 9 | | | 93.1 | | | | 26.6 | | | | | 8 | | | Table 4 | | | | | | | | | | |  |  |  |
| Mitchell 2020 | | IVD T2- time average | | L3-L4 | | 95.1 | | | | | | 13.9 | | | 9 | | | 89.6 | | | | 26.8 | | | | | 8 | | | Table 4 | | | | | | | | | | |  |  |  |
| Mitchell 2020 | | IVD T2- time average | | L4-L5 | | 83.9 | | | | | | 12.2 | | | 9 | | | 85.7 | | | | 32.7 | | | | | 8 | | | Table 4 | | | | | | | | | | |  |  |  |
| Mitchell 2020 | | IVD T2- time average | | L5-S1 | | 85.1 | | | | | | 14.8 | | | 9 | | | 78.8 | | | | 14.9 | | | | | 8 | | | Table 4 | | | | | | | | | | |  |  |  |
| Mitchell 2020 | | Apparent diffusion coefficient | | LxAx | | 249 | | | | | | 175.2 | | | 9 | | | 202.3 | | | | 149.5 | | | | | 8 | | | Table 4 | | | | | | | | | | |  |  |  |
| **Mitchell 2020** | | **Synthetic effect size: ρ \| Hedges’ g \| standard error** | | | | | | | | | | | | | | | | | | | | | | | | | | | | | | | | | | | | | | |  |  |  |
|  | | ρ=0.0 \| 0.88 \| 0.18 | | | | | | | | | | | | | | | | | | | | | | | | | | | | | | | | |  | | | | | |  |  |  |
|  | | ρ=0.2 \| 0.88 \| 0.18 | |  | |  | | | | | |  | | |  | | |  | | | |  | | | | |  | | |  | | | | | | | | | | |  |  |  |
|  | | ρ=0.4 \| 0.88 \| 0.18 | |  | |  | | | | | |  | | |  | | |  | | | |  | | | | |  | | |  | | | | | | | | | | |  |  |  |
|  | | ρ=0.6 \| 0.88 \| 0.18 | |  | |  | | | | | |  | | |  | | |  | | | |  | | | | |  | | |  | | | | | | | | | | |  |  |  |
|  | | ρ=0.8 \| 0.88 \| 0.18 | |  | |  | | | | | |  | | |  | | |  | | | |  | | | | |  | | |  | | | | | | | | | | |  |  |  |
|  | | ρ=1.0 \| 0.88 \| 0.18 | |  | |  | | | | | |  | | |  | | |  | | | |  | | | | |  | | |  | | | | | | | | | | |  |  |  |
| Hangai 2009 & Owen 2021 | | IVD width | | L1-L2 | | 26.78 | | | | | | 3.16 | | | 153 | | | 25.7 | | | | 3.3 | | | | | 71 | | | 2021 report Table 1, pooled running, basketball and soccer groups | | | | | | | | | | |  |  |  |
| Hangai 2009 & Owen 2021 | | IVD width | | L2-L3 | | 28.71 | | | | | | 3.19 | | | 153 | | | 27.7 | | | | 3.9 | | | | | 71 | | | 2021 report Table 1, pooled running, basketball and soccer groups | | | | | | | | | | |  |  |  |
| Hangai 2009 & Owen 2021 | | IVD width | | L3-L4 | | 29.44 | | | | | | 3.31 | | | 153 | | | 28.7 | | | | 3.4 | | | | | 71 | | | 2021 report Table 1, pooled running, basketball and soccer groups | | | | | | | | | | |  |  |  |
| Hangai 2009 & Owen 2021 | | IVD width | | L4-L5 | | 28.62 | | | | | | 3.61 | | | 153 | | | 27.8 | | | | 3.3 | | | | | 71 | | | 2021 report Table 1, pooled running, basketball and soccer groups | | | | | | | | | | |  |  |  |
| Hangai 2009 & Owen 2021 | | IVD width | | L5-S1 | | 23.46 | | | | | | 3.05 | | | 153 | | | 22.8 | | | | 3 | | | | | 71 | | | 2021 report Table 1, pooled running, basketball and soccer groups | | | | | | | | | | |  |  |  |
| Hangai 2009 & Owen 2021 | | IVD nucleus-annulus signal intensity ratio | | L1-L2 | | 4.06 | | | | | | 0.83 | | | 153 | | | 3.78 | | | | 0.87 | | | | | 71 | | | 2021 report Table 2, pooled running, basketball and soccer groups | | | | | | | | | | |  |  |  |
| Hangai 2009 & Owen 2021 | | IVD nucleus-annulus signal intensity ratio | | L2-L3 | | 4.57 | | | | | | 0.98 | | | 153 | | | 4.4 | | | | 0.98 | | | | | 71 | | | 2021 report Table 2, pooled running, basketball and soccer groups | | | | | | | | | | |  |  |  |
| Hangai 2009 & Owen 2021 | | IVD nucleus-annulus signal intensity ratio | | L3-L4 | | 5.05 | | | | | | 1.04 | | | 153 | | | 4.96 | | | | 1.09 | | | | | 71 | | | 2021 report Table 2, pooled running, basketball and soccer groups | | | | | | | | | | |  |  |  |
| Hangai 2009 & Owen 2021 | | IVD nucleus-annulus signal intensity ratio | | L4-L5 | | 4.3 | | | | | | 1.27 | | | 153 | | | 4.34 | | | | 1.33 | | | | | 71 | | | 2021 report Table 2, pooled running, basketball and soccer groups | | | | | | | | | | |  |  |  |
| Hangai 2009 & Owen 2021 | | IVD nucleus-annulus signal intensity ratio | | L5-S1 | | 3.59 | | | | | | 1.1 | | | 153 | | | 3.47 | | | | 1.16 | | | | | 71 | | | 2021 report Table 2, pooled running, basketball and soccer groups | | | | | | | | | | |  |  |  |
| Hangai 2009 & Owen 2021 | | IVD height to vertebral body height ratio | | L1-L2 | | 0.32 | | | | | | 0.04 | | | 153 | | | 0.31 | | | | 0.04 | | | | | 71 | | | 2021 report Table 2, pooled running, basketball and soccer groups | | | | | | | | | | |  |  |  |
| Hangai 2009 & Owen 2021 | | IVD height to vertebral body height ratio | | L2-L3 | | 0.37 | | | | | | 0.05 | | | 153 | | | 0.33 | | | | 0.05 | | | | | 71 | | | 2021 report Table 2, pooled running, basketball and soccer groups | | | | | | | | | | |  |  |  |
| Hangai 2009 & Owen 2021 | | IVD height to vertebral body height ratio | | L3-L4 | | 0.41 | | | | | | 0.05 | | | 153 | | | 0.38 | | | | 0.05 | | | | | 71 | | | 2021 report Table 2, pooled running, basketball and soccer groups | | | | | | | | | | |  |  |  |
| Hangai 2009 & Owen 2021 | | IVD height to vertebral body height ratio | | L4-L5 | | 0.46 | | | | | | 0.06 | | | 153 | | | 0.43 | | | | 0.07 | | | | | 71 | | | 2021 report Table 2, pooled running, basketball and soccer groups | | | | | | | | | | |  |  |  |
| Hangai 2009 & Owen 2021 | | IVD height to vertebral body height ratio | | L5-S1 | | 0.43 | | | | | | 0.07 | | | 153 | | | 0.43 | | | | 0.08 | | | | | 71 | | | 2021 report Table 2, pooled running, basketball and soccer groups | | | | | | | | | | |  |  |  |
| **Hangai 2009 & Owen 2021** | | **Synthetic effect size: ρ \| Hedges’ g \| standard error** | | | | | | | | | | | | | | | | | | | | | | | | | | | | | | | | | | | | | | |  |  |  |
|  | | ρ=0.0 \| 0.27 \| 0.04 | | | | | | | | | |  | | | | | | | | | | | | | | | | | | | | | |  | | | | | | |  |  |  |
|  | | ρ=0.2 \| 0.27 \| 0.04 | |  | |  | | | | | |  | | |  | | |  | | | |  | | | | |  | | |  | | | | | | | | | | |  |  |  |
|  | | ρ=0.4 \| 0.27 \| 0.04 | |  | |  | | | | | |  | | |  | | |  | | | |  | | | | |  | | |  | | | | | | | | | | |  |  |  |
|  | | ρ=0.6 \| 0.27 \| 0.04 | |  | |  | | | | | |  | | |  | | |  | | | |  | | | | |  | | |  | | | | | | | | | | |  |  |  |
|  | | ρ=0.8 \| 0.27 \| 0.04 | |  | |  | | | | | |  | | |  | | |  | | | |  | | | | |  | | |  | | | | | | | | | | |  |  |  |
|  | | ρ=1.0 \| 0.27 \| 0.04 | |  | |  | | | | | |  | | |  | | |  | | | |  | | | | |  | | |  | | | | | | | | | | |  |  |  |
| Teichtahl 2015 | | IVD height average | | L5-S1 | | 11 | | | | | | 15.79 | | | 57 | | | 10.2 | | | | 15.88 | | | | | 15 | | | Imputed SD from SE in Table 1, pooled moderately active and active groups | | | | | | | | | | |  |  |  |
| **Primary subgroup synthesis: Non-upright, non-contact, cross-sectional** | | | | | | | | | | | | | | | | | | | | | | | | | | | | | | | | | | | | | | | | |  |  |  |
| Belavy 2019 & 2020 | | IVD anteroposterior width | | LxAx | | 26.5 | | | | | | 2.2 | | | 18 | | | 25.7 | | | | 2.2 | | | | | 18 | | | 2019 report: Table 2 | | | | | | | | | | |  |  |  |
| Belavy 2019 & 2020 | | IVD volume | | LxAx | | 10.9 | | | | | | 2.5 | | | 18 | | | 9.6 | | | | 2.1 | | | | | 18 | | | 2019 report: Table 3 | | | | | | | | | | |  |  |  |
| Belavy 2019 & 2020 | | IVD distance | | LxAx | | 35.1 | | | | | | 2.4 | | | 18 | | | 34.4 | | | | 1.8 | | | | | 18 | | | 2019 report: Table 4 | | | | | | | | | | |  |  |  |
| Belavy 2019 & 2020 | | IVD height relative to vertebral body height | | LxAx | | 0.32 | | | | | | 0.03 | | | 18 | | | 0.3 | | | | 0.04 | | | | | 18 | | | 2019 report: Table 5 | | | | | | | | | | |  |  |  |
| Belavy 2019 & 2020 | | Whole disc T2-time | | L1-L2 | | 109.28 | | | | | | 20.29 | | | 18 | | | 95.25 | | | | 6.27 | | | | | 18 | | | 2019 report: Accessed from author | | | | | | | | | | |  |  |  |
| Belavy 2019 & 2020 | | Whole disc T2-time | | L2-L3 | | 104.83 | | | | | | 13.44 | | | 18 | | | 95.51 | | | | 10.87 | | | | | 18 | | | 2019 report: Accessed from author | | | | | | | | | | |  |  |  |
| Belavy 2019 & 2020 | | Whole disc T2-time | | L3-L4 | | 95.13 | | | | | | 12.82 | | | 18 | | | 92.83 | | | | 7.83 | | | | | 18 | | | 2019 report: Accessed from author | | | | | | | | | | |  |  |  |
| Belavy 2019 & 2020 | | Whole disc T2-time | | L4-L5 | | 90.7 | | | | | | 9.7 | | | 18 | | | 88.03 | | | | 10.76 | | | | | 18 | | | 2019 report: Accessed from author | | | | | | | | | | |  |  |  |
| Belavy 2019 & 2020 | | Whole disc T2-time | | L5-S1 | | 95.32 | | | | | | 15.97 | | | 18 | | | 88.91 | | | | 13.63 | | | | | 18 | | | 2019 report: Accessed from author | | | | | | | | | | |  |  |  |
| Belavy 2019 & 2020 | | T2-time nucleus/annulus ratio | | LxAx | | 1.49 | | | | | | 0.14 | | | 22 | | | 1.4 | | | | 0.15 | | | | | 24 | | | 2020 report: Accessed n from author, imputed SD from SE from Table 1, cycle group | | | | | | | | | | |  |  |  |
| Belavy 2019 & 2020 | | T2 weight signal intensity whole IVD | | LxAx | | 329.7 | | | | | | 23.92 | | | 22 | | | 326.9 | | | | 24 | | | | | 24 | | | 2020 report: Accessed n from author, imputed SD from SE from Table 1, cycle group | | | | | | | | | | |  |  |  |
| Belavy 2019 & 2020 | | T2 weight signal intensity nucleus/annulus ratio | | LxAx | | 3.29 | | | | | | 0.33 | | | 22 | | | 3.31 | | | | 0.34 | | | | | 24 | | | 2020 report: Accessed n from author, imputed SD from SE from Table 1, cycle group | | | | | | | | | | |  |  |  |
| Belavy 2019 & 2020 | | Dixon water fraction whole IVD | | LxAx | | 87.1 | | | | | | 3.28 | | | 22 | | | 86 | | | | 2.94 | | | | | 24 | | | 2020 report: Accessed n from author, imputed SD from SE from Table 1, cycle group | | | | | | | | | | |  |  |  |
| Belavy 2019 & 2020 | | Dixon water fraction nucleus/annulus ratio | | LxAx | | 1.01 | | | | | | 0.01 | | | 22 | | | 1.01 | | | | 0.01 | | | | | 24 | | | 2020 report: Accessed n from author, imputed SD from SE from Table 1, cycle group | | | | | | | | | | |  |  |  |
| **Belavy 2019 & 2020** | | **Synthetic effect size: ρ \| Hedges’ g \| standard error** | | | | | | | | | | | | | | | | | | | | | | | | | | | | | | | | | | | | | | |  |  |  |
|  | | ρ=0.0 \| 0.33 \| 0.12 | | | | | | | | | |  | | | | | | | | | | | | | | | | | | | | | |  | | | | | | |  |  |  |
|  | | ρ=0.2 \| 0.33 \| 0.12 | |  | |  | | | | | |  | | |  | | |  | | | |  | | | | |  | | |  | | | | | | | | | | |  |  |  |
|  | | ρ=0.4 \| 0.33 \| 0.12 | |  | |  | | | | | |  | | |  | | |  | | | |  | | | | |  | | |  | | | | | | | | | | |  |  |  |
|  | | ρ=0.6 \| 0.33 \| 0.12 | |  | |  | | | | | |  | | |  | | |  | | | |  | | | | |  | | |  | | | | | | | | | | |  |  |  |
|  | | ρ=0.8 \| 0.33 \| 0.12 | |  | |  | | | | | |  | | |  | | |  | | | |  | | | | |  | | |  | | | | | | | | | | |  |  |  |
|  | | ρ=1.0 \| 0.33 \| 0.12 | |  | |  | | | | | |  | | |  | | |  | | | |  | | | | |  | | |  | | | | | | | | | | |  |  |  |
| Benedikter 2022 | | IVD T2- time | | L2-L3 | | 32.82 | | | | | | 30.31 | | | 17 | | | 37.99 | | | | 32.71 | | | | | 37 | | | Table 2 | | | | | | | | | | |  |  |  |
| Benedikter 2022 | | IVD T2- time | | L3-L4 | | 35.94 | | | | | | 27.94 | | | 17 | | | 43.42 | | | | 32.49 | | | | | 37 | | | Table 2 | | | | | | | | | | |  |  |  |
| Benedikter 2022 | | IVD T2- time | | L4-L5 | | 40.31 | | | | | | 34.98 | | | 17 | | | 52.5 | | | | 39.76 | | | | | 37 | | | Table 2 | | | | | | | | | | |  |  |  |
| Benedikter 2022 | | IVD T2- time | | L5-S1 | | 39.05 | | | | | | 40.15 | | | 17 | | | 46.12 | | | | 35.09 | | | | | 37 | | | Table 2 | | | | | | | | | | |  |  |  |
| **Benedikter 2022** | | **Synthetic effect size: ρ \| Hedges’ g \| standard error** | | | | | | | | | | | | | | | | | | | | | | | | | | | | | | | | | | | | | | |  |  |  |
|  | | ρ=0.0 \| -0.22 \| 0.15 | | | | | | | | | | | | | | | |  | | | | | | | | | | | | | | | | | | | | | | |  |  |  |
|  | | ρ=0.2 \| -0.22 \| 0.15 | |  | |  | | | | | |  | | |  | | |  | | | |  | | | | |  | | |  | | | | | | | | | | |  |  |  |
|  | | ρ=0.4 \| -0.22 \| 0.15 | |  | |  | | | | | |  | | |  | | |  | | | |  | | | | |  | | |  | | | | | | | | | | |  |  |  |
|  | | ρ=0.6 \| -0.22 \| 0.15 | |  | |  | | | | | |  | | |  | | |  | | | |  | | | | |  | | |  | | | | | | | | | | |  |  |  |
|  | | ρ=0.8 \| -0.22 \| 0.15 | |  | |  | | | | | |  | | |  | | |  | | | |  | | | | |  | | |  | | | | | | | | | | |  |  |  |
|  | | ρ=1.0 \| -0.22 \| 0.15 | |  | |  | | | | | |  | | |  | | |  | | | |  | | | | |  | | |  | | | | | | | | | | |  |  |  |
| Frenken 2022 | | IVD glycosaminoglycan content (%), average lumbar spine | | LxAx | | 2.58 | | | | | | 0.27 | | | 17 | | | 1.66 | | | | 0.38 | | | | | 44 | | | Results section (pg.3) | | | | | | | | | | |  |  |  |
| Hangai 2009 & Owen 2021 | | IVD width | | L1-L2 | | 25 | | | | | | 3.6 | | | 47 | | | 25.7 | | | | 3.3 | | | | | 71 | | | 2021 report:Table 1, swimming group | | | | | | | | | | |  |  |  |
| Hangai 2009 & Owen 2021 | | IVD width | | L2-L3 | | 27.9 | | | | | | 3.4 | | | 47 | | | 27.7 | | | | 3.9 | | | | | 71 | | | 2021 report:Table 1, swimming group | | | | | | | | | | |  |  |  |
| Hangai 2009 & Owen 2021 | | IVD width | | L3-L4 | | 28.4 | | | | | | 3 | | | 47 | | | 28.7 | | | | 3.4 | | | | | 71 | | | 2021 report:Table 1, swimming group | | | | | | | | | | |  |  |  |
| Hangai 2009 & Owen 2021 | | IVD width | | L4-L5 | | 29 | | | | | | 3.7 | | | 47 | | | 27.8 | | | | 3.3 | | | | | 71 | | | 2021 report:Table 1, swimming group | | | | | | | | | | |  |  |  |
| Hangai 2009 & Owen 2021 | | IVD width | | L5-S1 | | 25.6 | | | | | | 4 | | | 47 | | | 22.8 | | | | 3 | | | | | 71 | | | 2021 report:Table 1, swimming group | | | | | | | | | | |  |  |  |
| Hangai 2009 & Owen 2021 | | IVD nucleus-annulus signal intensity ratio | | L1-L2 | | 3.59 | | | | | | 0.93 | | | 47 | | | 3.78 | | | | 0.87 | | | | | 71 | | | 2021 report:Table 2, swimming group | | | | | | | | | | |  |  |  |
| Hangai 2009 & Owen 2021 | | IVD nucleus-annulus signal intensity ratio | | L2-L3 | | 4.51 | | | | | | 0.93 | | | 47 | | | 4.4 | | | | 0.98 | | | | | 71 | | | 2021 report:Table 2, swimming group | | | | | | | | | | |  |  |  |
| Hangai 2009 & Owen 2021 | | IVD nucleus-annulus signal intensity ratio | | L3-L4 | | 4.94 | | | | | | 1.03 | | | 47 | | | 4.96 | | | | 1.09 | | | | | 71 | | | 2021 report:Table 2, swimming group | | | | | | | | | | |  |  |  |
| Hangai 2009 & Owen 2021 | | IVD nucleus-annulus signal intensity ratio | | L4-L5 | | 4.13 | | | | | | 1.26 | | | 47 | | | 4.34 | | | | 1.33 | | | | | 71 | | | 2021 report:Table 2, swimming group | | | | | | | | | | |  |  |  |
| Hangai 2009 & Owen 2021 | | IVD nucleus-annulus signal intensity ratio | | L5-S1 | | 3.2 | | | | | | 1.1 | | | 47 | | | 3.47 | | | | 1.16 | | | | | 71 | | | 2021 report:Table 2, swimming group | | | | | | | | | | |  |  |  |
| Hangai 2009 & Owen 2021 | | IVD height to vertebral body height ratio | | L1-L2 | | 0.36 | | | | | | 0.04 | | | 47 | | | 0.31 | | | | 0.04 | | | | | 71 | | | 2021 report:Table 2, swimming group | | | | | | | | | | |  |  |  |
| Hangai 2009 & Owen 2021 | | IVD height to vertebral body height ratio | | L2-L3 | | 0.37 | | | | | | 0.05 | | | 47 | | | 0.33 | | | | 0.05 | | | | | 71 | | | 2021 report:Table 2, swimming group | | | | | | | | | | |  |  |  |
| Hangai 2009 & Owen 2021 | | IVD height to vertebral body height ratio | | L3-L4 | | 0.41 | | | | | | 0.05 | | | 47 | | | 0.38 | | | | 0.05 | | | | | 71 | | | 2021 report:Table 2, swimming group | | | | | | | | | | |  |  |  |
| Hangai 2009 & Owen 2021 | | IVD height to vertebral body height ratio | | L4-L5 | | 0.47 | | | | | | 0.06 | | | 47 | | | 0.43 | | | | 0.07 | | | | | 71 | | | 2021 report:Table 2, swimming group | | | | | | | | | | |  |  |  |
| Hangai 2009 & Owen 2021 | | IVD height to vertebral body height ratio | | L5-S1 | | 0.45 | | | | | | 0.07 | | | 47 | | | 0.43 | | | | 0.08 | | | | | 71 | | | 2021 report:Table 2, swimming group | | | | | | | | | | |  |  |  |
| **Hangai 2009 & Owen 2021** | | **Synthetic effect size: ρ \| Hedges’ g \| standard error** | | | | | | | | | | | | | | | | | | | | | | | | | | | | | | | | | | | | | | |  |  |  |
|  | | ρ=0.0 \| 0.26 \| 0.05 | | | | | | | | | |  | | | | | | | | | | | | | | | | | | | | | |  | | | | | | |  |  |  |
|  | | ρ=0.2 \| 0.26 \| 0.05 | |  | |  | | | | | |  | | |  | | |  | | | |  | | | | |  | | |  | | | | | | | | | | |  |  |  |
|  | | ρ=0.4 \| 0.26 \| 0.05 | |  | |  | | | | | |  | | |  | | |  | | | |  | | | | |  | | |  | | | | | | | | | | |  |  |  |
|  | | ρ=0.6 \| 0.26 \| 0.05 | |  | |  | | | | | |  | | |  | | |  | | | |  | | | | |  | | |  | | | | | | | | | | |  |  |  |
|  | | ρ=0.8 \| 0.26 \| 0.05 | |  | |  | | | | | |  | | |  | | |  | | | |  | | | | |  | | |  | | | | | | | | | | |  |  |  |
|  | | ρ=1.0 \| 0.26 \| 0.05 | |  | |  | | | | | |  | | |  | | |  | | | |  | | | | |  | | |  | | | | | | | | | | |  |  |  |
| **Primary subgroup synthesis: Extreme trunk range of motion, cross-sectional** | | | | | | | | | | | | | | | | | | | | | | | | | | | | | | | | | | | | | | | | |  |  |  |
| Jentzsch 2020 | | IVD T2-values average whole spine | | LxAx | | 85.6 | | | | | | 14.9 | | | 12 | | | 87.2 | | | | 13.5 | | | | | 13 | | | Table 2, evening data | | | | | | | | | | |  |  |  |
| Hangai 2009 & Owen 2021 | | IVD width | | L1-L2 | | 27.72 | | | | | | 3.1 | | | 108 | | | 25.7 | | | | 3.3 | | | | | 71 | | | 2021 report Table 1, baseball and kendo | | | | | | | | | | |  |  |  |
| Hangai 2009 & Owen 2021 | | IVD width | | L2-L3 | | 28.83 | | | | | | 3.57 | | | 108 | | | 27.7 | | | | 3.9 | | | | | 71 | | | 2021 report Table 1, baseball and kendo | | | | | | | | | | |  |  |  |
| Hangai 2009 & Owen 2021 | | IVD width | | L3-L4 | | 29.32 | | | | | | 3.29 | | | 108 | | | 28.7 | | | | 3.4 | | | | | 71 | | | 2021 report Table 1, baseball and kendo | | | | | | | | | | |  |  |  |
| Hangai 2009 & Owen 2021 | | IVD width | | L4-L5 | | 28.82 | | | | | | 3.16 | | | 108 | | | 27.8 | | | | 3.3 | | | | | 71 | | | 2021 report Table 1, baseball and kendo | | | | | | | | | | |  |  |  |
| Hangai 2009 & Owen 2021 | | IVD width | | L5-S1 | | 23.78 | | | | | | 3.18 | | | 108 | | | 22.8 | | | | 3 | | | | | 71 | | | 2021 report Table 1, baseball and kendo | | | | | | | | | | |  |  |  |
| Hangai 2009 & Owen 2021 | | IVD nucleus-annulus signal intensity ratio | | L1-L2 | | 3.61 | | | | | | 0.83 | | | 108 | | | 3.78 | | | | 0.87 | | | | | 71 | | | 2021 report Table 1, baseball and kendo | | | | | | | | | | |  |  |  |
| Hangai 2009 & Owen 2021 | | IVD nucleus-annulus signal intensity ratio | | L2-L3 | | 4.38 | | | | | | 0.94 | | | 108 | | | 4.4 | | | | 0.98 | | | | | 71 | | | 2021 report Table 1, baseball and kendo | | | | | | | | | | |  |  |  |
| Hangai 2009 & Owen 2021 | | IVD nucleus-annulus signal intensity ratio | | L3-L4 | | 4.75 | | | | | | 1.04 | | | 108 | | | 4.96 | | | | 1.09 | | | | | 71 | | | 2021 report Table 1, baseball and kendo | | | | | | | | | | |  |  |  |
| Hangai 2009 & Owen 2021 | | IVD nucleus-annulus signal intensity ratio | | L4-L5 | | 4.04 | | | | | | 1.28 | | | 108 | | | 4.34 | | | | 1.33 | | | | | 71 | | | 2021 report Table 1, baseball and kendo | | | | | | | | | | |  |  |  |
| Hangai 2009 & Owen 2021 | | IVD nucleus-annulus signal intensity ratio | | L5-S1 | | 3.2 | | | | | | 1.11 | | | 108 | | | 3.47 | | | | 1.16 | | | | | 71 | | | 2021 report Table 1, baseball and kendo | | | | | | | | | | |  |  |  |
| Hangai 2009 & Owen 2021 | | IVD height to vertebral body height ratio | | L1-L2 | | 0.31 | | | | | | 0.04 | | | 108 | | | 0.31 | | | | 0.04 | | | | | 71 | | | 2021 report Table 1, baseball and kendo | | | | | | | | | | |  |  |  |
| Hangai 2009 & Owen 2021 | | IVD height to vertebral body height ratio | | L2-L3 | | 0.35 | | | | | | 0.05 | | | 108 | | | 0.33 | | | | 0.05 | | | | | 71 | | | 2021 report Table 1, baseball and kendo | | | | | | | | | | |  |  |  |
| Hangai 2009 & Owen 2021 | | IVD height to vertebral body height ratio | | L3-L4 | | 0.4 | | | | | | 0.05 | | | 108 | | | 0.38 | | | | 0.05 | | | | | 71 | | | 2021 report Table 1, baseball and kendo | | | | | | | | | | |  |  |  |
| Hangai 2009 & Owen 2021 | | IVD height to vertebral body height ratio | | L4-L5 | | 0.45 | | | | | | 0.07 | | | 108 | | | 0.43 | | | | 0.07 | | | | | 71 | | | 2021 report Table 1, baseball and kendo | | | | | | | | | | |  |  |  |
| Hangai 2009 & Owen 2021 | | IVD height to vertebral body height ratio | | L5-S1 | | 0.42 | | | | | | 0.07 | | | 108 | | | 0.43 | | | | 0.08 | | | | | 71 | | | 2021 report Table 1, baseball and kendo | | | | | | | | | | |  |  |  |
| **Hangai 2009 & Owen 2021** | | **Synthetic effect size: ρ \| Hedges’ g \| standard error** | | | | | | | | | | | | | | | | | | | | | | | | | | | | | | | | | | | | | | |  |  |  |
|  | | ρ=0.0 \| 0.12 \| 0.04 | | | | | | | | | | | | | | | |  | | | | | | | | | | | | | | | | | | | | | | |  |  |  |
|  | | ρ=0.2 \| 0.12 \| 0.04 | |  | |  | | | | | |  | | |  | | |  | | | |  | | | | |  | | |  | | | | | | | | | | |  |  |  |
|  | | ρ=0.4 \| 0.12 \| 0.04 | |  | |  | | | | | |  | | |  | | |  | | | |  | | | | |  | | |  | | | | | | | | | | |  |  |  |
|  | | ρ=0.6 \| 0.12 \| 0.04 | |  | |  | | | | | |  | | |  | | |  | | | |  | | | | |  | | |  | | | | | | | | | | |  |  |  |
|  | | ρ=0.8 \| 0.12 \| 0.04 | |  | |  | | | | | |  | | |  | | |  | | | |  | | | | |  | | |  | | | | | | | | | | |  |  |  |
|  | | ρ=1.0 \| 0.12 \| 0.04 | |  | |  | | | | | |  | | |  | | |  | | | |  | | | | |  | | |  | | | | | | | | | | |  |  |  |
| Vadala 2014 | | IVD T1p of nucleus pulposus | | L1-L2 | | 102.53 | | | | | | 26.91 | | | 13 | | | 116.1 | | | | 36.54 | | | | | 13 | | | Imputed from median IQR to mean SD in Table 1 | | | | | | | | | | |  |  |  |
| Vadala 2014 | | IVD T1p of nucleus pulposus | | L2-L3 | | 99.65 | | | | | | 17.77 | | | 13 | | | 114.9 | | | | 22.59 | | | | | 13 | | | Imputed from median IQR to mean SD in Table 1 | | | | | | | | | | |  |  |  |
| Vadala 2014 | | IVD T1p of nucleus pulposus | | L3-L4 | | 94.18 | | | | | | 16.53 | | | 13 | | | 112.2 | | | | 28.16 | | | | | 13 | | | Imputed from median IQR to mean SD in Table 1 | | | | | | | | | | |  |  |  |
| Vadala 2014 | | IVD T1p of nucleus pulposus | | L4-L5 | | 77.48 | | | | | | 20.51 | | | 13 | | | 110.7 | | | | 21.59 | | | | | 13 | | | Imputed from median IQR to mean SD in Table 1 | | | | | | | | | | |  |  |  |
| Vadala 2014 | | IVD T1p of nucleus pulposus | | L5-S1 | | 79.78 | | | | | | 21.76 | | | 13 | | | 100.7 | | | | 24.92 | | | | | 13 | | | Imputed from median IQR to mean SD in Table 1 | | | | | | | | | | |  |  |  |
| **Vadala 2014** | | **Synthetic effect size: ρ \| Hedges’ g \| standard error** | | | | | | | | | | | | | | | | | | | | | | | | | | | | | | | | | | | | | | |  |  |  |
|  | | ρ=0.0 \| -0.86 \| 0.19 | | | | | | | | | | | | | | | | | | | | | | | |  | | | | | | | | | | | |  | | |  |  |  |
|  | | ρ=0.2 \| -0.86 \| 0.19 | |  | |  | | | | | |  | | |  | | |  | | | |  | | | | |  | | |  | | | | | | | | | | |  |  |  |
|  | | ρ=0.4 \| -0.86 \| 0.19 | |  | |  | | | | | |  | | |  | | |  | | | |  | | | | |  | | |  | | | | | | | | | | |  |  |  |
|  | | ρ=0.6 \| -0.86 \| 0.19 | |  | |  | | | | | |  | | |  | | |  | | | |  | | | | |  | | |  | | | | | | | | | | |  |  |  |
|  | | ρ=0.8 \| -0.86 \| 0.19 | |  | |  | | | | | |  | | |  | | |  | | | |  | | | | |  | | |  | | | | | | | | | | |  |  |  |
|  | | ρ=1.0 \| -0.86 \| 0.19 | |  | |  | | | | | |  | | |  | | |  | | | |  | | | | |  | | |  | | | | | | | | | | |  |  |  |
| Zhang 2023 | | Nucleus pulposus T2 time, Average lumbar | | LxAx | | 110.7 | | | | | | 24.92 | | | *27* | | | 98.3 | | | | 22.2 | | | | | 18 | | | Imputed from Figure 4 (WebPlotDigitizer) | | | | | | | | | | |  |  |  |
| **Primary subgroup synthesis: Aerobic exercise, cross-sectional** | | | | | | | | | | | | | | | | | | | | | | | | | | | | | | | | | | | | | | | | |  |  |  |
| Belavy 2017 & 2019 & 2020 | | IVD anteroposterior width average lumbar spine | | LxAx | | 26.25 | | | | | | 3.16 | | | 73 | | | 25.6 | | | | 2.4 | | | | | 24 | | | Averaged from 2 reports: 2017: Table 1, pooled running groups and 2019: Table 2 | | | | | | | | | | |  |  |  |
| Belavy 2017 & 2019 & 2020 | | IVD average area lumbar average | | LxAx | | 263.33 | | | | | | 59.39 | | | 55 | | | 250.2 | | | | 41.2 | | | | | 24 | | | Averaged from 2 reports: 2017: Table 1, pooled running groups and 2019: Table 2 | | | | | | | | | | |  |  |  |
| Belavy 2017 & 2019 & 2020 | | IVD average distance lumbar average | | LxAx | | 34.31 | | | | | | 2.75 | | | 73 | | | 34.2 | | | | 2 | | | | | 24 | | | Averaged from 2 reports: 2017: Table 1, pooled running groups and 2019: Table 2 | | | | | | | | | | |  |  |  |
| Belavy 2017 & 2019 & 2020 | | IVD average volume lumbar average | | LxAx | | 10.3 | | | | | | 3.14 | | | 73 | | | 9.5 | | | | 2.3 | | | | | 24 | | | Averaged from 2 reports: 2017: Table 1, pooled running groups and 2019: Table 2 | | | | | | | | | | |  |  |  |
| Belavy 2017 & 2019 & 2020 | | IVD height relative to vertebral body height, average lumbar spine | | LxAx | | 0.3 | | | | | | 0.05 | | | 73 | | | 0.28 | | | | 0.05 | | | | | 24 | | | Averaged from 2 reports: 2020: Imputed from Figure 2 (WebPlotDigitizer), pooled running groups, averaged levels and 2019: Table 2 | | | | | | | | | | |  |  |  |
| Belavy 2017 & 2019 & 2020 | | IVD T2-time | | L1-L2 | | 116.24 | | | | | | 20.2 | | | 73 | | | 107.6 | | | | 6.62 | | | | | 24 | | | Averaged from 2 reports: 2017: Imputed from Figure 2 (WebPlotDigitizer), pooled running groups and 2019: accessed from author | | | | | | | | | | |  |  |  |
| Belavy 2017 & 2019 & 2020 | | IVD T2- time | | L2-L3 | | 114.99 | | | | | | 17.08 | | | 73 | | | 108.8 | | | | 11.49 | | | | | 24 | | | Averaged from 2 reports: 2017: Imputed from Figure 2 (WebPlotDigitizer), pooled running groups and 2019: accessed from author | | | | | | | | | | |  |  |  |
| Belavy 2017 & 2019 & 2020 | | IVD T2- time | | L3-L4 | | 111.19 | | | | | | 18.29 | | | 73 | | | 108.3 | | | | 12.61 | | | | | 24 | | | Averaged from 2 reports: 2017: Imputed from Figure 2 (WebPlotDigitizer), pooled running groups and 2019: accessed from author | | | | | | | | | | |  |  |  |
| Belavy 2017 & 2019 & 2020 | | IVD T2- time | | L4-L5 | | 105.65 | | | | | | 18.93 | | | 73 | | | 100.1 | | | | 16.97 | | | | | 24 | | | Averaged from 2 reports: 2017: Imputed from Figure 2 (WebPlotDigitizer), pooled running groups and 2019: accessed from author | | | | | | | | | | |  |  |  |
| Belavy 2017 & 2019 & 2020 | | IVD T2- time | | L5-S1 | | 104.91 | | | | | | 19.31 | | | 73 | | | 94.98 | | | | 20.71 | | | | | 24 | | | Averaged from 2 reports: 2017: Imputed from Figure 2 (WebPlotDigitizer), pooled running groups and 2019: accessed from author | | | | | | | | | | |  |  |  |
| Belavy 2017 & 2019 & 2020 | | T2-time nucleus/annulus rati0 | | LxAx | | 1.49 | | | | | | 0.13 | | | 77 | | | 1.4 | | | | 0.15 | | | | | 24 | | | 2020 report: Accessed n from author, SD imputed from SE from Table 1, pooled exercise groups | | | | | | | | | | |  |  |  |
| Belavy 2017 & 2019 & 2020 | | T2 weight signal intensity whole IVD | | LxAx | | 322 | | | | | | 24.23 | | | 77 | | | 326.9 | | | | 24 | | | | | 24 | | | 2020 report: Accessed n from author, SD imputed from SE from Table 1, pooled exercise groups | | | | | | | | | | |  |  |  |
| Belavy 2017 & 2019 & 2020 | | T2 weight signal intensity nucleus/annulus ratio | | LxAx | | 3.35 | | | | | | 0.34 | | | 77 | | | 3.31 | | | | 0.34 | | | | | 24 | | | 2020 report: Accessed n from author, SD imputed from SE from Table 1, pooled exercise groups | | | | | | | | | | |  |  |  |
| Belavy 2017 & 2019 & 2020 | | Dixon water fraction whole IVD | | LxAx | | 87.42 | | | | | | 3.19 | | | 77 | | | 86 | | | | 2.94 | | | | | 24 | | | 2020 report: Accessed n from author, SD imputed from SE from Table 1, pooled exercise groups | | | | | | | | | | |  |  |  |
| Belavy 2017 & 2019 & 2020 | | Dixon water fraction nucleus/annulus ratio | | LxAx | | 1.01 | | | | | | 0.01 | | | 77 | | | 1.01 | | | | 0.01 | | | | | 24 | | | 2020 report: Accessed n from author, SD imputed from SE from Table 1, pooled exercise groups | | | | | | | | | | |  |  |  |
| **Belavy 2017 & 2019 & 2020** | | **Synthetic effect size: ρ \| Hedges’ g \| standard error** | | | | | | | | | | | | | | | | | | | | | | | | | | | | | | | | | | | | | | |  |  |  |
|  | | ρ=0.0 \| 0.27 \| 0.06 | | | | | |  | | | | | | | | | |  | | | | | | | | | | | | | | |  | | | | | | | |  |  |  |
|  | | ρ=0.2 \| 0.27 \| 0.06 | |  | |  | | | | | |  | | |  | | |  | | | |  | | | | |  | |  | | | | | | | | | | | |  |  |  |
|  | | ρ=0.4 \| 0.27 \| 0.06 | |  | |  | | | | | |  | | |  | | |  | | | |  | | | | |  | |  | | | | | | | | | | | |  |  |  |
|  | | ρ=0.6 \| 0.27 \| 0.06 | |  | |  | | | | | |  | | |  | | |  | | | |  | | | | |  | |  | | | | | | | | | | | |  |  |  |
|  | | ρ=0.8 \| 0.27 \| 0.06 | |  | |  | | | | | |  | | |  | | |  | | | |  | | | | |  | |  | | | | | | | | | | | |  |  |  |
|  | | ρ=1.0 \| 0.27 \| 0.06 | |  | |  | | | | | |  | | |  | | |  | | | |  | | | | |  | |  | | | | | | | | | | | |  |  |  |
| Benedikter 2022 | | IVD T2- time | | L2-L3 | | 32.82 | | | | | | 30.31 | | | 17 | | | 37.99 | | | | 32.71 | | | | | 37 | | Table 2 | | | | | | | | | | | |  |  |  |
| Benedikter 2022 | | IVD T2- time | | L3-L4 | | 35.94 | | | | | | 27.94 | | | 17 | | | 43.42 | | | | 32.49 | | | | | 37 | | Table 2 | | | | | | | | | | | |  |  |  |
| Benedikter 2022 | | IVD T2- time | | L4-L5 | | 40.31 | | | | | | 34.98 | | | 17 | | | 52.5 | | | | 39.76 | | | | | 37 | | Table 2 | | | | | | | | | | | |  |  |  |
| Benedikter 2022 | | IVD T2- time | | L5-S1 | | 39.05 | | | | | | 40.15 | | | 17 | | | 46.12 | | | | 35.09 | | | | | 37 | | Table 2 | | | | | | | | | | | |  |  |  |
| **Benedikter 2022** | | **Synthetic effect size:ρ \| Hedges’ g \| standard error** | | | | | | | | | | | | | | | | | | | | | | | | | | | | | | | | | | | | | | |  |  |  |
|  | | ρ=0.0 \| -0.22 \| 0.15 | | | | | | | | | | | | | | | |  | | | | | | | | | | | | | | | | | | | | | | |  |  |  |
|  | | ρ=0.2 \| -0.22 \| 0.15 | |  | |  | | | | | |  | | |  | | |  | | | |  | | | | |  | |  | | | | | | | | | | | |  |  |  |
|  | | ρ=0.4 \| -0.22 \| 0.15 | |  | |  | | | | | |  | | |  | | |  | | | |  | | | | |  | |  | | | | | | | | | | | |  |  |  |
|  | | ρ=0.6 \| -0.22 \| 0.15 | |  | |  | | | | | |  | | |  | | |  | | | |  | | | | |  | |  | | | | | | | | | | | |  |  |  |
|  | | ρ=0.8 \| -0.22 \| 0.15 | |  | |  | | | | | |  | | |  | | |  | | | |  | | | | |  | |  | | | | | | | | | | | |  |  |  |
|  | | ρ=1.0 \| -0.22 \| 0.15 | |  | |  | | | | | |  | | |  | | |  | | | |  | | | | |  | |  | | | | | | | | | | | |  |  |  |
| Bowden 2018 | | IVD Fractional anisotropy | | LxAx | | 0.15 | | | | | | 0.06 | | | 14 | | | 0.17 | | | | 0.11 | | | | | 12 | | Table 3 | | | | | | | | | | | |  |  |  |
| Bowden 2018 | | IVD apparent diffusion coefficient | | LxAx | | 1.15 | | | | | | 0.48 | | | 14 | | | 0.79 | | | | 0.49 | | | | | 12 | | Table 3 | | | | | | | | | | | |  |  |  |
| Bowden 2018 | | IVD T2 signal intensity | | LxAx | | 55.8 | | | | | | 24.7 | | | 14 | | | 49.7 | | | | 21.4 | | | | | 12 | | Table 3 | | | | | | | | | | | |  |  |  |
| **Bowden 2018** | | **Synthetic effect size: ρ \| Hedges’ g \| standard error** | | | | | | | | | | | | | | | | | | | | | | | | | | | | | | | | | | | | | | |  |  |  |
|  | | ρ=0.0 \| 0.40 \| 0.23 | | | | | | | | | | | | | | | |  | | | | | | | | | | | | | | | | | | | | | | |  |  |  |
|  | | ρ=0.2 \| 0.40 \| 0.24 | |  | |  | | | | | |  | | |  | | |  | | | |  | | | | |  | |  | | | | | | | | | | | |  |  |  |
|  | | ρ=0.4 \| 0.40 \| 0.24 | |  | |  | | | | | |  | | |  | | |  | | | |  | | | | |  | |  | | | | | | | | | | | |  |  |  |
|  | | ρ=0.6 \| 0.40 \| 0.25 | |  | |  | | | | | |  | | |  | | |  | | | |  | | | | |  | |  | | | | | | | | | | | |  |  |  |
|  | | ρ=0.8 \| 0.40 \| 0.25 | |  | |  | | | | | |  | | |  | | |  | | | |  | | | | |  | |  | | | | | | | | | | | |  |  |  |
|  | | ρ=1.0 \| 0.40 \| 0.26 | |  | |  | | | | | |  | | |  | | |  | | | |  | | | | |  | |  | | | | | | | | | | | |  |  |  |
| Frenken 2022 | | IVD glycosaminoglycan content (%) | | LxAx | | 2.58 | | | | | | 0.27 | | | 17 | | | 1.66 | | | | 0.38 | | | | | 44 | | Results section (pg.3) | | | | | | | | | | | |  |  |  |
| Lagerstrand 2021 & Rosenqvist 2023 | | IVD T2 value subregion 1 | | Lx/Tx Ax | | 0.74 | | | | | | 0.33 | | | 58 | | | 0.76 | | | | 0.34 | | | | | 26 | | 2021 report: Imputed from Figure 3 (WebPlotDigitizer) | | | | | | | | | | | |  |  |  |
| Lagerstrand 2021 & Rosenqvist 2023 | | IVD T2 value subregion 2 | | Lx/Tx Ax | | 1.06 | | | | | | 0.35 | | | 58 | | | 1.14 | | | | 0.4 | | | | | 26 | | 2021 report: Imputed from Figure 3 (WebPlotDigitizer) | | | | | | | | | | | |  |  |  |
| Lagerstrand 2021 & Rosenqvist 2023 | | IVD T2 value subregion 3 | | Lx/Tx Ax | | 1.3 | | | | | | 0.48 | | | 58 | | | 1.33 | | | | 0.43 | | | | | 26 | | 2021 report: Imputed from Figure 3 (WebPlotDigitizer) | | | | | | | | | | | |  |  |  |
| Lagerstrand 2021 & Rosenqvist 2023 | | IVD T2 value subregion 4 | | Lx/Tx Ax | | 1.13 | | | | | | 0.44 | | | 58 | | | 1.12 | | | | 0.41 | | | | | 26 | | 2021 report: Imputed from Figure 3 (WebPlotDigitizer) | | | | | | | | | | | |  |  |  |
| Lagerstrand 2021 & Rosenqvist 2023 | | IVD T2 value subregion 5 | | Lx/Tx Ax | | 0.53 | | | | | | 0.28 | | | 58 | | | 0.47 | | | | 0.23 | | | | | 26 | | 2021 report: Imputed from Figure 3 (WebPlotDigitizer) | | | | | | | | | | | |  |  |  |
| **Lagerstrand 2021 & Rosenqvist 2023** | | **Synthetic effect size: ρ \| Hedges’ g \| standard error** | | | | | | | | | | | | | | | | | | | | | | | | | | | | | | | | | | | | | | |  |  |  |
|  | | ρ=0.0 \| -0.02 \| 0.11 | | | | | | | | |  | | | | | | | | | | | | | | | | | | | | |  | | | | | | | | |  |  |  |
|  | ρ=0.2 \| -0.02 \| 0.11 | |  | | | |  | | |  | | | |  | | |  | | |  | | | |  | | | |  | | | | | | | | | | | | |  |  |  |
|  | ρ=0.4 \| -0.02 \| 0.11 | |  | | | |  | | |  | | | |  | | |  | | |  | | | |  | | | |  | | | | | | | | | | | | |  |  |  |
|  | ρ=0.6 \| -0.02 \| 0.11 | |  | | | |  | | |  | | | |  | | |  | | |  | | | |  | | | |  | | | | | | | | | | | | |  |  |  |
|  | ρ=0.8 \| -0.02 \| 0.11 | |  | | | |  | | |  | | | |  | | |  | | |  | | | |  | | | |  | | | | | | | | | | | | |  |  |  |
|  | ρ=1.0 \| -0.02 \| 0.11 | |  | | | |  | | |  | | | |  | | |  | | |  | | | |  | | | |  | | | | | | | | | | | | |  |  |  |
| Li 2024 | | IVD T2 average annulus | | L1-L2 | | 21.54 | | | | | | 4.47 | | | 54 | | | 19.42 | | | | 3.68 | | | | | 30 | | | Imputed from Figure 4A (WebPlotDigitizer), | | | | | | | | | | |  |  |  |
| Li 2024 | | IVD T2 average annulus | | L2-L3 | | 22.5 | | | | | | 5.07 | | | 54 | | | 21.28 | | | | 5.09 | | | | | 30 | | | Imputed from Figure 4A (WebPlotDigitizer), | | | | | | | | | | |  |  |  |
| Li 2024 | | IVD T2 average annulus | | L3-L4 | | 21.99 | | | | | | 4.72 | | | 54 | | | 19.35 | | | | 3.47 | | | | | 30 | | | Imputed from Figure 4A (WebPlotDigitizer), | | | | | | | | | | |  |  |  |
| Li 2024 | | IVD T2 average annulus | | L4-L5 | | 23.3 | | | | | | 5.3 | | | 54 | | | 21.68 | | | | 4.58 | | | | | 30 | | | Imputed from Figure 4A (WebPlotDigitizer), | | | | | | | | | | |  |  |  |
| Li 2024 | | IVD T2 average annulus | | L5-S1 | | 21.59 | | | | | | 5.55 | | | 54 | | | 19.85 | | | | 4.51 | | | | | 30 | | | Imputed from Figure 4A (WebPlotDigitizer), | | | | | | | | | | |  |  |  |
| Li 2024 | | IVD T2 average nucleus | | L1-L2 | | 42.23 | | | | | | 11.27 | | | 54 | | | 39.95 | | | | 10.72 | | | | | 30 | | | Imputed from Figure 4B (WebPlotDigitizer), | | | | | | | | | | |  |  |  |
| Li 2024 | | IVD T2 average nucleus | | L2-L3 | | 47.51 | | | | | | 7.74 | | | 54 | | | 44.71 | | | | 13.51 | | | | | 30 | | | Imputed from Figure 4B (WebPlotDigitizer), | | | | | | | | | | |  |  |  |
| Li 2024 | | IVD T2 average nucleus | | L3-L4 | | 49.34 | | | | | | 13.4 | | | 54 | | | 47.01 | | | | 15.79 | | | | | 30 | | | Imputed from Figure 4B (WebPlotDigitizer), | | | | | | | | | | |  |  |  |
| Li 2024 | | IVD T2 average nucleus | | L4-L5 | | 51.21 | | | | | | 15.94 | | | 54 | | | 43.3 | | | | 15.84 | | | | | 30 | | | Imputed from Figure 4B (WebPlotDigitizer), | | | | | | | | | | |  |  |  |
| Li 2024 | | IVD T2 average nucleus | | L5-S1 | | 47.66 | | | | | | 18.48 | | | 54 | | | 44.16 | | | | 17.41 | | | | | 30 | | | Imputed from Figure 4B (WebPlotDigitizer), | | | | | | | | | | |  |  |  |
| **Li 2024** | | **Synthetic effect size: ρ \| Hedges’ g \| standard error** | | | | | | | | | | | | | | | | | | | |  | | | | |  | | |  | | | | | | | | | | |  |  |  |
|  | | ρ=0.0 \| 0.33 \| 0.07 | |  | |  | | | | | |  | | |  | | |  | | | |  | | | | |  | | |  | | | | | | | | | | |  |  |  |
|  | | ρ=0.2 \| -0.33 \| 0.07 | |  | |  | | | | | |  | | |  | | |  | | | |  | | | | |  | | |  | | | | | | | | | | |  |  |  |
|  | | ρ=0.4 \| 0.33 \| 0.07 | |  | |  | | | | | |  | | |  | | |  | | | |  | | | | |  | | |  | | | | | | | | | | |  |  |  |
|  | | ρ=0.6 \| 0.33 \| 0.07 | |  | |  | | | | | |  | | |  | | |  | | | |  | | | | |  | | |  | | | | | | | | | | |  |  |  |
|  | | ρ=0.8 \| 0.33 \| 0.07 | |  | |  | | | | | |  | | |  | | |  | | | |  | | | | |  | | |  | | | | | | | | | | |  |  |  |
|  | | ρ=1.0 \| 0.33 \| 0.07 | |  | |  | | | | | |  | | |  | | |  | | | |  | | | | |  | | |  | | | | | | | | | | |  |  |  |
| Mitchell 2020 | IVD height to vertebral body height ratio | | L2-L3 | | | | 0.33 | | | 0.04 | | | | 9 | | | 0.23 | | | 0.02 | | | | 8 | | | | Table 3 | | | | | | | | | | | | |  |  |  |
| Mitchell 2020 | IVD height to vertebral body height ratio | | L3-L4 | | | | 0.32 | | | 0.05 | | | | 9 | | | 0.25 | | | 0.03 | | | | 8 | | | | Table 4 | | | | | | | | | | | | |  |  |  |
| Mitchell 2020 | IVD height to vertebral body height ratio | | L4-L5 | | | | 0.31 | | | 0.06 | | | | 9 | | | 0.26 | | | 0.04 | | | | 8 | | | | Table 4 | | | | | | | | | | | | |  |  |  |
| Mitchell 2020 | IVD height to vertebral body height ratio | | L5-S1 | | | | 0.28 | | | 0.05 | | | | 9 | | | 0.22 | | | 0.04 | | | | 8 | | | | Table 4 | | | | | | | | | | | | |  |  |  |
| Mitchell 2020 | IVD T2- time | | L2-L3 | | | | 101.2 | | | 13.6 | | | | 9 | | | 93.1 | | | 26.6 | | | | 8 | | | | Table 4 | | | | | | | | | | | | |  |  |  |
| Mitchell 2020 | IVD T2- time | | L3-L4 | | | | 95.1 | | | 13.9 | | | | 9 | | | 89.6 | | | 26.8 | | | | 8 | | | | Table 4 | | | | | | | | | | | | |  |  |  |
| Mitchell 2020 | IVD T2- time | | L4-L5 | | | | 83.9 | | | 12.2 | | | | 9 | | | 85.7 | | | 32.7 | | | | 8 | | | | Table 4 | | | | | | | | | | | | |  |  |  |
| Mitchell 2020 | IVD T2- time | | L5-S1 | | | | 85.1 | | | 14.8 | | | | 9 | | | 78.8 | | | 14.9 | | | | 8 | | | | Table 4 | | | | | | | | | | | | |  |  |  |
| Mitchell 2020 | Apparent diffusion coefficient | | L5-S1 | | | | 249 | | | 175.2 | | | | 9 | | | 202.3 | | | 149.5 | | | | 8 | | | | Table 4 | | | | | | | | | | | | |  |  |  |
| **Mitchell 2020** | **Synthetic effect size: ρ \| Hedges’ g \| standard error** | | | | | | | | | | | | | | | | | | | | | | | | | | | | | | | | | | | | | | | |  |  |  |
|  | ρ=0.0 \| 0.88 \| 0.18 | | | |  | | | | | | | | | | | |  | | | | | | | | | | | | | |  | | | | | | | | | |  |  |  |
|  | ρ=0.2 \| 0.88 \| 0.18 | |  | | | |  | | |  | | | |  | | |  | | |  | | | |  | | | |  | | | | | | | | | | | | |  |  |  |
|  | ρ=0.4 \| 0.88 \| 0.18 | |  | | | |  | | |  | | | |  | | |  | | |  | | | |  | | | |  | | | | | | | | | | | | |  |  |  |
|  | ρ=0.6 \| 0.88 \| 0.18 | |  | | | |  | | |  | | | |  | | |  | | |  | | | |  | | | |  | | | | | | | | | | | | |  |  |  |
|  | ρ=0.8 \| 0.88 \| 0.18 | |  | | | |  | | |  | | | |  | | |  | | |  | | | |  | | | |  | | | | | | | | | | | | |  |  |  |
|  | ρ=1.0 \| 0.88 \| 0.18 | |  | | | |  | | |  | | | |  | | |  | | |  | | | |  | | | |  | | | | | | | | | | | | |  |  |  |
| Hangai 2009 & Owen 2021 | IVD width | | L1-L2 | | | | 26.57 | | | 3.38 | | | | 251 | | | 25.7 | | | 3.3 | | | | 71 | | | | 2021 report Table 1, all sports pooled except baseball | | | | | | | | | | | | |  |  |  |
| Hangai 2009 & Owen 2021 | IVD width | | L2-L3 | | | | 28.48 | | | 3.32 | | | | 251 | | | 27.7 | | | 3.9 | | | | 71 | | | | 2021 report Table 1, all sports pooled except baseball | | | | | | | | | | | | |  |  |  |
| Hangai 2009 & Owen 2021 | IVD width | | L3-L4 | | | | 29.15 | | | 3.28 | | | | 251 | | | 28.7 | | | 3.4 | | | | 71 | | | | 2021 report Table 1, all sports pooled except baseball | | | | | | | | | | | | |  |  |  |
| Hangai 2009 & Owen 2021 | IVD width | | L4-L5 | | | | 28.64 | | | 3.58 | | | | 251 | | | 27.8 | | | 3.3 | | | | 71 | | | | 2021 report Table 1, all sports pooled except baseball | | | | | | | | | | | | |  |  |  |
| Hangai 2009 & Owen 2021 | IVD width | | L5-S1 | | | | 23.81 | | | 3.4 | | | | 251 | | | 22.8 | | | 3 | | | | 71 | | | | 2021 report Table 1, all sports pooled except baseball | | | | | | | | | | | | |  |  |  |
| Hangai 2009 & Owen 2021 | IVD nucleus-annulus signal intensity ratio | | L1-L2 | | | | 3.88 | | | 0.87 | | | | 251 | | | 3.78 | | | 0.87 | | | | 71 | | | | 2021 report Table 2, all sports pooled except baseball | | | | | | | | | | | | |  |  |  |
| Hangai 2009 & Owen 2021 | IVD nucleus-annulus signal intensity ratio | | L2-L3 | | | | 4.51 | | | 0.96 | | | | 251 | | | 4.4 | | | 0.98 | | | | 71 | | | | 2021 report Table 2, all sports pooled except baseball | | | | | | | | | | | | |  |  |  |
| Hangai 2009 & Owen 2021 | IVD nucleus-annulus signal intensity ratio | | L3-L4 | | | | 4.99 | | | 1.04 | | | | 251 | | | 4.96 | | | 1.09 | | | | 71 | | | | 2021 report Table 2, all sports pooled except baseball | | | | | | | | | | | | |  |  |  |
| Hangai 2009 & Owen 2021 | IVD nucleus-annulus signal intensity ratio | | L4-L5 | | | | 4.25 | | | 1.26 | | | | 251 | | | 4.34 | | | 1.33 | | | | 71 | | | | 2021 report Table 2, all sports pooled except baseball | | | | | | | | | | | | |  |  |  |
| Hangai 2009 & Owen 2021 | IVD nucleus-annulus signal intensity ratio | | L5-S1 | | | | 3.46 | | | 1.11 | | | | 251 | | | 3.47 | | | 1.16 | | | | 71 | | | | 2021 report Table 2, all sports pooled except baseball | | | | | | | | | | | | |  |  |  |
| Hangai 2009 & Owen 2021 | IVD height to vertebral body height ratio | | L1-L2 | | | | 0.32 | | | 0.04 | | | | 251 | | | 0.31 | | | 0.04 | | | | 71 | | | | 2021 report Table 2, all sports pooled except baseball | | | | | | | | | | | | |  |  |  |
| Hangai 2009 & Owen 2021 | IVD height to vertebral body height ratio | | L2-L3 | | | | 0.36 | | | 0.05 | | | | 251 | | | 0.33 | | | 0.05 | | | | 71 | | | | 2021 report Table 2, all sports pooled except baseball | | | | | | | | | | | | |  |  |  |
| Hangai 2009 & Owen 2021 | IVD height to vertebral body height ratio | | L3-L4 | | | | 0.41 | | | 0.05 | | | | 251 | | | 0.38 | | | 0.05 | | | | 71 | | | | 2021 report Table 2, all sports pooled except baseball | | | | | | | | | | | | |  |  |  |
| Hangai 2009 & Owen 2021 | IVD height to vertebral body height ratio | | L4-L5 | | | | 0.46 | | | 0.07 | | | | 251 | | | 0.43 | | | 0.07 | | | | 71 | | | | 2021 report Table 2, all sports pooled except baseball | | | | | | | | | | | | |  |  |  |
| Hangai 2009 & Owen 2021 | IVD height to vertebral body height ratio | | L5-S1 | | | | 0.43 | | | 0.07 | | | | 251 | | | 0.43 | | | 0.08 | | | | 71 | | | | 2021 report Table 2, all sports pooled except baseball | | | | | | | | | | | | |  |  |  |
| **Hangai 2009 & Owen 2021** | **Synthetic effect size: ρ \| Hedges’ g \| standard error** | | | | | | | | | | | | | | | | | | | | | | | | | | | | | | | | | | | | | | | |  |  |  |
|  | ρ=0.0 \| 0.21 \| 0.03 | | | | | | | | | | | | | | | | | | | | | | |  | | | | | | | | | | | | |  | | | |  |  |  |
|  | ρ=0.2 \| 0.21 \| 0.03 | |  | | | |  | | |  | | | |  | | |  | | |  | | | |  | | | |  | | | | | | | | | | | | |  |  |  |
|  | ρ=0.4 \| 0.21 \| 0.03 | |  | | | |  | | |  | | | |  | | |  | | |  | | | |  | | | |  | | | | | | | | | | | | |  |  |  |
|  | ρ=0.6 \| 0.21 \| 0.03 | |  | | | |  | | |  | | | |  | | |  | | |  | | | |  | | | |  | | | | | | | | | | | | |  |  |  |
|  | ρ=0.8 \| 0.21 \| 0.03 | |  | | | |  | | |  | | | |  | | |  | | |  | | | |  | | | |  | | | | | | | | | | | | |  |  |  |
|  | ρ=1.0 \| 0.21 \| 0.03 | |  | | | |  | | |  | | | |  | | |  | | |  | | | |  | | | |  | | | | | | | | | | | | |  |  |  |
| Teichtahl 2015 | IVD height average L1-S1 | | L5-S1 | | | | 11 | | | 15.79 | | | | 57 | | | 10.2 | | | 15.88 | | | | 15 | | | | SD Imputed from SE in Table 1, pooled moderately active and active groups | | | | | | | | | | | | |  |  |  |
| **Primary subgroup synthesis: High vs low volume** | | | | | | | | | | | | | | | | | | | | | | | | | | | | | | | | | | | | | | | | |  |  |  |
| Belavy 2017 & 2019 & 2020 | IVD average volume lumbar average (cm3) | | LxAx | | | | 10.1 | | | 3.3 | | | | 25 | | | 10.1 | | | 3.4 | | | | 30 | | | | 2017 report: Table 1, running 20-4k and Running 50k+ only | | | | | | | | | | | | |  |  |  |
| Belavy 2017 & 2019 & 2020 | IVD average area lumbar average | | LxAx | | | | 263 | | | 58.9 | | | | 25 | | | 263.6 | | | 60.8 | | | | 30 | | | | 2017 report: Table 1, running 20-4k and Running 50k+ only | | | | | | | | | | | | |  |  |  |
| Belavy 2017 & 2019 & 2020 | IVD average anteriorposterior width lumbar average | | LxAx | | | | 25.9 | | | 3.4 | | | | 25 | | | 26.4 | | | 3.5 | | | | 30 | | | | 2017 report: Table 1, running 20-4k and Running 50k+ only | | | | | | | | | | | | |  |  |  |
| Belavy 2017 & 2019 & 2020 | IVD average distance lumbar average | | LxAx | | | | 34 | | | 2.8 | | | | 25 | | | 34.1 | | | 2.9 | | | | 30 | | | | 2017 report: Table 1, running 20-4k and Running 50k+ only | | | | | | | | | | | | |  |  |  |
| Belavy 2017 & 2019 & 2020 | IVD height to vertebral body height ratio | | L1-L2 | | | | 0.33 | | | 0.05 | | | | 25 | | | 0.33 | | | 0.04 | | | | 30 | | | | 2017 report: Imputed from Figure 2 (WebPlotDigitizer) Running 20-4k and Running 50k+ only | | | | | | | | | | | | |  |  |  |
| Belavy 2017 & 2019 & 2020 | IVD height to vertebral body height ratio | | L2-L3 | | | | 0.39 | | | 0.09 | | | | 25 | | | 0.36 | | | 0.05 | | | | 30 | | | | 2017 report: Imputed from Figure 2 (WebPlotDigitizer) Running 20-4k and Running 50k+ only | | | | | | | | | | | | |  |  |  |
| Belavy 2017 & 2019 & 2020 | IVD height to vertebral body height ratio | | L3-L4 | | | | 0.36 | | | 0.11 | | | | 25 | | | 0.31 | | | 0.07 | | | | 30 | | | | 2017 report: Imputed from Figure 2 (WebPlotDigitizer) Running 20-4k and Running 50k+ only | | | | | | | | | | | | |  |  |  |
| Belavy 2017 & 2019 & 2020 | IVD height to vertebral body height ratio, | | L4-L5 | | | | 0.23 | | | 0.06 | | | | 25 | | | 0.22 | | | 0.04 | | | | 30 | | | | 2017 report: Imputed from Figure 2 (WebPlotDigitizer) Running 20-4k and Running 50k+ only | | | | | | | | | | | | |  |  |  |
| Belavy 2017 & 2019 & 2020 | IVD height to vertebral body height ratio | | L5-S1 | | | | 0.24 | | | 0.04 | | | | 25 | | | 0.23 | | | 0.05 | | | | 30 | | | | 2017 report: Imputed from Figure 2 (WebPlotDigitizer) Running 20-4k and Running 50k+ only | | | | | | | | | | | | |  |  |  |
| Belavy 2017 & 2019 & 2020 | IVD T2-time | | L1-L2 | | | | 120.58 | | | 19.24 | | | | 25 | | | 116.8 | | | 20.46 | | | | 30 | | | | 2017 report: Imputed from Figure 2 (WebPlotDigitizer) Running 20-4k and Running 50k+ only | | | | | | | | | | | | |  |  |  |
| Belavy 2017 & 2019 & 2020 | IVD T2- time, | | L2-L3 | | | | 118.01 | | | 18.17 | | | | 25 | | | 118.6 | | | 16.13 | | | | 30 | | | | 2017 report: Imputed from Figure 2 (WebPlotDigitizer) Running 20-4k and Running 50k + only | | | | | | | | | | | | |  |  |  |
| Belavy 2017 & 2019 & 2020 | IVD T2- time, | | L3-L4 | | | | 117.39 | | | 17.72 | | | | 25 | | | 115.7 | | | 16.14 | | | | 30 | | | | 2017 report: Imputed from Figure 2 (WebPlotDigitizer) Running 20-4k and Running 50k+ only | | | | | | | | | | | | |  |  |  |
| Belavy 2017 & 2019 & 2020 | IVD T2- time | | L4-L5 | | | | 112.63 | | | 21.34 | | | | 25 | | | 108.8 | | | 16.33 | | | | 30 | | | | 2017 report: Imputed from Figure 2 (WebPlotDigitizer) Running 20-4k and Running 50k+ only | | | | | | | | | | | | |  |  |  |
| Belavy 2017 & 2019 & 2020 | IVD T2- time | | L5-S1 | | | | 109.22 | | | 21.77 | | | | 25 | | | 107.1 | | | 17.5 | | | | 30 | | | | 2017 report: Imputed from Figure 2 (WebPlotDigitizer) Running 20-4k and Running 50k+ only | | | | | | | | | | | | |  |  |  |
| **Belavy 2017 & 2019 & 2020** | **Synthetic effect size: ρ \| Hedges’ g \| standard error** | | | | | | | | | | | | | | | | | | | | | | | | | | | | | | | | | | | | | | | |  |  |  |
|  | ρ=0.0 \| 0.13 \| 0.10 | | | | | | | | | | | | | | | | | | | | | | | | | | |  | | |  | | | | | | | | | |  |  |  |
|  | ρ=0.2 \| 0.13 \| 0.11 | |  | | | |  | | |  | | | |  | | |  | | |  | | | |  | | | |  | | | | | | | | | | | | |  |  |  |
|  | ρ=0.4 \| 0.13 \| 0.11 | |  | | | |  | | |  | | | |  | | |  | | |  | | | |  | | | |  | | | | | | | | | | | | |  |  |  |
|  | ρ=0.6 \| 0.13 \| 0.12 | |  | | | |  | | |  | | | |  | | |  | | |  | | | |  | | | |  | | | | | | | | | | | | |  |  |  |
|  | ρ=0.8 \| 0.13 \| 0.12 | |  | | | |  | | |  | | | |  | | |  | | |  | | | |  | | | |  | | | | | | | | | | | | |  |  |  |
|  | ρ=1.0 \| 0.13 \| 0.13 | |  | | | |  | | |  | | | |  | | |  | | |  | | | |  | | | |  | | | | | | | | | | | | |  |  |  |
| Lagerstrand 2021 & Rosenqvist 2023 | IVD T2 value subregion 1 | | Lx/Tx Ax | | | | 0.09 | | | 0.05 | | | | 10 | | | 0.09 | | | 0.05 | | | | 7 | | | | 2023 report: Imputed from Figure 3 follow-up time point (WebPlotDigitizer) | | | | | | | | | | | | |  |  |  |
| Lagerstrand 2021 & Rosenqvist 2023 | IVD T2 value subregion 2 | | Lx/Tx Ax | | | | 0.15 | | | 0.08 | | | | 10 | | | 0.15 | | | 0.08 | | | | 7 | | | | 2023 report: Imputed from Figure 3 follow-up time point (WebPlotDigitizer) | | | | | | | | | | | | |  |  |  |
| Lagerstrand 2021 & Rosenqvist 2023 | IVD T2 value subregion 3 | | Lx/Tx Ax | | | | 0.2 | | | 0.12 | | | | 10 | | | 0.2 | | | 0.09 | | | | 7 | | | | 2023 report: Imputed from Figure 3 follow-up time point (WebPlotDigitizer) | | | | | | | | | | | | |  |  |  |
| Lagerstrand 2021 & Rosenqvist 2023 | IVD T2 value subregion 4 | | Lx/Tx Ax | | | | 0.17 | | | 0.11 | | | | 10 | | | 0.18 | | | 0.09 | | | | 7 | | | | 2023 report: Imputed from Figure 3 follow-up time point (WebPlotDigitizer) | | | | | | | | | | | | |  |  |  |
| Lagerstrand 2021 & Rosenqvist 2023 | IVD T2 value subregion 5 | | Lx/Tx Ax | | | | 0.08 | | | 0.06 | | | | 10 | | | 0.09 | | | 0.06 | | | | 7 | | | | 2023 report: Imputed from Figure 3 follow-up time point (WebPlotDigitizer) | | | | | | | | | | | | |  |  |  |
| **Lagerstrand 2021 & Rosenqvist 2023** | **Synthetic effect size: ρ \| Hedges’ g \| standard error** | | | | | | | | | | | | | | | | | | | | | | | | | | | | | | | | | | | | | | | |  |  |  |
|  | ρ=0.0 \| -0.05 \| 0.22 | | | | | | | | | | | | |  | | | | | | | | | | | | | |  | | |  | | | | | | | | | |  |  |  |
|  | ρ=0.2 \| -0.05 \| 0.22 | |  | | | |  | | |  | | | |  | | |  | | |  | | | |  | | | |  | | | | | | | | | | | | |  |  |  |
|  | ρ=0.4 \| -0.05 \| 0.22 | |  | | | |  | | |  | | | |  | | |  | | |  | | | |  | | | |  | | | | | | | | | | | | |  |  |  |
|  | ρ=0.6 \| -0.05 \| 0.22 | |  | | | |  | | |  | | | |  | | |  | | |  | | | |  | | | |  | | | | | | | | | | | | |  |  |  |
|  | ρ=0.8 \| -0.05 \| 0.22 | |  | | | |  | | |  | | | |  | | |  | | |  | | | |  | | | |  | | | | | | | | | | | | |  |  |  |
|  | ρ=1.0 \| -0.05 \| 0.23 | |  | | | |  | | |  | | | |  | | |  | | |  | | | |  | | | |  | | | | | | | | | | | | |  |  |  |
| Teichtahl 2015 | IVD height average | | LxAx | | | | 11.8 | | | 15.69 | | | | 19 | | | 10.6 | | | 16.03 | | | | 38 | | | | Imputed SD from SE in Table 1, moderately active and active groups only | | | | | | | | | | | | |  |  |  |
| Zhang 2023 | Nucleus pulposus T2 time | | L1-L2 | | | | 103.00 | | | 15.57 | | | | 9 | | | 89.86 | | | 13.68 | | | | 18 | | | | Imputed from Figure 4 (WebPlotDigitizer), elite and amateur only | | | | | | | | | | | | |  |  |  |
| Zhang 2023 | Nucleus pulposus T2 time | | L2-L3 | | | | 105.21 | | | 35.66 | | | | 9 | | | 108.2 | | | 19.44 | | | | 18 | | | | Imputed from Figure 4 (WebPlotDigitizer), elite and amateur only | | | | | | | | | | | | |  |  |  |
| Zhang 2023 | Nucleus pulposus T2 time | | L3-L4 | | | | 136.37 | | | 29.19 | | | | 9 | | | 122.9 | | | 23.15 | | | | 18 | | | | Imputed from Figure 4 (WebPlotDigitizer), elite and amateur only | | | | | | | | | | | | |  |  |  |
| Zhang 2023 | Nucleus pulposus T2 time | | L4-L5 | | | | 104.51 | | | 32.63 | | | | 9 | | | 120.5 | | | 16.1 | | | | 18 | | | | Imputed from Figure 4 (WebPlotDigitizer), elite and amateur only | | | | | | | | | | | | |  |  |  |
| Zhang 2023 | Nucleus pulposus T2 time | | L5-S1 | | | | 115.9 | | | 31.81 | | | | 9 | | | 100.7 | | | 22.65 | | | | 18 | | | | Imputed from Figure 4 (WebPlotDigitizer), elite and amateur only | | | | | | | | | | | | |  |  |  |
| **Zhang 2023** | **Synthetic effect size: ρ \| Hedges’ g \| standard error** | | | | | | | | | | | | | | | | | | | | | | | | | | | | | | | | | | | | | | | |  |  |  |
|  | ρ=0.0 \| 0.24 \| 0.19 | | | | | | | | | | | | | | | | | | | | | | | | | | |  | | |  | | | | | | | | | |  |  |  |
|  | ρ=0.2 \| 0.24 \| 0.19 | |  | | | |  | | |  | | | |  | | |  | | |  | | | |  | | | |  | | |  | | | | | | | | | |  |  |  |
|  | ρ=0.4 \| 0.24 \| 0.19 | |  | | | |  | | |  | | | |  | | |  | | |  | | | |  | | | |  | | |  | | | | | | | | | |  |  |  |
|  | ρ=0.6 \| 0.24 \| 0.19 | |  | | | |  | | |  | | | |  | | |  | | |  | | | |  | | | |  | | |  | | | | | | | | | |  |  |  |
|  | ρ=0.8 \| 0.24 \| 0.19 | |  | | | |  | | |  | | | |  | | |  | | |  | | | |  | | | |  | | |  | | | | | | | | | |  |  |  |
|  | ρ=1.0 \| 0.24 \| 0.19 | |  | | | |  | | |  | | | |  | | |  | | |  | | | |  | | | |  | | |  | | | | | | | | | |  |  |  |
| **Secondary synthesis: Average Pfirrmann grade, cross-sectional** | | | | | | | | | | | | | | | | | | | | | | | | | | | | | | | | | | | | | | | | |  |  |  |
| Belavy 2017 & 2019 | Average Pfirrmann grade | | LxAx | | | | 2.1623288 | | | 0.3685049 | | | | 73 | | | 2.3 | | | 0.38944 | | | | 42 | | | | Averaged from 2 reports 2017: Table 1 and 2019: Table 2 | | | | | | | | | | | | |  |  |  |
| Benedikter 2022 | Average Pfirrmann grade | | L2-L3 | | | | 1.7 | | | 0.78 | | | | 20 | | | 1.3 | | | 0.56 | | | | 37 | | | | Imputed from Figure 4 (WebPlotDigitizer), average Pfirrmann calculated from raw data of frequency of all Pfirrmann grades using Excel AVERAGE | | | | | | | | | | | | |  |  |  |
| Benedikter 2022 | Average Pfirrmann grade | | L3-L4 | | | | 1.8 | | | 0.98 | | | | 20 | | | 1.24 | | | 0.98 | | | | 37 | | | | Imputed from Figure 4 (WebPlotDigitizer), average Pfirrmann calculated from raw data of frequency of all Pfirrmann grades using Excel AVERAGE | | | | | | | | | | | | |  |  |  |
| Benedikter 2022 | Average Pfirrmann grade | | L4-L5 | | | | 1.65 | | | 0.85 | | | | 20 | | | 1.16 | | | 0.44 | | | | 37 | | | | Imputed from Figure 4 (WebPlotDigitizer), average Pfirrmann calculated from raw data of frequency of all Pfirrmann grades using Excel AVERAGE | | | | | | | | | | | | |  |  |  |
| Benedikter 2022 | Average Pfirrmann grade | | L5-S1 | | | | 2 | | | 1.1 | | | | 20 | | | 1.62 | | | 0.71 | | | | 37 | | | | Imputed from Figure 4 (WebPlotDigitizer), average Pfirrmann calculated from raw data of frequency of all Pfirrmann grades using Excel AVERAGE | | | | | | | | | | | | |  |  |  |
| **Benedikter 2022** | **Synthetic effect size: ρ \| Hedges’ g \| standard error** | | | | | | | | | | | | | | | | | | | | | | | | | | | | | | | | | | | | | | | |  |  |  |
|  | ρ=0.0 \| 0.60 \| 0.14 | | | | | | | | | | | | | | | | | | | | | | | | | | |  | | |  | | | | | | | | | |  |  |  |
|  | ρ=0.2 \| 0.60 \| 0.14 | |  | | | |  | | |  | | | |  | | |  | | |  | | | |  | | | |  | | | | | | | | | | | | |  |  |  |
|  | ρ=0.4 \| 0.60 \| 0.14 | |  | | | |  | | |  | | | |  | | |  | | |  | | | |  | | | |  | | | | | | | | | | | | |  |  |  |
|  | ρ=0.6 \| 0.60 \| 0.14 | |  | | | |  | | |  | | | |  | | |  | | |  | | | |  | | | |  | | | | | | | | | | | | |  |  |  |
|  | ρ=0.8 \| 0.60 \| 0.14 | |  | | | |  | | |  | | | |  | | |  | | |  | | | |  | | | |  | | | | | | | | | | | | |  |  |  |
|  | ρ=1.0 \| 0.60 \| 0.14 | |  | | | |  | | |  | | | |  | | |  | | |  | | | |  | | | |  | | | | | | | | | | | | |  |  |  |
| Cheng 2008 | Average Pfirrmann grade | | L1-L2 | | | | 2.25 | | | 0.27 | | | | 18 | | | 2.25 | | | 0.27 | | | | 18 | | | | Table 1, data imputed from median range to mean SD | | | | | | | | | | | | |  |  |  |
| Cheng 2008 | Average Pfirrmann grade | | L2-L3 | | | | 2.25 | | | 0.27 | | | | 18 | | | 2.75 | | | 0.55 | | | | 18 | | | | Table 1, data imputed from median range to mean SD | | | | | | | | | | | | |  |  |  |
| Cheng 2008 | Average Pfirrmann grade | | L3-L4 | | | | 2.5 | | | 0.55 | | | | 18 | | | 2.75 | | | 0.82 | | | | 18 | | | | Table 1, data imputed from median range to mean SD | | | | | | | | | | | | |  |  |  |
| Cheng 2008 | Average Pfirrmann grade | | L4-L5 | | | | 2.75 | | | 0.82 | | | | 18 | | | 2.75 | | | 0.82 | | | | 18 | | | | Table 1, data imputed from median range to mean SD | | | | | | | | | | | | |  |  |  |
| Cheng 2008 | Average Pfirrmann grade | | L5-S1 | | | | 2.25 | | | 0.82 | | | | 18 | | | 2.75 | | | 0.82 | | | | 18 | | | | Table 1, data imputed from median range to mean SD | | | | | | | | | | | | |  |  |  |
| **Cheng 2008** | **Synthetic effect size: ρ \| Hedges’ g \| standard error** | | | | | | | | | | | | | | | | | | | | | | | | | | | | | | | | | | | | | | | |  |  |  |
|  | ρ=0.0 \| -0.37 \| 0.17 | | | | | | | | | | | | | | | | | | | | | | | | | | |  | | |  | | | | | | | | | |  |  |  |
|  | ρ=0.2 \| -0.37 \| 0.17 | |  | | | |  | | |  | | | |  | | |  | | |  | | | |  | | | |  | | | | | | | | | | | | |  |  |  |
|  | ρ=0.4 \| -0.37 \| 0.17 | |  | | | |  | | |  | | | |  | | |  | | |  | | | |  | | | |  | | | | | | | | | | | | |  |  |  |
|  | ρ=0.6 \| -0.37 \| 0.17 | |  | | | |  | | |  | | | |  | | |  | | |  | | | |  | | | |  | | | | | | | | | | | | |  |  |  |
|  | ρ=0.8 \| -0.37 \| 0.17 | |  | | | |  | | |  | | | |  | | |  | | |  | | | |  | | | |  | | | | | | | | | | | | |  |  |  |
|  | ρ=1.0 \| -0.37 \| 0.18 | |  | | | |  | | |  | | | |  | | |  | | |  | | | |  | | | |  | | | | | | | | | | | | |  |  |  |
| Maurer 2020 | Pfirmmann grade, whole spine | | Lx/ Tx Ax | | | | 3.61 | | | 5.51 | | | | 288 | | | 5.86 | | | 5.52 | | | | 97 | | | | Imputed from Figure 4 (WebPlotDigitizer), 95%CI imputed to SD | | | | | | | | | | | | |  |  |  |
| Mitchell 2020 | Average Pfirrmann grade | | L2-L3 | | | | 1.7 | | | 0.5 | | | | 9 | | | 2.3 | | | 0.7 | | | | 8 | | | | Table 4 | | | | | | | | | | | | |  |  |  |
| Mitchell 2020 | Average Pfirrmann grade | | L3-L4 | | | | 1.9 | | | 0.6 | | | | 9 | | | 2.3 | | | 0.5 | | | | 8 | | | | Table 4 | | | | | | | | | | | | |  |  |  |
| Mitchell 2020 | Average Pfirrmann grade | | L4-L5 | | | | 2.1 | | | 0.8 | | | | 9 | | | 2.3 | | | 1 | | | | 8 | | | | Table 4 | | | | | | | | | | | | |  |  |  |
| Mitchell 2020 | Average Pfirrmann grade | | L5-S1 | | | | 2.2 | | | 0.7 | | | | 9 | | | 3.3 | | | 1 | | | | 8 | | | | Table 4 | | | | | | | | | | | | |  |  |  |
| **Mitchell 2020** | **Synthetic effect size: ρ \| Hedges’ g \| standard error** | | | | | | | | | | | | | | | | | | | | | | | | | | | | | | | | | | | | | | | |  |  |  |
|  | ρ=0.0 \| -0.77 \| 0.26 | | | | | | | | | | | | | | | | | | | | | | | | | | |  | | |  | | | | | | | | | |  |  |  |
|  | ρ=0.2 \| -0.77 \| 0.26 | |  | | | |  | | |  | | | |  | | |  | | |  | | | |  | | | |  | | | | | | | | | | | | |  |  |  |
|  | ρ=0.4 \| -0.77 \| 0.26 | |  | | | |  | | |  | | | |  | | |  | | |  | | | |  | | | |  | | | | | | | | | | | | |  |  |  |
|  | ρ=0.6 \| -0.77 \| 0.26 | |  | | | |  | | |  | | | |  | | |  | | |  | | | |  | | | |  | | | | | | | | | | | | |  |  |  |
|  | ρ=0.8 \| -0.77 \| 0.27 | |  | | | |  | | |  | | | |  | | |  | | |  | | | |  | | | |  | | | | | | | | | | | | |  |  |  |
|  | ρ=1.0 \| -0.77 \| 0.27 | |  | | | |  | | |  | | | |  | | |  | | |  | | | |  | | | |  | | | | | | | | | | | | |  |  |  |
| Wegner 2023 | Average Pfirrmann grade | | L1-L2 | | | | 2 | | | 0.84 | | | | 9 | | | 2 | | | 0.84 | | | | 10 | | | | Table 6. Median used as mean, Imputed SD from assumed variance for average Pfirrmann grade | | | | | | | | | | | | |  |  |  |
| Wegner 2023 | Average Pfirrmann grade | | L2-L3 | | | | 2 | | | 0.84 | | | | 9 | | | 2 | | | 0.84 | | | | 10 | | | | Table 6. Median used as mean, Imputed SD from assumed variance for average Pfirrmann grade | | | | | | | | | | | | |  |  |  |
| Wegner 2023 | Average Pfirrmann grade | | L3-L4 | | | | 2 | | | 0.84 | | | | 9 | | | 2 | | | 0.84 | | | | 10 | | | | Table 6. Median used as mean, Imputed SD from assumed variance for average Pfirrmann grade | | | | | | | | | | | | |  |  |  |
| Wegner 2023 | Average Pfirrmann grade | | L4-L5 | | | | 2 | | | 0.84 | | | | 9 | | | 2 | | | 0.84 | | | | 10 | | | | Table 6. Median used as mean, Imputed SD from assumed variance for average Pfirrmann grade | | | | | | | | | | | | |  |  |  |
| Wegner 2023 | Average Pfirrmann grade | | L5-S1 | | | | 2 | | | 0.84 | | | | 9 | | | 2 | | | 0.84 | | | | 10 | | | | Table 6. Median used as mean, Imputed SD from assumed variance for average Pfirrmann grade | | | | | | | | | | | | |  |  |  |
| **Wegner 2023** | **Synthetic effect size: ρ \| Hedges’ g \| standard error** | | | | | | | | | | | | | | | | | | | | | | | | | | | | | | | | | | | | | | | |  |  |  |
|  | ρ=0.0 \| 0.00 \| 0.21 | | | | | | | | | | | | | | | |  | | | | | | | | | | |  | | |  | | | | | | | | | |  |  |  |
|  | ρ=0.2 \| 0.00 \| 0.21 | |  | | | |  | | |  | | | |  | | |  | | |  | | | |  | | | |  | | |  | | | | | | | | | |  |  |  |
|  | ρ=0.4 \| 0.00 \| 0.21 | |  | | | |  | | |  | | | |  | | |  | | |  | | | |  | | | |  | | |  | | | | | | | | | |  |  |  |
|  | ρ=0.6 \| 0.00 \| 0.21 | |  | | | |  | | |  | | | |  | | |  | | |  | | | |  | | | |  | | |  | | | | | | | | | |  |  |  |
|  | ρ=0.8 \| 0.00 \| 0.21 | |  | | | |  | | |  | | | |  | | |  | | |  | | | |  | | | |  | | |  | | | | | | | | | |  |  |  |
|  | ρ=1.0 \| 0.00 \| 0.21 | |  | | | |  | | |  | | | |  | | |  | | |  | | | |  | | | |  | | |  | | | | | | | | | |  |  |  |
| **Secondary synthesis: Average Pfirrmann grade, RCT/cohort studies with baseline as control. Mean change from baseline to follow-up.** | | | | | | | | | | | | | | | | | | | | | | | | | | | | | | | | | | | | | | | | |  |  |  |
| Horga 2022 | Average Pfirrmann grade | | L3-L4 | | | | 1.46 | | | 0.84 | | | | 21 | | | 1.46 | | | | 0.84 | | | | 28 | | | Table 3. Imputed SD from assumed variance for average Pfirrmann grade | | | | | | | | | | | | |  |  |  |
| Horga 2022 | Average Pfirrmann grade | | L4-L5 | | | | 1.62 | | | 0.84 | | | | 21 | | | 1.62 | | | | 0.84 | | | | 28 | | | Table 3. Imputed SD from assumed variance for average Pfirrmann grade | | | | | | | | | | | | |  |  |  |
| Horga 2022 | Average Pfirrmann grade | | L5-S1 | | | | 1.86 | | | 0.84 | | | | 21 | | | 1.86 | | | | 0.84 | | | | 28 | | | Table 3. Imputed SD from assumed variance for average Pfirrmann grade | | | | | | | | | | | | |  |  |  |
| **Horga 2022** | **Synthetic effect size: ρ \| Hedges’ g \| standard error** | | | | | | | | | | | | | | | | | | | | | | | | | | | | | | | | | | | | | | | |  |  |  |
|  | ρ=0.0 \| 0.00 \| 0.17 | | | | | | | | |  | | | | | | | | | | | | | | | | | | | | |  | | | | | | | | | |  |  |  |
|  | ρ=0.2 \| 0.00 \| 0.17 | |  | | | |  | | |  | | | |  | | |  | | | |  | | | |  | | |  | | | | | | | | | | | | |  |  |  |
|  | ρ=0.4 \| 0.00 \| 0.17 | |  | | | |  | | |  | | | |  | | |  | | | |  | | | |  | | |  | | | | | | | | | | | | |  |  |  |
|  | ρ=0.6 \| 0.00 \| 0.18 | |  | | | |  | | |  | | | |  | | |  | | | |  | | | |  | | |  | | | | | | | | | | | | |  |  |  |
|  | ρ=0.8 \| 0.00 \| 0.18 | |  | | | |  | | |  | | | |  | | |  | | | |  | | | |  | | |  | | | | | | | | | | | | |  |  |  |
|  | ρ=1.0 \| 0.00 \| 0.18 | |  | | | |  | | |  | | | |  | | |  | | | |  | | | |  | | |  | | | | | | | | | | | | |  |  |  |
| Shimozaki 2018 | Average Pfirrmann grade | | L1-L2 | | | | 1.33 | | | 0.62 | | | | 12 | | | 1.083 | | | | 0.28 | | | | 12 | | | Table 3 and Table 5, average Pfirrmann calculated from raw data of frequency of all Pfirrmann grades using Excel AVERAGE, SD imputed from assumed variance for average Pfirrmann grade | | | | | | | | | | | | |  |  |  |
| Shimozaki 2018 | Average Pfirrmann grade | | L2-L3 | | | | 1.17 | | | 0.37 | | | | 12 | | | 1 | | | | 0.84 | | | | 12 | | | Table 3 and Table 5, average Pfirrmann calculated from raw data of frequency of all Pfirrmann grades using Excel AVERAGE, SD imputed from assumed variance for average Pfirrmann grade | | | | | | | | | | | | |  |  |  |
| Shimozaki 2018 | Average Pfirrmann grade | | L3-L4 | | | | 1.08 | | | 0.28 | | | | 12 | | | 1 | | | | 0.84 | | | | 12 | | | Table 3 and Table 5, average Pfirrmann calculated from raw data of frequency of all Pfirrmann grades using Excel AVERAGE, SD imputed from assumed variance for average Pfirrmann grade | | | | | | | | | | | | |  |  |  |
| Shimozaki 2018 | Average Pfirrmann grade | | L4-L5 | | | | 1.83 | | | 0.69 | | | | 12 | | | 1.08 | | | | 0.28 | | | | 12 | | | Table 3 and Table 5, average Pfirrmann calculated from raw data of frequency of all Pfirrmann grades using Excel AVERAGE, SD imputed from assumed variance for average Pfirrmann grade | | | | | | | | | | | | |  |  |  |
| Shimozaki 2018 | Average Pfirrmann grade | | L5-S1 | | | | 1.25 | | | 0.6 | | | | 12 | | | 1 | | | | 0.84 | | | | 12 | | | Table 3 and Table 5, average Pfirrmann calculated from raw data of frequency of all Pfirrmann grades using Excel AVERAGE, SD imputed from assumed variance for average Pfirrmann grade | | | | | | | | | | | | |  |  |  |
| **Shimozaki 2018** | **Synthetic effect size: ρ \| Hedges’ g \| standard error** | | | | | | | | | | | | | | | | | | | | | | | | | | | | | | | | | | | | | | | |  |  |  |
|  | ρ=0.0 \| 0.52 \| 0.19 | | | | | | | | | | | | | | | | | | | |  | | | |  | | |  | | | | | | | | | | | | |  |  |  |
|  | | ρ=0.2 \| 0.52 \| 0.19 | |  | |  | | | | | | |  | | |  | | | | | | | | | | | | | | | | | | | | | | | | |  |  |  |
|  | | ρ=0.4 \| 0.52 \| 0.19 | |  | |  | | | | | | |  | | |  | | | | | | | | | | | | | | | | | | | | | | | | |  |  |  |
|  | | ρ=0.6 \| 0.52 \| 0.19 | |  | |  | | | | | | |  | | |  | | | | | | | | | | | | | | | | | | | | | | | | |  |  |  |
|  | | ρ=0.8 \| 0.52 \| 0.19 | |  | |  | | | | | | |  | | |  | | | | | | | | | | | | | | | | | | | | | | | | |  |  |  |
|  | | ρ=1.0 \| 0.52 \| 0.19 | |  | |  | | | | | | |  | | |  | | | | | | | | | | | | | | | | | | | | | | | | |  |  |  |
| Telles 2016 | Average Pfirrmann grade | | LxAx | | | | 1.57 | | | 0.49 | | | | 14 | | | 1.7 | | | | 0.59 | | | | 20 | | | Table 2, average Pfirrmann calculated from raw data of frequency of all Pfirrmann grades using Excel AVERAGE | | | | | | | | | | | | |  |  |  |

|  |  |  |  | | **Physical loading group** | | | **Control group** | |  |
| --- | --- | --- | --- | --- | --- | --- | --- | --- | --- | --- |
|  | **Secondary synthesis: Binary Pfirrmann grade >2, cross-sectional** | | **Level** | **Yes** | | **No** | **Yes** | | **No** | **Data source** |
|  | Benedikter 2022 | N of participants with Pfirrmann grade >2 | L2L3-L5S1 | 21 | | 59 | 11 | | 137 | Table 5, binary degeneration prevalence calculated from raw data of frequency of all Pfirrmann grades, Grades 1 & 2= no and Grades 3, 4 & 5= yes |
|  | Cheng 2008 | N of participants with Pfirrmann grade >2 | LxAx | 36 | | 54 | 54 | | 36 | Figure 2, binary degeneration prevalence calculated from raw data of frequency of all Pfirrmann grades, Grades 1 & 2= no and Grades 3, 4 & 5= yes |
|  | Folkvardsen 2016 | N of participants with Pfirrmann grade >2 | LxAx | 97 | | 403 | 78 | | 402 | Table 3 |
|  | Hangai 2009 & Owen 2021 | N of participants with Pfirrmann grade >2 | L2L3-L5S1 | 163 | | 1069 | 24 | | 260 | 2009 report: Table 2 |
|  | Identeg 2023 | N of participants with Pfirrmann grade >2 | LxAx | 6 | | 9 | 10 | | 5 | Table 5 |
|  | Kaneoka 2007 | N of participants with Pfirrmann grade >2 | LxAx | 59 | | 221 | 16 | | 174 | Table 2 |
|  | Koyama 2022 | N of participants with Pfirrmann grade >2 | LxAx | 175 | | 1100 | 24 | | 191 | Table 5 |
|  | Kraft 2009 | N of participants with Pfirrmann grade >2 | L3L4-L5S1 | 34 | | 24 | 18 | | 12 | Table 3 |
|  | Ranson 2005 | N of participants with Pfirrmann grade >2 | LxAx | 18 | | 18 | 8 | | 9 | Results section pg.1114 |
|  | Witwit 2018 & 2022 | N of participants with Pfirrmann grade >2 | T6T7-L5S1 | 36 | | 39 | 8 | | 19 | 2018 report: Table 3 |
|  | **Secondary synthesis: Binary Pfirrmann grade >2, RCT/cohort studies with baseline as control. Mean change from baseline to follow-up.** | | | | | | | | | |
|  | Burnett 1996 | N of participants with Pfirrmann grade >2 | LxAx | 15 | | 80 | 5 | | 90 | Table 1 |
|  | Koyama 2022 | N of participants with Pfirrmann grade >2 | LxAx | 24 | | 27 | 23 | | 28 | Table 5 |
|  | Shimozaki 2018 | N of participants with Pfirrmann grade >2 | LxAx | 4 | | 56 | 0 | | 60 | Table 3 and Table 5, binary degeneration prevalence calculated from raw data of frequency of all Pfirrmann grades, Grades 1 & 2= no and Grades 3, 4 & 5= yes |
|  | Telles 2016 | N of participants with Pfirrmann grade >2 | LxAx | 0 | | 14 | 7 | | 13 | Table 2, binary degeneration prevalence calculated from raw data of frequency of all Pfirrmann grades, Grades 1 & 2= no and Grades 3, 4 & 5= yes |
|  | Witwit 2018 & 2022 | N of participants with Pfirrmann grade >2 | T5T6 -L5S1 | 19 | | 11 | 16 | | 14 | 2022:report Table 1 |
|  | **Secondary synthesis: Binary Pfirrmann grade >2, RCT/cohort studies with separate control. Mean change from control and intervention at follow-up time point.** | | | | | | | | | |
|  | Telles 2016 | N of participants with Pfirrmann grade >2 | LxAx | 0 | | 14 | 0 | | 10 | Table 2, binary degeneration calculated from raw data of frequency of all Pfirrmann grades, Grades 1 & 2= no and Grades 3, 4 & 5= yes |
|  | Witwit 2018 & 2022 | N of participants with Pfirrmann grade >2 | LxAx | 19 | | 11 | 4 | | 12 | 2022 report: Table 1 |
|  | **Secondary synthesis: IVD signal reduction >1 (Scale:0=normal, 1=mod reduction, 2=severe reduction), cross-sectional** | | | | | | | | | |
|  | Baranto 2009 | N of participants with IVD signal reduction ≥ 1 | LxAx | 35 | | 1 | 10 | | 0 | Table 1 |
|  | Hellstrom 1990 & Sward 1991 | N of participants with IVD signal reduction ≥ 1 | T6T7-L5S1 | 18 | | 6 | 5 | | 11 | 1990 report:Table 1 |
|  | Maurer 2011 | N of participants with IVD signal reduction ≥ 1 | LxAx | 4 | | 18 | 2 | | 20 | Results section pg.394 |
|  | Thoreson 2017 | N of participants with IVD signal reduction ≥ 1 | Lx/ Tx Ax | 11 | | 5 | 12 | | 16 | Table 5 |
|  | Witwit 2018 & 2022 | N of participants with IVD signal reduction ≥ 1 | T6-T7 to L5-S1 | 45 | | 30 | 11 | | 16 | 2018 report: Table 3 |
| **Secondary synthesis: Any IVD signal reduction, RCT/cohort studies with baseline as control. Mean change from baseline to follow-up.** | | | | | | | | | | |
| Feuerriegel 2025 | | Signal reduced disk | L2/L3 to L5/S1 | 21 | | 42 | 10 | | 53 | Table 4 |
| Witwit 2022 | | IVD signal reduction | T5/T6 -L5/S1 | 20 | | 10 | 20 | | 10 | Table 3 |
| Baranto 2006 | | IVD signal reduction | T12-L1 | 4 | | 15 | 3 | | 13 | Table 4 |
|  | **Secondary synthesis: Any IVD height reduction, cross-sectional** | | | | | | | | | |
|  | Baranto 2009 | IVD height reduction | T6T7-L5S1 | 32 | | 4 | 9 | | 1 | Table 1 |
|  | Thoreson 2017 | IVD height reduction | Lx/ Tx Ax | 8 | | 8 | 0 | | 28 | Table 5 |
|  | Witwit 2018 & 2022 | IVD height reduction | T6T7-L5S1 | 24 | | 51 | 0 | | 27 | 2018 report: Table 3 |
|  | **Secondary synthesis: >50% IVD height reduction, cross-sectional** | | | | | | | | | |
|  | Granhed 1988 | N of participants with >50% IVD height reduction | T6T7-L5S1 | 17 | | 28 | 23 | | 693 | Results section pg.532 |
|  | Hellstrom 1990 & Sward 1991 | N of participants with >50% IVD height reduction | T6T7-L5S1 | 11 | | 122 | 2 | | 28 | 1990 report:Table 1 |

Key: Lx: Lumbar, Tx: thoracic, Ax: average, SD: standard deviation, SE: standard error, IQR: interquartile range,

Formulas used for imputed data:

1. SD imputed from SE: Excel formula =SE*SQRT(N)
2. Median IQR imputed to mean SD: Excel formula: Mean = (Q1+median+Q3)/3, SD= =(Q3-Q1)/(2*NORM.INV((0.75*N-0.125)/(N+0.25),0,1))
3. 95% CI imputed to SD: Excel formula= SQRT(N)*( (95%CI upper)-(95%CI lower))/3.92
4. Assumed variance for imputing SD: In excel, taking an average of all SD values for the same outcome.
5. Data imputed from median and data range to mean SD: Excel formula: Mean= =((minimum+2)* median+ maximum)/4, SD: =(maximum-minimum)/(2*NORM.INV((N-0.375)/(N+0.25),0,1))
6. Pooling groups: Excel formula (2 groups): Pooled mean = ((Mean1*N1) + (Mean2*N2))/Pooled N, Pooled SD: =SQRT((((N1-1)*(SD1^2))+((N2-1)*(SD2^2))+(((N1*N2)/(N1+N2))*((MEAN1^2)+(MEAN2^2)-(2*(MEAN1*MEAN2)))))/(N1+N2-1)), Pooled N=N1 + N2

# Supplement D: Data requests

| **Reports** | **Trial 1** | **Trial 2** | **Trial 3** | **Replies** |  |
| --- | --- | --- | --- | --- | --- |
| Jentzsch 2020 | 18/08/2023 | 25/08/2023 | 1/09/2023 | No reply |  |
| Horga 2022 | 18/08/2023 | 25/08/2023 | - | Replied 26/08/23: data pending  Replied 16/09/23: unable to find the data | |
| Frenken 2022 | 18/08/2023 | 25/08/2023 | 1/09/2023 | No reply |  |
| Belavy 2019 and 2020 | 18/08/2023 | 1/09/2023 | - | Replied 8/9/2023: provided data | |
| Benedikter 2022 | 21/03/2024 | 28/03/2024 | 8/04/2024 | No reply | |
| Hangai 2008 | 28/03/2024 | 8/04/2024 | - | Replied 9-Apr/24: unable to find the data | |
| Capel 2009 | 28/03/2024 | 8/04/2024 | 15/04/2024 | No reply | |
| Tertti 1990 | 28/03/2024 | 8/04/2024 | 15/04/2024 | No reply | |
| Videman 1997 | 28/03/2024 | 8/04/2024 | 15/04/2024 | No reply | |
| Elfering 2002 | 28/03/2024 | 8/04/2024 | 15/04/2024 | Replied 19/04/2024: provided raw baseline data | |

# Supplement E: Statistical code and data

| ***************************************************************************  **#1 Cross-sectional studies, INT: Any exercise, OUTCOME: continuous IVD measure, n=11  ***************************************************************************  //Calculate effect estimates  clear  input str50 study n1 m1 s1 n2 m2 s2  "Belavy 2017, 2019 & 2020" 73 26.25 3.16 24 25.60 2.40  "Belavy 2017, 2019 & 2020" 55 263.33 59.39 24 250.20 41.20  "Belavy 2017, 2019 & 2020" 73 34.31 2.75 24 34.20 2.00  "Belavy 2017, 2019 & 2020" 73 10.30 3.14 24 9.50 2.30  "Belavy 2017, 2019 & 2020" 73 0.30 0.05 24 0.28 0.05  "Belavy 2017, 2019 & 2020" 73 116.24 20.20 24 107.62 6.62  "Belavy 2017, 2019 & 2020" 73 114.99 17.08 24 108.82 11.49  "Belavy 2017, 2019 & 2020" 73 111.19 18.29 24 108.27 12.61  "Belavy 2017, 2019 & 2020" 73 105.65 18.93 24 100.13 16.97  "Belavy 2017, 2019 & 2020" 73 104.91 19.31 24 94.98 20.71  "Belavy 2017, 2019 & 2020" 77 1.49 0.13 24 1.40 0.15  "Belavy 2017, 2019 & 2020" 77 322.00 24.23 24 326.90 24.00  "Belavy 2017, 2019 & 2020" 77 3.35 0.34 24 3.31 0.34  "Belavy 2017, 2019 & 2020" 77 87.42 3.19 24 86.00 2.94  "Belavy 2017, 2019 & 2020" 77 1.01 0.01 24 1.01 0.01  "Benedikter 2022" 17 32.82 30.31 37 37.99 32.71  "Benedikter 2022" 17 35.94 27.94 37 43.42 32.49  "Benedikter 2022" 17 40.31 34.98 37 52.50 39.76  "Benedikter 2022" 17 39.05 40.15 37 46.12 35.09  "Bowden 2018" 14 -0.15 0.06 12 -0.17 0.11  "Bowden 2018" 14 1.15 0.48 12 0.79 0.49  "Bowden 2018" 14 55.80 24.70 12 49.70 21.40  "Frenken 2022" 17 2.58 0.27 44 1.66 0.38  "Jentzsch 2020" 12 85.60 14.90 13 87.20 13.50  "Lagerstrand 2021 & Rosenqvist 2023" 58 0.74 0.33 26 0.76 0.34  "Lagerstrand 2021 & Rosenqvist 2023" 58 1.06 0.35 26 1.14 0.40  "Lagerstrand 2021 & Rosenqvist 2023" 58 1.30 0.48 26 1.33 0.43  "Lagerstrand 2021 & Rosenqvist 2023" 58 1.13 0.44 26 1.12 0.41  "Lagerstrand 2021 & Rosenqvist 2023" 58 0.53 0.28 26 0.47 0.23  "Li 2024" 54 21.54 4.47 30 19.42 3.68  "Li 2024" 54 22.50 5.07 30 21.28 5.09  "Li 2024" 54 21.99 4.72 30 19.35 3.47  "Li 2024" 54 23.30 5.30 30 21.68 4.58  "Li 2024" 54 21.59 5.55 30 19.85 4.51  "Li 2024" 54 42.23 11.27 30 39.95 10.72  "Li 2024" 54 47.51 7.74 30 44.71 13.51  "Li 2024" 54 49.34 13.40 30 47.01 15.79  "Li 2024" 54 51.21 15.94 30 43.30 15.84  "Li 2024" 54 47.66 18.48 30 44.16 17.41  "Mitchell 2020" 9 249.00 175.20 8 202.30 149.50  "Mitchell 2020" 9 0.33 0.04 8 0.23 0.02  "Mitchell 2020" 9 0.32 0.05 8 0.25 0.03  "Mitchell 2020" 9 0.31 0.06 8 0.26 0.04  "Mitchell 2020" 9 0.28 0.05 8 0.22 0.04  "Mitchell 2020" 9 101.20 13.60 8 93.10 26.60  "Mitchell 2020" 9 95.10 13.90 8 89.60 26.80  "Mitchell 2020" 9 83.90 12.20 8 85.70 32.70  "Mitchell 2020" 9 85.10 14.80 8 78.80 14.90  "Hangai 2009 & Owen 2021" 308 0.32 0.04 71 0.31 0.04  "Hangai 2009 & Owen 2021" 308 0.36 0.05 71 0.33 0.05  "Hangai 2009 & Owen 2021" 308 0.41 0.05 71 0.38 0.05  "Hangai 2009 & Owen 2021" 308 0.46 0.07 71 0.43 0.07  "Hangai 2009 & Owen 2021" 308 0.43 0.07 71 0.43 0.08  "Hangai 2009 & Owen 2021" 308 3.83 0.87 71 3.78 0.87  "Hangai 2009 & Owen 2021" 308 4.49 0.96 71 4.40 0.98  "Hangai 2009 & Owen 2021" 308 4.93 1.04 71 4.96 1.09  "Hangai 2009 & Owen 2021" 308 4.19 1.27 71 4.34 1.33  "Hangai 2009 & Owen 2021" 308 3.40 1.12 71 3.47 1.16  "Hangai 2009 & Owen 2021" 308 26.84 3.32 71 25.70 3.30  "Hangai 2009 & Owen 2021" 308 28.63 3.36 71 27.70 3.90  "Hangai 2009 & Owen 2021" 308 29.24 3.27 71 28.70 3.40  "Hangai 2009 & Owen 2021" 308 28.75 3.47 71 27.80 3.30  "Hangai 2009 & Owen 2021" 308 23.90 3.33 71 22.80 3.00  "Teichtahl 2015" 57 11.00 15.79 15 10.20 15.88  "Vadala 2014" 13 102.53 26.91 13 116.07 36.54  "Vadala 2014" 13 99.65 17.77 13 114.90 22.59  "Vadala 2014" 13 94.18 16.53 13 112.16 28.16  "Vadala 2014" 13 77.48 20.51 13 110.69 21.59  "Vadala 2014" 13 79.78 21.76 13 100.74 24.92  "Zhang 2023" 27 110.70 24.92 18 98.30 22.20  end  format %9.2f m1  format %9.2f s1  format %9.2f m2  format %9.2f s2  meta esize n1 m1 s1 n2 m2 s2, studylabel(study) esize(hedges) eslabel(Hedges' g)  encode(study), gen(study2)  gen _meta_var =((n1+n2)/(n1*n2))+((_meta_es^2)/(2*(n1+n2-2)))  //Meta-analysis: ρ=0.8  clear  input str50 study es se  "Belavy 2017, 2019 & 2020" 0.27 0.06  "Benedikter 2022" -0.22 0.15  "Bowden 2018" 0.40 0.25  "Frenken 2022" 2.57 0.37  "Jentzsch 2020" -0.11 0.39  "Lagerstrand 2021 & Rosenqvist 2023" -0.02 0.11  "Li 2024" 0.33 0.07  "Mitchell 2020" 0.88 0.18  "Hangai 2009 & Owen 2021" 0.21 0.03  "Teichtahl 2015" 0.05 0.29  "Vadala 2014" -0.86 0.19  "Zhang 2023" 0.51 0.30  end  format %9.0f es  format %9.2f se  meta set es se, studylabel(study) eslabel(Hedges' g)  meta summarize, predint  meta forestplot, nullrefline(favorsleft("Favours control", al(baseline) height(45)) favorsright("Favours exercise", al(baseline) height(45))) xline(0, lcolor(gs7) lpattern(solid)) title("Any Physical Loading vs Control") xlab(-2(1)3) predint(,pstyle(ci) lc(red))  meta funnelplot, title("Any Physical Loading vs Control") graphregion(color(white))  meta trimfill  meta trimfill, funnel(title("Cross-sectional continuous IVD health, any physical loading vs control") graphregion(color(white)))  meta bias, egger  //Meta-regression: age  clear  input str50 study es se age  "Belavy 2017, 2019 & 2020" 0.27 0.06 29.91  "Benedikter 2022" -0.22 0.15 24.05  "Bowden 2018" 0.40 0.25 44.67  "Frenken 2022" 2.57 0.37 25.25  "Jentzsch 2020" -0.11 0.39 29.58  "Lagerstrand 2021 & Rosenqvist 2023" -0.02 0.11 17.64  "Li 2024" 0.33 0.07  "Mitchell 2020" 0.88 0.18 48.94  "Hangai 2009 & Owen 2021" 0.21 0.03 19.46  "Teichtahl 2015" 0.05 0.29 45.91  "Vadala 2014" -0.86 0.19 24.40  "Zhang 2023" 0.51 0.30 20.97  end  format %9.0f es  format %9.2f se  meta set es se, studylabel(study) eslabel(Hedges' g)  meta regress age  //Meta-regression: sex  clear  input str50 study es se sex  "Belavy 2017, 2019 & 2020" 0.27 0.06 53.47  "Benedikter 2022" -0.22 0.15 47.37  "Bowden 2018" 0.40 0.25 65.38  "Frenken 2022" 2.57 0.37 53.85  "Jentzsch 2020" -0.11 0.39 40.00  "Lagerstrand 2021 & Rosenqvist 2023" -0.02 0.11 53.57  "Li 2024" 0.33 0.07  "Mitchell 2020" 0.88 0.18 0.00  "Hangai 2009 & Owen 2021" 0.21 0.03 23.48  "Teichtahl 2015" 0.05 0.29 68.06  "Vadala 2014" -0.86 0.19 0.00  "Zhang 2023" 0.51 0.30 55.56  end  format %9.0f es  format %9.2f se  meta set es se, studylabel(study) eslabel(Hedges' g)  meta regress sex  //Sensitivity analysis: ρ=0.0  clear  input str50 study es se  "Belavy 2017, 2019 & 2020" 0.27 0.06  "Benedikter 2022" -0.22 0.15  "Bowden 2018" 0.40 0.23  "Frenken 2022" 2.57 0.37  "Jentzsch 2020" -0.11 0.39  "Lagerstrand 2021 & Rosenqvist 2023" -0.02 0.11  "Li 2024" 0.33 0.07  "Mitchell 2020" 0.88 0.18  "Hangai 2009 & Owen 2021" 0.21 0.03  "Teichtahl 2015" 0.05 0.29  "Vadala 2014" -0.86 0.19  "Zhang 2023" 0.51 0.30  end  format %9.0f es  format %9.2f se  meta set es se, studylabel(study) eslabel(Hedges' g)  meta summarize, cformat(%9.2f)  //Sensitivity analysis: ρ=0.2  clear  input str50 study es se  "Belavy 2017, 2019 & 2020" 0.27 0.06  "Benedikter 2022" -0.22 0.15  "Bowden 2018" 0.40 0.24  "Frenken 2022" 2.57 0.37  "Jentzsch 2020" -0.11 0.39  "Lagerstrand 2021 & Rosenqvist 2023" -0.02 0.11  "Li 2024" 0.33 0.07  "Mitchell 2020" 0.88 0.18  "Hangai 2009 & Owen 2021" 0.21 0.03  "Teichtahl 2015" 0.05 0.29  "Vadala 2014" -0.86 0.19  "Zhang 2023" 0.51 0.30  end  format %9.0f es  format %9.2f se  meta set es se, studylabel(study) eslabel(Hedges' g)  meta summarize, cformat(%9.2f)  //Sensitivity analysis: ρ=0.4  clear  input str50 study es se  "Belavy 2017, 2019 & 2020" 0.27 0.06  "Benedikter 2022" -0.22 0.15  "Bowden 2018" 0.40 0.24  "Frenken 2022" 2.57 0.37  "Jentzsch 2020" -0.11 0.39  "Lagerstrand 2021 & Rosenqvist 2023" -0.02 0.11  "Li 2024" 0.33 0.07  "Mitchell 2020" 0.88 0.18  "Hangai 2009 & Owen 2021" 0.21 0.03  "Teichtahl 2015" 0.05 0.29  "Vadala 2014" -0.86 0.19  "Zhang 2023" 0.51 0.30  end  format %9.0f es  format %9.2f se  meta set es se, studylabel(study) eslabel(Hedges' g)  meta summarize, cformat(%9.2f)  //Sensitivity analysis: ρ=0.6  clear  input str50 study es se  "Belavy 2017, 2019 & 2020" 0.27 0.06  "Benedikter 2022" -0.22 0.15  "Bowden 2018" 0.40 0.25  "Frenken 2022" 2.57 0.37  "Jentzsch 2020" -0.11 0.39  "Lagerstrand 2021 & Rosenqvist 2023" -0.02 0.11  "Li 2024" 0.33 0.07  "Mitchell 2020" 0.88 0.18  "Hangai 2009 & Owen 2021" 0.21 0.03  "Teichtahl 2015" 0.05 0.29  "Vadala 2014" -0.86 0.19  "Zhang 2023" 0.51 0.30  end  format %9.0f es  format %9.2f se  meta set es se, studylabel(study) eslabel(Hedges' g)  meta summarize, cformat(%9.2f)  //Sensitivity analysis: ρ=1.0  clear  input str50 study es se  "Belavy 2017, 2019 & 2020" 0.27 0.06  "Benedikter 2022" -0.22 0.15  "Bowden 2018" 0.40 0.26  "Frenken 2022" 2.57 0.37  "Jentzsch 2020" -0.11 0.39  "Lagerstrand 2021 & Rosenqvist 2023" -0.02 0.11  "Li 2024" 0.33 0.07  "Mitchell 2020" 0.88 0.18  "Hangai 2009 & Owen 2021" 0.21 0.03  "Teichtahl 2015" 0.05 0.29  "Vadala 2014" -0.86 0.19  "Zhang 2023" 0.51 0.30  end  format %9.0f es  format %9.2f se  meta set es se, studylabel(study) eslabel(Hedges' g)  meta summarize, cformat(%9.2f)  //Sensitivity analysis: leave-one-out: ρ=0.8  clear  input str50 study es se  "Belavy 2017, 2019 & 2020" 0.27 0.06  "Benedikter 2022" -0.22 0.15  "Bowden 2018" 0.40 0.25  "Frenken 2022" 2.57 0.37  "Jentzsch 2020" -0.11 0.39  "Lagerstrand 2021 & Rosenqvist 2023" -0.02 0.11  "Li 2024" 0.33 0.07  "Mitchell 2020" 0.88 0.18  "Hangai 2009 & Owen 2021" 0.21 0.03  "Teichtahl 2015" 0.05 0.29  "Vadala 2014" -0.86 0.19  "Zhang 2023" 0.51 0.30  end  format %9.0f es  format %9.2f se  meta set es se, studylabel(study) eslabel(Hedges' g)  meta summarize, leaveoneout  //Sensitivity analysis: robust variance  clear  input str50 study n1 m1 s1 n2 m2 s2  "Belavy 2017, 2019 & 2020" 73 26.25 3.16 24 25.60 2.40  "Belavy 2017, 2019 & 2020" 55 263.33 59.39 24 250.20 41.20  "Belavy 2017, 2019 & 2020" 73 34.31 2.75 24 34.20 2.00  "Belavy 2017, 2019 & 2020" 73 10.30 3.14 24 9.50 2.30  "Belavy 2017, 2019 & 2020" 73 0.30 0.05 24 0.28 0.05  "Belavy 2017, 2019 & 2020" 73 116.24 20.20 24 107.62 6.62  "Belavy 2017, 2019 & 2020" 73 114.99 17.08 24 108.82 11.49  "Belavy 2017, 2019 & 2020" 73 111.19 18.29 24 108.27 12.61  "Belavy 2017, 2019 & 2020" 73 105.65 18.93 24 100.13 16.97  "Belavy 2017, 2019 & 2020" 73 104.91 19.31 24 94.98 20.71  "Belavy 2017, 2019 & 2020" 77 1.49 0.13 24 1.40 0.15  "Belavy 2017, 2019 & 2020" 77 322.00 24.23 24 326.90 24.00  "Belavy 2017, 2019 & 2020" 77 3.35 0.34 24 3.31 0.34  "Belavy 2017, 2019 & 2020" 77 87.42 3.19 24 86.00 2.94  "Belavy 2017, 2019 & 2020" 77 1.01 0.01 24 1.01 0.01  "Benedikter 2022" 17 32.82 30.31 37 37.99 32.71  "Benedikter 2022" 17 35.94 27.94 37 43.42 32.49  "Benedikter 2022" 17 40.31 34.98 37 52.50 39.76  "Benedikter 2022" 17 39.05 40.15 37 46.12 35.09  "Bowden 2018" 14 -0.15 0.06 12 -0.17 0.11  "Bowden 2018" 14 1.15 0.48 12 0.79 0.49  "Bowden 2018" 14 55.80 24.70 12 49.70 21.40  "Frenken 2022" 17 2.58 0.27 44 1.66 0.38  "Jentzsch 2020" 12 85.60 14.90 13 87.20 13.50  "Lagerstrand 2021 & Rosenqvist 2023" 58 0.74 0.33 26 0.76 0.34  "Lagerstrand 2021 & Rosenqvist 2023" 58 1.06 0.35 26 1.14 0.40  "Lagerstrand 2021 & Rosenqvist 2023" 58 1.30 0.48 26 1.33 0.43  "Lagerstrand 2021 & Rosenqvist 2023" 58 1.13 0.44 26 1.12 0.41  "Lagerstrand 2021 & Rosenqvist 2023" 58 0.53 0.28 26 0.47 0.23  "Li 2024" 54 21.54 4.47 30 19.42 3.68  "Li 2024" 54 22.50 5.07 30 21.28 5.09  "Li 2024" 54 21.99 4.72 30 19.35 3.47  "Li 2024" 54 23.30 5.30 30 21.68 4.58  "Li 2024" 54 21.59 5.55 30 19.85 4.51  "Li 2024" 54 42.23 11.27 30 39.95 10.72  "Li 2024" 54 47.51 7.74 30 44.71 13.51  "Li 2024" 54 49.34 13.40 30 47.01 15.79  "Li 2024" 54 51.21 15.94 30 43.30 15.84  "Li 2024" 54 47.66 18.48 30 44.16 17.41  "Mitchell 2020" 9 249.00 175.20 8 202.30 149.50  "Mitchell 2020" 9 0.33 0.04 8 0.23 0.02  "Mitchell 2020" 9 0.32 0.05 8 0.25 0.03  "Mitchell 2020" 9 0.31 0.06 8 0.26 0.04  "Mitchell 2020" 9 0.28 0.05 8 0.22 0.04  "Mitchell 2020" 9 101.20 13.60 8 93.10 26.60  "Mitchell 2020" 9 95.10 13.90 8 89.60 26.80  "Mitchell 2020" 9 83.90 12.20 8 85.70 32.70  "Mitchell 2020" 9 85.10 14.80 8 78.80 14.90  "Hangai 2009 & Owen 2021" 308 0.32 0.04 71 0.31 0.04  "Hangai 2009 & Owen 2021" 308 0.36 0.05 71 0.33 0.05  "Hangai 2009 & Owen 2021" 308 0.41 0.05 71 0.38 0.05  "Hangai 2009 & Owen 2021" 308 0.46 0.07 71 0.43 0.07  "Hangai 2009 & Owen 2021" 308 0.43 0.07 71 0.43 0.08  "Hangai 2009 & Owen 2021" 308 3.83 0.87 71 3.78 0.87  "Hangai 2009 & Owen 2021" 308 4.49 0.96 71 4.40 0.98  "Hangai 2009 & Owen 2021" 308 4.93 1.04 71 4.96 1.09  "Hangai 2009 & Owen 2021" 308 4.19 1.27 71 4.34 1.33  "Hangai 2009 & Owen 2021" 308 3.40 1.12 71 3.47 1.16  "Hangai 2009 & Owen 2021" 308 26.84 3.32 71 25.70 3.30  "Hangai 2009 & Owen 2021" 308 28.63 3.36 71 27.70 3.90  "Hangai 2009 & Owen 2021" 308 29.24 3.27 71 28.70 3.40  "Hangai 2009 & Owen 2021" 308 28.75 3.47 71 27.80 3.30  "Hangai 2009 & Owen 2021" 308 23.90 3.33 71 22.80 3.00  "Teichtahl 2015" 57 11.00 15.79 15 10.20 15.88  "Vadala 2014" 13 102.53 26.91 13 116.07 36.54  "Vadala 2014" 13 99.65 17.77 13 114.90 22.59  "Vadala 2014" 13 94.18 16.53 13 112.16 28.16  "Vadala 2014" 13 77.48 20.51 13 110.69 21.59  "Vadala 2014" 13 79.78 21.76 13 100.74 24.92  "Zhang 2023" 27 110.70 24.92 18 98.30 22.20  end  format %9.2f m1  format %9.2f s1  format %9.2f m2  format %9.2f s2  meta esize n1 m1 s1 n2 m2 s2, studylabel(study) esize(hedges) eslabel(Hedges' g)  encode(study), gen(study2)  gen _meta_var =((n1+n2)/(n1*n2))+((_meta_es^2)/(2*(n1+n2-2)))  robumeta _meta_es, study(study2) variance(_meta_var) rho(0.0)  robumeta _meta_es, study(study2) variance(_meta_var) rho(0.2)  robumeta _meta_es, study(study2) variance(_meta_var) rho(0.4)  robumeta _meta_es, study(study2) variance(_meta_var) rho(0.6)  robumeta _meta_es, study(study2) variance(_meta_var) rho(0.8)  robumeta _meta_es, study(study2) variance(_meta_var) rho(0.999)  ***************************************************************************  **#2 Cohort/RCT studies, INT: any exercise compared to serpearte control, OUTCOME: continuous data, n=2  ***************************************************************************  //Calculate effect estimates  clear  input str50 study n1 m1 s1 n2 m2 s2  "Horga 2022" 21 -0.20 1.00 4 0.00 1.00 //more  "Horga 2022" 21 -0.09 1.00 4 0.00 1.00 //more  "Horga 2022" 21 0.04 1.00 4 0.00 1.00 //more  "Horga 2022" 21 -0.18 3.31 4 0.00 3.31 //more  "Horga 2022" 21 -0.16 3.31 4 0.00 3.31 //more  "Horga 2022" 21 -0.05 3.31 4 0.00 3.31 //more  "Horga 2022" 21 -0.12 2.38 4 0.00 2.38 //more  "Horga 2022" 21 -0.09 2.38 4 0.00 2.38 //more  "Horga 2022" 21 0.13 2.38 4 0.00 2.38 //more  "Owen 2020" 17 -1.70 4.70 15 -0.30 5.40 //more  "Owen 2020" 17 -8.70 94.80 15 20.40 126.50 //more  "Owen 2020" 17 -17.90 94.80 15 9.80 110.30 //more  "Owen 2020" 17 -41.80 102.90 15 -37.10 117.50 //more  "Owen 2020" 17 -6.20 149.60 15 -1.20 147.40 //more  "Owen 2020" 17 -19.30 121.20 15 -29.60 101.80 //more  "Owen 2020" 17 0.00 0.60 15 0.30 0.60 //more  "Owen 2020" 17 0.00 0.20 15 0.10 0.20 //more  end  format %9.2f m1  format %9.2f s1  format %9.2f m2  format %9.2f s2  meta esize n1 m1 s1 n2 m2 s2, studylabel(study) esize(hedges) eslabel(Hedges' g)  encode(study), gen(study2)  gen _meta_var =((n1+n2)/(n1*n2))+((_meta_es^2)/(2*(n1+n2-2)))  //Meta-analysis: ρ=0.8  clear  input str50 study es se  "Horga 2022" -0.04 0.18  "Owen 2020" -0.22 0.13  end  format %9.0f es  format %9.2f se  meta set es se, studylabel(study) eslabel(Hedges' g)  meta summarize, predint se(khartung)  meta forestplot, nullrefline(favorsleft("Favours control", al(baseline) height(45)) favorsright("Favours exercise", al(baseline) height(45))) xline(0, lcolor(gs7) lpattern(solid)) title("Exercise vs control") xlab(-2(1)3) predint(,pstyle(ci) lc(red)) se(khartung)  meta funnelplot, title("Exercise up vs Control") graphregion(color(white))  meta trimfill  meta bias, egger  //Sensitivity analysis: ρ=0.0  clear  input str50 study es se  "Horga 2022" -0.04 0.18  "Owen 2020" -0.22 0.13  end  format %9.0f es  format %9.2f se  meta set es se, studylabel(study) eslabel(Hedges' g)  meta summarize, cformat(%9.2f) se(khartung)  //Sensitivity analysis: ρ=0.2  clear  input str50 study es se  "Horga 2022" -0.04 0.18  "Owen 2020" -0.22 0.13  end  format %9.0f es  format %9.2f se  meta set es se, studylabel(study) eslabel(Hedges' g)  meta summarize, cformat(%9.2f) se(khartung)  //Sensitivity analysis: ρ=0.4  clear  input str50 study es se  "Horga 2022" -0.04 0.18  "Owen 2020" -0.22 0.13  end  format %9.0f es  format %9.2f se  meta set es se, studylabel(study) eslabel(Hedges' g)  meta summarize, cformat(%9.2f) se(khartung)  //Sensitivity analysis: ρ=0.6  clear  input str50 study es se  "Horga 2022" -0.04 0.18  "Owen 2020" -0.22 0.13  end  format %9.0f es  format %9.2f se  meta set es se, studylabel(study) eslabel(Hedges' g)  meta summarize, cformat(%9.2f) se(khartung)  //Sensitivity analysis: ρ=1.0  clear  input str50 study es se  "Horga 2022" -0.04 0.18  "Owen 2020" -0.22 0.13  end  format %9.0f es  format %9.2f se  meta set es se, studylabel(study) eslabel(Hedges' g)  meta summarize, cformat(%9.2f) se(khartung)  //Sensitivity analysis: leave-one-out: ρ=0.8  clear  input str50 study es se  "Horga 2022" -0.04 0.18  "Owen 2020" -0.22 0.13  end  format %9.0f es  format %9.2f se  meta set es se, studylabel(study) eslabel(Hedges' g)  meta summarize, leaveoneout se(khartung)  **************************************************************  **#3 Cohort/RCT, INT: Any exercise continuous data with baseline as control group, OUTCOME: continuous data, n=5  *****************************************************************  //Calculate effect estimates  clear  input str50 study n1 m1 s1 n2 m2 s2  "Frenken 2022" 17 2.58 0.27 17 2.11 0.18 //more  "Horga 2022" 28 10.86 1.00 21 10.41 1.00 //more  "Horga 2022" 28 11.26 1.00 21 11.17 1.00 //more  "Horga 2022" 28 9.56 1.00 21 9.60 1.00 //more  "Horga 2022" 28 33.61 3.31 21 33.43 3.31 //more  "Horga 2022" 28 33.67 3.31 21 33.51 3.31 //more  "Horga 2022" 28 32.64 3.31 21 32.59 3.31 //more  "Horga 2022" 28 26.98 2.38 21 26.86 2.38 //more  "Horga 2022" 28 24.63 2.38 21 24.54 2.38 //more  "Horga 2022" 28 24.04 2.38 21 24.17 2.38 //more  "Owen 2020" 17 94.10 4.70 20 95.80 11.30 //more  "Owen 2020" 17 810.90 94.80 20 819.60 131.30 //more  "Owen 2020" 17 789.40 94.80 20 807.30 102.30 //more  "Owen 2020" 17 797.90 102.90 20 807.30 102.30 //more  "Owen 2020" 17 743.60 149.60 20 749.80 171.50 //more  "Owen 2020" 17 605.60 121.20 20 624.90 162.60 //more  "Owen 2020" 17 8.60 0.60 20 8.60 1.90 //more  "Owen 2020" 17 8.10 0.20 20 8.10 0.80 //more  "Lagerstand 2021 & Rosenqvist 2023, high load" 10 0.22 0.23 10 0.23 0.20 //more  "Lagerstand 2021 & Rosenqvist 2023, low load" 7 0.22 0.14 7 0.25 0.18 //more  end  format %9.2f m1  format %9.2f s1  format %9.2f m2  format %9.2f s2  meta esize n1 m1 s1 n2 m2 s2, studylabel(study) esize(hedges) eslabel(Hedges' g)  encode(study), gen(study2)  gen _meta_var =((n1+n2)/(n1*n2))+((_meta_es^2)/(2*(n1+n2-2)))  //Meta-analysis: ρ=0.8  clear  input str50 study es se  "Frenken 2022" 2.00 0.41  "Horga 2022" 0.07 0.10  "Owen 2020" -0.09 0.12  "Lagerstand 2021 & Rosenqvist 2023, high load" -0.04 0.43  "Lagerstand 2021 & Rosenqvist 2023, low load" -0.17 0.50  end  format %9.0f es  format %9.2f se  meta set es se, studylabel(study) eslabel(Hedges' g)  meta summarize, predint se(khartung)  meta forestplot, nullrefline(favorsleft("Favours baseline", al(baseline) height(45)) favorsright("Favours follow up", al(baseline) height(45))) xline(0, lcolor(gs7) lpattern(solid)) title("Exercise follow up vs baseline (varying time frames)") xlab(-2(1)3) predint(,pstyle(ci) lc(red)) se(khartung)  meta funnelplot, title("Exercise follow up vs baseline control") graphregion(color(white))  meta trimfill  meta bias, egger  //Sensitivity analysis: ρ=0.0  clear  input str50 study es se  "Frenken 2022" 2.00 0.41  "Horga 2022" 0.07 0.10  "Owen 2020" -0.09 0.12  "Lagerstand 2021 & Rosenqvist 2023, high load" -0.04 0.43  "Lagerstand 2021 & Rosenqvist 2023, low load" -0.17 0.50  end  format %9.0f es  format %9.2f se  meta set es se, studylabel(study) eslabel(Hedges' g)  meta summarize, cformat(%9.2f) se(khartung)  //Sensitivity analysis: ρ=0.2  clear  input str50 study es se  "Frenken 2022" 2.00 0.41  "Horga 2022" 0.07 0.10  "Owen 2020" -0.09 0.12  "Lagerstand 2021 & Rosenqvist 2023, high load" -0.04 0.43  "Lagerstand 2021 & Rosenqvist 2023, low load" -0.17 0.50  end  format %9.0f es  format %9.2f se  meta set es se, studylabel(study) eslabel(Hedges' g)  meta summarize, cformat(%9.2f) se(khartung)  //Sensitivity analysis: ρ=0.4  clear  input str50 study es se  "Frenken 2022" 2.00 0.41  "Horga 2022" 0.07 0.10  "Owen 2020" -0.09 0.12  "Lagerstand 2021 & Rosenqvist 2023, high load" -0.04 0.43  "Lagerstand 2021 & Rosenqvist 2023, low load" -0.17 0.50  end  format %9.0f es  format %9.2f se  meta set es se, studylabel(study) eslabel(Hedges' g)  meta summarize, cformat(%9.2f) se(khartung)  //Sensitivity analysis: ρ=0.6  clear  input str50 study es se  "Frenken 2022" 2.00 0.41  "Horga 2022" 0.07 0.10  "Owen 2020" -0.09 0.12  "Lagerstand 2021 & Rosenqvist 2023, high load" -0.04 0.43  "Lagerstand 2021 & Rosenqvist 2023, low load" -0.17 0.50  end  format %9.0f es  format %9.2f se  meta set es se, studylabel(study) eslabel(Hedges' g)  meta summarize, cformat(%9.2f) se(khartung)  //Sensitivity analysis: ρ=1.0  clear  input str50 study es se  "Frenken 2022" 2.00 0.41  "Horga 2022" 0.07 0.10  "Owen 2020" -0.09 0.12  "Lagerstand 2021 & Rosenqvist 2023, high load" -0.04 0.43  "Lagerstand 2021 & Rosenqvist 2023, low load" -0.17 0.50  end  format %9.0f es  format %9.2f se  meta set es se, studylabel(study) eslabel(Hedges' g)  meta summarize, cformat(%9.2f) se(khartung)  //Sensitivity analysis: leave-one-out: ρ=0.8  clear  input str50 study es se  "Frenken 2022" 2.00 0.41  "Horga 2022" 0.07 0.10  "Owen 2020" -0.09 0.12  "Lagerstand 2021 & Rosenqvist 2023, high load" -0.04 0.43  "Lagerstand 2021 & Rosenqvist 2023, low load" -0.17 0.50  end  format %9.0f es  format %9.2f se  meta set es se, studylabel(study) eslabel(Hedges' g)  meta summarize, leaveoneout se(khartung)  ***************************************************************************  **Primary-subgroup analysis, different intervention types by loading on spine  ***************************************************************************  ***************************************************************************  **#1 INT: Upright Bipedal vs control, OUTCOME: Continuous IVD health, n=6  ***************************************************************************  clear  input str50 study n1 m1 s1 n2 m2 s2  "Belavy 2017, 2019 & 2020" 55 10.10 3.32 24 9.50 2.30 //more  "Belavy 2017, 2019 & 2020" 55 263.33 59.39 24 250.20 41.20 //more  "Belavy 2017, 2019 & 2020" 55 26.17 3.43 24 25.60 2.40 //more  "Belavy 2017, 2019 & 2020" 55 34.05 2.83 24 34.20 2.00 //more  "Belavy 2017, 2019 & 2020" 55 0.33 0.04 24 0.33 0.05 //more  "Belavy 2017, 2019 & 2020" 55 0.37 0.07 24 0.36 0.06 //more  "Belavy 2017, 2019 & 2020" 55 0.33 0.09 24 0.31 0.05 //more  "Belavy 2017, 2019 & 2020" 55 0.22 0.05 24 0.21 0.04 //more  "Belavy 2017, 2019 & 2020" 55 0.23 0.05 24 0.21 0.03 //more  "Belavy 2017, 2019 & 2020" 55 118.52 19.82 24 107.62 6.62 //more  "Belavy 2017, 2019 & 2020" 55 118.32 16.93 24 108.82 11.49 //more  "Belavy 2017, 2019 & 2020" 55 116.44 16.74 24 108.27 12.61 //more  "Belavy 2017, 2019 & 2020"55 110.54 18.69 24 100.13 16.97 //more  "Belavy 2017, 2019 & 2020" 55 108.05 19.40 24 94.98 20.71 //more  "Belavy 2017, 2019 & 2020" 55 1.49 0.13 24 1.40 0.15 //more  "Belavy 2017, 2019 & 2020" 55 318.92 23.88 24 326.90 24.00 //more  "Belavy 2017, 2019 & 2020" 55 3.37 0.35 24 3.31 0.34 //more  "Belavy 2017, 2019 & 2020" 55 87.55 3.17 24 86.00 2.94 //more  "Belavy 2017, 2019 & 2020" 55 1.01 0.01 24 1.01 0.01 //more  "Bowden 2018" 14 -0.15 0.06 12 -0.17 0.11 //less  "Bowden 2018" 14 1.15 0.48 12 0.79 0.49 //more  "Bowden 2018" 14 55.80 24.70 12 49.70 21.40 //more  "Lagerstrand 2021 & Rosenqvist 2023"58 0.74 0.33 26 0.76 0.34 //more  "Lagerstrand 2021 & Rosenqvist 2023"58 1.06 0.35 26 1.14 0.40 //more  "Lagerstrand 2021 & Rosenqvist 2023" 58 1.30 0.48 26 1.33 0.43 //more  "Lagerstrand 2021 & Rosenqvist 2023" 58 1.13 0.44 26 1.12 0.41 //more  "Lagerstrand 2021 & Rosenqvist 2023" 58 0.53 0.28 26 0.47 0.23 //more  "Li 2024" 54 21.54 4.47 30 19.42 3.68  "Li 2024" 54 22.50 5.07 30 21.28 5.09  "Li 2024" 54 21.99 4.72 30 19.35 3.47  "Li 2024" 54 23.30 5.30 30 21.68 4.58  "Li 2024" 54 21.59 5.55 30 19.85 4.51  "Li 2024" 54 42.23 11.27 30 39.95 10.72  "Li 2024" 54 47.51 7.74 30 44.71 13.51  "Li 2024" 54 49.34 13.40 30 47.01 15.79  "Li 2024" 54 51.21 15.94 30 43.30 15.84  "Li 2024" 54 47.66 18.48 30 44.16 17.41  "Mitchell 2020" 9 0.33 0.04 8 0.23 0.02 //more  "Mitchell 2020" 9 0.32 0.05 8 0.25 0.03 //more  "Mitchell 2020" 9 0.31 0.06 8 0.26 0.04 //more  "Mitchell 2020" 9 0.28 0.05 8 0.22 0.04 //more  "Mitchell 2020" 9 101.20 13.60 8 93.10 26.60 //more  "Mitchell 2020" 9 95.10 13.90 8 89.60 26.80 //more  "Mitchell 2020" 9 83.90 12.20 8 85.70 32.70 //more  "Mitchell 2020" 9 85.10 14.80 8 78.80 14.90 //more  "Mitchell 2020" 9 249.00 175.20 8 202.30 149.50 //more  "Hangai 2009 & Owen 2021" 153 26.78 3.16 71 25.70 3.30 //more  "Hangai 2009 & Owen 2021" 153 28.71 3.19 71 27.70 3.90 //more  "Hangai 2009 & Owen 2021" 153 29.44 3.31 71 28.70 3.40 //more  "Hangai 2009 & Owen 2021"153 28.62 3.61 71 27.80 3.30 //more  "Hangai 2009 & Owen 2021"153 23.46 3.05 71 22.80 3.00 //more  "Hangai 2009 & Owen 2021" 153 4.06 0.83 71 3.78 0.87 //more  "Hangai 2009 & Owen 2021"153 4.57 0.98 71 4.40 0.98 //more  "Hangai 2009 & Owen 2021" 153 5.05 1.04 71 4.96 1.09 //more  "Hangai 2009 & Owen 2021" 153 4.30 1.27 71 4.34 1.33 //more  "Hangai 2009 & Owen 2021" 153 3.59 1.10 71 3.47 1.16 //more  "Hangai 2009 & Owen 2021"153 0.37 0.05 71 0.33 0.05 //more  "Hangai 2009 & Owen 2021" 153 0.46 0.06 71 0.43 0.07 //more  "Hangai 2009 & Owen 2021" 153 0.43 0.07 71 0.43 0.08 //more  "Teichtahl 2015" 57 11.00 15.79 15 10.20 15.88 //more  end  format %9.2f m1  format %9.2f s1  format %9.2f m2  format %9.2f s2  meta esize n1 m1 s1 n2 m2 s2, studylabel(study) esize(hedges) eslabel(Hedges' g)  encode(study), gen(study2)  gen _meta_var =((n1+n2)/(n1*n2))+((_meta_es^2)/(2*(n1+n2-2)))  //Main analysis with rho=0.8  clear  input str50 study es se  "Belavy 2017, 2019 & 2020" 0.29 0.07  "Bowden 2018" 0.40 0.25  "Lagerstrand 2021 & Rosenqvist 2023" -0.02 0.11  "Li 2024" 0.33 0.07  "Mitchell 2020" 0.88 0.18  "Hangai 2009 & Owen 2021" 0.27 0.04  "Teichtahl 2015" 0.05 0.29  end  format %9.0f es  format %9.2f se  meta set es se, studylabel(study) eslabel(Hedges' g)  meta summarize, predint  meta forestplot, nullrefline(favorsleft("Favours control", al(baseline) height(45)) favorsright("Favours exercise", al(baseline) height(45))) xline(0, lcolor(gs7) lpattern(solid)) title("Upright Bipedal vs Control") xlab(-2(1)3) predint(,pstyle(ci) lc(red))  meta funnelplot, title("Upright Bipedal vs Control") graphregion(color(white))  meta bias, egger  //Sensitivity analyses with different rho  *****sensitivity analysis: rho=0.0  clear  input str50 study es se  "Belavy 2017, 2019 & 2020" 0.29 0.07  "Bowden 2018" 0.40 0.23  "Lagerstrand 2021 & Rosenqvist 2023" -0.02 0.11  "Li 2024" 0.33 0.07  "Mitchell 2020" 0.88 0.18  "Hangai 2009 & Owen 2021" 0.27 0.04  "Teichtahl 2015" 0.05 0.29  end  format %9.0f es  format %9.2f se  meta set es se, studylabel(study) eslabel(Hedges' g)  meta summarize, cformat(%9.2f)  *************sensitivity analysis: rho=0.2  clear  input str50 study es se  "Belavy 2017, 2019 & 2020" 0.29 0.07  "Bowden 2018" 0.40 0.24  "Lagerstrand 2021 & Rosenqvist 2023" -0.02 0.11  "Li 2024" 0.33 0.07  "Mitchell 2020" 0.88 0.18  "Hangai 2009 & Owen 2021" 0.27 0.04  "Teichtahl 2015" 0.05 0.29  end  format %9.0f es  format %9.2f se  meta set es se, studylabel(study) eslabel(Hedges' g)  meta summarize, cformat(%9.2f)  *********sensitivity analysis: rho=0.4  clear  input str50 study es se  "Belavy 2017, 2019 & 2020" 0.29 0.07  "Bowden 2018" 0.40 0.24  "Lagerstrand 2021 & Rosenqvist 2023" -0.02 0.11  "Li 2024" 0.33 0.07  "Mitchell 2020" 0.88 0.18  "Hangai 2009 & Owen 2021" 0.27 0.04  "Teichtahl 2015" 0.05 0.29  end  format %9.0f es  format %9.2f se  meta set es se, studylabel(study) eslabel(Hedges' g)  meta summarize, cformat(%9.2f)  **********sensitivity analysis: rho=0.6  clear  input str50 study es se  "Belavy 2017, 2019 & 2020" 0.29 0.07  "Bowden 2018" 0.40 0.25  "Lagerstrand 2021 & Rosenqvist 2023" -0.02 0.11  "Li 2024" 0.33 0.07  "Mitchell 2020" 0.88 0.18  "Hangai 2009 & Owen 2021" 0.27 0.04  "Teichtahl 2015" 0.05 0.29  end  format %9.0f es  format %9.2f se  meta set es se, studylabel(study) eslabel(Hedges' g)  meta summarize, cformat(%9.2f)  ************sensitivity analysis: rho=1.0  clear  input str50 study es se  "Belavy 2017, 2019 & 2020" 0.29 0.07  "Bowden 2018" 0.40 0.26  "Lagerstrand 2021 & Rosenqvist 2023" -0.02 0.11  "Li 2024" 0.33 0.07  "Mitchell 2020" 0.88 0.18  "Hangai 2009 & Owen 2021" 0.27 0.04  "Teichtahl 2015" 0.05 0.29  end  format %9.0f es  format %9.2f se  meta set es se, studylabel(study) eslabel(Hedges' g)  meta summarize, cformat(%9.2f)  **********************sensitivity analysis: leave-one-out with rho=0.8  clear  input str50 study es se  "Belavy 2017, 2019 & 2020" 0.29 0.07  "Bowden 2018" 0.40 0.25  "Lagerstrand 2021 & Rosenqvist 2023" -0.02 0.11  "Li 2024" 0.33 0.07  "Mitchell 2020" 0.88 0.18  "Hangai 2009 & Owen 2021" 0.27 0.04  "Teichtahl 2015" 0.05 0.29  end  format %9.0f es  format %9.2f se  meta set es se, studylabel(study) eslabel(Hedges' g)  meta summarize, leaveoneout    ***************************************************************************  **#2 Primary-subgroup analysis: INT: Non-upright vs control, OUTCOME: Continuous IVD health, n=4  ***************************************************************************  clear  input str50 study n1 m1 s1 n2 m2 s2  "Belavy 2017, 2019 & 2020" 18 26.50 2.20 18 25.70 2.20 //more  "Belavy 2017, 2019 & 2020" 18 10.90 2.50 18 9.60 2.10 //more  "Belavy 2017, 2019 & 2020" 18 35.10 2.40 18 34.40 1.80 //more  "Belavy 2017, 2019 & 2020" 18 32.40 2.90 18 29.60 3.80 //more  "Belavy 2017, 2019 & 2020" 18 109.28 20.29 18 95.25 6.27 //more  "Belavy 2017, 2019 & 2020" 18 104.83 13.44 18 95.51 10.87 //more  "Belavy 2017, 2019 & 2020" 18 95.13 12.82 18 92.83 7.83 //more  "Belavy 2017, 2019 & 2020" 18 90.70 9.70 18 88.03 10.76 //more  "Belavy 2017, 2019 & 2020" 18 95.32 15.97 18 88.91 13.63 //more  "Belavy 2017, 2019 & 2020" 22 1.49 0.14 24 1.40 0.15 //more  "Belavy 2017, 2019 & 2020" 22 329.70 23.92 24 326.90 24.00 //more  "Belavy 2017, 2019 & 2020" 22 3.29 0.33 24 3.31 0.34 //more  "Belavy 2017, 2019 & 2020" 22 87.10 3.28 24 86.00 2.94 //more  "Belavy 2017, 2019 & 2020" 22 1.00 0.01 24 1.01 0.01 //more  "Benedikter 2022" 17 32.82 30.31 37 37.99 32.71 //more  "Benedikter 2022" 17 35.94 27.94 37 43.42 32.49 //more  "Benedikter 2022" 17 40.31 34.98 37 52.50 39.76 //more  "Benedikter 2022" 17 39.05 40.15 37 46.12 35.09 //more  "Frenken 2022" 17 2.58 0.27 44 1.66 0.38 //more  "Hangai 2009 & Owen 2021" 47 25.00 3.60 71 25.70 3.30 //more  "Hangai 2009 & Owen 2021"47 27.90 3.40 71 27.70 3.90 //more  "Hangai 2009 & Owen 2021" 47 28.40 3.00 71 28.70 3.40 //more  "Hangai 2009 & Owen 2021" 47 29.00 3.70 71 27.80 3.30 //more  "Hangai 2009 & Owen 2021"47 25.60 4.00 71 22.80 3.00 //more  "Hangai 2009 & Owen 2021"47 3.59 0.93 71 3.78 0.87 //more  "Hangai 2009 & Owen 2021" 47 4.51 0.93 71 4.40 0.98 //more  "Hangai 2009 & Owen 2021" 47 4.94 1.03 71 4.96 1.09 //more  "Hangai 2009 & Owen 2021" 47 4.13 1.26 71 4.34 1.33 //more  "Hangai 2009 & Owen 2021" 47 3.20 1.10 71 3.47 1.16 //more  "Hangai 2009 & Owen 2021" 47 0.36 0.04 71 0.31 0.04 //more  "Hangai 2009 & Owen 2021" 47 0.37 0.05 71 0.33 0.05 //more  "Hangai 2009 & Owen 2021" 47 0.41 0.05 71 0.38 0.05 //more  "Hangai 2009 & Owen 2021" 47 0.47 0.06 71 0.43 0.07 //more  "Hangai 2009 & Owen 2021" 47 0.45 0.07 71 0.43 0.08 //more  end  format %9.2f m1  format %9.2f s1  format %9.2f m2  format %9.2f s2  meta esize n1 m1 s1 n2 m2 s2, studylabel(study) esize(hedges) eslabel(Hedges' g)  encode(study), gen(study2) /  gen _meta_var =((n1+n2)/(n1*n2))+((_meta_es^2)/(2*(n1+n2-2)))  //Main analysis with rho=0.8 and Knapp Hartung adjustment.  clear  input str50 study es se  "Belavy 2017, 2019 & 2020" 0.33 0.14  "Benedikter 2022" -0.22 0.15  "Frenken 2022" 2.57 0.37  "Hangai 2009 & Owen 2021" 0.26 0.05  end  format %9.0f es  format %9.2f se  meta set es se, studylabel(study) eslabel(Hedges' g)  meta summarize, predint se(khartung)  meta forestplot, nullrefline(favorsleft("Favours control", al(baseline) height(45)) favorsright("Favours exercise", al(baseline) height(45))) xline(0, lcolor(gs7) lpattern(solid)) title("Non-upright Exercise vs Control") xlab(-2(1)3) predint(,pstyle(ci) lc(red)) se(khartung)  meta funnelplot, title("Non-upright Exercise vs Control") graphregion(color(white))  meta bias, egger  //Sensitivity analyses with different rho  ********************sensitivity analysis: rho=0.0*******************  clear  input str50 study es se  "Belavy 2017, 2019 & 2020" 0.33 0.12  "Benedikter 2022" -0.22 0.15  "Frenken 2022" 2.57 0.37  "Hangai 2009 & Owen 2021" 0.26 0.05  end  format %9.0f es  format %9.2f se  meta set es se, studylabel(study) eslabel(Hedges' g)  meta summarize, cformat(%9.2f) se(khartung)  ********************sensitivity analysis: rho=0.2*******************  clear  input str50 study es se  "Belavy 2017, 2019 & 2020" 0.33 0.13  "Benedikter 2022" -0.22 0.15  "Frenken 2022" 2.57 0.37  "Hangai 2009 & Owen 2021" 0.26 0.05  end  format %9.0f es  format %9.2f se  meta set es se, studylabel(study) eslabel(Hedges' g)  meta summarize, cformat(%9.2f) se(khartung)  ********************sensitivity analysis: rho=0.4*******************  clear  input str50 study es se  "Belavy 2017, 2019 & 2020" 0.33 0.13  "Benedikter 2022" -0.22 0.15  "Frenken 2022" 2.57 0.37  "Hangai 2009 & Owen 2021" 0.26 0.05  end  format %9.0f es  format %9.2f se  meta set es se, studylabel(study) eslabel(Hedges' g)  meta summarize, cformat(%9.2f) se(khartung)  ********************sensitivity analysis: rho=0.6*******************  clear  input str50 study es se  "Belavy 2017, 2019 & 2020" 0.33 0.14  "Benedikter 2022" -0.22 0.15  "Frenken 2022" 2.57 0.37  "Hangai 2009 & Owen 2021" 0.26 0.05  end  format %9.0f es  format %9.2f se  meta set es se, studylabel(study) eslabel(Hedges' g)  meta summarize, cformat(%9.2f) se(khartung)  ********************sensitivity analysis: rho=1.0*******************  clear  input str50 study es se  "Belavy 2017, 2019 & 2020" 0.33 0.15  "Benedikter 2022" -0.22 0.15  "Frenken 2022" 2.57 0.37  "Hangai 2009 & Owen 2021" 0.26 0.05  end  format %9.0f es  format %9.2f se  meta set es se, studylabel(study) eslabel(Hedges' g)  meta summarize, cformat(%9.2f) se(khartung)  ********************sensitivity analysis: leave-one-out*******************  //with rho=0.8  clear  input str50 study es se  "Belavy 2017, 2019 & 2020" 0.33 0.14  "Benedikter 2022" -0.22 0.15  "Frenken 2022" 2.57 0.37  "Hangai 2009 & Owen 2021" 0.26 0.05  end  format %9.0f es  format %9.2f se  meta set es se, studylabel(study) eslabel(Hedges' g)  meta summarize, leaveoneout se(khartung)    *************************************************************  **#3 Primary-subgroup analysis, INT: Extreme Trunk ROM vs control, OUTCOME: Continuous IVD health. n=4  **************************************************************  clear  input str50 study n1 m1 s1 n2 m2 s2  "Jentzsch 2020" 12 85.60 14.90 13 87.20 13.50 //more  "Hangai 2009 & Owen 2021" 108 27.72 3.10 71 25.70 3.30 //more  "Hangai 2009 & Owen 2021" 108 28.83 3.57 71 27.70 3.90 //more  "Hangai 2009 & Owen 2021"108 29.32 3.29 71 28.70 3.40 //more  "Hangai 2009 & Owen 2021" 108 28.82 3.16 71 27.80 3.30 //more  "Hangai 2009 & Owen 2021"108 23.78 3.18 71 22.80 3.00 //more  "Hangai 2009 & Owen 2021" 108 3.61 0.83 71 3.78 0.87 //more  "Hangai 2009 & Owen 2021" 108 4.38 0.94 71 4.40 0.98 //more  "Hangai 2009 & Owen 2021" 108 4.75 1.04 71 4.96 1.09 //more  "Hangai 2009 & Owen 2021" 108 4.04 1.28 71 4.34 1.33 //more  "Hangai 2009 & Owen 2021" 108 3.20 1.11 71 3.47 1.16 //more  "Hangai 2009 & Owen 2021"108 0.31 0.04 71 0.31 0.04 //more  "Hangai 2009 & Owen 2021" 108 0.35 0.05 71 0.33 0.05 //more  "Hangai 2009 & Owen 2021" 108 0.40 0.05 71 0.38 0.05 //more  "Hangai 2009 & Owen 2021" 108 0.45 0.07 71 0.43 0.07 //more  "Hangai 2009 & Owen 2021" 108 0.42 0.07 71 0.43 0.08 //more  "Vadala 2014" 13 102.53 26.91 13 116.07 36.54 //more  "Vadala 2014" 13 99.65 17.77 13 114.90 22.59 //more  "Vadala 2014" 13 94.18 16.53 13 112.16 28.16 //more  "Vadala 2014" 13 77.48 20.51 13 110.69 21.59 //more  "Vadala 2014" 13 79.78 21.76 13 100.74 24.92 //more  "Zhang 2023" 27 110.70 24.92 18 98.30 22.20 //more  end  format %9.2f m1  format %9.2f s1  format %9.2f m2  format %9.2f s2  meta esize n1 m1 s1 n2 m2 s2, studylabel(study) esize(hedges) eslabel(Hedges' g)  encode(study), gen(study2)  gen _meta_var =((n1+n2)/(n1*n2))+((_meta_es^2)/(2*(n1+n2-2)))  //Main analysis with rho=0.8  clear  input str50 study es se  "Jentzsch 2020" -0.11 0.39  "Hangai 2009 & Owen 2021" 0.12 0.04  "Vadala 2014" -0.86 0.19  "Zhang 2023" 0.51 0.30  end  format %9.0f es  format %9.2f se  meta set es se, studylabel(study) eslabel(Hedges' g)  meta summarize, predint se(khartung)  meta forestplot, nullrefline(favorsleft("Favours control", al(baseline) height(45)) favorsright("Favours exercise", al(baseline) height(45))) xline(0, lcolor(gs7) lpattern(solid)) xlab(-2(1)3) predint(,pstyle(ci) lc(red)) se(khartung)  meta funnelplot, title("Trunk Extreme ROM Exercise vs Control") graphregion(color(white))  meta bias, egger //P=0.829  //Sensitivity analyses with different rho  *********************************************sensitivity analysis: rho=0.0  clear  input str50 study es se  "Jentzsch 2020" -0.11 0.39  "Hangai 2009 & Owen 2021" 0.12 0.04  "Vadala 2014" -0.86 0.19  "Zhang 2023" 0.51 0.30  end  format %9.0f es  format %9.2f se  meta set es se, studylabel(study) eslabel(Hedges' g)  meta summarize, cformat(%9.2f) se(khartung)  ***********************************************sensitivity analysis: rho=0.2  clear  input str50 study es se  "Jentzsch 2020" -0.11 0.39  "Hangai 2009 & Owen 2021" 0.12 0.04  "Vadala 2014" -0.86 0.19  "Zhang 2023" 0.51 0.30  end  format %9.0f es  format %9.2f se  meta set es se, studylabel(study) eslabel(Hedges' g)  meta summarize, cformat(%9.2f) se(khartung)  *******************************************sensitivity analysis: rho=0.4  clear  input str50 study es se  "Jentzsch 2020" -0.11 0.39  "Hangai 2009 & Owen 2021" 0.12 0.04  "Vadala 2014" -0.86 0.19  "Zhang 2023" 0.51 0.30  end  format %9.0f es  format %9.2f se  meta set es se, studylabel(study) eslabel(Hedges' g)  meta summarize, cformat(%9.2f) se(khartung)  ***************************************sensitivity analysis: rho=0.6  clear  input str50 study es se  "Jentzsch 2020" -0.11 0.39  "Hangai 2009 & Owen 2021"0.12 0.04  "Vadala 2014" -0.86 0.19  "Zhang 2023" 0.51 0.30  end  format %9.0f es  format %9.2f se  meta set es se, studylabel(study) eslabel(Hedges' g)  meta summarize, cformat(%9.2f) se(khartung)  *****************************************sensitivity analysis: rho=1.0  clear  input str50 study es se  "Jentzsch 2020" -0.11 0.39  "Hangai 2009 & Owen 2021" 0.12 0.04  "Vadala 2014" -0.86 0.19  "Zhang 2023" 0.51 0.30  end  format %9.0f es  format %9.2f se  meta set es se, studylabel(study) eslabel(Hedges' g)  meta summarize, cformat(%9.2f) se(khartung)  ***************************sensitivity analysis: leave-one-out  //Main analysis with rho=0.8  clear  input str50 study es se  "Jentzsch 2020" -0.11 0.39  "Hangai 2009 & Owen 2021" 0.12 0.04  "Vadala 2014" -0.86 0.19  "Zhang 2023" 0.51 0.30  end  format %9.0f es  format %9.2f se  meta set es se, studylabel(study) eslabel(Hedges' g)  meta summarize, leaveoneou se(khartung)  **************************************************************  *#4 Primary-subgroup analysis, INT:Aerobic Exercise Vs Control, OUTCOME: Continuous IVD health, n=8  **************************************************************  clear  input str50 study n1 m1 s1 n2 m2 s2  "Belavy 2017, 2019 & 2020" 73 26.25 3.16 24 25.60 2.40 //more  "Belavy 2017, 2019 & 2020" 55 263.33 59.39 24 250.20 41.20 //more  "Belavy 2017, 2019 & 2020" 73 34.31 2.75 24 34.20 2.00 //more  "Belavy 2017, 2019 & 2020" 73 10.30 3.14 24 9.50 2.30 //more  "Belavy 2017, 2019 & 2020" 73 0.30 0.05 24 0.28 0.05 //more  "Belavy 2017, 2019 & 2020" 73 116.24 20.20 24 107.62 6.62 //more  "Belavy 2017, 2019 & 2020" 73 114.99 17.08 24 108.82 11.49 //more  "Belavy 2017, 2019 & 2020" 73 111.19 18.29 24 108.27 12.61 //more  "Belavy 2017, 2019 & 2020" 73 105.65 18.93 24 100.13 16.97 //more  "Belavy 2017, 2019 & 2020" 73 104.91 19.31 24 94.98 20.71 //more  "Belavy 2017, 2019 & 2020" 77 1.49 0.13 24 1.40 0.15 //more  "Belavy 2017, 2019 & 2020" 77 322.00 24.23 24 326.90 24.00 //more  "Belavy 2017, 2019 & 2020" 77 3.35 0.34 24 3.31 0.34 //more  "Belavy 2017, 2019 & 2020" 77 87.42 3.19 24 86.00 2.94 //more  "Belavy 2017, 2019 & 2020" 77 1.01 0.01 24 1.01 0.01 //more  "Benedikter 2022" 17 32.82 30.31 37 37.99 32.71 //more  "Benedikter 2022" 17 35.94 27.94 37 43.42 32.49 //more  "Benedikter 2022" 17 40.31 34.98 37 52.50 39.76 //more  "Benedikter 2022" 17 39.05 40.15 37 46.12 35.09 //more  "Bowden 2018" 14 -0.15 0.06 12 -0.17 0.11 //less  "Bowden 2018" 14 1.15 0.48 12 0.79 0.49 //more  "Bowden 2018" 14 55.80 24.70 12 49.70 21.40 //more  "Frenken 2022" 17 2.58 0.27 44 1.66 0.38 //more  "Lagerstrand 2021 & Rosenqvist 2023" 58 0.74 0.33 26 0.76 0.34 //more  "Lagerstrand 2021 & Rosenqvist 2023" 58 1.06 0.35 26 1.14 0.40 //more  "Lagerstrand 2021 & Rosenqvist 2023" 58 1.30 0.48 26 1.33 0.43 //more  "Lagerstrand 2021 & Rosenqvist 2023" 58 1.13 0.44 26 1.12 0.41 //more  "Lagerstrand 2021 & Rosenqvist 2023" 58 0.53 0.28 26 0.47 0.23 //more  "Li 2024" 54 21.54 4.47 30 19.42 3.68  "Li 2024" 54 22.50 5.07 30 21.28 5.09  "Li 2024" 54 21.99 4.72 30 19.35 3.47  "Li 2024" 54 23.30 5.30 30 21.68 4.58  "Li 2024" 54 21.59 5.55 30 19.85 4.51  "Li 2024" 54 42.23 11.27 30 39.95 10.72  "Li 2024" 54 47.51 7.74 30 44.71 13.51  "Li 2024" 54 49.34 13.40 30 47.01 15.79  "Li 2024" 54 51.21 15.94 30 43.30 15.84  "Li 2024" 54 47.66 18.48 30 44.16 17.41  "Mitchell 2020" 9 0.33 0.04 8 0.23 0.02 //more  "Mitchell 2020" 9 0.32 0.05 8 0.25 0.03 //more  "Mitchell 2020" 9 0.31 0.06 8 0.26 0.04 //more  "Mitchell 2020" 9 0.28 0.05 8 0.22 0.04 //more  "Mitchell 2020" 9 101.20 13.60 8 93.10 26.60 //more  "Mitchell 2020" 9 95.10 13.90 8 89.60 26.80 //more  "Mitchell 2020" 9 83.90 12.20 8 85.70 32.70 //more  "Mitchell 2020" 9 85.10 14.80 8 78.80 14.90 //more  "Mitchell 2020" 9 249.00 175.20 8 202.30 149.50 //more  "Hangai 2009 & Owen 2021" 251 26.57 3.38 71 25.70 3.30 //more  "Hangai 2009 & Owen 2021" 251 28.48 3.32 71 27.70 3.90 //more  "Hangai 2009 & Owen 2021" 251 29.15 3.28 71 28.70 3.40 //more  "Hangai 2009 & Owen 2021" 251 28.64 3.58 71 27.80 3.30 //more  "Hangai 2009 & Owen 2021" 251 23.81 3.40 71 22.80 3.00 //more  "Hangai 2009 & Owen 2021" 251 3.88 0.87 71 3.78 0.87 //more  "Hangai 2009 & Owen 2021" 251 4.51 0.96 71 4.40 0.98 //more  "Hangai 2009 & Owen 2021" 251 4.99 1.04 71 4.96 1.09 //more  "Hangai 2009 & Owen 2021" 251 4.25 1.26 71 4.34 1.33 //more  "Hangai 2009 & Owen 2021" 251 3.46 1.11 71 3.47 1.16 //more  "Hangai 2009 & Owen 2021" 251 0.32 0.04 71 0.31 0.04 //more  "Hangai 2009 & Owen 2021" 251 0.36 0.05 71 0.33 0.05 //more  "Hangai 2009 & Owen 2021" 251 0.41 0.05 71 0.38 0.05 //more  "Hangai 2009 & Owen 2021" 251 0.46 0.07 71 0.43 0.07 //more  "Hangai 2009 & Owen 2021" 251 0.43 0.07 71 0.43 0.08 //more  "Teichtahl 2015" 57 11.00 15.79 15 10.20 15.88 //more  end  format %9.2f m1  format %9.2f s1  format %9.2f m2  format %9.2f s2  meta esize n1 m1 s1 n2 m2 s2, studylabel(study) esize(hedges) eslabel(Hedges' g)  encode(study), gen(study2)  gen _meta_var =((n1+n2)/(n1*n2))+((_meta_es^2)/(2*(n1+n2-2)))  //Main analysis with rho=0.8  clear  input str50 study es se  "Belavy 2017, 2019 & 2020" 0.27 0.06  "Benedikter 2022" -0.22 0.15  "Bowden 2018" 0.40 0.25  "Frenken 2022" 2.57 0.37  "Lagerstrand 2021 & Rosenqvist 2023" -0.02 0.11  "Li 2024" 0.33 0.07  "Mitchell 2020" 0.88 0.18  "Hangai 2009 & Owen 2021" 0.21 0.03  "Teichtahl 2015" 0.05 0.29  end  format %9.0f es  format %9.2f se  meta set es se, studylabel(study) eslabel(Hedges' g)  meta summarize, predint  meta forestplot, nullrefline(favorsleft("Favours control", al(baseline) height(45)) favorsright("Favours exercise", al(baseline) height(45))) xline(0, lcolor(gs7) lpattern(solid)) xlab(-2(1)3) predint(,pstyle(ci) lc(red))  meta funnelplot, title("Aerobic Exercise vs Control") graphregion(color(white))  meta bias, egger  //Sensitivity analyses with different rho  *********************************************sensitivity analysis: rho=0.0  clear  input str50 study es se  "Belavy 2017, 2019 & 2020" 0.27 0.06  "Benedikter 2022" -0.22 0.15  "Bowden 2018" 0.40 0.23  "Frenken 2022" 2.57 0.37  "Lagerstrand 2021 & Rosenqvist 2023" -0.02 0.11  "Li 2024" 0.33 0.07  "Mitchell 2020" 0.88 0.18  "Hangai 2009 & Owen 2021" 0.21 0.03  "Teichtahl 2015" 0.05 0.29  end  format %9.0f es  format %9.2f se  meta set es se, studylabel(study) eslabel(Hedges' g)  meta summarize, cformat(%9.2f)  ***********************************************sensitivity analysis: rho=0.2  clear  input str50 study es se  "Belavy 2017, 2019 & 2020" 0.27 0.06  "Benedikter 2022" -0.22 0.15  "Bowden 2018" 0.40 0.24  "Frenken 2022" 2.57 0.37  "Lagerstrand 2021 & Rosenqvist 2023" -0.02 0.11  "Li 2024" 0.33 0.07  "Mitchell 2020" 0.88 0.18  "Hangai 2009 & Owen 2021" 0.21 0.03  "Teichtahl 2015" 0.05 0.29  end  format %9.0f es  format %9.2f se  meta set es se, studylabel(study) eslabel(Hedges' g)  meta summarize, cformat(%9.2f)  *******************************************sensitivity analysis: rho=0.4  clear  input str50 study es se  "Belavy 2017, 2019 & 2020" 0.27 0.06  "Benedikter 2022" -0.22 0.15  "Bowden 2018" 0.40 0.24  "Frenken 2022" 2.57 0.37  "Lagerstrand 2021 & Rosenqvist 2023" -0.02 0.11  "Li 2024" 0.33 0.07  "Mitchell 2020" 0.88 0.18  "Hangai 2009 & Owen 2021" 0.21 0.03  "Teichtahl 2015" 0.05 0.29  end  format %9.0f es  format %9.2f se  meta set es se, studylabel(study) eslabel(Hedges' g)  meta summarize, cformat(%9.2f)  ***************************************sensitivity analysis: rho=0.6  clear  input str50 study es se  "Belavy 2017, 2019 & 2020" 0.27 0.06  "Benedikter 2022" -0.22 0.15  "Bowden 2018" 0.40 0.25  "Frenken 2022" 2.57 0.37  "Lagerstrand 2021 & Rosenqvist 2023" -0.02 0.11  "Mitchell 2020" 0.88 0.18  "Li 2024" 0.33 0.07  "Hangai 2009 & Owen 2021" 0.21 0.03  "Teichtahl 2015" 0.05 0.29  end  format %9.0f es  format %9.2f se  meta set es se, studylabel(study) eslabel(Hedges' g)  meta summarize, cformat(%9.2f)  *****************************************sensitivity analysis: rho=1.0  clear  input str50 study es se  "Belavy 2017, 2019 & 2020" 0.27 0.06  "Benedikter 2022" -0.22 0.15  "Bowden 2018" 0.40 0.26  "Frenken 2022" 2.57 0.37  "Lagerstrand 2021 & Rosenqvist 2023" -0.02 0.11  "Li 2024" 0.33 0.07  "Mitchell 2020" 0.88 0.18  "Hangai 2009 & Owen 2021" 0.21 0.03  "Teichtahl 2015" 0.05 0.29  end  format %9.0f es  format %9.2f se  meta set es se, studylabel(study) eslabel(Hedges' g)  meta summarize, cformat(%9.2f)  ***************************sensitivity analysis: leave-one-out  //Step 10: main analysis with rho=0.8  clear  input str50 study es se  "Belavy 2017, 2019 & 2020" 0.27 0.06  "Benedikter 2022" -0.22 0.15  "Bowden 2018" 0.40 0.25  "Frenken 2022" 2.57 0.37  "Lagerstrand 2021 & Rosenqvist 2023" -0.02 0.11  "Li 2024" 0.33 0.07  "Mitchell 2020" 0.88 0.18  "Hangai 2009 & Owen 2021" 0.21 0.03  "Teichtahl 2015" 0.05 0.29  end  format %9.0f es  format %9.2f se  meta set es se, studylabel(study) eslabel(Hedges' g)  meta summarize, leaveoneout    ***************************************************************************  *#5 Primary-subgroup analysis, INT: HIGHER LOAD Vs LOWER LOAD, OUTCOME: Continuous IVD health, n=4  ***************************************************************************  clear  input str50 study n1 m1 s1 n2 m2 s2  "Belavy 2017" 25 10.10 3.30 30 10.10 3.40 //more  "Belavy 2017" 25 263.00 58.90 30 263.60 60.80 //more  "Belavy 2017" 25 25.90 3.40 30 26.40 3.50 //more  "Belavy 2017" 25 34.00 2.80 30 34.10 2.90 //more  "Belavy 2017" 25 0.33 0.05 30 0.33 0.04 //more  "Belavy 2017" 25 0.39 0.09 30 0.36 0.05 //more  "Belavy 2017" 25 0.36 0.11 30 0.31 0.07 //more  "Belavy 2017" 25 0.23 0.06 30 0.22 0.04 //more  "Belavy 2017" 25 0.24 0.04 30 0.23 0.05 //more  "Belavy 2017" 25 120.58 19.24 30 116.80 20.46 //more  "Belavy 2017" 25 118.01 18.17 30 118.58 16.13 //more  "Belavy 2017" 25 117.39 17.72 30 115.65 16.14 //more  "Belavy 2017" 25 112.63 21.34 30 108.79 16.33 //more  "Belavy 2017" 25 109.22 21.77 30 107.08 17.50 //more  "Lagerstand 2021 & Rosenqvist 2023" 10 0.09 0.05 7 0.09 0.05 //more  "Lagerstand 2021 & Rosenqvist 2023" 10 0.15 0.08 7 0.15 0.08 //more  "Lagerstand 2021 & Rosenqvist 2023" 10 0.20 0.12 7 0.20 0.09 //more  "Lagerstand 2021 & Rosenqvist 2023" 10 0.17 0.11 7 0.18 0.09 //more  "Lagerstand 2021 & Rosenqvist 2023" 10 0.08 0.06 7 0.09 0.06 //more  "Teichtahl 2015" 19 11.80 15.69 38 10.60 16.03 //more  "Zhang 2023" 9 103.00 15.57 18 89.86 13.68 //more  "Zhang 2023" 9 105.21 35.66 18 108.19 19.44 //more  "Zhang 2023" 9 136.37 29.19 18 122.86 23.15 //more  "Zhang 2023" 9 104.51 32.63 18 120.46 16.10 //more  "Zhang 2023" 9 115.90 31.81 18 100.70 22.65 //more  end  format %9.2f m1  format %9.2f s1  format %9.2f m2  format %9.2f s2  meta esize n1 m1 s1 n2 m2 s2, studylabel(study) esize(hedges) eslabel(Hedges' g)  encode(study), gen(study2) below  gen _meta_var =((n1+n2)/(n1*n2))+((_meta_es^2)/(2*(n1+n2-2)))  ***Meta-analysis, rho=0.8  clear  input str50 study es se  "Belavy 2017" 0.13 0.12  "Lagerstand 2021 & Rosenqvist 2023" -0.05 0.22  "Teichtahl 2015" 0.07 0.28  "Zhang 2023" 0.24 0.19  end  format %9.0f es  format %9.2f se  meta set es se, studylabel(study) eslabel(Hedges' g)  meta summarize, predint se(khartung)  meta forestplot, nullrefline(favorsleft("Favours control", al(baseline) height(45)) favorsright("Favours exercise", al(baseline) height(45))) xline(0, lcolor(gs7) lpattern(solid)) xlab(-2(1)3) predint(,pstyle(ci) lc(red)) se(khartung)  meta funnelplot, title("High load vs low load") graphregion(color(white))  meta bias, egger  //Sensitivity analyses with different rho  *********************************************sensitivity analysis: rho=0.0  clear  input str50 study es se  "Belavy 2017" 0.13 0.10  "Lagerstand 2021 & Rosenqvist 2023" -0.05 0.22  "Teichtahl 2015" 0.07 0.28  "Zhang 2023" 0.24 0.19  end  format %9.0f es  format %9.2f se  meta set es se, studylabel(study) eslabel(Hedges' g)  meta summarize, cformat(%9.2f) se(khartung)  ***********************************************sensitivity analysis: rho=0.2  clear  input str50 study es se  "Belavy 2017" 0.13 0.11  "Lagerstand 2021 & Rosenqvist 2023" -0.05 0.22  "Teichtahl 2015" 0.07 0.28  "Zhang 2023" 0.24 0.19  end  format %9.0f es  format %9.2f se  meta set es se, studylabel(study) eslabel(Hedges' g)  meta summarize, cformat(%9.2f) se(khartung)  *******************************************sensitivity analysis: rho=0.4  clear  input str50 study es se  "Belavy 2017" 0.13 0.11  "Lagerstand 2021 & Rosenqvist 2023" -0.05 0.22  "Teichtahl 2015" 0.07 0.28  "Zhang 2023" 0.24 0.19  end  format %9.0f es  format %9.2f se  meta set es se, studylabel(study) eslabel(Hedges' g)  meta summarize, cformat(%9.2f) se(khartung)  ***************************************sensitivity analysis: rho=0.6  clear  input str50 study es se  "Belavy 2017" 0.13 0.12  "Lagerstand 2021 & Rosenqvist 2023" -0.05 0.22  "Teichtahl 2015" 0.07 0.28  "Zhang 2023" 0.24 0.19  end  format %9.0f es  format %9.2f se  meta set es se, studylabel(study) eslabel(Hedges' g)  meta summarize, cformat(%9.2f) se(khartung)  *****************************************sensitivity analysis: rho=1.0  clear  input str50 study es se  "Belavy 2017" 0.13 0.13  "Lagerstand 2021 & Rosenqvist 2023" -0.05 0.23  "Teichtahl 2015" 0.07 0.28  "Zhang 2023" 0.24 0.19  end  format %9.0f es  format %9.2f se  meta set es se, studylabel(study) eslabel(Hedges' g)  meta summarize, cformat(%9.2f) se(khartung)  ***************************sensitivity analysis: leave-one-out  //Main analysis with rho=0.8  clear  input str50 study es se  "Belavy 2017" 0.13 0.12  "Lagerstand 2021 & Rosenqvist 2023" -0.05 0.22  "Teichtahl 2015" 0.07 0.28  "Zhang 2023" 0.24 0.19  end  format %9.0f es  format %9.2f se  meta set es se, studylabel(study) eslabel(Hedges' g)  meta summarize, leaveoneout se(khartung)  ***************************************************************************  **# MA-ExIVD, Secondary analysis, IN: Any exercise intervention, OUTCOME: categorical measures of IVD degeneration.  ***************************************************************************  **#1 Secondary analysis cross-sectional, INT: Any exercise compared to control, OUTCOME: average Pfirrmann as continuous data, n=6  ***************************************************************************  //Calculate effect estimates  clear  input str50 study n1 m1 s1 n2 m2 s2  "Belavy 2017, 2019 & 2020" 73 2.16 0.37 42 2.30 0.39 //less  "Benedikter 2022" 20 1.70 0.78 37 1.30 0.56 //less  "Benedikter 2022" 20 1.80 0.98 37 1.24 0.98 //less  "Benedikter 2022" 20 1.65 0.85 37 1.16 0.44 //less  "Benedikter 2022" 20 2.00 1.10 37 1.62 0.71 //less  "Cheng 2008" 18 2.25 0.27 18 2.25 0.27 //less  "Cheng 2008" 18 2.25 0.27 18 2.75 0.55 //less  "Cheng 2008" 18 2.50 0.55 18 2.75 0.82 //less  "Cheng 2008" 18 2.75 0.82 18 2.75 0.82 //less  "Cheng 2008" 18 2.25 0.82 18 2.75 0.82 //less  "Maurer 2020" 288 3.61 5.51 97 5.86 5.52 //less  "Mitchell 2020" 9 1.70 0.50 8 2.30 0.70 //less  "Mitchell 2020" 9 1.90 0.60 8 2.30 0.50 //less  "Mitchell 2020" 9 2.10 0.80 8 2.30 1.00 //less  "Mitchell 2020" 9 2.20 0.70 8 3.30 1.00 //less  "Wegner 2023" 9 2.00 0.84 10 2.00 0.84 //less  "Wegner 2023" 9 2.00 0.84 10 2.00 0.84 //less  "Wegner 2023" 9 2.00 0.84 10 2.00 0.84 //less  "Wegner 2023" 9 2.00 0.84 10 2.00 0.84 //less  "Wegner 2023" 9 2.00 0.84 10 2.00 0.84 //less  "Elfering 2002" 35 2.03 0.17 11 2.09 0.30 //less  "Elfering 2002" 35 2.06 0.34 11 2.09 0.30 //less  "Elfering 2002" 35 2.12 0.32 11 2.09 0.30 //less  "Elfering 2002" 35 2.23 0.49 11 2.09 0.30 //less  "Elfering 2002" 35 2.40 0.74 11 2.64 0.67 //less  end  format %9.2f m1  format %9.2f s1  format %9.2f m2  format %9.2f s2  meta esize n1 m1 s1 n2 m2 s2, studylabel(study) esize(hedges) eslabel(Hedges' g)  encode(study), gen(study2)  gen _meta_var =((n1+n2)/(n1*n2))+((_meta_es^2)/(2*(n1+n2-2)))  //Meta-analysis: ρ=0.8  /*clear  input str50 study es se  "Belavy 2017, 2019 & 2020" -0.37 0.19  "Benedikter 2022" 0.60 0.14  "Cheng 2008" -0.37 0.17  "Maurer 2020" -0.41 0.12  "Mitchell 2020" -0.77 0.27  "Wegner 2023" 0.00 0.21  "Elfering 2002" -0.06 0.16    end  */  *****swapped negative and positive  clear  input str50 study es se  "Belavy 2017, 2019 & 2020" 0.37 0.19  "Benedikter 2022" -0.60 0.14  "Cheng 2008" 0.37 0.17  "Maurer 2020" 0.41 0.12  "Mitchell 2020" 0.77 0.27  "Wegner 2023" 0.00 0.21  "Elfering 2002" 0.06 0.16  end  format %9.0f es  format %9.2f se  meta set es se, studylabel(study) eslabel(Hedges' g)  meta summarize, predint  meta forestplot, esrefline(lcolor(gs12) lpattern(dash)) nullrefline(favorsleft("Favours control", al(baseline) height(45)) favorsright("Favours exercise", al(baseline) height(45))) xline(0, lcolor(gs7) lpattern(solid)) xlab(-2(1)3) predint(,pstyle(ci) lc(red))  meta funnelplot, title("Exercise vs control") graphregion(color(white))  meta trimfill  //Sensitivity analysis: ρ=0.0  clear  input str50 study es se  "Belavy 2017, 2019 & 2020" 0.37 0.19  "Benedikter 2022" -0.60 0.14  "Cheng 2008" 0.37 0.17  "Maurer 2020" 0.41 0.12  "Mitchell 2020" 0.77 0.26  "Wegner 2023" 0.00 0.21  "Elfering 2002" 0.06 0.16  end  format %9.0f es  format %9.2f se  meta set es se, studylabel(study) eslabel(Hedges' g)  meta summarize, cformat(%9.2f)  //Sensitivity analysis: ρ=0.2  clear  input str50 study es se  "Belavy 2017, 2019 & 2020" 0.37 0.19  "Benedikter 2022" -0.60 0.14  "Cheng 2008" 0.37 0.17  "Maurer 2020" 0.41 0.12  "Mitchell 2020" 0.77 0.26  "Wegner 2023" 0.00 0.21  "Elfering 2002" 0.06 0.16  end  format %9.0f es  format %9.2f se  meta set es se, studylabel(study) eslabel(Hedges' g)  meta summarize, cformat(%9.2f)  //Sensitivity analysis: ρ=0.4  clear  input str50 study es se  "Belavy 2017, 2019 & 2020" 0.37 0.19  "Benedikter 2022" -0.60 0.14  "Cheng 2008" 0.37 0.17  "Maurer 2020" 0.41 0.12  "Mitchell 2020" 0.77 0.26  "Wegner 2023" 0.00 0.21  "Elfering 2002" 0.06 0.16  end  format %9.0f es  format %9.2f se  meta set es se, studylabel(study) eslabel(Hedges' g)  meta summarize, cformat(%9.2f)  //Sensitivity analysis: ρ=0.6  clear  input str50 study es se  "Belavy 2017, 2019 & 2020" 0.37 0.19  "Benedikter 2022" -0.60 0.14  "Cheng 2008" 0.37 0.17  "Maurer 2020" 0.41 0.12  "Mitchell 2020" 0.77 0.26  "Wegner 2023" 0.00 0.21  "Elfering 2002" 0.06 0.16  end  format %9.0f es  format %9.2f se  meta set es se, studylabel(study) eslabel(Hedges' g)  meta summarize, cformat(%9.2f)  //Sensitivity analysis: ρ=1.0  clear  input str50 study es se  "Belavy 2017, 2019 & 2020" 0.37 0.19  "Benedikter 2022" -0.60 0.14  "Cheng 2008" 0.37 0.18  "Maurer 2020" 0.41 0.12  "Mitchell 2020" 0.77 0.27  "Wegner 2023" 0.00 0.21  "Elfering 2002" 0.06 0.16  end  format %9.0f es  format %9.2f se  meta set es se, studylabel(study) eslabel(Hedges' g)  meta summarize, cformat(%9.2f)  //Sensitivity analysis: leave-one-out: ρ=0.8  clear  input str50 study es se  "Belavy 2017, 2019 & 2020" 0.37 0.19  "Benedikter 2022" -0.60 0.14 //*-0.357[-0.504,-0.209] p=0.000  "Cheng 2008" 0.37 0.17  "Maurer 2020" 0.41 0.12  "Mitchell 2020" 0.77 0.27  "Wegner 2023" 0.00 0.21  "Elfering 2002" 0.06 0.16  end  format %9.0f es  format %9.2f se  meta set es se, studylabel(study) eslabel(Hedges' g)  meta summarize, leaveoneout  *************************************  **#2 Secondary analysis cross-sectional, Any exercise binary degeneration Pfirrmann >2 compared to control, Total n=10  ****************************************  //Calculate effect estimates  clear  input str50 study int_yes int_no con_yes con_no  "Benedikter 2022" 21 59 11 137  "Cheng 2008" 36 54 54 36  "Elfering 2002" 22 153 10 45  "Folkvardsen 2016" 97 403 78 402  "Hangai 2009 & Owen 2021" 163 1069 24 260  "Identeg 2023" 6 9 10 5  "Kaneoka 2007" 59 221 16 174  "Koyama 2022" 175 1100 24 191  "Kraft 2009" 34 24 18 12  "Ranson 2005" 18 18 8 9  "Witwit 2018 & 2022" 36 39 8 19  end  meta esize int_yes int_no con_yes con_no, studylabel(study)  //Meta-analysis  meta summarize, eform(Odds ratio) random(ebayes) predint  meta forestplot, esrefline(lcolor(gs12) lpattern(dash)) nullrefline(favorsleft("Favours exercise", al(baseline) height(40)) favorsright("Favours control", al(baseline) height(40))) columnopts(_data1, supertitle(Intervention)) columnopts(_data2, supertitle(Control)) xline(0, lcolor(gs7) lpattern(solid)) title("Prevalence of Pfirrmann degeneration any exercsie vs control") eform(Odds ratio) random(ebayes) xlab(minmax 1)  meta funnelplot, title("Any Physical Loading vs Control") graphregion(color(white))  meta trimfill  meta bias, egger  //Sensitivity analysis: leave-one-out  clear  input str50 study int_yes int_no con_yes con_no  "Benedikter 2022" 21 59 11 137  "Cheng 2008" 36 54 54 36  "Elfering 2002" 22 153 10 45  "Folkvardsen 2016" 97 403 78 402  "Hangai 2009 & Owen 2021" 163 1069 24 260  "Identeg 2023" 6 9 10 5  "Kaneoka 2007" 59 221 16 174  "Koyama 2022" 175 1100 24 191  "Kraft 2009" 34 24 18 12  "Ranson 2005" 18 18 8 9  "Witwit 2018 & 2022" 36 39 8 19  end  meta esize int_yes int_no con_yes con_no, studylabel(study)  meta summarize, eform(Odds ratio) random(ebayes) leaveoneout  //Meta-regression: age  clear  input str50 study int_yes int_no con_yes con_no age  "Benedikter 2022" 21 59 11 137 47.4  "Cheng 2008" 36 54 54 36 77.8  "Elfering 2002" 22 153 10 45 35.1  "Folkvardsen 2016" 97 403 78 402 54.6  "Hangai 2009 & Owen 2021" 163 1069 24 260 23.5  "Identeg 2023" 6 9 10 5 53.3  "Kaneoka 2007" 59 221 16 174 37.2  "Koyama 2022" 175 1100 24 191 21.1  "Kraft 2009" 34 24 18 12 60.2  "Ranson 2005" 18 18 8 9 0  "Witwit 2018 & 2022" 36 39 8 19 52  end  meta esize int_yes int_no con_yes con_no, studylabel(study)  meta regress age  //Meta-regression: sex  clear  input str50 study int_yes int_no con_yes con_no sex  "Benedikter 2022" 21 59 11 137 23.9  "Cheng 2008" 36 54 54 36 47.9  "Elfering 2002" 22 153 10 45 27  "Folkvardsen 2016" 97 403 78 402 19.8  "Hangai 2009 & Owen 2021" 163 1069 24 260 19.51  "Identeg 2023" 6 9 10 5 23.7  "Kaneoka 2007" 59 221 16 174 20.35  "Koyama 2022" 175 1100 24 191 19.96  "Kraft 2009" 34 24 18 12 30.6  "Ranson 2005" 18 18 8 9 25.5  "Witwit 2018 & 2022" 36 39 8 19 17.3  end  meta esize int_yes int_no con_yes con_no, studylabel(study)  meta regress sex  //Meta-regression: LBP  clear  input str50 study int_yes int_no con_yes con_no LBP  "Benedikter 2022" 21 59 11 137 77.6  "Cheng 2008" 36 54 54 36 0  "Elfering 2002" 22 153 10 45 43.9  "Folkvardsen 2016" 97 403 78 402 0  "Hangai 2009 & Owen 2021" 163 1069 24 260 59.2  "Identeg 2023" 6 9 10 5 66.7  "Kaneoka 2007" 59 221 16 174 80.9  "Koyama 2022" 175 1100 24 191 13.8  "Kraft 2009" 34 24 18 12 69.3  "Ranson 2005" 18 18 8 9 0  "Witwit 2018 & 2022" 36 39 8 19 47.1  end  meta esize int_yes int_no con_yes con_no, studylabel(study)  meta regress LBP      ***************************************************************************  **#3 Secondary analysis Cohort/RCT, INT: Any exercise compared to baseline as the control, OUTCOME: average Pfirrmann as continuous data, n=3  ***************************************************************************  //Calculate effect estimates  clear  input str50 study n1 m1 s1 n2 m2 s2  "Horga 2022" 21 1.46 0.84 28 1.46 0.84 //less  "Horga 2022" 21 1.62 0.84 28 1.62 0.84 //less  "Horga 2022" 21 1.86 0.84 28 1.86 0.84 //less  "Shimozaki 2018" 12 1.33 0.62 12 1.08 0.28 //less  "Shimozaki 2018" 12 1.17 0.37 12 1.00 0.84 //less  "Shimozaki 2018" 12 1.08 0.28 12 1.00 0.84 //less  "Shimozaki 2018" 12 1.83 0.69 12 1.08 0.28 //less  "Shimozaki 2018" 12 1.25 0.60 12 1.00 0.84 //less  "Telles 2016" 14 1.57 0.49 20 1.70 0.59 //less  end  format %9.2f m1  format %9.2f s1  format %9.2f m2  format %9.2f s2  meta esize n1 m1 s1 n2 m2 s2, studylabel(study) esize(hedges) eslabel(Hedges' g)  encode(study), gen(study2)  gen _meta_var =((n1+n2)/(n1*n2))+((_meta_es^2)/(2*(n1+n2-2)))  //Meta-analysis: ρ=0.8  ***negative's swapped  clear  input str50 study es se  "Horga 2022" -0.00 0.18  "Shimozaki 2018" -.52 0.19  "Telles 2016" 0.23 0.34  end  format %9.0f es  format %9.2f se  meta set es se, studylabel(study) eslabel(Hedges' g)  meta summarize, predint se(khartung)  meta forestplot, esrefline(lcolor(gs12) lpattern(dash)) nullrefline(favorsleft("Favours exercise", al(baseline) height(45)) favorsright("Favours control", al(baseline) height(45))) xline(0, lcolor(gs7) lpattern(solid)) title("Exercise follow up vs baseline") xlab(-2(1)3) predint(,pstyle(ci) lc(red))se(khartung)  //Sensitivity analysis: ρ=0.0  clear  input str50 study es se  "Horga 2022" 0.00 0.17  "Shimozaki 2018" 0.52 0.19  "Telles 2016" -0.23 0.34  end  format %9.0f es  format %9.2f se  meta set es se, studylabel(study) eslabel(Hedges' g)  meta summarize, cformat(%9.2f) se(khartung)  //Sensitivity analysis: ρ=0.2  clear  input str50 study es se  "Horga 2022" 0.00 0.17  "Shimozaki 2018" 0.52 0.19  "Telles 2016" -0.23 0.34  end  format %9.0f es  format %9.2f se  *********************************************************************************************************************************************************************  **#4 Cohort, INT: Any exercise compared to control, OUTCOME: binary Pfirrmann degeneration, n=2,  *********************************************************************************************************************************************************************  //Calculate effect estimates  // #4 a) Meta-analysis with N as number of PARTICIPANTS, n=2  clear  input str50 study int_yes int_no con_yes con_no  "Telles 2016" 0 14 0 10  "Witwit 2018 & 2022" 19 11 4 12  end  meta esize int_yes int_no con_yes con_no, studylabel(study)  //Meta-analysis  meta summarize, eform(Odds ratio) random(ebayes) se(khartung) predint  meta forestplot, esrefline(lcolor(gs12) lpattern(dash)) nullrefline(favorsleft("Favours exercise", al(baseline) height(40)) favorsright("Favours control", al(baseline) height(40))) columnopts(_data1, supertitle(Intervention)) columnopts(_data2, supertitle(Control)) xline(0, lcolor(gs7) lpattern(solid)) title("Prevalence of Pfirrmann degneration any exercsie vs baseline control") eform(Odds ratio) random(ebayes) se(khartung) xlab(minmax 1 100000)  //Sensitivity analysis: leave-one-out  clear  input str50 study int_yes int_no con_yes con_no  "Telles 2016" 0 14 0 10  "Witwit 2018 & 2022" 19 11 4 12  end  meta esize int_yes int_no con_yes con_no, studylabel(study)  meta summarize, eform(Odds ratio) random(ebayes) leaveoneout se(khartung)  *********************************************************************************************************************************************************************  **#5 Cohort, INT: Any exercise compared to baseline as control, OUTCOME: binary Pfirrmann degeneration, n=5,  *********************************************************************************************************************************************************************  //Calculate effect estimates  // #4 Meta-analysis with N as number of PARTICIPANTS or IVDS, n=5  clear  input str50 study int_yes int_no con_yes con_no  "Burnett 1996" 15 80 5 90  "Koyama 2022" 24 27 23 28  "Shimozaki 2018" 4 56 0 60  "Telles 2016" 0 14 7 13  "Witwit 2018 & 2022" 19 11 16 14  end  meta esize int_yes int_no con_yes con_no, studylabel(study)  //Meta-analysis  meta summarize, eform(Odds ratio) random(ebayes) predint  meta forestplot, esrefline(lcolor(gs12) lpattern(dash)) nullrefline(favorsleft("Favours exercise", al(baseline) height(40)) favorsright("Favours control", al(baseline) height(40))) columnopts(_data1, supertitle(Exercise)) columnopts(_data2, supertitle(Control)) xline(0, lcolor(gs7) lpattern(solid)) title("Prevalence of Pfirrmann degneration any exercsie vs baseline control") eform(Odds ratio) random(ebayes) xlab(minmax 1)  //Sensitivity analysis: leave-one-out  clear  input str50 study int_yes int_no con_yes con_no  "Burnett 1996" 15 80 5 90  "Koyama 2022" 24 27 23 28  "Shimozaki 2018" 4 56 0 60  "Telles 2016" 0 14 7 13  "Witwit 2018 & 2022" 19 11 16 14  end  meta esize int_yes int_no con_yes con_no, studylabel(study)  meta summarize, eform(Odds ratio) random(ebayes) leaveoneout  ********************************************************************************************************************************************************************  **#5(b) Cohort, INT: Any exercise compared to baseline as control, OUTCOME: signal intensity reduction, n=3,  *********************************************************************************************************************************************************************  //Calculate effect estimates  // #4 Meta-analysis with N as number of PARTICIPANTS or IVDS, n=5  clear  input str50 study int_yes int_no con_yes con_no  "Feuerriegel 2025" 21 42 10 53  "Witwit 2022" 20 10 20 10  "Baranto 2006" 4 15 3 13  end  meta esize int_yes int_no con_yes con_no, studylabel(study)  //Meta-analysis  meta summarize, eform(Odds ratio) random(ebayes) predint  meta forestplot, esrefline(lcolor(gs12) lpattern(dash)) nullrefline(favorsleft("Favours exercise", al(baseline) height(40)) favorsright("Favours control", al(baseline) height(40))) columnopts(_data1, supertitle(Exercise)) columnopts(_data2, supertitle(Control)) xline(0, lcolor(gs7) lpattern(solid)) title("Prevalence of Pfirrmann degneration any exercsie vs baseline control") eform(Odds ratio) random(ebayes) xlab(minmax 1)  //Sensitivity analysis: leave-one-out  clear  input str50 study int_yes int_no con_yes con_no  "Feuerriegel 2025" 21 42 10 53  "Witwit 2022" 20 10 20 10  "Baranto 2006" 4 15 3 13  end  meta esize int_yes int_no con_yes con_no, studylabel(study)  meta summarize, eform(Odds ratio) random(ebayes) leaveoneout  ************************************************************************************  **#6 Cross-sectional, INT: Any exercise vs control, OUTCOME: IVD signal intensity reuction, n=5,  *********************************************************************************************************************************************************************  clear  input str50 study int_yes int_no con_yes con_no  "Baranto 2009" 35 1 10 0  "Hellstrom 1990 & Sward 1991" 18 6 5 11  "Maurer 2011" 4 18 2 20  "Thoreson 2017" 11 5 12 16  "Witwit 2018 & 2022" 45 30 11 16  end  meta esize int_yes int_no con_yes con_no, studylabel(study)  //Meta-analysis  meta summarize, eform(Odds ratio) random(ebayes) predint  meta forestplot, esrefline(lcolor(gs12) lpattern(dash)) nullrefline(favorsleft("Favours exercise", al(baseline) height(40)) favorsright("Favours control", al(baseline) height(40))) columnopts(_data1, supertitle(Intervention)) columnopts(_data2, supertitle(Control)) xline(0, lcolor(gs7) lpattern(solid)) title("Prevalence of IVD signal intensity reduction any exercsie vs baseline control") eform(Odds ratio) random(ebayes) xlab(minmax 0.0 1)  //Sensitivity analysis: leave-one-out  clear  input str50 study int_yes int_no con_yes con_no  "Baranto 2009" 35 1 10 0  "Hellstrom 1990 & Sward 1991" 18 6 5 11  "Maurer 2011" 4 18 2 20  "Thoreson 2017" 11 5 12 16  "Witwit 2018 & 2022" 45 30 11 16  end  meta esize int_yes int_no con_yes con_no, studylabel(study)  meta summarize, eform(Odds ratio) random(ebayes) leaveoneout se(khartung)  ************************************************************************************  **#7 Cross-sectional, INT: Any exercise vs control, OUTCOME: any IVD height reduction , n=3  *********************************************************************************************************************************************************************  clear  input str50 study int_yes int_no con_yes con_no  "Baranto 2009" 32 4 9 1  "Thoreson 2017" 8 8 0 28  "Witwit 2018 & 2022" 24 51 0 27  end  meta esize int_yes int_no con_yes con_no, studylabel(study)  //Meta-analysis  meta summarize, eform(Odds ratio) random(ebayes) se(khartung) predint  meta forestplot, esrefline(lcolor(gs12) lpattern(dash)) nullrefline(favorsleft("Favours exercise", al(baseline) height(40)) favorsright("Favours control", al(baseline) height(40))) columnopts(_data1, supertitle(Intervention)) columnopts(_data2, supertitle(Control)) xline(0, lcolor(gs7) lpattern(solid)) title("Prevalence of any IVD height reduction any exercsie vs baseline control") eform(Odds ratio) random(ebayes) xlab(minmax 0.0 1) se(khartung)  //Sensitivity analysis: leave-one-out  clear  input str50 study int_yes int_no con_yes con_no  "Baranto 2009" 32 4 9 1  "Thoreson 2017" 8 8 0 28  "Witwit 2018 & 2022" 24 51 0 27  end  meta esize int_yes int_no con_yes con_no, studylabel(study)  meta summarize, eform(Odds ratio) random(ebayes) se(khartung) leaveoneout  ************************************************************************************  **#8 Cross-sectional, INT: Any exercise vs control, OUTCOME: >50 % IVD height reduction , n=3  *********************************************************************************************************************************************************************  clear  input str50 study int_yes int_no con_yes con_no  "Granhed 1988" 17 28 23 693  "Hellstrom 1990 & Sward 1991" 11 122 2 28  end  meta esize int_yes int_no con_yes con_no, studylabel(study)  //Meta-analysis  meta summarize, eform(Odds ratio) random(ebayes) se(khartung) predint  meta forestplot, esrefline(lcolor(gs12) lpattern(dash)) nullrefline(favorsleft("Favours exercise", al(baseline) height(40)) favorsright("Favours control", al(baseline) height(40))) columnopts(_data1, supertitle(Intervention)) columnopts(_data2, supertitle(Control)) xline(0, lcolor(gs7) lpattern(solid)) title("Prevalence of >50% IVD height signal reduction any exercsie vs baseline control") eform(Odds ratio) random(ebayes) xlab(minmax 0.0 1 100000000) se(khartung)  //Sensitivity analysis: leave-one-out  clear  input str50 study int_yes int_no con_yes con_no  "Granhed 1988" 17 28 23 693  "Hellstrom 1990 & Sward 1991" 11 122 2 28  end  meta esize int_yes int_no con_yes con_no, studylabel(study)  meta summarize, eform(Odds ratio) random(ebayes) leaveoneout se(khartung) |
| --- |

# Supplement F: Operationalisation of GRADE criteria

| Criteria | Operationalisation |
| --- | --- |
| **Risk of bias** | Downgrade one level if applicable:   - ≥50% of studies at high risk of bias   Downgrade two levels if applicable:   - ≥75% of studies at high risk of bias |
| **Inconsistency** | If heterogeneity unexplained, downgrade one level if applicable:   - Some concerns per figure below   If heterogeneity unexplained, downgrade two levels if applicable:   - Major concerns per figure below   **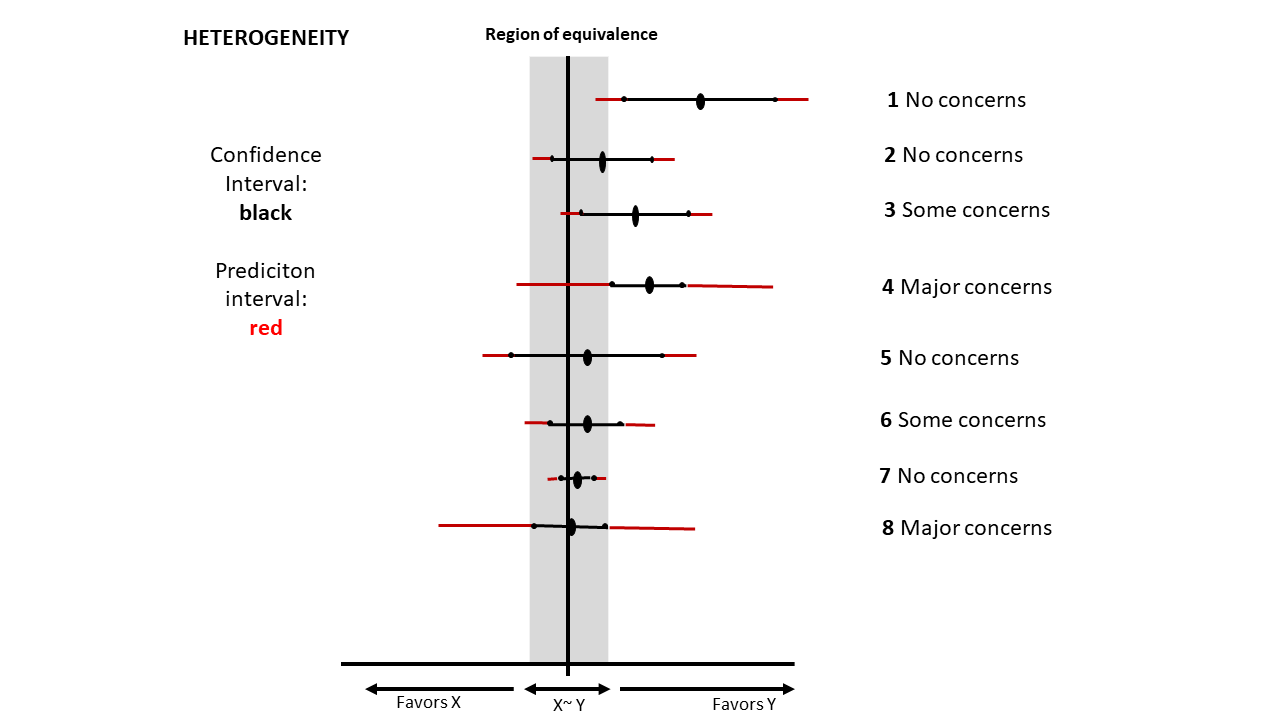**  *Region of equivalence defined as g=-0.5 to g=0.5 and OR=0.8 to OR=1.25* |
| **Indirectness** | Not considered as eligibility were defined per PECO framework |
| **Imprecision** | Downgrade one level if applicable:   - Some concerns per figure below   Downgrade two levels if applicable:   - Major concerns per figure below   **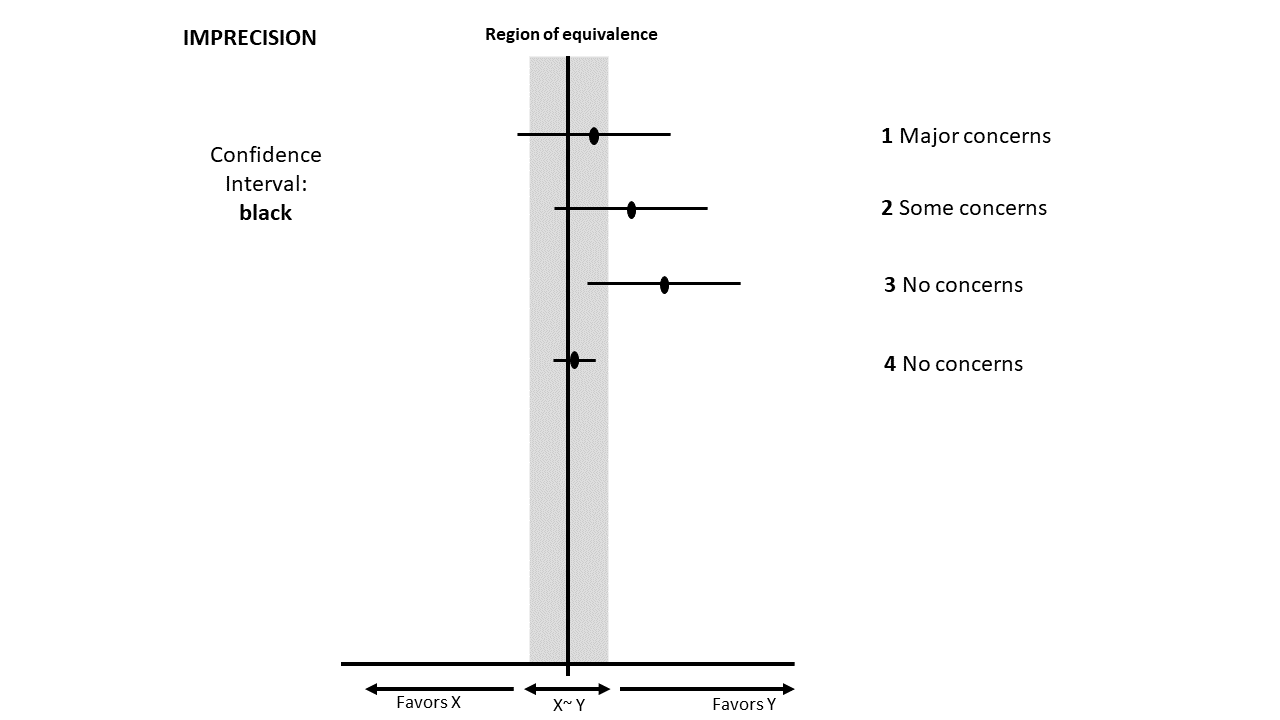**  *Region of equivalence defined as g=-0.5 to g=0.5 and OR=0.8 to OR=1.25* |
| **Publication bias** | Downgrade one level if any applicable:   - Funnel plot asymmetry with ≥10 studies - ≥50% of studies with conflicts of interest |

# Supplement G: Reasons for exclusion at full-text screening

| **First author** | **Year** | **Title** | **Reason** |
| --- | --- | --- | --- |
| Sekine | 2021 | Lumbar intervertebral disc degeneration does not affect muscle synergy for rowing activities | No relevant outcome |
| Simson | 2017 | Optimising conservative management of chronic low back pain: study protocol for a randomised controlled trial | Study design |
| Abdalkader | 2020 | MRI-detected spinal disc degenerative changes in athletes participating in the Rio de Janeiro 2016 Summer Olympics games. | No relevant comparator |
| Belavy | 2015 | Public title: Optimising conservative management of chronic low back pain Scientific title: In patients with low back pain, does general spine and body conditioning, compared to manual therapy, improve intervertebral disc characteristics? | Duplicate of same publication |
| Alyas | 2007 | MRI findings in the lumbar spines of asymptomatic, adolescent, elite tennis players. | No relevant comparator |
| Annear | 1992 | Pars interarticularis stress and disc degeneration in cricket's potent strike force: the fast bowler. | No relevant comparator |
| Arnold | 1974 | Rehabilitation results of patients with damaged lumbar intervertebral discs (author's transl) | Study design |
| Atalay | 2012 | Deconditioning in chronic low back pain: might there be a relationship between fitness and magnetic resonance imaging findings? | No relevant comparator |
| Baltimore | 2009 | Back pain and disc degeneration among horseback riders. | Study design |
| Bartolozzi | 1991 | The incidence of disk changes in volleyball players. The magnetic resonance findings. | No relevant comparator |
| Battié | 2002 | Occupational driving and lumbar disc degeneration: a case-control study. | No relevant comparator |
| Berry | 2017 | Noninvasive assessment of lumbar muscle architecture in active-duty marines | Study design |
| Bezuglov | 2021 | Asymptomatic Degenerative Changes in the Lumbar Spine Among Professional Soccer Players | No relevant comparator |
| Bowden | 2017 | Moderate-vigorous activity and sedentary time are indicators of IVD health | Study design |
| Brox | 2003 | Randomized clinical trial of lumbar instrumented fusion and cognitive intervention and exercises in patients with chronic low back pain and disc degeneration. | No relevant outcome |
| Burns | 2006 | Low Back Pain in a Female Varsity Ice-Hockey Player. | Study design |
| Cannon | 1984 | Back pain in athletes. | No relevant comparator |
| Cassel | 2015 | Load-dependant segmental spinal stability in functional MRI of adolescent athletes with spondylolisthesis with and without low back pain | No relevant comparator |
| Castillo | 2018 | Shock attenuation in the human lumbar spine during walking and running. | No relevant comparator |
| Cha | 2017 | A longitudinal single-blind randomized controlled study of UGCS with the le exercise in LBP | Study design |
| Chen | 2006 | Survey and analysis of lumbar trauma in 308 athletes who participated in the national championship contest of badminton in 2003 | No relevant comparator |
| Connolly | 2020 | Lumbar spine abnormalities and facet joint angles in asymptomatic elite junior tennis players. | No relevant comparator |
| Compere | 1968 | Generalized intervertebral disk degeneration throughout the lumbar spine with secondary osteoarthritis. | Study design |
| Dmitriyev | 2020 | Training process impact on vertebral column state of junior athletes. | No relevant comparator |
| Demirel | 2017 | Regression of lumbar disc herniation by physiotherapy. Does non-surgical spinal decompression therapy make a difference? Double-blind randomized controlled trial. | No relevant exposture |
| Elliott | 1993 | Disc degeneration and the young fast bowler in cricket | No relevant comparator |
| Elliott | 2002 | Disk degeneration and fast bowling in cricket: an intervention study. / Etude de la degenerescence discale et du lancer en cricket. | Duplicate of same publication |
| Elliott | 1992 | The influence of fast bowling and physical factors on radiologic features in high performance young fast bowlers. | No relevant comparator |
| Elliott | 2002 | Disk degeneration and fast bowling in cricket: an intervention study. | No relevant comparator |
| Enoki | 2022 | The prevalence of spondylolysis and intervertebral disc degeneration in male pole vaulters. | No relevant comparator |
| Fabrice K | 2013 | The lumbar spines of professional beach volleyball players-high incidence of spondylolysis | No relevant comparator |
| Goldstein | 1991 | Spine injuries in gymnasts and swimmers. An epidemiologic investigation | No relevant outcome |
| Gopalan | 2018 | Lumbosacral transitional vertebra-related low back pain: resolving the controversy. | No relevant outcome |
| HADLEY | 1964 | Exercises in the treatment of lumbar intervertebral disc protrusions. | Study design |
| Hanımoğlu | 2019 | Effects of modic type 1 changes in the vertebrae on low back pain. | No relevant exposture |
| Hardcastle | 1992 | Spinal abnormalities in young fast bowlers. | No relevant comparator |
| Hassett | 2003 | Risk factors for progression of lumbar spine disc degeneration: the Chingford Study. | No relevant outcome |
| Hasson | 2006 | Former athletes shown not to suffer more lumbar back pain than nonathletes. | Study design |
| Hayashi | 2011 | Lumbar intervertebral disk degeneration in Japanese male volleyball players belonging to the national team. | Study design |
| Horlyck | 1979 | Spondylolysis and disintegration of the lumbar discs in competitive athletes | Full text not available |
| Hupli | 1997 | Height changes among chronic low back pain patients during intense physical exercise. | No relevant outcome |
| Hupli | 1997 | Height changes among chronic low back pain patients during intense physical exercise. / Modification de la taille chez des patients ayant des problemes chroniques de dos lors d' un exercice physique intense. | No relevant outcome |
| Iwai | 2016 | Asymmetrical and smaller size of trunk muscles in combat sports athletes with lumbar intervertebral disc degeneration. | No relevant comparator |
| Iwamoto | 2005 | Relationship between radiographic abnormalities of lumbar spine and incidence of low back pain in high school rugby players: a prospective study | No relevant comparator |
| Külling | 2014 | High prevalence of disc degeneration and spondylolysis in the lumbar spine of professional beach volleyball players. | No relevant comparator |
| Khanzadeh | 2020 | The effect of suspension and conventional core stability exercises on characteristics of intervertebral disc and chronic pain in office staff due to lumbar herniated disc. | No relevant outcome |
| Kojima | 2018 | Lumbar intervertebral disc degeneration in professional surfers | No relevant comparator |
| Koyama | 2012 | Physical characteristics of male collegiate rubber baseball players with disc degeneration. | No relevant comparator |
| Koyama | 2013 | Categorical abnormalities and low back pain in gymnasts. | No relevant comparator |
| Koyama | 2013 | Anterior Limbus Vertebra and Intervertebral Disk Degeneration in Japanese Collegiate Gymnasts. | No relevant comparator |
| Koyama | 2013 | Radiological abnormalities and low back pain in gymnasts. | No relevant comparator |
| Koyama | 2013 | Are the determinants of vertebral endplate changes and severe disc degeneration in the lumbar spine the same? A magnetic resonance imaging study in middle-aged male workers. | No relevant exposture |
| Lee | 2016 | The effect of sling exercise on sagittal lumbosacral angle and intervertebral disc area of chronic low back pain patients. | No relevant outcome |
| Lundin | 2001 | Back pain and categorical changes in the thoraco-lumbar spine of athletes. A long-term follow-up. | No relevant outcome |
| Lundin | 2001 | Back pain and categorical changes in the thoraco-lumbar spine of athletes. A long-term follow-up: Back pain in athletes | No relevant comparator |
| Montoliu | 1994 | Disco intervertebral y deporte. / Intervertebral disk and sport. | No relevant comparator |
| Moreira | 2021 | Impacto do exercÃcio fÃsico na qualidade do disco intervertebral da coluna lombar | Study design |
| Mutoh | 1977 | Low back pain in butterfliers | No relevant comparator |
| Nagashima | 2013 | Risk factors for lumbar disc degeneration in high school American football players: a prospective 2-year follow-up study. | No relevant comparator |
| Demirel | 2016 | Comparison of different treatment methods in lumbal disc herniation treatment | No relevant outcome |
| Demirel | 2016 | Remission of lumbar disc herniation by physiotherapy | No relevant outcome |
| Gullbrand | 2022 | The role of disc nutrition in the etiology and clinical treatment of disc degeneration | No relevant exposture |
| Neubert | 2014 | Validity and reliability of computerized measurement of lumbar intervertebral disc height and volume from magnetic resonance images. | No relevant exposture |
| Ogon | 2001 | Radiologic abnormalities and low back pain in elite skiers. | No relevant outcome |
| Ogurkowska | 2007 | Pathological changes in lumbar-sacral intervertebral discs in professional rowers. | No relevant comparator |
| Ogurkowska | 2016 | Pathological changes in the lumbar intervertebral discs among professional field hockey players. | No relevant comparator |
| Ong | 2003 | A pilot study of the prevalence of lumbar disc degeneration in elite athletes with lower back pain at the Sydney 2000 Olympic Games. | No relevant comparator |
| Pandya M | 2014 | Lumbar spine distraction exercise-surprisingly innovation in conservative treatment of degenerative lumbar spine disorder | Study design |
| Papic M | 2017 | Relation between grades of intervertebral disc degeneration and occupational activities of patients with lumbar disc herniation | No relevant exposture |
| Peterhans | 2020 | High Rates of Overuse-Related Structural Abnormalities in the Lumbar Spine of Youth Competitive Alpine Skiers: A Cross-sectional MRI Study in 108 Athletes. | No relevant comparator |
| Quittner | 2018 | Intervertebral disc status is associated with vertebral marrow adipose tissue and muscular endurance. | Duplicate of same publication (Study) |
| Rachbauer | 2001 | Radiographic Abnormalities in the Thoracolumbar Spine of Young Elite Skiers | No relevant comparator |
| Rajeswaran | 2014 | MRI findings in the lumbar spines of asymptomatic elite junior tennis players. | No relevant comparator |
| Ranson | 2010 | Injuries to the lower back in elite fast bowlers: acute stress changes on MRI predict stress fracture. | No relevant comparator |
| Refior | 1970 | [Vertebral column and competitive gymnastics. Changes of the vertebral bodies and of the intervertebral discs in children and adolescents]. | No relevant outcome |
| Rodriguez-Soto | 2017 | The effect of training on lumbar spine posture and intervertebral disc degeneration in active-duty Marines. | No relevant comparator |
| Renjun | 2025 | Causal relationship between sedentary and physical activity levels in the Oswestry disability index score and intervertebral disc degeneration | No relevant outcome |
| Rozan | 2016 | Influence of Physiological Loading on the Lumbar Spine of National Level Athletes in Different Sports. | No relevant outcome |
| Salminen | 1993 | Magnetic resonance imaging findings of lumbar spine in the young: correlation with leisure time physical activity, spinal mobility, and trunk muscle strength in 15-year-old pupils with or without low-back pain. | No relevant outcome |
| Salo | 2022 | Association between severe lumbar disc degeneration and self-reported occupational physical loading. | No relevant exposture |
| Samartzis | 2011 | A population-based study of juvenile disc degeneration and its association with overweight and obesity, low back pain, and diminished functional status. | No relevant exposture |
| Savage | 1997 | The relationship between the magnetic resonance imaging appearance of the lumbar spine and low back pain, age and occupation in males. | No relevant exposture |
| Schroeder | 2014 | The role of athletic activity on structural lumbar abnormalities in adolescent patients with symptomatic low back pain | Study design |
| Stith | 1990 | Exercise and the intervertebral disc. | Study design |
| Sun | 2010 | Case-control study of the risk factors of lumbar intervertebral disc herniation in 5 northern provinces of China | language (not German, not French, not English) |
| Sward | 1990 | Back pain and radiologic changes in the thoraco-lumbar spine of athletes | No relevant comparator |
| Swärd | 1990 | The back of the young top athlete: symptoms, muscle strength, mobility, anthropometric and categorical findings. | Study design |
| Takatalo | 2010 | Overweight and unhealthy behaviour predict lumbar disc degeneration in young males | Study design |
| Takatalo | 2012 | Body mass index and smoking predict lumbar disk degeneration among young finnish males | Study design |
| Takatalo | 2017 | Association between adolescent sport activities and lumbar disk degeneration among young adults. | No relevant exposture |
| Takatalo | 2013 | Body mass index is associated with lumbar disc degeneration in young Finnish males: subsample of Northern Finland birth cohort study 1986. | No relevant outcome |
| Teichtahl | 2015 | Physical inactivity is associated with reduced lumbar intervertebral disc height, high fat content of paraspinal muscles and low back pain and disability | No relevant outcome |
| Toyooka K | 2019 | High incidence rate of lumbar spinal disease among child and adolescent weightlifting athletes: A prospective 4-year cohort study | Study design |
| Toyooka | 2019 | High incidence rate of lumbar spinal disease among child and adolescent weightlifting athletes: a prospective 4-year cohort study...american orthopaedic society for sports medicine annual meeting, july 11-14, 2019, boston, massachusetts | Study design |
| Udby | 2019 | Which MRI findings are associated with long-term disability in low back pain patients? | Study design |
| Udby | 2021 | The association of mri findings and long-term disability in patients with chronic low back pain. | No relevant exposture |
| Videman | 1990 | 1990 volvo award in clinical sciences. lumbar spinal pathology in cadaveric material in relation to history of back pain, occupation, and physical loading | No relevant exposture |
| Videman | 1995 | The long-term effects of physical loading and exercise lifestyles on back-related symptoms, disability, and spinal pathology among men. | No relevant comparator |
| Videman | 2006 | Determinants of the progression in lumbar degeneration: a 5-year follow-up study of adult male monozygotic twins | No relevant exposture |
| Videman | 2007 | The effects of anthropometrics, lifting strength, and physical activities in disc degeneration. | No relevant comparator |
| Wang | 2016 | The characteristics of spino-pelvic sagittal parameters and obesity factors for adolescents with lumbar disc herniation | No relevant outcome |
| Wasserman | 2018 | Evaluation of spine MRIs in athletes participating in the Rio de Janeiro 2016 Summer Olympic Games. | No relevant outcome |
| Yu-ju Hung | 2014 | The dose-response relationship between cumulative lifting load and lumbar disk degeneration based on magnetic resonance imaging findings. | No relevant exposture |
| Zhao P | 1996 | The biomechanical significance of herniated lumbar intervertebral disk: a clinical comparison analysis of 22 multiple and 39 single segments in patients with lumbar intervertebral disk herniation. | No relevant exposture |
| Zhu S | 2004 | A long-term follow-up study for treatment of intervertebral disc herniation with rehabilitative methods | No relevant exposture |

**Supplement H:** Forest plots for primary and secondary synthesis.


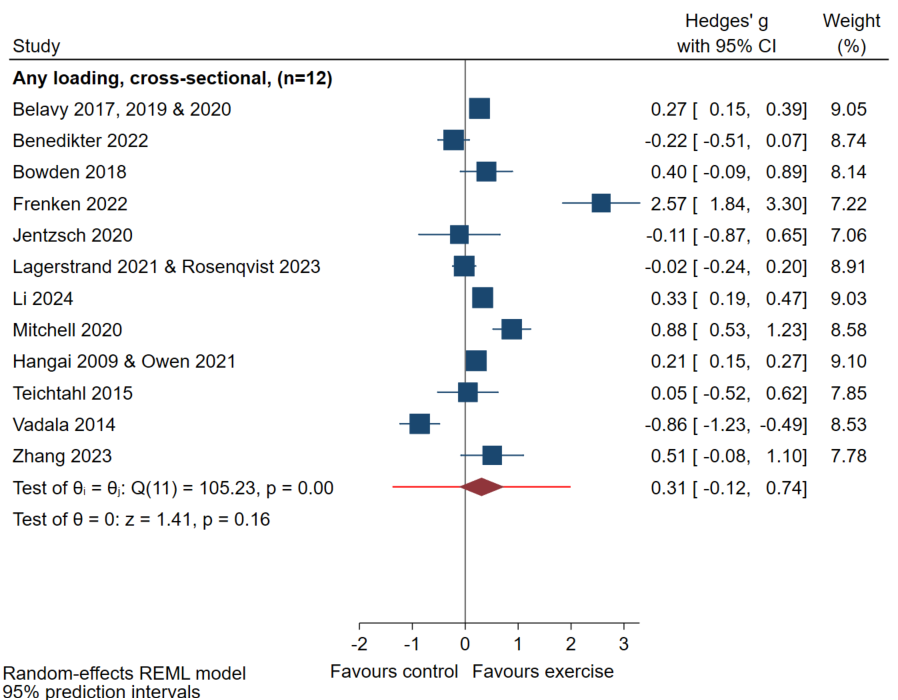
 **Figure 1:** Pairwise random-effects restricted maximum likelihood meta-analysis of continuous IVD outcomes from combined physical loading compared to control from cross-sectional studies. Red line represents prediction interval

**
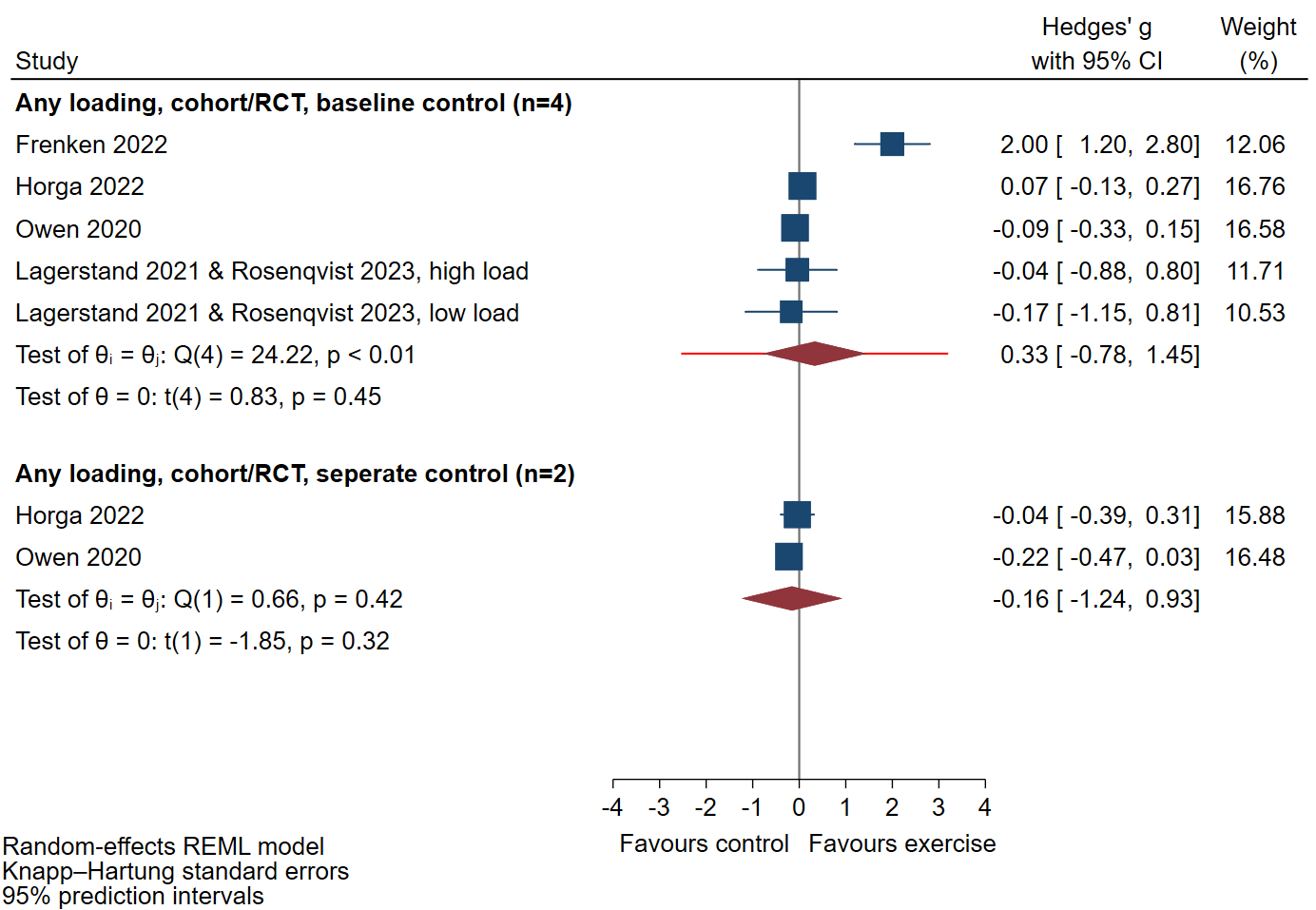
**

**Figure 2:** Pairwise random-effects restricted maximum likelihood meta-analysis of continuous IVD outcomes from RCT or cohort studies with combined physical loading with Hartung-Knapp-Sidik-Jonkman adjustment for < five studies. Red line represents prediction


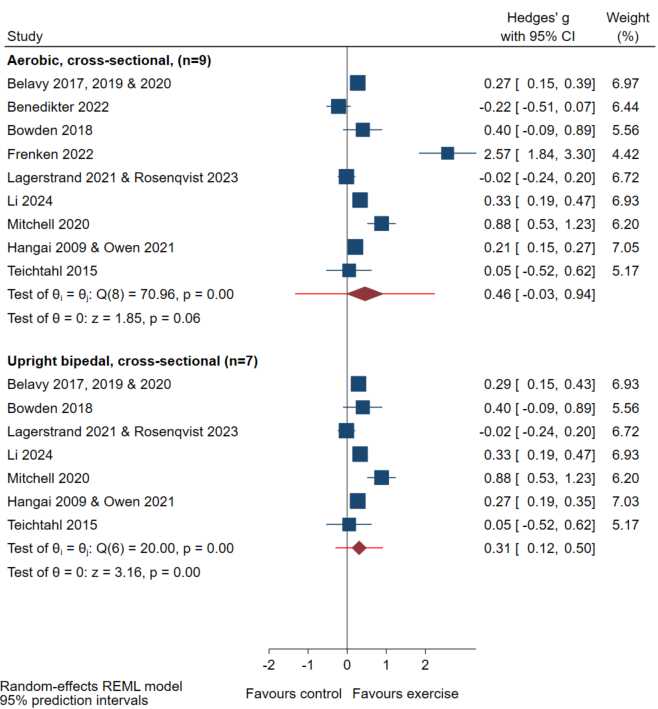
 **Figure 3:** Pairwise random-effects restricted maximum likelihood meta-analysis of continuous IVD outcomes from cross-sectional studies with physical loading sub groups with >five studies compared to control. Red line represents prediction interval.

**
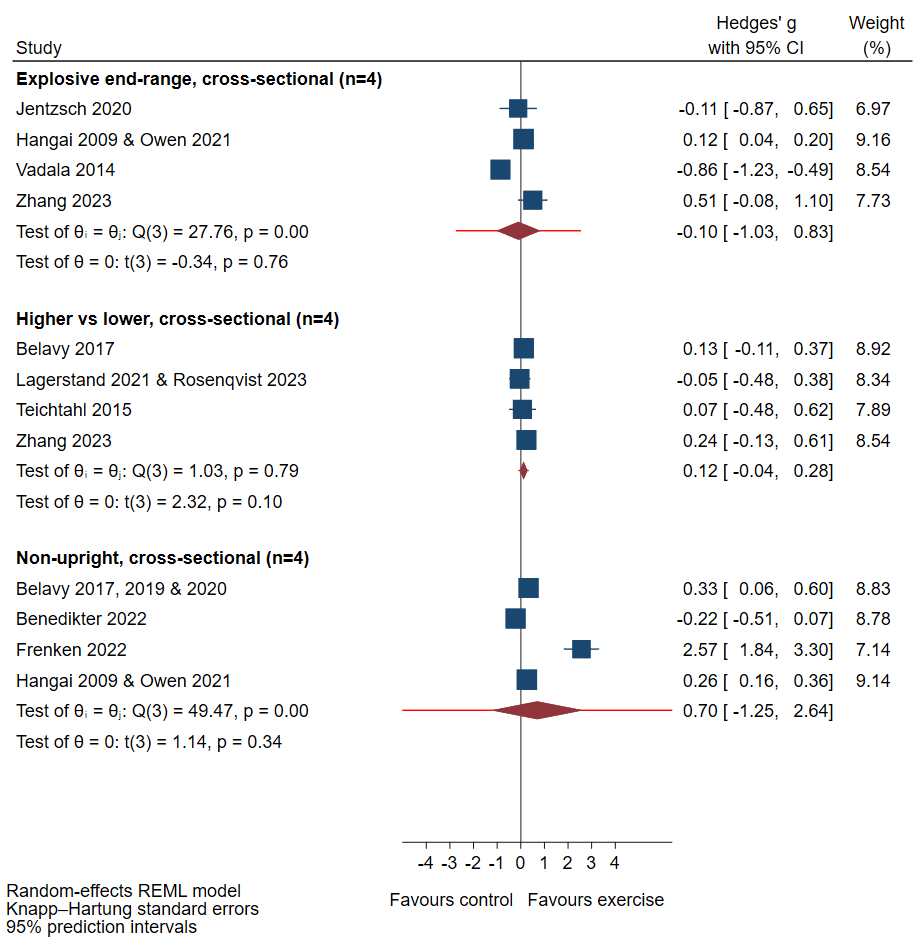
 Figure 4:** Pairwise random-effects restricted maximum likelihood meta-analysis of continuous IVD outcomes from cross-sectional studies for physical loading sub-groups with Hartung-Knapp-Sidik-Jonkman adjustment for < five studies. Red line represents prediction interval.

**
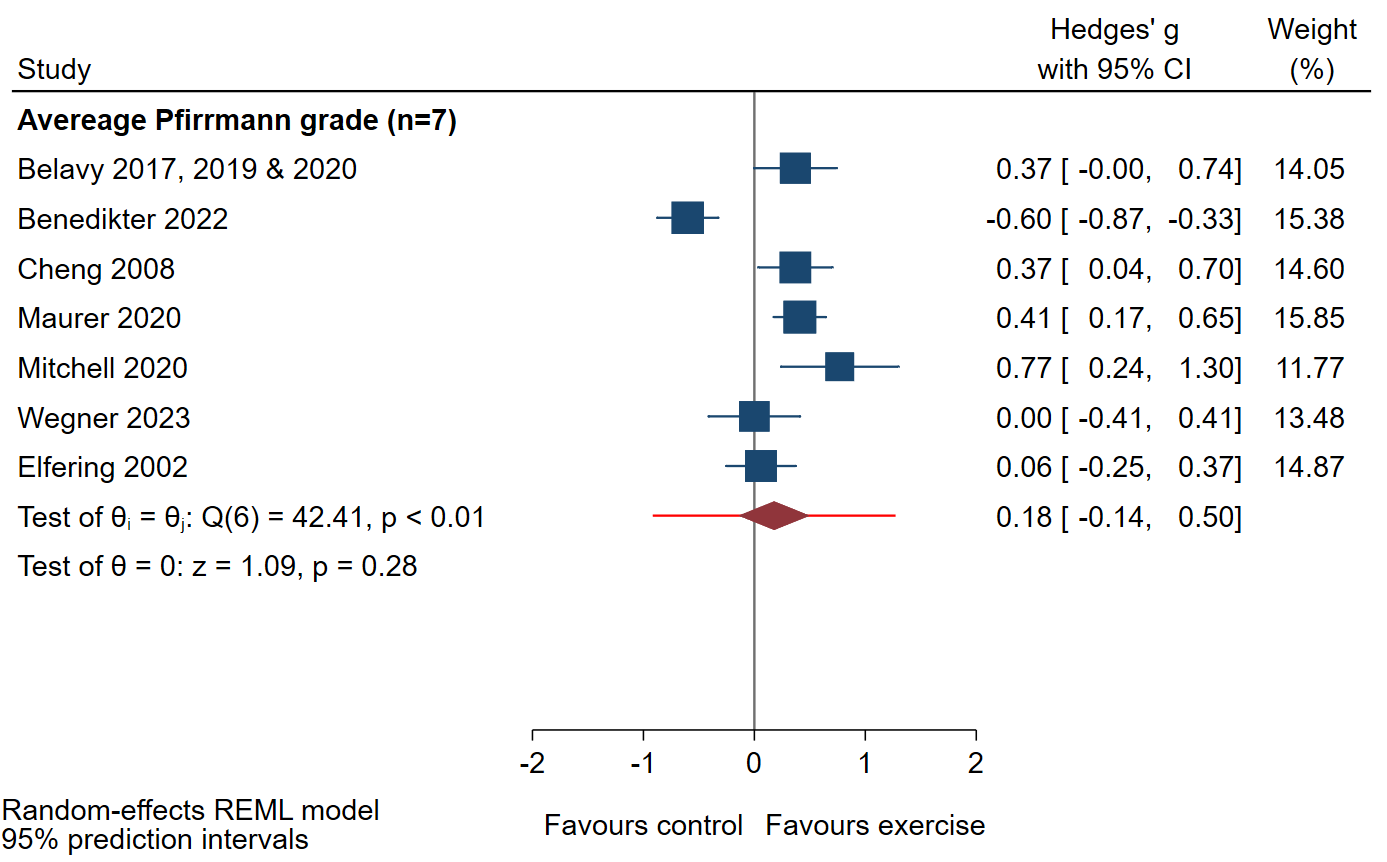
Figure 5:** Pairwise random-effects restricted maximum likelihood meta-analysis of average Pfirrmann grade from cross-sectional studies for combined physical loading. Red line represents prediction interval

**
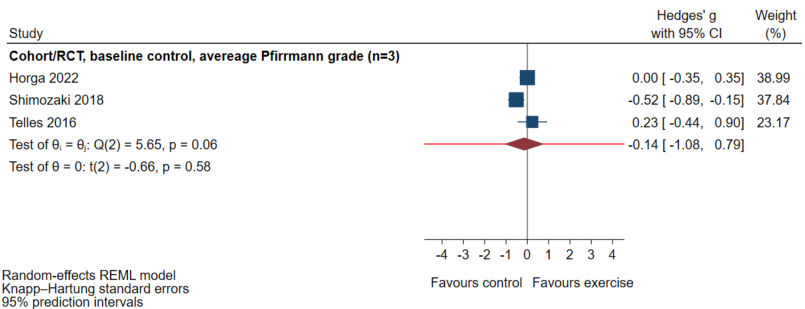
 Figure 6:** Pairwise random-effects restricted maximum likelihood meta-analysis of average Pfirrmann grade from cohort studies for combined physical loading with baseline a control with Hartung-Knapp-Sidik-Jonkman adjustment for < five studies. Red line represents prediction interval

**
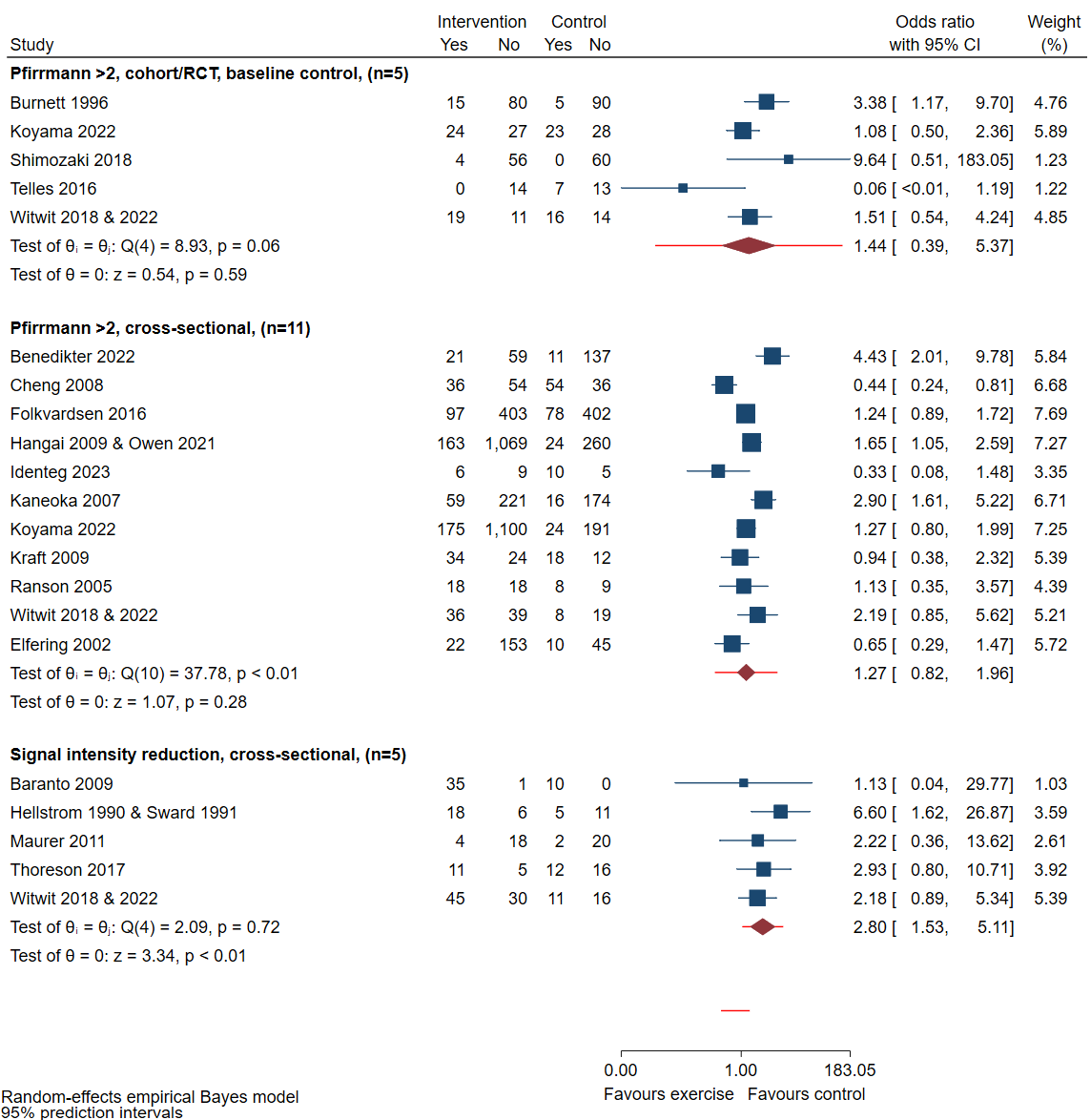
**



**Figure 7:** Pairwise random effects-meta-analysis of odds ratio with a Paule-Mandel estimator of categorical outcomes of IVD degeneration from cross-sectional and RCT/cohort studies for combined physical loading. Red line represents prediction interval.

**
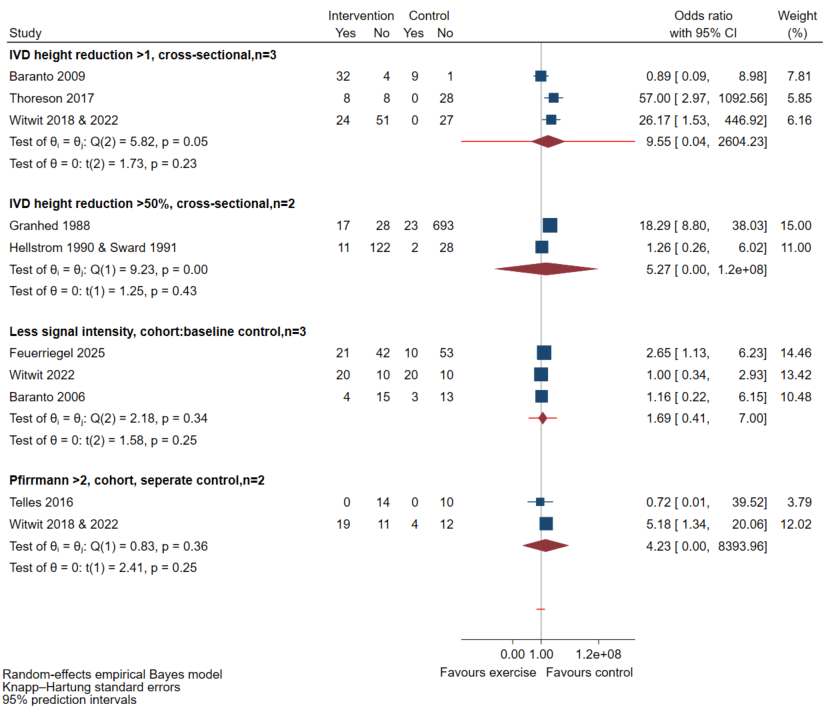
**

**Figure 8:** Pairwise random effects-meta-analysis of odds ratio with a Paule-Mandel estimator meta-analysis of categorical outcomes of IVD degeneration from cross-sectional and RCT/cohort studies for combined physical loading with Hartung-Knapp-Sidik-Jonkman adjustment for < five studies. Red line represents prediction interval.

# **Supplement I:** Random-effects meta-analysis sensitivity analyses for primary syntheses continuous IVD health and combined physical loading compared to control.

| Cross-sectional sensitivity analysis | Hedges’ g (95%CI) | P-value |
| --- | --- | --- |
| **Leave-one-out (restricted maximum likelihood; ρ=0.8)** |  |  |
| #1: Belavy 2017 & 2019 & 2020 | 0.32 (-0.16, 0.79) | 0.195 |
| #2: Benedikter 2022 | 0.36 (-0.10, 0.82) | 0.126 |
| #3: Bowden 2018 | 0.30 (-0.17, 0.78) | 0.208 |
| #4: Frenken 2022 | 0.14 (-0.13, 0.40) | 0.322 |
| #5: Hangai 2009 & Owen 2021 | 0.34 (-0.12, 0.80) | 0.145 |
| #6: Jentzsch 2020 | 0.34 (-0.13, 0.81) | 0.153 |
| #7: Lagerstand 2021 & Rosenqvist 2023 | 0.31 (-0.17, 0.79) | 0.203 |
| #8: Li 2024 | 0.26 (-0.20, 0.71) | 0.271 |
| #9: Mitchell 2020 | 0.32 (-0.16, 0.80) | 0.186 |
| #10: Teichtahl 2015 | 0.33 (-0.14, 0.80) | 0.163 |
| #11: Vadala 2014 | 0.41 (0.01, 0.81) | 0.044 |
| #12: Zhang 2023 | 0.29 (-0.17, 0.76) | 0.219 |
| **Assumed correlation (restricted maximum likelihood)** |  |  |
| #13: ρ=0.0 | 0.31 (-0.12, 0.74) | 0.157 |
| #14: ρ=0.2 | 0.31 (-0.12, 0.74) | 0.157 |
| #15: ρ=0.4 | 0.31 (-0.12, 0.74) | 0.157 |
| #16: ρ=0.6 | 0.31 (-0.12, 0.74) | 0.158 |
| #17: ρ=1.0 | 0.31 (-0.12, 0.74) | 0.158 |
| **Assumed correlation (robust variance)** |  |  |
| #18: ρ=0.0 | 0.32 (0.18, -0.17) | 0.177 |
| #19: ρ=0.2 | 0.32 (0.18, -0.17) | 0.177 |
| #20: ρ=0.4 | 0.32 (0.18, -0.17) | 0.177 |
| #21: ρ=0.6 | 0.32 (0.18, -0.17) | 0.177 |
| #22: ρ=0.8 | 0.32 (0.18, -0.17) | 0.177 |
| #23: ρ=1.0 | 0.32 (0.18, -0.17) | 0.177 |
| **Linearity assumption for age meta regression (robust variance estimation)** | | |
| #24: ρ=0.8 | 0.31 (0.19, -0.18) | 0.193 |
| **Linearity assumption for sex meta regression (robust variance estimation)** | | |
| #25: ρ=0.8 | 0.32 (0.18, -0.17) | 0.177 |
| **Cohort/RCT with separate control sensitivity analysis** | **Hedges’ g (95%CI)** | **P-value** |
| **Leave-one-out (restricted maximum likelihood; ρ=0.8)** |  |  |
| #1: Horga 2022 | -0.22 (-0.48, 0.04) | 0.091 |
| #2: Owen 2020 | -0.04 (-0.39, 0.31) | 0.824 |
| **Assumed correlation (restricted maximum likelihood)** |  |  |
| #3: ρ=0.0 | -0.16 (-1.24, 0.93) | 0.315 |
| #4: ρ=0.2 | -0.16 (-1.24, 0.93) | 0.315 |
| #5: ρ=0.4 | -0.16 (-1.24, 0.93) | 0.315 |
| #6: ρ=0.6 | -0.16 (-1.24, 0.93) | 0.315 |
| #7: ρ=1.0 | -0.16 (-1.24, 0.93) | 0.315 |
| **Normality assumption (robust variance)** |  |  |
| #8: ρ=0.8 | -0.17 (-1.19, 0.86) | 0.287 |
| **Cohort/RCT with baseline as control sensitivity analysis** | **Hedges’ g (95%CI)** | **P-value** |
| **Leave-one-out (restricted maximum likelihood; ρ=0.8)** |  |  |
| #1: Frenken 2022 | 0.00 (-0.15, 0.15) | 0.987 |
| #2: Horga 2022 | 0.42 (-1.24, 2.08) | 0.483 |
| #3: Lagerstand 2021 & Rosenqvist 2023, high load | 0.43 (-1.18, 2.03) | 0.461 |
| #4: Lagerstand 2021 & Rosenqvist 2023, low load | 0.45 (-1.12, 2.01) | 0.432 |
| #5: Owen 2020 | 0.46 (-1.16, 2.08) | 0.431 |
| **Assumed correlation (restricted maximum likelihood)** |  |  |
| #6: ρ=0.0 | 0.33 (-0.78, 1.45) | 0.454 |
| #7: ρ=0.2 | 0.33 (-0.78, 1.45) | 0.454 |
| #8: ρ=0.4 | 0.33 (-0.78, 1.45) | 0.454 |
| #9: ρ=0.6 | 0.33 (-0.78, 1.45) | 0.454 |
| #10: ρ=1.0 | 0.33 (-0.78, 1.45) | 0.454 |
| **Normality assumption (robust variance)** |  |  |
| #11: ρ=0.8 | 0.35 (-0.78, 1.48) | 0.435 |


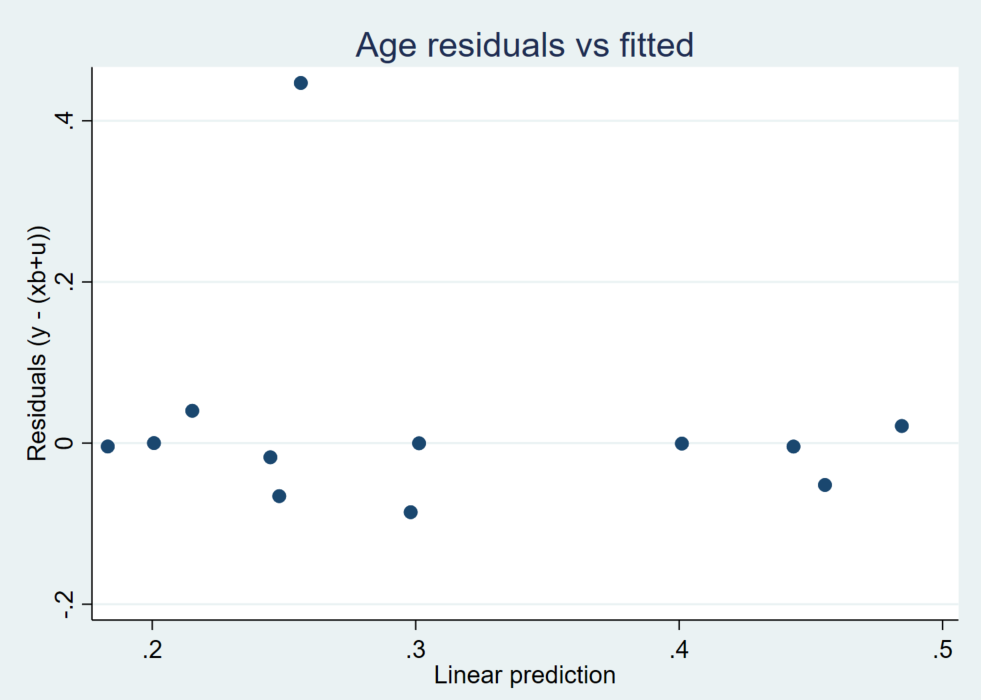


**Figure 1:** Residual versus fitted values for age meta regression

# Supplement J Random-effects meta-analysis sensitivity analyses for primary sub-group syntheses if continuous IVD health

1. **Upright bipedal physical loading compared to control**

| Cross-sectional study sensitivity analysis | Hedges’g (95%CI) | P-value |
| --- | --- | --- |
| **Leave-one-out (restricted maximum likelihood; ρ=0.8)** |  |  |
| #1: Belavy 2017, 2019& 2020 | 0.31 (0.07, 0.56) | 0.012 |
| #2: Bowden 2018 | 0.30 (0.08, 0.51) | 0.007 |
| #3: Hangai 2009 & Owen 2021 | 0.36 (0.20, 0.51) | <0.001 |
| #4: Lagerstrand 2021 | 0.25 (0.14, 0.35) | <0.001 |
| #5: Li 2024 | 0.32 (0.07, 0.56) | 0.011 |
| #6: Mitchell 2020 | 0.33 (0.12, 0.54) | 0.002 |
| #7: Teichtahl 2015 | 0.31 (0.06, 0.55) | 0.014 |
| **Assumed correlation (restricted maximum likelihood)** |  |  |
| #8: ρ=0.0 | 0.31 (0.12, 0.49) | 0.001 |
| #9: ρ=0.2 | 0.31 (0.12, 0.50) | 0.002 |
| #10: ρ=0.4 | 0.31 (0.12, 0.50) | 0.002 |
| #11: ρ=0.6 | 0.31 (0.12, 0.50) | 0.002 |
| **Normality assumption (robust variance)** |  |  |
| #12: ρ=0.8 | 0.25 (0.01, 0.07) | 0.015 |

1. **Non-upright, non-contact physical loading compared to control**

| Cross-sectional study sensitivity analysis | Hedges’ g (95%CI) | P-value |
| --- | --- | --- |
| **Leave-one-out (restricted maximum likelihood; ρ=0.8)** |  |  |
| #1: Belavy 2019, 2017 & 2020 | 0.84 (-2.8, 4.51) | 0.429 |
| #2: Benedikter 2022 | 1.02 (-2.21, 4.24) | 0.308 |
| #3: Frenken 2022 | 0.14 (-0.58, 0.86) | 0.496 |
| #4: Hangai 2009 & Owen 2021 | 0.86 (-2.78, 4.50) | 0.415 |
| **Assumed correlation (restricted maximum likelihood)** |  |  |
| #5: ρ=0.0 | 0.70 (-1.25, 2.64) | 0.336 |
| #6: ρ=0.2 | 0.70 (-1.25, 2.64) | 0.336 |
| #7: ρ=0.4 | 0.70 (-1.25, 2.64) | 0.336 |
| #8: ρ=0.6 | 0.70 (-1.25, 2.64) | 0.336 |
| #9: ρ=1.0 | 0.70 (-1.25, 2.64) | 0.336 |
| **Normality assumption (robust variance)** |  |  |
| #10: ρ=0.8 | 0.71 (-1.22, 2.64) | 0.325 |

1. **Extreme range-of-motion physical loading compared to control**

| Cross-sectional study sensitivity analysis | Hedges’ g (95%CI) | P-value |
| --- | --- | --- |
| **Leave-one-out (restricted maximum likelihood; ρ=0.8)** |  |  |
| #1: Hangai 2009 & Owen 2021 | -0.09 (-1.82, 1.64) | 0.841 |
| #2: Jentzsch 2020 | -0.18 (-1.93, 1.57) | 0.699 |
| #3: Vadala 2014 | 0.12 (-0.05, 0.30) | 0.089 |
| #4: Zhang 2023 | -0.28 (-1.60, 1.04) | 0.456 |
| **Assumed correlation (restricted maximum likelihood)** |  |  |
| #5: ρ=0.0 | -0.10 (-1.03, 0.83) | 0.756 |
| #6: ρ=0.2 | -0.10 (-1.03, 0.83) | 0.756 |
| #7: ρ=0.4 | -0.10 (-1.03, 0.83) | 0.756 |
| #8: ρ=0.6 | -0.10 (-1.03, 0.83) | 0.756 |
| #9: ρ=1.0 | -0.10 (-1.03, 0.83) | 0.756 |
| **Normality assumption (robust variance)** |  |  |
| #10: ρ=0.8 | -0.03 (-0.87, 0.81) | 0.911 |

1. **Aerobic physical loading compared to control**

| Cross-sectional study sensitivity analysis | Hedges’ g (95%CI) | P-value |
| --- | --- | --- |
| **Leave-one-out (restricted maximum likelihood; ρ=0.8)** |  |  |
| #1: Belavy 2017 & 2019 & 2020 | 0.49 (-0.07, 1.05) | 0.088 |
| #2: Benedikter 2022 | 0.54 (0.02, 1.06) | 0.040 |
| #3: Bowden 2018 | 0.47 (-0.09, 1.02) | 0.098 |
| #4: Frenken 2022 | 0.23 (0.02, 0.45) | 0.033 |
| #5: Lagerstand 2021 & Rosenqvist 2023 | 0.52 (-0.02, 1.06) | 0.059 |
| #6: Li 2024 | 0.41 (-0.13, 0.95) | 0.140 |
| #7: Mitchell 2020 | 0.50 (-0.06, 1.05) | 0.082 |
| #8: Hangai 2009 & Owen 2021 | 0.51 (-0.04, 1.05) | 0.067 |
| #9: Teichtahl 2015 | 0.48 (-0.08, 1.04) | 0.094 |
| **Assumed correlation (restricted maximum likelihood)** |  |  |
| #10: ρ=0.0 | 0.46 (-0.03, 0.94) | 0.064 |
| #11: ρ=0.2 | 0.46 (-0.03, 0.94) | 0.064 |
| #12: ρ=0.4 | 0.46 (-0.03, 0.94) | 0.064 |
| #13: ρ=0.6 | 0.46 (-0.03, 0.94) | 0.065 |
| #14: ρ=1.0 | 0.46 (-0.03, 0.94) | 0.065 |
| **Normality assumption (robust variance)** |  |  |
| #15: ρ=0.8 | 0.45 (0.12, -0.15) | 0.123 |

1. **Higher vs lower physical loading compared to control**

| Cross-sectional study sensitivity analysis | Hedges’ g (95%CI) | P-value |
| --- | --- | --- |
| **Leave-one-out (restricted maximum likelihood; ρ=0.8)** |  |  |
| #1: Belavy 2017 & 2019 & 2020 | 0.11 (-0.29, 0.50) | 0.363 |
| #2: Lagerstand 2021 & Rosenqvist 2023 | 0.15 (-0.02, 0.32) | 0.061 |
| #3: Teichtahl 2015 | 0.12 (-0.16, 0.41) | 0.197 |
| #4: Zhang 2023 | 0.09 (-0.13, 0.30) | 0.228 |
| **Assumed correlation (restricted maximum likelihood)** |  |  |
| #5: ρ=0.0 | 0.12 (-0.03, 0.27) | 0.080 |
| #6: ρ=0.2 | 0.12 (-0.04, 0.28) | 0.092 |
| #7: ρ=0.4 | 0.12 (-0.04, 0.28) | 0.092 |
| #8: ρ=0.6 | 0.12 (-0.04, 0.28) | 0.104 |
| #9: ρ=1.0 | 0.12 (-0.05, 0.29) | 0.106 |
| **Normality assumption (robust variance)** |  |  |
| #10: ρ=0.8 | 0.11 (-0.02, 23) | 0.074 |

# Supplement K: Random-effects meta-analysis sensitivity analyses for secondary synthesis IVD degeneration according to average Pfirrmann grade compared to controls.

|  | Cross-sectional study sensitivity analysis | Hedges’ g (95%CI) | P-value |
| --- | --- | --- | --- |
|  | **Leave-one-out (restricted maximum likelihood; ρ=0.8)** |  |  |
|  | #1: Belavy 2017 & 2019 & 2020 | 0.15 (-0.22, 0.52) | 0.429 |
|  | #2: Benedikter 2022 | 0.31 (-0.13, 0.48) | 0.001 |
|  | #3: Cheng 2008 | 0.15 (-0.22, 0.52) | 0.435 |
|  | #4: Elfering 2002 | 0.20 (-0.18, 0.58) | 0.296 |
|  | #5: Maurer 2020 | 0.14 (-0.23, 0.51) | 0.467 |
|  | #6: Mitchell 2020 | 0.10 (-0.22, 0.42) | 0.545 |
|  | #7: Wegner 2023 | 0.21 (-0.16, 0.58) | 0.270 |
|  | **Assumed correlation (restricted maximum likelihood)** |  |  |
|  | #5: ρ=0.0 | 0.18 (-0.14, 0.50) | 0.272 |
|  | #6: ρ=0.2 | 0.18 (-0.14, 0.50) | 0.272 |
|  | #7: ρ=0.4 | 0.18 (-0.14, 0.50) | 0.272 |
|  | #8: ρ=0.6 | 0.18 (-0.14, 0.50) | 0.273 |
|  | #9: ρ=1.0 | 0.18 (-0.14, 0.50) | 0.278 |
| **Normality assumption (robust variance)** | |  |  |
| #10: ρ=0.8 | | 0.20 (-0.60, 0.21) | 0.270 |
|  | **Cohot/RCT with baseline as control sensitivity analysis** | **Hedges’ g (95%CI)** | **P-value** |
|  | **Leave-one-out (restricted maximum likelihood; ρ=0.8)** |  |  |
|  | #1: Horga 2022 | 0.20 (-4.52, 4.92) | 0.688 |
|  | #2: Shimozaki 2018 | -0.05 (-1.26, 1.16) | 0.690 |
|  | #3: Telles 2016 | 0.26 (-3.05, 3.56) | 0.504 |
|  | **Assumed correlation (restricted maximum likelihood)** |  |  |
|  | #5: ρ=0.0 | 0.14 (-0.79, 1.07) | 0.578 |
|  | #6: ρ=0.2 | 0.14 (-0.79, 1.07) | 0.578 |
|  | #7: ρ=0.4 | 0.14 (-0.79, 1.07) | 0.578 |
|  | #8: ρ=0.6 | 0.14 (-0.79, 1.08) | 0.576 |
|  | #9: ρ=1.0 | 0.14 (-0.79, 1.08) | 0.576 |
| **Normality assumption (robust variance)** | |  |  |
| #10: ρ=0.8 | | NA |  |

# Supplement L: Random-effects meta-analysis sensitivity analyses for secondary synthesis IVD degeneration according to prevalence of Pfirrmann grade >2 compared to controls.

| Cross-sectional study sensitivity analysis | OR (95%CI) | P-value |
| --- | --- | --- |
| **Leave-one-out (restricted maximum likelihood; ρ=0.8)** |  |  |
| #1: Benedikter 2022 | 1.14 (0.77, 1.69) | 0.520 |
| #2: Cheng 2008 | 1.44 (0.96, 2.18) | 0.078 |
| #3: Elfering 2002 | 1.35 (0.86, 2.14) | 0.196 |
| #4: Folkvardsen 2016 | 1.27 (0.78, 2.07) | 0.342 |
| #5: Hangai 2009 & Owen 2021 | 1.22 (0.76, 1.99) | 0.412 |
| #6: Identeg 2023 | 1.36 (0.89, 2.08) | 0.150 |
| #7: Kaneoka 2007 | 1.16 (0.74, 1.80) | 0.518 |
| #8: Koyama 2022 | 1.26 (0.78, 2.06) | 0.346 |
| #9: Kraft 2009 | 1.30 (0.81, 2.09) | 0.282 |
| #10: Ranson 2005 | 1.28 (0.79, 2.06) | 0.317 |
| #11: Witwit 2018 & 2019 | 1.21 (0.76, 1.93) | 0.427 |
| **Cohot/RCT with separate control sensitivity analysis** | **OR (95%CI)** | **P-value** |
| **Leave-one-out (restricted maximum likelihood; ρ=0.8)** |  |  |
| #1: Telles 2016 | 5.18 (1.34, 20.06) | 0.017 |
| #2: Witwit 2018 & 2022 | 0.72 (0.01, 39.52) | 0.874 |
| **Cohot/RCT with baseline as control sensitivity analysis** | **OR (95%CI)** | **P-value** |
| **Leave-one-out (restricted maximum likelihood; ρ=0.8)** |  |  |
| #1: Burnett 1996 | 1.07 (0.19, 5.93) | 0.936 |
| #2: Koyama 2022 | 1.52 (0.23, 9.87) | 0.663 |
| #3: Shimozaki 2018 | 1.13 (0.29, 4.43) | 0.866 |
| #4: Telles 2016 | 1.81 (0.92, 3.57) | 0.086 |
| #5: Witwit 2018 & 2022 | 1.36 (0.21, 8.84) | 0.748 |

# Supplement M: Random-effects meta-analysis sensitivity analyses for secondary synthesis according to prevalence of other measures of IVD compared to control.

| Signal intensity reduction, cross-sectional study sensitivity analysis | OR (95%CI) | P-value |
| --- | --- | --- |
| **Leave-one-out (restricted maximum likelihood; ρ=0.8)** |  |  |
| #1: Baranto 2009 | 2.89 (1.34, 6.23) | 0.022 |
| #2: Hellstrom 1990 & Sward 1991 | 2.30 (1.60, 3.30) | 0.005 |
| #3: Maurer 2011 | 2.88 (1.23, 6.76) | 0.029 |
| #4: Thoreson 2017 | 2.76 (1.10, 6.95) | 0.040 |
| #5: Witwit 2018 & 2019 | 3.44 (1.32, 8.92) | 0.026 |
| **Signal intensity reduction, cohort study with baseline control, sensitivity analysis** | **OR (95%CI)** | **P-value** |
| **Leave-one-out (restricted maximum likelihood; ρ=0.8)** |  |  |
| #1: Baranto 2006 | 1.72 (0.00, 807.22) | 0.463 |
| #2: Feuerriegel 2025 | 1.04 (0.45, 2.41) | 0.636 |
| #3: Witwit 2022 | 2.23 (0.03, 160.56) | 0.253 |
| **Any IVD height reduction, cross-sectional study sensitivity analysis** | **OR (95%CI)** | **P-value** |
| **Leave-one-out (restricted maximum likelihood; ρ=0.8)** |  |  |
| #1: Baranto 2009 | 38.03 (0.27, 5329.55) | 0.068 |
| #2: Thoreson 2017 | 4.35 (0.00, 8.96e+09) | 0.544 |
| #3: Witwit 2018 & 2022 | 6.41 (0.00, 1.87e+12) | 0.536 |
| **IVD height reduction >50%, cross-sectional study sensitivity analysis** | **OR (95%CI)** | **P-value** |
| **Leave-one-out (restricted maximum likelihood; ρ=0.8)** |  |  |
| #1: Granhed 1988 | 1.26 (0.27, 6.02) | 0.770 |
| #2: Hellstrom 1990 & Sward 1991 | 18.29 (8.80, 38.03) | 0.000 |

# Supplement N: Funnel plots for primary and secondary syntheses where n≥10


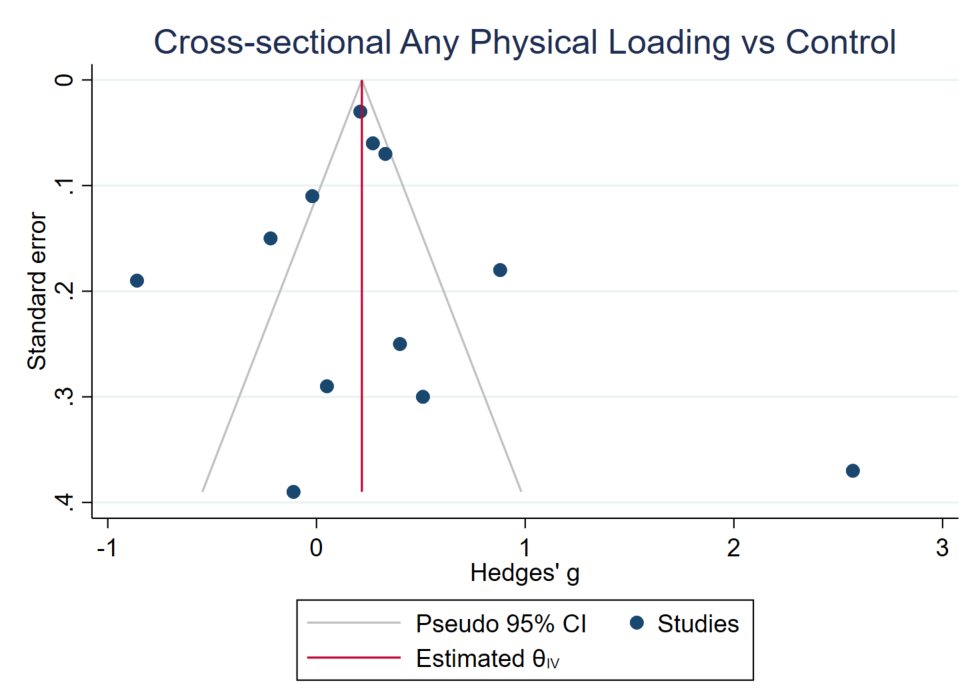


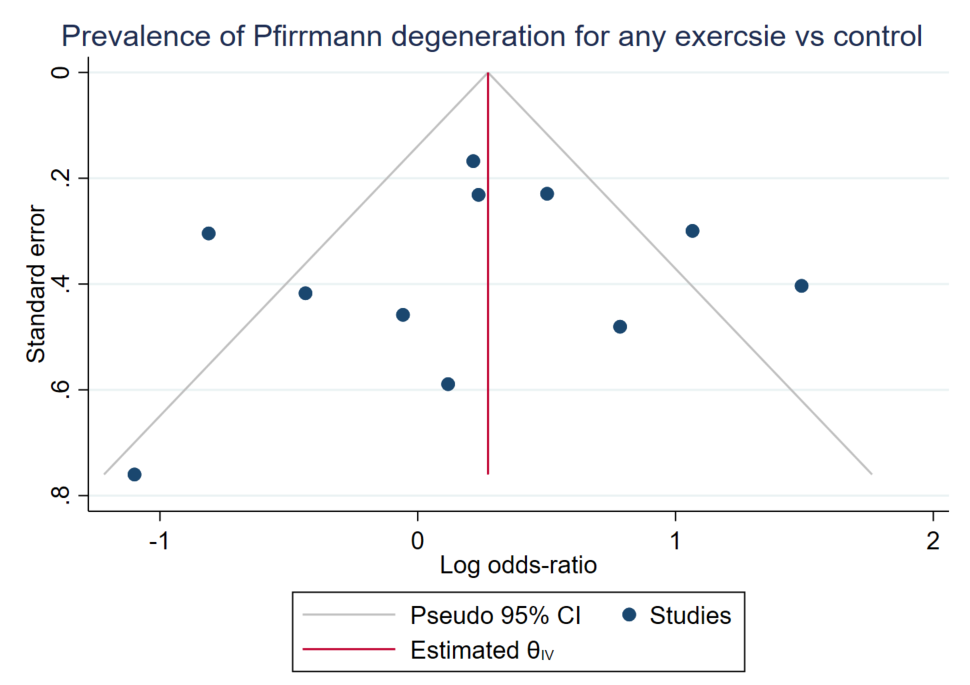


# Supplement O: Trim-and-fill funnel plots for primary and secondary syntheses where n≥10


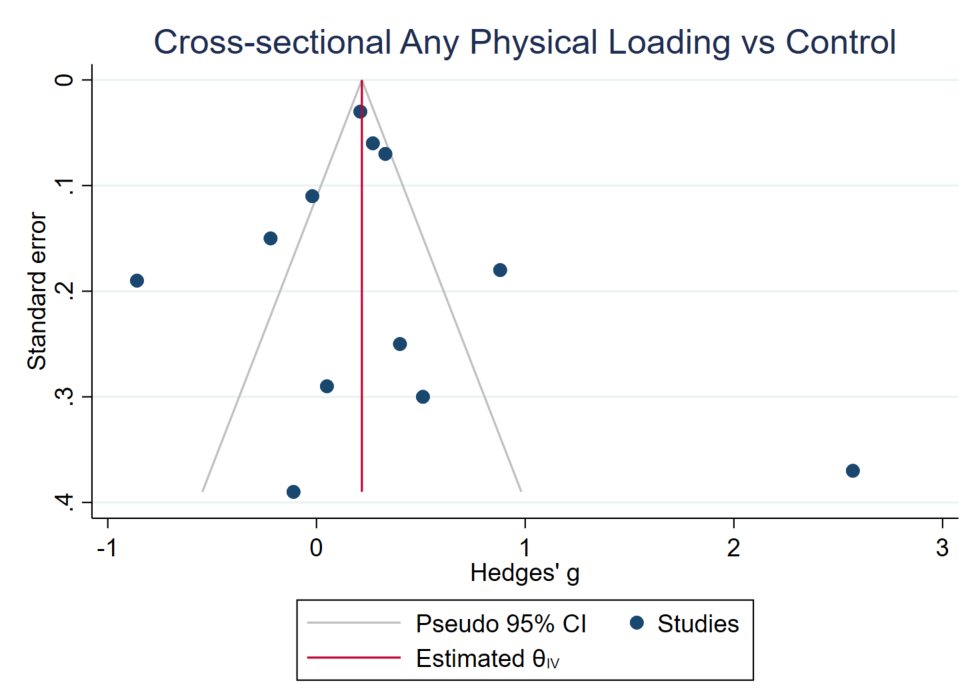


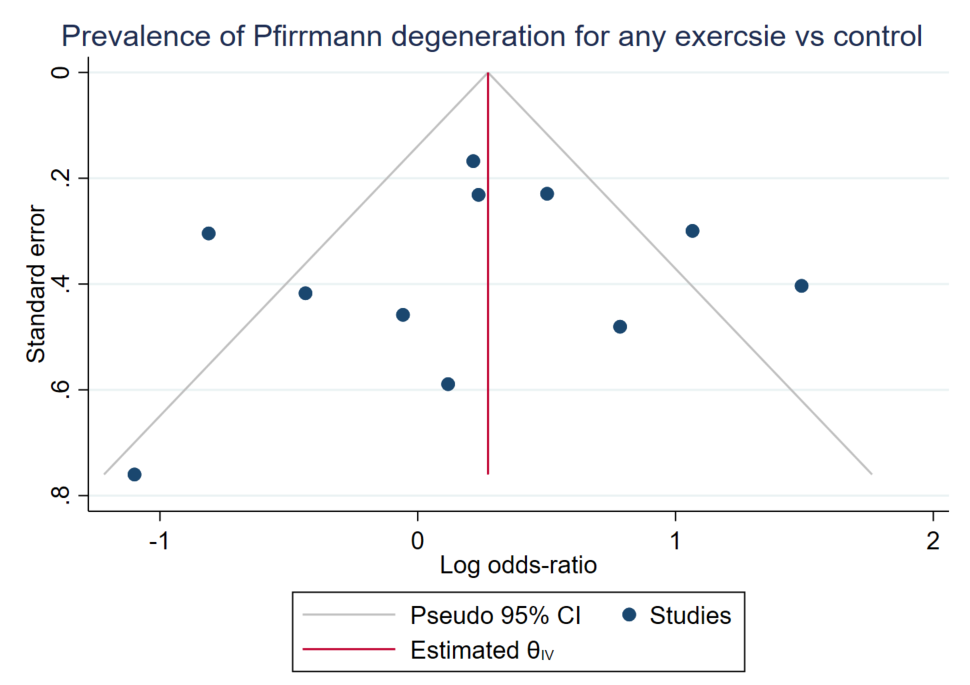


# Supplement P: Protocol amendments since registration

**Amendments to the search criteria**

**Exposure**

- McKenzie treatment will not be classified as an exercise intervention, therefore, these studies will be excluded
- Post-surgical and operational technique studies will be excluded given these studies normally do not include relevant outcomes (e.g. MRI data) in post-surgical rehabilitation

**Population**

- Patients with or without pain and all diagnoses will be included

**Outcomes**

- Only studies on lumbar spine and lower thoracic spine will be included

**Study**

- Case-control studies should be screened as they may contain cross-sectional data on the research topic, which will be included

**Planned aims and synthesis amendments**

- Protocol indicated pooling all exposures and other individual exposure in synthesis strategy however these were not considered as aims and the types of exposures not clarified. Therefore, further primary sub aims were added for combined physical loading, non-upright noncontact, extreme ranges of motion, aerobic and high vs low volume of the same physical loading exposure.
- Cohort studies <1 year follow-up were excluded from the cross-sectional analysis for clear distinction between intervention and control groups at one time point.

**Quality assessment criteria amendments**

Due to the subjective nature of the JBI tools, two reviewers (CLS, NKA) developed a criteria to ensure implementation of the checklists were standardised.

**JBI Critical Appraisal Checklist for analytical cross sectional studies**

1. **Were the criteria for inclusion in the sample clearly defined?**

Clearly identifies and reports inclusion and exclusion. No if unclear- lacking detail on criteria.

1. **Were the study subjects and the setting described in detail?**

Describes participants, demographics, location, time period of study

1. **Was the exposure measured in a valid and reliable way?**

No if unclear exposure of sport e.g. include no details on years, experience, level of experience

1. **Were objective, standard criteria used for measurement of the condition?**

Were groups similar at baseline according to age? No if age is statistically different or >10% difference

1. **Were confounding factors identified?**

If cofounding factors were identified anywhere in the paper e.g. methods, results or discussion

1. **Were strategies to deal with confounding factors stated?**

Confounding factors were addressed in analysis or reported in results section.

1. **Were the outcomes measured in a valid and reliable way?**

MRI is always valid. Reliable if they state inter-rater reliability was good of kappa above 0.6. If reliability not mentioned then should be unclear.

1. **Was appropriate statistical analysis used?**

Go with instinct and look for red flags.

**JBI Critical Appraisal Checklist for cohort studies**

Were the two groups similar and recruited from the same population?

1. **Clearly identifies and reports inclusion and exclusion and participant demographics similar across stratified groups.**

No if unclear- lacking detail on criteria

1. **Were the exposures measured similarly to assign people to both exposed and unexposed groups?**

No if unclear exposure of sport e.g. include no details on years, experience, level of experience

1. **Was the exposure measured in a valid and reliable way?**

No if unclear exposure of sport e.g. include no details on years, experience, level of experience

1. **Were confounding factors identified?**

If cofounding factors were identified anywhere in the paper e.g. methods, results or discussion

1. **Were strategies to deal with confounding factors stated?**

Confounding factors were addressed in analysis or reported in results section.

1. **Were the groups/participants free of the outcome at the start of the study (or at the moment of exposure)?**

No findings of degeneration at baseline or unclear degeneration no measured.

1. **Were the outcomes measured in a valid and reliable way?**

MRI is always valid. Reliable if they state inter-rater reliability was good of kappa above 0.6.- If reliability not mentioned then should be unclear.

1. **Was the follow up time reported and sufficient to be long enough for outcomes to occur?**

Yes, as per protocol, >1 week.

1. **Was follow up complete, and if not, were the reasons to loss to follow up described and explored?**

Yes, if follow up was complete or if incomplete and any detail provided on reasons why.

1. **Were strategies to address incomplete follow up utilized?**

Yes, if any mention of strategies to avoid incomplete follow up or address incomplete follow up.

1. **Was appropriate statistical analysis used?**

Go with instinct and look for red flags.
